# Hangprinter for large scale additive manufacturing using fused particle fabrication with recycled plastic and continuous feeding

**DOI:** 10.1016/j.ohx.2023.e00401

**Published:** 2023-02-09

**Authors:** Ravneet S. Rattan, Nathan Nauta, Alessia Romani, Joshua M. Pearce

**Affiliations:** a Department of Electrical & Computer Engineering, Western University, London, ON, Canada; b Department of Chemistry, Materials and Chemical Engineering (Giulio Natta), Politecnico di Milano, Milano, Italy; c Design Department, Politecnico di Milano, Milano, Italy; d Ivey Business School, Western University, London, ON, Canada

**Keywords:** Open hardware, 3D printing, FGF printing, Hangprinter, Recyclebot, Pellet extrusion, Waste plastic, Recycling, Open source hardware

## Abstract

The life cycle of plastic is a key source of carbon emissions. Yet, global plastics production has quadrupled in 40 years and only 9 % has been recycled. If these trends continue, carbon emissions from plastic wastes would reach 15 % of global carbon budgets by 2050. An approach to reducing plastic waste is to use distributed recycling for additive manufacturing (DRAM) where virgin plastic products are replaced by locally manufactured recycled plastic products that have no transportation-related carbon emissions. Unfortunately, the design of most 3-D printers forces an increase in the machine cost to expand for recycling plastic at scale. Recently, a fused granular fabrication (FGF)/fused particle fabrication (FPF) large-scale printer was demonstrated with a GigabotX extruder based on the open source cable driven Hangprinter concept. To further improve that system, here a lower-cost recyclebot direct waste plastic extruder is demonstrated and the full designs, assembly and operation are detailed. The <$1,700 machine’s accuracy and printing performance are quantified, and the printed parts mechanical strength is within the range of other systems. Along with support from the Hangprinter and DUET3 communities, open hardware developers have a rich ecosystem to modify in order to print directly from waste plastic for DRAM.

## Graphical abstract

**Figure:**
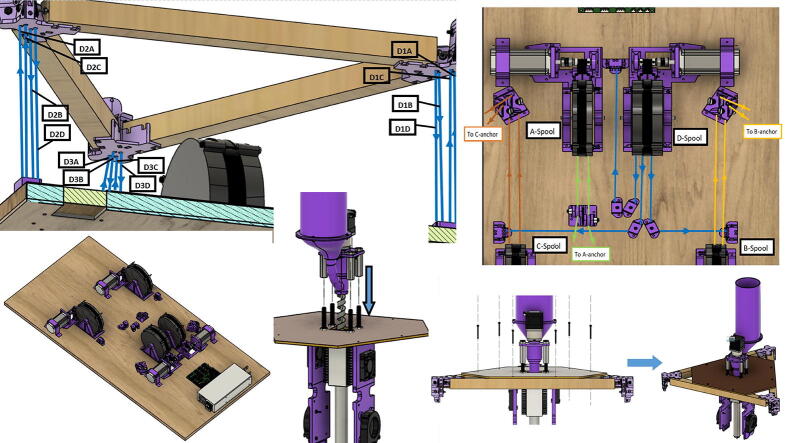

**Specifications table:** 

Hardware name	*Hybrid Hangprinter with Direct Drive Particle Extruder*
Subject area	• Engineering and Material Science
Hardware type	• Electrical engineering and computer science • Mechanical engineering and materials science
Open Source License	GNU General Public License v.3; CERN OHL v2
Cost of Hardware	CAD$2,125 (USD$1638)
Source File Repository	https://doi.org/10.17605/OSF.IO/NDWS6

## Hardware in context

In the race to reign in carbon emissions to halt climate destabilization, plastic – a key source of carbon emissions, is often ignored [1]. Yet, in the last 40 years, global plastics production has quadrupled, while only 9 % has been recycled [2]. If these trends are allowed to continue, the greenhouse gas (GHG) emissions from the global use and plastic wastes would reach 15 % of the global carbon budget by 2050 [3]. To control overall GHG emissions, it is important to develop strategies to mitigate the life-cycle GHG emissions of plastics [4]. Yet, it appears that the problem of our weak approach to plastic recycling [5] is compounded by the popularity of desktop consumer 3D printing, which both reduces consumers costs for plastic parts [6], [7], [8] and stimulates experimental design and innovations, while increasing the number of defective parts, failed prints, and discarded supports and thus waste plastic [9]. Although the first open source self-replicating rapid prototyper (RepRap) 3D printer [10], [11], [12] created an explosion of innovation and democratized additive manufacturing (AM) by radically reducing the costs, an increasingly significant amount of 3D printing plastic waste is landfilled creating locked in future GHG emissions [13].

A profitable and rapidly growing approach to reach a circular economy for plastics in the AM industry [14], [15] by increasing recycling rates is distributed recycling for additive manufacturing (DRAM) [16], [17]. Prosumers (a portmanteau of producing consumers) have a substantial economic incentive [14] based on savings for recycling with DRAM, as opposed to the traditional recycling models where there is often no economic incentive for consumers to recycle plastic [17]. In the DRAM model, prosumers utilize their own plastic waste as raw material for AM feedstocks (a relatively high value for plastic at $20 per 1 kg spool of filament). In the most environmentally-friendly version of DRAM, which reduces embodied energy and GHG emissions of products [18], [19], prosumers manufacture their own products from the 3D printing feedstocks directly in their own homes [20]. Thus, DRAM is a means of GHG emissions reduction because virgin plastic products are replaced by locally manufactured recycled plastic products (that cut almost all GHG emissions from transportation). DRAM is applicable everywhere globally including areas that are not currently serviced by recycling and in the most extreme cases electricity [21], [22]. DRAM thus has the potential to radically impact global value chains already being impacted by more localized manufacturing with 3-D printing [23].

DRAM research has been evolving quickly but has primarily focused on using some form of recyclebot (waste plastic extruder) [24], [25] to provide raw materials for fused filament fabrication (FFF) (the open source version of the patented fused deposition modeling (FDM)) used for low-cost RepRap-class 3-D printers that now represent the majority of the 3-D printers on the market. A more efficient approach to DRAM has recently been developed using direct extrusion of ground waste plastic in 3-D printing called fused granular fabrication (FGF)/ fused particle fabrication (FPF) [26], [27], [28], [29], [30]. Although FPF/FGF is possible on the desktop [31], it is extremely challenging and more easily applied to large plastic products [32]. From a GHG emissions perspectives, large plastic products that have a long lifecycle (e.g. furniture or building components) provide a long-lifetime carbon sink and have the potential to use large quantities of plastic waste.

Unfortunately, the standard mechanical design of most FFF/FDM 3-D printers limits the size of the print, and forces an increase in the machine size and cost to expand the volume of the printed parts. Today's high-volume 3-D printers are expensive (e.g., The BOX Large by BLB Industries costs $298,000 [33] and even the open source offerings from re:3D of the Gigabot XLT cost $17,000 [34]. Recently, however, Petsiuk et al. [35] demonstrated the potential to make a FGF/FPF large scale printer with a GigabotX extruder based on the open source cable driven Hangprinter concept [36]. This radically reduced the cost of large-scale printing below $5,000 and offers the potential to make an impact on GHG emissions be allowing large-scale recycled plastic waste printing at more accessible capital costs.

Torbjørn Ludvigsen started the open source Hangprinter project in November 2013 in Umeå, Sweden, being inspired by the RepRap project [36]. Such wire, or cable robots have been well described in the literature over the past decade or two [37], [38], [39], [40], [41], [42], [43] and there are other large-scale experimental printers [44], [45], [46]. In this article, the hybrid approach that Petsiuk used based on Ludvigsen’s Hangprinter design is further developed to use a lower-cost recyclebot-like direct waste plastic extruder and the full designs [47], assembly and operation are detailed.

## Hardware description

The proposed open source Hangprinter is a hybrid design based on the Hangprinter V3 and V4 [48], [49], [50] systems developed by original inventor, Torbjørn Ludvigsen. Utilizing open source mechanical and electrical components, this hybrid system consists of three parts, 1) pellet/granule feeding system, 2) FPF/FGF extruder system and 3) Hangprinter end effector. These three components combine to realize a closed loop solution for DRAM all while achieving lower costs compared to other FGF/FPF printers on the market, scalable build volume and frameless design. This machine can be built for under USD$2000. The electronics are controlled by the popular DUET3 controller and RepRap firmware. This hardware and firmware combination offers more powerful and robust control compared to most hobby level RepRap printers, which use an 8-bit RAMPS board with Marlin firmware. Coupled with an easy-to-use user interface, frequent firmware improvements and active community, this hybrid Hangprinter design is more reliable and easily upgradable for any future projects.

### Hangprinter

**Fig. 1:**
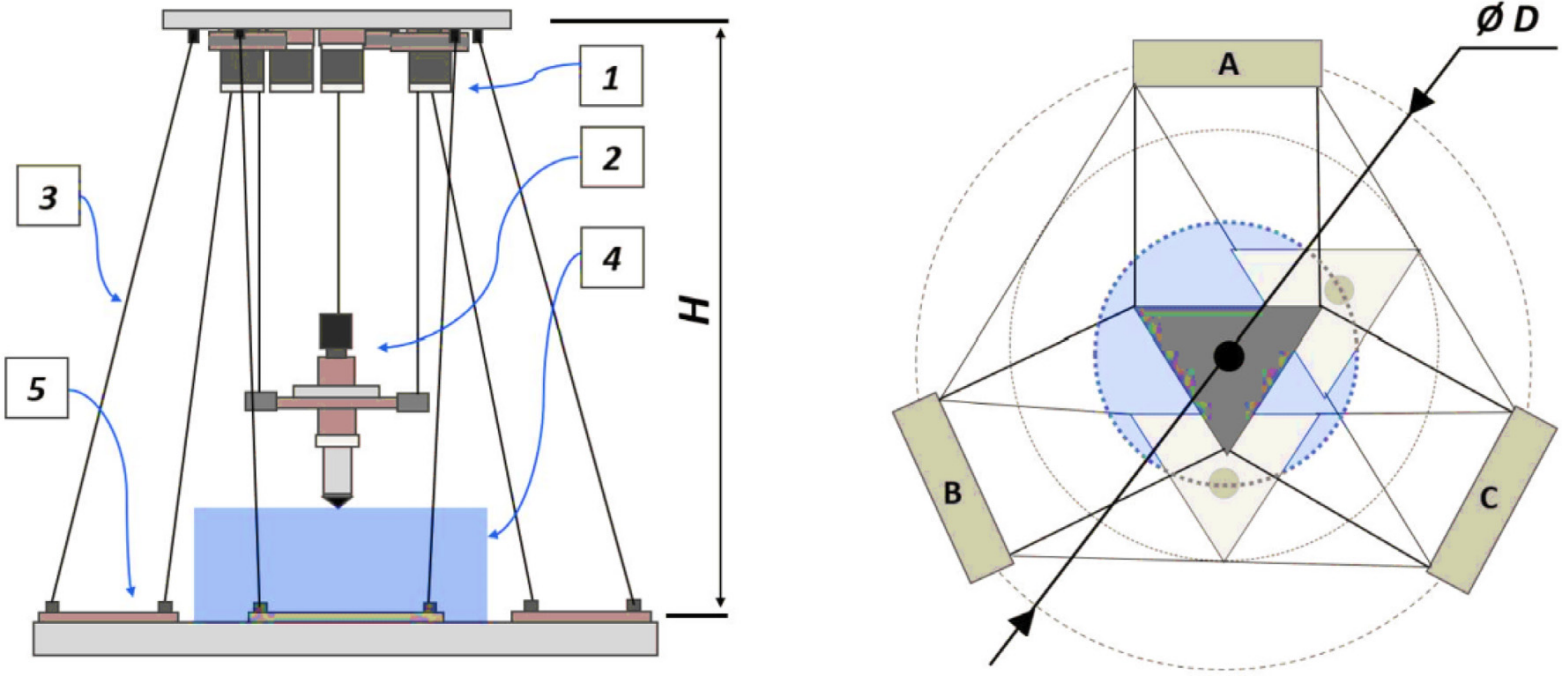
Waste plastic FGF/FPF Hangprinter diagram .

The primary motion system used for this project is the Hangprinter and its unique kinematics, which. can be classified as a redundantly actuated, 3°-of-freedom parallel cable driven robot (CDPR) [41]. Due to a frameless design, it is scalable and relatively low cost compared to large gantry machines making it an accessible solution for DRAM when coupled with the concept of FGF/FPF printing using recycled feedstock [35]. The Hangprinter consists of 4 local anchors (3 on the ground and 1 on ceiling), which control movement of the Hangprinter end effector in 4 local axes (A, B, C and D axis) shown in Fig. 1:

1. Ceiling unit housing controls and 4 motors for A, B, C and D axes.
2. End effector with FGF/FPF extruder.
3. A, B, C, and D lines/cables.
4. Scalable, conical build volume.
5. Print bed and ground anchors.

adapted from [35]

The FPF/FGF Hangprinter has an outer diameter *D*.

**Fig. 2:**
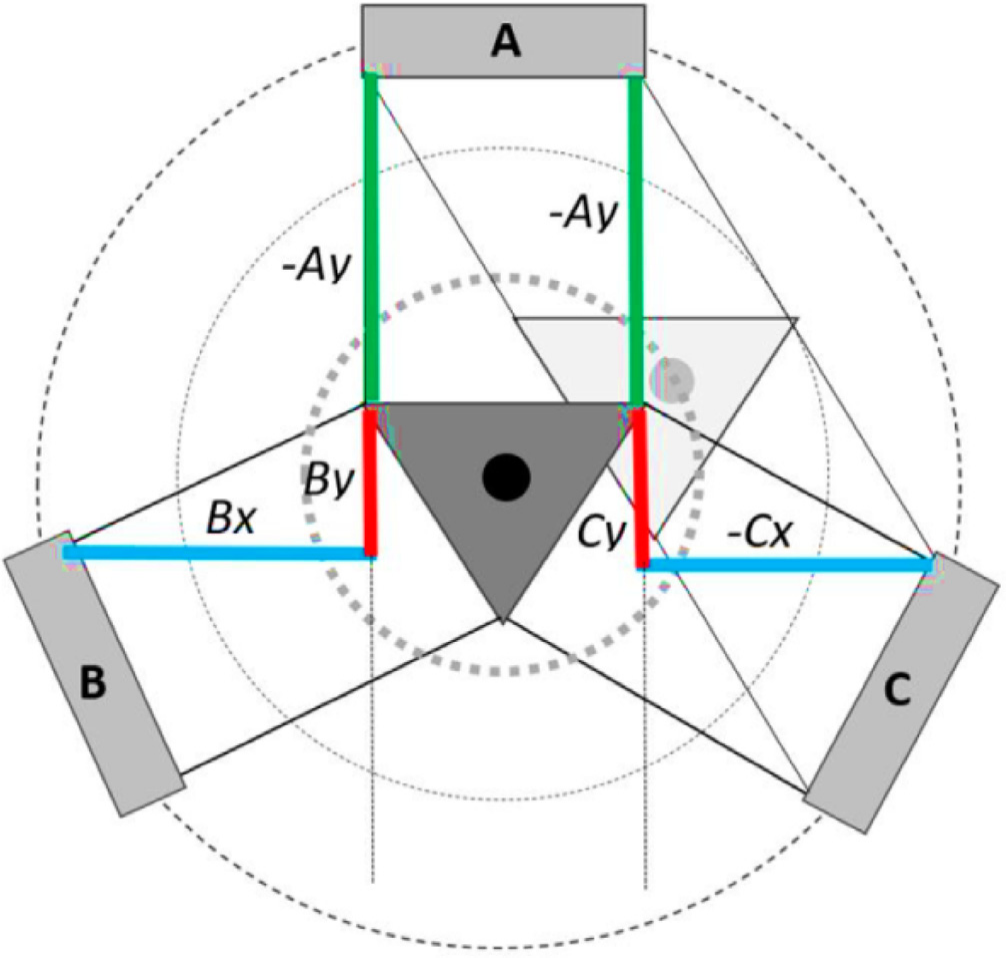
X and Y components of A, B, C anchors in the hangprinter geometry [35].

The A, B, C axes allow the end effector to move in plane while the D axis moves the end vertically. Parallel wires prevent the end effector from rotating around its own axis while moving along a given G-code path [35]. Through the unique Hangprinter kinematics, inverse and forward transforms allow conversion from cartesian coordinates to local joint angles and vice versa respectively. The firmware operates on the premise of line lengths to calculate and perform movement in cartesian space using the position of the end effector relative to each anchor based on the Pythagorean norm (line length). The firmware uses these equations ((1), ((2) and ((3) and Fig. 2 in cutting G-code trajectories into straight line segments along the XY plane.

(1) $$l_{A}=\sqrt{{\left(P_{X}-A_{X}\right)}^{2}+{\left(P_{Y}-A_{Y}\right)}^{2}}$$

(2) $$l_{B}=\sqrt{{\left(P_{X}-B_{X}\right)}^{2}+{\left(P_{Y}-B_{Y}\right)}^{2}}$$

(3) $$l_{C}=\sqrt{{\left(P_{X}-C\right)}^{2}+{\left(P_{Y}-C_{Y}\right)}^{2}}$$

where $l_{A}$, $l_{B}$ , $l_{C}$ are line segments for the corresponding anchors, ($P_{X}{,P}_{Y}$) are the coordinates of the end effector, and (AX,AY, BX,BY, CX,CY) are coordinates of the anchors A, B, and C, respectively [35].

**Fig. 3:**
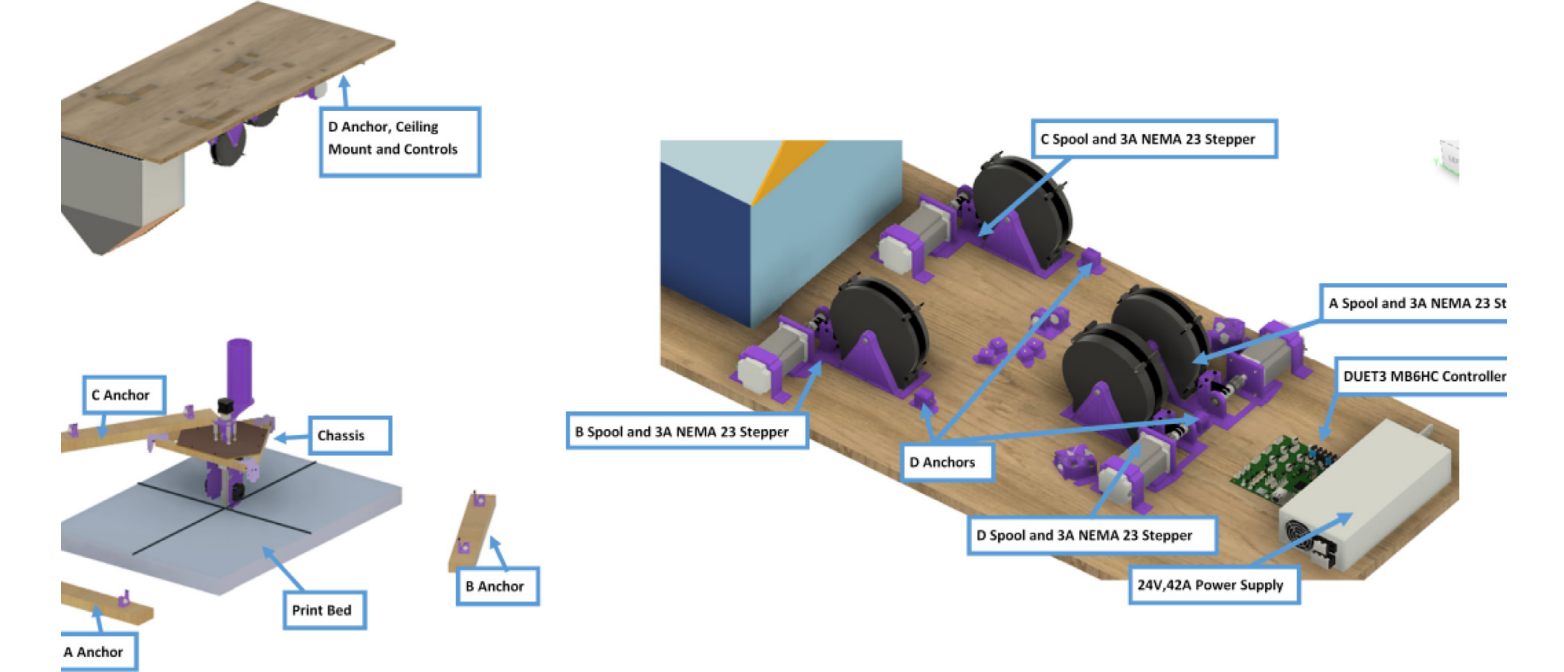
FGF/FPF hybrid hangprinter render.

The hybrid Hangprinter shown in Fig. 3 developed in this project offers the following modifications and improvements to the hybrid Hangprinter V3 developed by Petsiuk et al. [35]:

• 32bit, ARM based microcontroller (DUET3 MBHC).
• Integrated motor control of NEMA 23 motors by the DUET3 board reduces overall footprint and complexities in wiring to provide a more plug and play solution.
• Easier to use native graphic user interface over ethernet providing more control to researchers and users.
• Doubled lines and spools along with stronger cabling to support a heavier end effector.
• Use of timing belts instead of gears reduces backlash in system.
• Better mechanical assembly from the Hangprinter V4 while still using the familiar and easier to use stepper motors from Hangprinter V3 for 3D printing.

### Pellet extruder

**Fig. 4:**
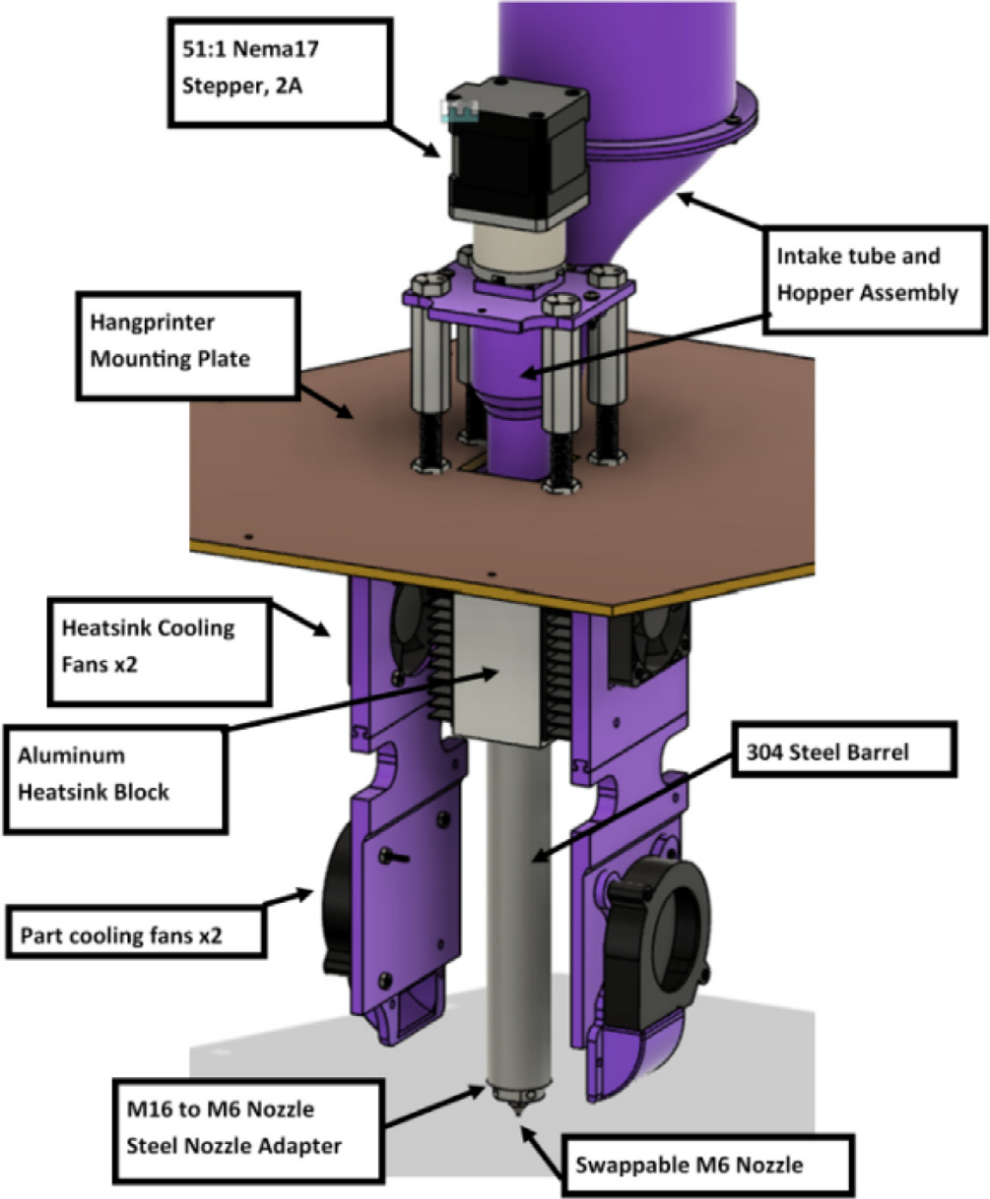
Pellet extruder details.

The direct pellet extruder design is partly based on the design of the recyclebot [25]. This extruder was designed with weight, modularity, and simplicity in mind. This direct pellet extruder features a geared NEMA 17 stepper motor, 5/8″ wood auger as the extrusion screw, a steel barrel, an aluminum heat dissipation block and heatsinks, a 100 W, 120VAC band heater, a pt1000 RTD, passive cooling through axial and radial blower fans, printed support frame with interchangeable hopper and swappable nozzle adapters and M6 nozzles (Fig. 4). All electronics connect to the DUET3 controller located in the ceiling mount of the Hangprinter via a breakout board. This breakout board allows for easy swapping of mechanical components as well as easily reaching all connections. While this extruder retains primary elements of the Recyclebot, some components are not off the shelf, but are still simple to fabricate. Due to the inherent design challenges associated with a large scale, cable suspended printer, weight is of high importance. The end effector must be as light weight as possible to reach appreciable print speeds and accelerations. This in turn, requires some parts to be more customized and optimized than simple off the shelf components. Detailed instructions and alternate modes of manufacturing have been provided for those parts. This extruder operates on a drip-feeding mechanism and a non-compression screw extruder with a lighter NEMA17 stepper motor. This is to ensure that weight is minimal on the Hangprinter end effector while still capable of extruding at appreciable flow rates. While using an off-the-shelf auger is not an ideal extrusion screw due to its lack of compression and pressure build up, pellet extruders in the academic and maker space such as those developed by Whyman [30], Drotman [51] and Richrap3d [52] have validated the use of these screws in pellet extruders. Some differences of this extruder compared to these extruders are that this extruder:

• Has a single zone 100 W AC band heater capable of reaching stable temperatures up to 300 °C rather than using heating cartridges, which are not efficient for heating cylindrical surfaces or DC heating which consumes more current.
• Use a lighter, 51:1 geared NEMA 17 instead of a larger NEMA23 motor.
• Print at higher flow rates of approximately 0.5 kg/hr.
• Print large scale objects faster with 1–3 mm nozzle.
• More modular and easily configurable and upgradable (i.e. adding more heaters, heatsinks, bigger fans, and nozzles).

The primary feature of this extruder is the use of a powerful 100 W, 120Vac band heater. This is more efficient than using typical 12–24 V DC cartridges, which consume more current and are not suitable at providing sufficient heating for a large amount of plastic at higher flow rates. Another main feature of the pellet extruder is that it can allow for a wide range of popular 3-D printer nozzles [53] including the popular E3D V6 and MK8 series nozzles [54]. Any nozzle with the standard M6 thread specification can be used.

### Pellet feeding system

**Fig. 5:**
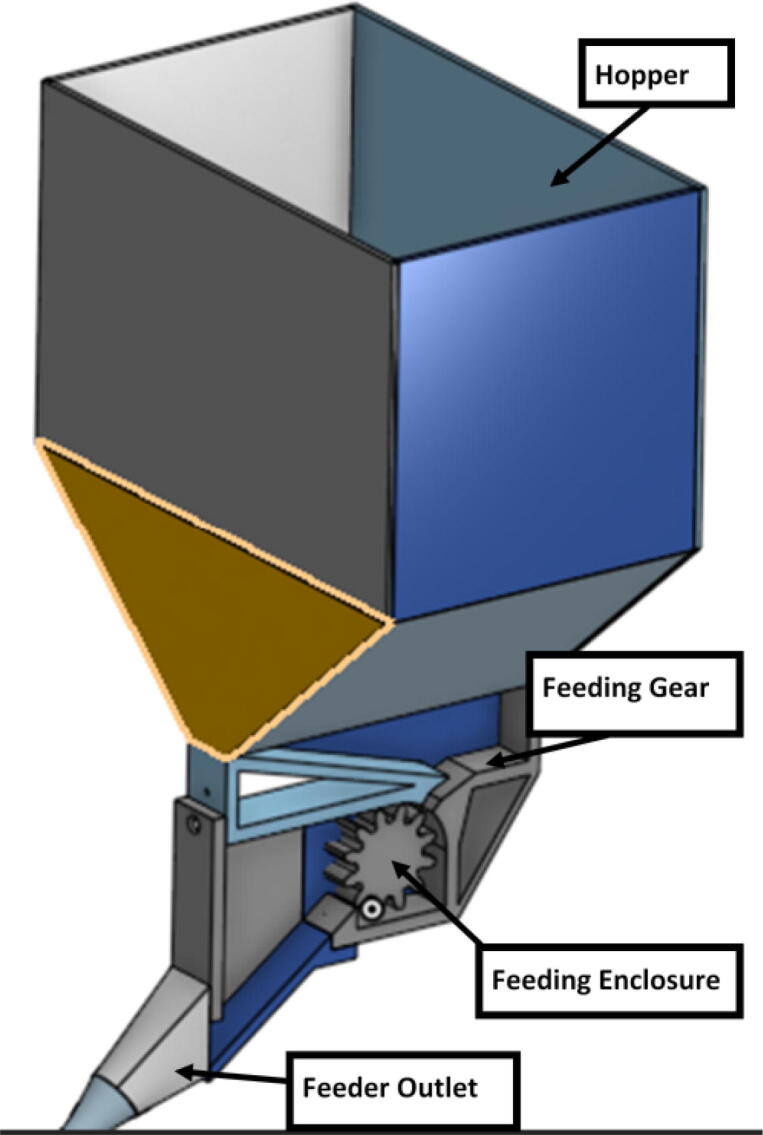
Pellet Feeder Details.

The pellet feeding system (Fig. 5) is designed to store up to 5 kg of pellets, particles or granules in a hopper. It is constructed entirely out of 3-D printed and off-the-shelf components. Utilizing a gravity-based mechanism, pellets are released from the large hopper via controlled rotation of a gear. Pellets are fed to the extruder by way of a flexible duct tube. Finally, pellets are synchronously released to the extruder as needed via the DUET3 controller’s “mixing” extruder functionality. An advantage of this functionality is that it is slicer independent the user does not need to concern themselves change any slicer settings to accommodate for the 2nd “pseudo extruder” system. This is the primary difference compared to other open source pellet feed systems such as the Auto Pellet Dispenser for the Lily Pellet Extruder [55] and the Automatic Pellet Feeding System for the DYZE Pulsar Pellet Extruder [56] where these extruders have an external controller and hardware that feed pellets automatically to the extruder. Another advantage is that it achieves a drip feeder functionality such as the one used in [30] without the need of any additional feedback sensors, etc. This gives the user more control over the flow of pellets and granules in real time via sliders for various feedstock geometries.

### DUET3 controller and electronics

**Fig. 6:**
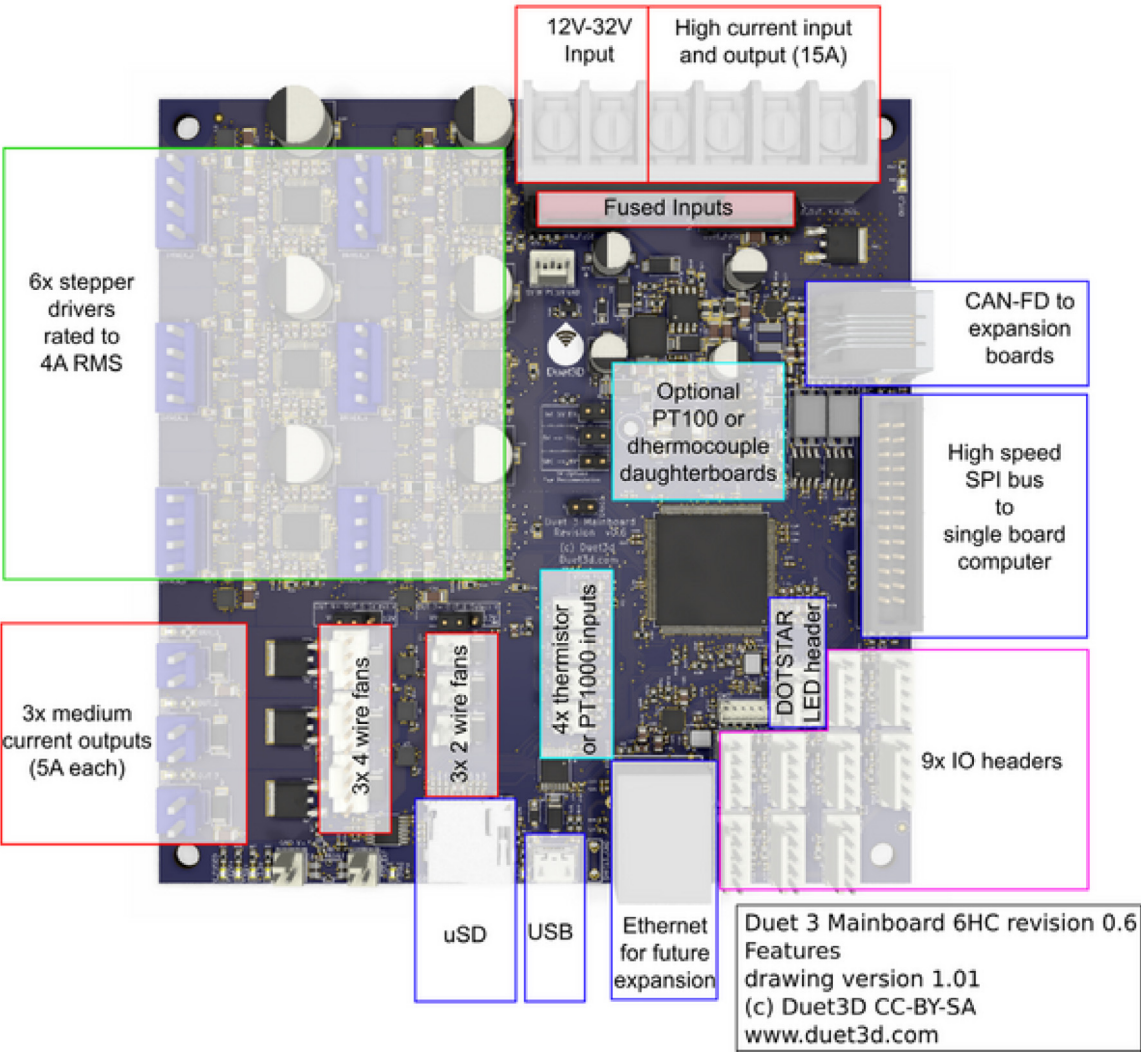
DUET3 Specifications [57].

The microcontroller used in this design is the DUET3 MB6HC board (Fig. 6) [57]. It runs a C++ based RepRap firmware and consists of a powerful 32-bit MCU with high power heater outputs, stepper drivers and other input and output peripherals. All electronics are controlled via the DUET3 controller and are easily configured in a “config.g” file via G-code. The entire system is powered by a 24 V power supply except for the heaters, which are powered by 120Vac via a solid-state relay. The DUET3 is a powerful, high end open source 3-D printer controller that has the following features:

• Has a more powerful 32-bit ARM MCU compared to 8bit RAMPS boards that use Marlin firmware.
• Has an easy-to-use web GUI.
• Easy to use configuration file with all configurations done using G-code; no C/C++ programming knowledge required.
• Strong documentation and community knowledge base.
• Continuous improvements in firmware with new features every release.

Finally, as the whole system, the FGF/FPF style open source Hangprinter:

• Enables both researchers and prosumers to mix different plastics together and print objects directly for material science studies, thus enabling composite waste plastic 3-D printing (e.g., waste wood and plastic [58]).
• Allows for inexpensive repair, easy swapping of parts and modifications to suit the operator’s/researcher’s needs due to the extruder’s modular assembly.
• Reduces costs of 3-D printing by skipping associated costs of producing filament in house as well as extending the lifetime of the plastic by avoiding one melt/solidification cycle.
• Provides a method of large-scale and scalable DRAM in the lab, makerspace, fablab, library and small and medium size enterprise environment that offers the potential to collectively reduce GHG emissions associated with plastic products.
• Allows users easy control via ethernet based interface to monitor and control printer in real time.

For the remainder of the paper the open source hybrid waste plastic direct extrusion Hangprinter system will be referred to as the Hangprinter Hybrid v4.

## Design files

A summary of all design files involved in this project are shown below in Table 1. All files are in [47].

**Table 1:** Design files summary.

System	Design File Name	File Type	Open Source License	Picture of Part
Hangprinter	ABC_motor_mount_spool	.STEP,.STL	GNU GPL v3.	
Hangprinter	bearing_u_608	.SCAD,.STEP,.STL	GNU GPL v3.	
Hangprinter	bearing_u_623	.SCAD,.STEP,.STL	GNU GPL v3.	
Hangprinter	belt_roller_insert_extended	.SCAD,.STEP,.STL	GNU GPL v3.	
Hangprinter	ChassisBeam	.SCAD,.STEP,.STL	GNU GPL v3.	
Hangprinter	ChassisBeam Drawing	.F2D,.DWG,.PDF	GNU GPL v3.	N/A
Hangprinter	corner_clamp	.SCAD,.STEP,.STL	GNU GPL v3.	
Hangprinter	D_motor_mount_spool	.STEP,.STL	GNU GPL v3.	
Hangprinter	dleft_spool	.SCAD,.STEP,.STL	GNU GPL v3.	
Hangprinter	dleft_spool_cover	.SCAD,.STEP,.STL	GNU GPL v3.	
Hangprinter	dright_spool_bottom	.SCAD,.STEP,.STL	GNU GPL v3.	
Hangprinter	dright_spool_cover	.SCAD,.STEP,.STL	GNU GPL v3.	
Hangprinter	dright_spool_top	.SCAD,.STEP,.STL	GNU GPL v3.	
Hangprinter	GT2_spool_gear_double	.SCAD,.STEP,.STL	GNU GPL v3.	
Hangprinter	horizontal_line_deflector	.SCAD,.STEP,.STL	GNU GPL v3.	
Hangprinter	line_roller_anchor_half_tilt	.SCAD,.STEP,.STL	GNU GPL v3.	
Hangprinter	line_roller_anchor_half_tilt_mirrored	.SCAD,.STEP,.STL	GNU GPL v3.	
Hangprinter	line_roller_anchor_straight	.SCAD,.STEP,.STL	GNU GPL v3.	
Hangprinter	line_roller_anchor_template	.SCAD,.STEP,.STL	GNU GPL v3.	
Hangprinter	line_roller_double	.SCAD,.STEP,.STL	GNU GPL v3.	
Hangprinter	line_verticalizer	.SCAD,.STEP,.STL	GNU GPL v3.	
Hangprinter	motor_holder	.SCAD,.STEP,.STL	GNU GPL v3.	
Hangprinter	sep_disc	.SCAD,.STEP,.STL	GNU GPL v3.	
Hangprinter	spool	.SCAD,.STEP,.STL	GNU GPL v3.	
Hangprinter	spool_cover	.SCAD,.STEP,.STL	GNU GPL v3.	
Hangprinter	spool_cover_mirrored	.SCAD,.STEP,.STL	GNU GPL v3.	
Hangprinter	tilted_line_deflector	.SCAD,.STEP,.STL	GNU GPL v3.	
Hangprinter	tilted_line_deflector_mirrored	.SCAD,.STEP,.STL	GNU GPL v3.	
Hangprinter	ziptie_tensioner_wedge	.SCAD,.STEP,.STL	GNU GPL v3.	
Pellet Extruder	6015 Fan Shroud	.SLDPRT,.STEP,.STL	GNU GPL v3.	
Pellet Extruder	Aluminum_Heatsink_Block	.SLDPRT,.STEP	GNU GPL v3.	
Pellet Extruder	Barrel Bracket Base	.SLDPRT,.STEP,.STL	GNU GPL v3.	
Pellet Extruder	Barrel Bracket Wing 1	.SLDPRT,.STEP,.STL	GNU GPL v3.	
Pellet Extruder	Barrel Bracket Wing 2	.SLDPRT,.STEP,.STL	GNU GPL v3.	
Pellet Extruder	Blower Fan Mount	.STEP,.STL	GNU GPL v3.	
Pellet Extruder	Blower Fan Plate	.SLDPRT,.STEP,.STL	GNU GPL v3.	
Pellet Extruder	Chassis Mount Frame	.SLDRW,.DXF,.PDF	GNU GPL v3.	
Pellet Extruder	IntakeTube	.SLDPRT,.STEP,.STL	GNU GPL v3.	
Pellet Extruder	IntakeTube_Extension	.SLDPRT,.STEP,.STL	GNU GPL v3.	
Pellet Extruder	IntakeTube_Hopper	.SLDPRT,.STEP,.STL	GNU GPL v3.	
Pellet Extruder	Motor Mount Square Bracket	.SLDPRT,.STEP,.STL	GNU GPL v3.	
Pellet Extruder	Steel_Barrel	.SLDPRT,.STEP	GNU GPL v3.	
Pellet Feeder	Feed Gear Enclosure	.SCAD,.STEP,.STL	GNU GPL v3.	
Pellet Feeder	Feeding Tube Coupler	.SLDPRT,.STEP,.STL	GNU GPL v3.	
Pellet Feeder	Feeder Motor Enclosure	.SLDPRT,.STEP,.STL	GNU GPL v3.	
Pellet Feeder	Feeder Outlet	.SLDPRT,.STEP,.STL	GNU GPL v3.	
Pellet Feeder	Feeding Gear	.SLDPRT,.STEP,.STL	GNU GPL v3.	
Pellet Feeder	Hopper Corner Bracket	.SLDPRT,.STEP,.STL	GNU GPL v3.	
Pellet Feeder	Hopper Panel Bracket	.SLDRW,.DXF,.PDF	GNU GPL v3.	
Pellet Feeder	Hopper Rectangle Panel	.SLDRW,.DXF,.PDF	GNU GPL v3.	
Pellet Feeder	Hopper Rectangle2 Panel	.SLDRW,.DXF,.PDF	GNU GPL v3.	
Pellet Feeder	Hopper Rectangle3 Panel	.SLDRW,.DXF,.PDF	GNU GPL v3.	
Pellet Feeder	Hopper Trapezoid Panel	.SLDRW,.DXF,.PDF	GNU GPL v3.	

## Bill of materials

All components used to assemble the Hangprinter system, pellet extruder and pellet feeder can be found in the “BOM” folder in [47] and are listed in Table 2. While every attempt is made to use metric units where possible, there are components in the design files as well as purchased components that are specified with imperial units. Use of metric and imperial units are clearly mentioned in the build instructions. All STL files for the Hangprinter and pellet extruder (except for threaded features on the pellet extruder) are in millimeters (mm).

**Table 2:** Bill of materials.

**System**	**Source**	**Component**	**Quantity**	**Cost/unit-CAD $**	**Total Cost-CAD $**
Hangprinter	Amazon	Bergen Industries 3-Wire Power Tool Cord	**1**	**$9.80**	$9.80
Hangprinter	OZRobotics	NEMA 23 Motors (3Nm) (200 step)	4	$40.70	$162.80
Hangprinter	Ali Express	Dorisea Spectra Line (300 m 1.4 mm/400lbs)	1	$27.42	$27.42
Hangprinter	Amazon	Eyelets 100-pack	1	$14.98	$14.98
Hangprinter	Amazon	Shaft Couplings 8 mm to 10 mm 3-pack	2	$15.00	$30.00
Hangprinter	Amazon	8 mm Steel Rod (650 mm length)	1	16.99	$16.99
Hangprinter	Amazon	610 mm GT2 Rubber timing Belts −5 pack	2	19.99	$39.98
Hangprinter	Amazon	20 tooth GT2 Gear- 5 pack	2	10.99	$21.98
Hangprinter	Amazon	608 (608ZZ) Bearings- 10 pack	3	$9.99	$29.97
Hangprinter	Amazon	623 (623ZZ) Bearings-20 pack	2	$10.40	$20.80
Hangprinter	Amazon	623 (F623ZZ) or F695-2RS Flanged Bearings −10 pack	2	$9.99	$19.98
Hangprinter	Rona	MDF Board 1″ thick 5ftx3ft	1	$89.99	$89.99
Hangprinter	Digikey	M8 bolts	15	$0.59	$8.85
Hangprinter	Digikey	M8 nuts	15	$0.24	$3.60
Hangprinter	Digikey	M3 nuts	150	$0.06	$9.68
Hangprinter	Digikey	M3x16mm Hex nylon screws	50	$0.14	$7.00
Hangprinter	Digikey	M3x25mm Hex nylon screw	6	$0.24	$1.44
Hangprinter	Amazon	M3 Hex Socket Cap Screws Kit(30 mm − 50 mm) (50)	1	$29.19	$29.19
Hangprinter	Digikey	M3x42mm Bolts Nylon (42 mm) (25)	25	0.16	$4.00
Hangprinter	Home Depot	#5x3/4 Flat Head Wood Screw − 28 pack	3	$3.47	$10.41
Hangprinter	Amazon	Zipties (16 Heavy Duty, 20 assorted)	1	$9.99	$9.99
Hangprinter	Lowes	1″x36″ Square Poplar Wood Dowel	2	$7.99	$15.98
Hangprinter	Home Depot	2″by4″by8′ SPF Dimensional Lumber	1	$5.89	$5.89
Hangprinter	Amazon	8x10mm Flexible Coupling Jaw Shaft Coupler(3pack)	2	$15.00	$30.00
Hangprinter	Home Depot	#8 × 5/8-inch Round Head Square drive WoodScrews −100pack	1	$16.40	$16.40
					
Extruder	OMC-Stepperonline	Nema 17 Geared 51:1 Stepper Motor	1	$27.68	$27.68
Extruder	Amazon	UEETEK Flat Ribbon Cable Multiple 10 Conductors	1	$14.99	$14.99
Extruder	Grainger	TEMPCO BAND HEATER,900 DEG F,120 V,1 IN. DI	1	$42.34	$42.34
Extruder	Amazon	Bestgle 560pcs JST Connector Kit	1	$18.59	$18.59
Extruder	McMaster-Carr	Stainless Steel Round Tube, 0.188″ Wall Thick, 1ft	1	$63.44	$63.44
Extruder	Metal Supermarkets	Aluminum Square Bar 6061 T6511 1.500, 4″ Length	1	$13.30	$13.30
Extruder	Edmund Optics	M6 × 1.0 Female to M16 × 1.0 Male Adapter	1	$18.23	$18.23
Extruder	Ali Express	Stainless Steel Nozzles (E3D M6 clone)	1	$15.99	$15.99
Extruder	Lee Valley Tools	5/8″ x 18″ HCS Auger Bit	1	$28.60	$28.60
Extruder	Walmart	8mmx12mm motor shaft coupling	1	$13.99	$13.99
Extruder	Slice Engineering	RTD Pt1000	2	$40.00	$80.00
Extruder	Amazon	Thermal Adhesive Tape	1	$13.99	$13.99
Extruder	Newark	Heat Sinks	6	$2.37	$14.22
Extruder	Digikey	5015 Axial Fan	2	$17.13	$34.26
Extruder	3D Printing Canada	6015 Blower Fan	2	$14.95	$29.90
Extruder	Home Depot	5/16″-18 Hex Drive Screws, 1″ Length	4	$0.60	$2.40
Extruder	Lowes	5/16″-18 Oval head Screws − 5 pack	4	$13.49	$53.96
Extruder	Home Depot	5/16″-18 thread standoff, 1.75″	4	$1.48	$5.92
Extruder	Home Depot	5/16″-18 Hex Nuts	4	$0.28	$1.12
Extruder	Amazon	M2 M3 M4 Phillips Pan Head Screws and Nuts Assortment Kit	1	$18.99	$18.99
Extruder	Amazon	Inkbird SSR-40 DA, 40A −2 pack	2	$15.49	$30.98
Extruder	Amazon	Assorted 14-18AWG Wire Spools, 100′ each	1	$45.95	$45.95
Extruder	Ebay	Adafruit Universal Perf Board (10pck)	1	$10.00	$10.00
Extruder	Amazon	MEANWELL SE-1000–24	1	$280.00	$280.00
Extruder	Digikey	250 V, 2.1A Fuse, 5mmx20mm	2	$0.30	$0.60
Extruder	Digikey	Keystone, 5x20mm fuse holder	2	$1.94	$3.88
Extruder	Digikey	terminal strip	1	$4.14	$4.14
Extruder	Amazon	140Pcs Assorted Spade Connector Kit	1	$13.99	$13.99
Extruder	Digitmakers	Duet 3 Mainboard 6HC	1	$348.95	$348.95
Extruder	Home Depot	1/8-inch × 24-inch × 48-inch Standard Hardboard Handy Panel	1	$8.58	$8.58
					$0.00
Feeder	Amazon	#6 1/2″ flat head wood screw − 100 pack	1	$23.99	$23.99
Feeder	Home Depot	#5x3/4 Flat Head Wood Screw − 28 pack	2	$3.47	$6.94
Feeder	Rona	2″ x 5/8″ Mending Platel -Zinc	14	$0.89	$12.46
Feeder	Home Depot	4′x8′x1/8′ Utility Panel	1	$19.98	$19.98
Feeder	Lowes	11/16-in × 3–1/2-in × 8-ft MDF Board	1	$10.99	$10.99
Feeder	Home Depot	PEX 3/4″x10′ waterline pipe	1	$12.24	$12.24
Feeder	Amazon	12″x12″x1/8″ Acrylic sheet	1	$28.49	$28.49
Feeder	Amazon	2″x2″x1.89″ L bracket − 6 pack	1	$22.90	$22.90
Feeder	Home Depot	3 in. × 20 ft. Vinyl Flexible Hose	1	$13.69	$13.69
Feeder	TUDS	4x4 square wood beam	1	$27.49	$27.49
Feeder	Home Depot	2″by4″by8′ SPF Dimensional Lumber	1	$5.98	$5.98
Feeder	Amazon	8 mm to 8 mm Shaft Coupler	1	$11.99	$11.99
Feeder	Rona	2″x5/8″ l-corner brace	4	$1.19	$4.76
Feeder	OMC-Stepperonline	Nema 17 Stepper Motor, 5:1 Gearbox	1	$28.85	$28.85
Feeder	McMaster-Carr	20mmx20mmx914mm aluminum extrusion	1	$10.84	$10.84

Use of each of the design files is provided through descriptions and figures shown in Section 5, “Build Instructions”.

## Build instructions

Tips:

1. It is recommended that the user familiarize themselves with the items in the BOM, design files and general hangprinter terms. The user is advised to view detailed part descriptions in in the Bill of Materials Section above.
2. Read through the tool list in Table 3 and ensure you have access to the necessary tools.

**Table 3:** Tools used.

No.	Tool	Where Used
1	Screwdriver with Phillips *P*2, 5 mm flathead driver and 2.5 mm hex driver	On all M8/M3/M4 slotted nylon screws, Phillips screws, socket hex head cap screws and wood screws
2	Metric Allen key set	For all M3/M4 socket hex head cap screws used almost everywhere
3	Adjustable wrench	For the various nuts and bolts
4	Circular saw	To cut wooden planks/dowels
5	Angle grinder	To cut steel rods
6	Vise	Help support parts while drilling holes, cutting and helping press fit bearings into their holes
7	Drill Press	For drilling holes into nozzle and nozzle adapter
8	Medium grit (80) sandpaper	For sanding Hangprinter spool components and any other 3-D printed parts
9	Superglue /JB Weld	For joining together, the spool assembly and feeding system components
10	Strong double side tape	For fixing Hangprinter anchors to ground
11	Ratchet and Wrench set	For connecting
12	Bristle brush	For cleaning nozzle of extruder
13	Hacksaw	For cutting wood
14	Spirit level	For Hangprinter calibration
15	Builders square long and short	For Hangprinter calibration
16	Measuring tape	For Hangprinter calibration
17	Marker	Various measurement tasks and Hangprinter calibration
18	Ruler	Various measurement tasks and Hangprinter calibration
19	Wire strippers	For all wiring
20	Wire cutters	For all wiring
21	JST wire crimpers and spade connector wire crimpers	For various connectors on all electrical components
3. 3-D print all parts shown in Table 4 with the appropriate material on a FFF based 3D printer with slicer settings shown in Table 5 and Table 6.

**Table 4:** 3-D printed components list including material.

**Component**	**Quantity**	**Material**	**Note**
ABC_motor_mount_spool	3	PETG	
bearing_u_608	12	PLA	
bearing_u_623	27	PLA	
belt_roller_insert_extended	4	PLA	
ChassisBeam	3	PLA	optional; if not making out of wood
corner_clamp	3	PLA	
D_motor_mount_spool	1	PETG	
dleft_spool	1	PLA	
dleft_spool_cover	1	PLA	
dright_spool_bottom	1	PLA	
dright_spool_cover	1	PLA	
dright_spool_top	1	PLA	
GT2_spool_gear_double	4	PLA	
horizontal_line_deflector	4	PLA	
line_roller_anchor_half_tilt	2	PLA	
line_roller_anchor_half_tilt_mirrored	2	PLA	
line_roller_anchor_straight	2	PLA	
line_roller_anchor_template	1	PLA	
line_roller_double	1	PLA	
line_verticalizer	3	PLA	
motor_holder	4	PLA	
sep_disc	1	PLA	
spool	3	PLA	
spool_cover	3	PLA	
spool_cover_mirrored	3	PLA	
tilted_line_deflector	1	PLA	
tilted_line_deflector_mirrored	1	PLA	
ziptie_tensioner_wedge	16	PLA	
6015 Fan Shroud	2	PLA	
Barrel Bracket Base	1	PLA	
Barrel Bracket Wing 1	1	PLA	
Barrel Bracket Wing 2	1	PLA	
Blower Fan Mount	2	PLA	
Blower Fan Plate	2	PLA	
IntakeTube	1	PETG	
IntakeTube_Extension	1	PLA	
IntakeTube_Hopper	1	PLA	
Motor Mount Square Bracket	1	PETG	
Feed Gear Enclosure	1	PLA	
Feeding Tube Coupler	1	PLA	
Feeder Motor Enclosure	1	PLA	
Feeder Outlet	1	PLA	
Feeding Gear	1	PLA	
Hopper Corner Bracket	8	PLA

**Table 5:** Slicer settings for PLA components.

Description	Setting
Layer height	0.15 mm
Infill density	80 %
Wall Thickness	0.8 mm
Support Material	No supports

**Table 6:** Slicer settings for PET-G components.

Description	Setting
Layer height	0.15 mm
Infill density	80 %
Wall Thickness	0.8 mm
Support Material	No supports
4. Ensure all parts have good layer adhesion as well as no distorted or missing layers. To assess the quality of the print and determine if a reprint is to be done, perform a visual inspection as summarized in [59] or use computer vision-based layer-wise 3-D printing analysis [60], [61]. As the Hangprinter components require sufficiently strong parts to hold up a relatively heavy end effector, any deficient parts should be reprinted.
5. Always wear safety glasses and protective gloves when using power tools such as angle grinders, circular saws, and jigsaws.

Most of the tools used are common to makerspaces, fablabs and workshops. Use of angle grinders and drill presses can be replaced by a proper work holding in a vise and using an impact driver and hacksaw. All the tools used are listed below in Table 3. Quantities and materials for the 3-D printed parts from the design files above are listed in Table 4 along with slicer settings in Table 5 and Table 6.

### Assembling all the Hangprinter components

This project uses components that are part of the original design of the Hangprinter v4. As such, in addition to [47], these components can also be found in Torbjørn’s Gitlab repository [62]. Certain components pertaining to the original Hangprinter motor and spool assemblies have been modified using TinkerCAD. These parts have been modified to accommodate for the larger size of the Nema23 stepper motors and a heavier end effector. For these modified parts both the STL and STEP files have been provided but not the.SCAD files. Regardless, STL, STEP and.SCAD files of all other Hangprinter components have been provided for the user to modify as needed in their preferred CAD program.

**Fig. 7:**
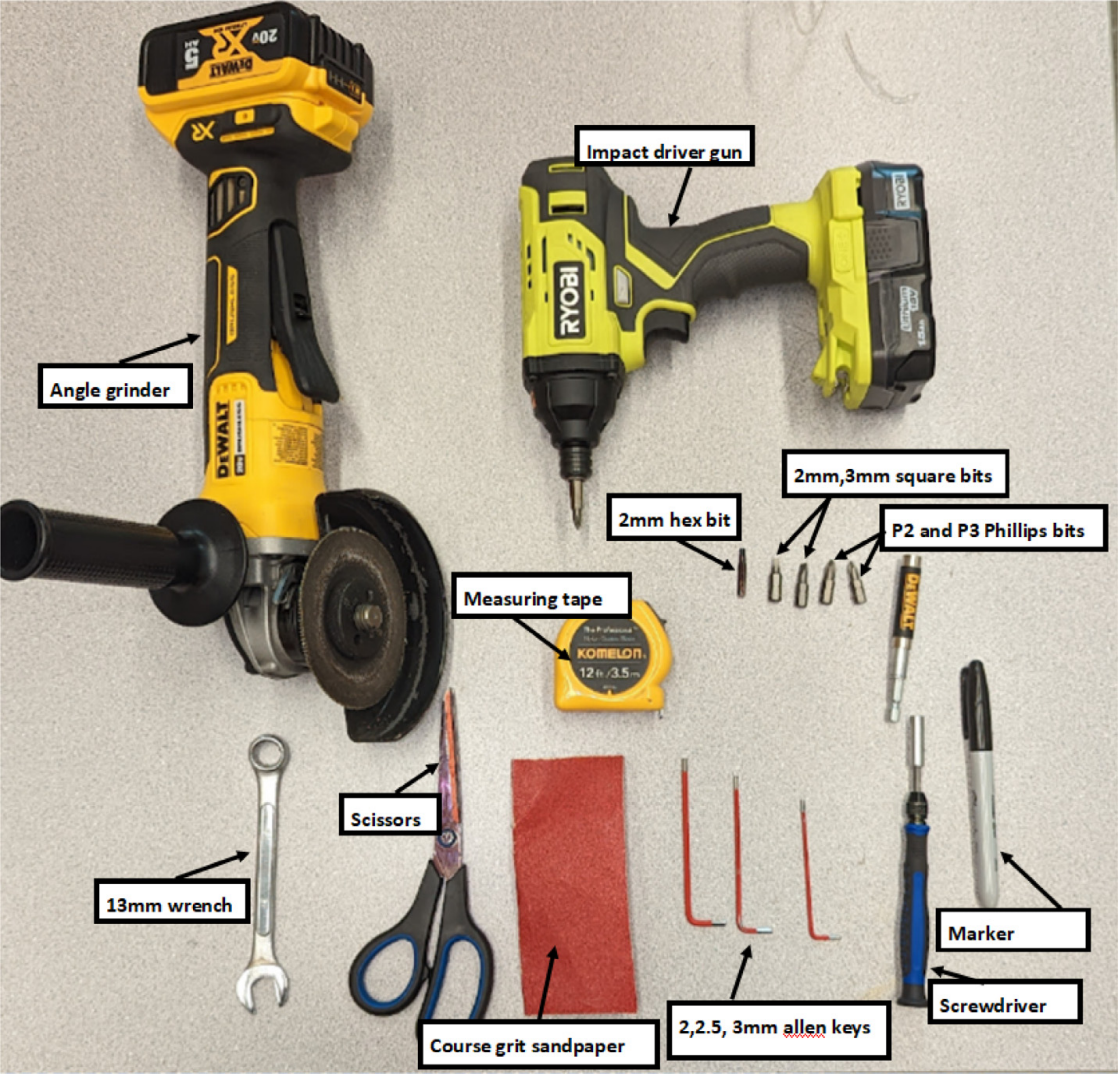
Tools needed for hybrid Hangprinter assembly.

All Hangprinter components are either off the shelf or are 3-D printed. Ensure that all the components in Table 1 are printed in either PLA or PETG or superior 3-D printed plastic material. PETG is recommended for its high tensile and flexural strength, its durability making it well suited for parts bearing loads over time, as well as its easiness of use and availability on the market. Make note of the tools used in each of the sections below shown in Fig. 7.

#### Determining line lengths, anchor placement, print area and heights

Due to the scalable nature of the Hangprinter system, it is up to the discretion of the user to determine the quantity of string to buy as well as any other components that are dependent on line lengths (such as electrical wiring from the ceiling). Quantities for the specific machine built for this paper are provided as well as methods for determining a custom system are shown below.

**Fig. 8:**
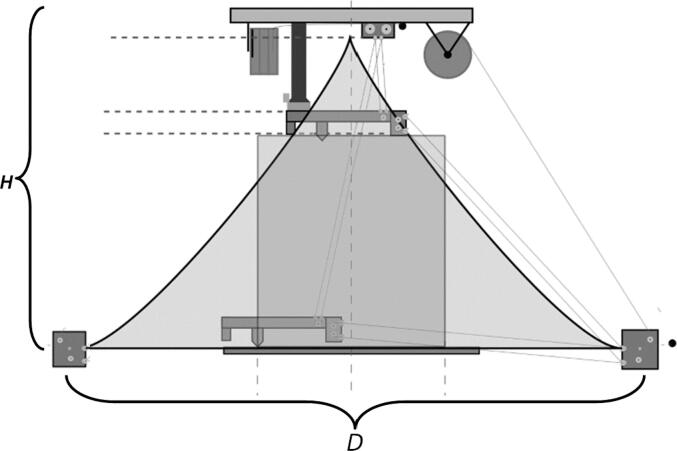
Build and reachable volume of Hangprinter (Only one ground anchor shown). .

Adapted from [63]
• Furthest anchor is the D anchor on the ceiling mount [49].

Assumptions:

• All heights are colinear with the Z-axis.
• A, B, C anchors are all placed equally the same distance from the triangle (+/-5mm).
• Anchor norms are followed as per hangprinter v4 conventions (i.e., A anchor is in -Y direction, B anchor is in + XY direction, C anchor is in −X + Y direction).
• Due to a cylindrical build volume with a hyperbolic movable area of the hangprinter (Fig. 8).
  o Minimum ABC line length determined when end effector is at the highest reachable Z coordinate [63].
  o Minimum D line length determined when end effector is at Z = 0 (i.e., nozzle touching print bed) and parked near one of the A, B, or C anchors [63].

**Fig. 9:**
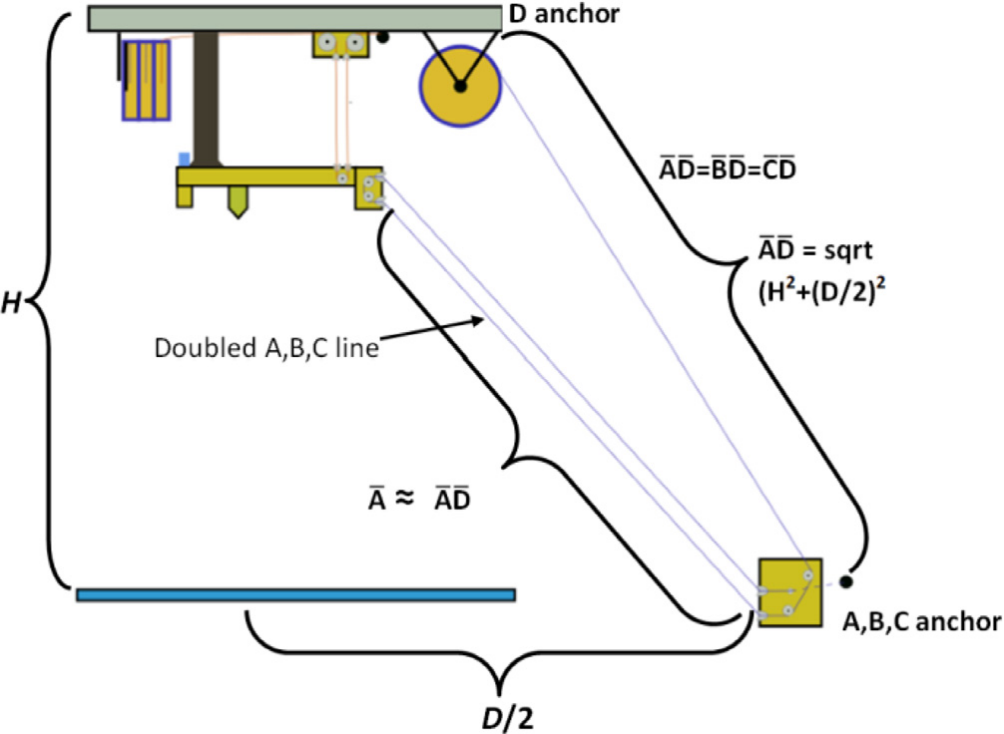
ABC anchor line lengths in effector's extreme position. .

Adapted from [63]

**Fig. 10:**
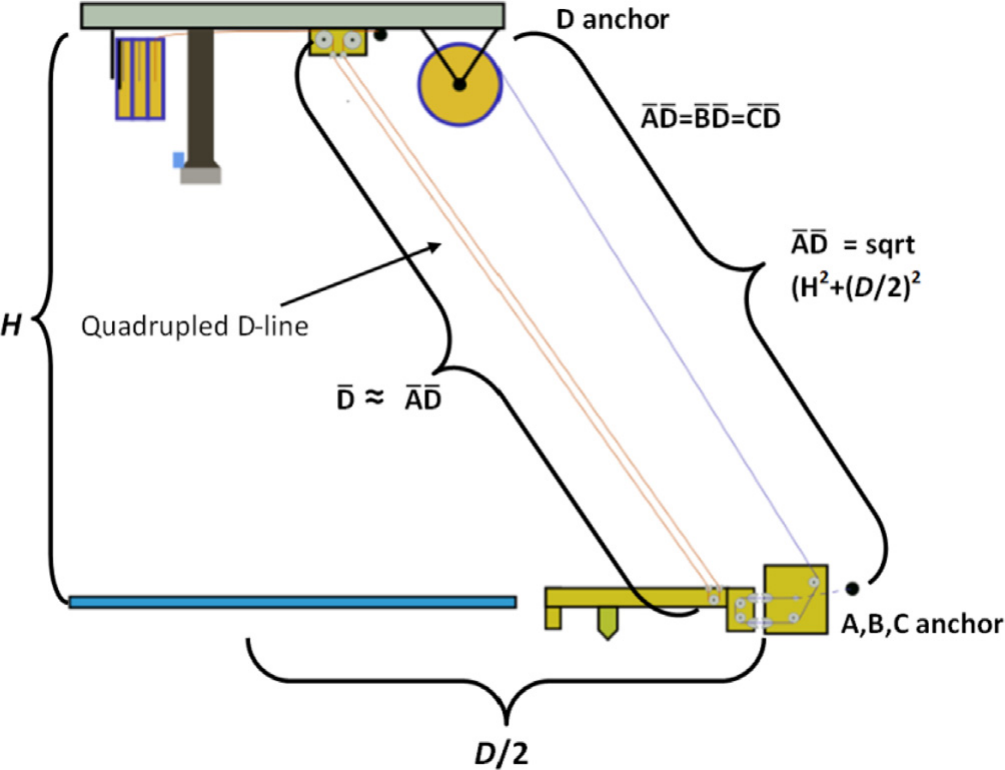
D line length in end effectors extreme position. .

Adapted from [63]

The A,B,C and D line lengths can be calculated using when the end effector is in the position shown in Fig. 9 and Fig. 10 where:

• *H* is the desired ceiling mount height (defined by user).
• *D* is the outer diameter of the circle which the A, B, C ground anchors lie on (defined by user).
• *spoolbuffer* is spare line in case of possible print area expansion and for safety (set to 2000 mm).
• *extra* is approximated to be the amount of line routed on the ceiling mount (set to 500 mm).

(4) $$l_{\mathrm{ABC}}=3*\sqrt{H^{2}+{\left(\frac{D}{2}\right)}^{2}}+spoolbuffer+extra$$

(5) $$l_{D}=4*\sqrt{H^{2}+{\left(\frac{D}{2}\right)}^{2}}+spoolbuffer+extra$$

Thus, the total amount of spectra line to be cut is:

(6) $$l_{\mathrm{TOTAL}}=6l_{\mathrm{ABC}}+3l_{D}$$

This translates to the following number of windings around the spool:

(7) $$W_{\mathrm{ABC}}=\frac{l_{\mathrm{ABC}}}{\pi d_{\mathrm{spool}}}$$

(8) $$W_{D}=\frac{l_{D}}{\pi d_{\mathrm{spool}}}$$

In the equations above, the line lengths for the A, B, C lines when the end effector is in its extreme position are approximated to be the hypotenuse formed by H and D/2 (multiply by 3 due to doubled lines and distance between D and A, B, C anchors). This provides a conservative estimate. A similar method for determining the D line was used however is multiplied by 4 due to quadrupled lines on the end effector. To learn more detail on the nature of the above formulations, please see [63], [49]. Quantities for this project’s set up are shown below in Table 7.

**Table 7:** Hangprinter parameters used.

**Parameter**	**Units**	**Note:**
*l_D_*	16.7 m * 3 lines = 50.1 m	Total length of spectra line from D spool to end effector with quadrupled lines to and from end effector
*l_ABC_*	11.07 m/spool * 3spools * 2 lines = 66.42 m	Total length of spectra line from A, B, C spools to anchors to end effector. Doubled lines between end effector and ground anchor.
*W_ABC_*	23.5 m	No. of windings of spectra line around A, B, C spools
*W_D_*	35.59 m	No. of windings of spectra line around D spool
*d_spool_*	0.48 m	Diameter of “spool.stl”
*H*	2500 mm	Distance from anchor to chassis
*D*	1.6 m	Diameter on which ground anchors A, B,C rest on. User defined.

Parts required:

• (1) Dorisea Spectra line 300 mm spool

Steps:

1. Measure the area of the room and decide on a suitable mounting height, *H* and a max outer diameter *D* (circle which anchors are on). Alternatively, use the *H and D* parameters from Table 7.

2. Cut the D line according to equation (5) or Table 7.

3. Cut the ABC lines according to equation (4) or Table 7.

4. Label these cut lines as they will be spooled in the subsequent sub-sections.

5. Anchors shall be placed on the max outer diameter *D* parameter. Do not affix these to the ground yet as the proper placement of these anchors will be in relation to the physical hangprinter triangle chassis position.

#### Building the ceiling mount and anchor line routing components

The line “rollers”, “verticalizers” and “deflectors” are the components on the ceiling mount and anchor points that route the A, B, C and D lines from the spools to the hangprinter chassis triangle.

Parts Required:

• (13) M8 Bolts and (13)M8 nuts
• (4) M3 Hex nylon screws(16 mm) and (20) M3 nuts
• (3) M3 Hex nylon screws (25 mm)
• (12) M3, 50 mm long screw from M3 Hex Socket Cap Screw set
• (13) 608 (608ZZ) Bearings
• (10) 623 (623ZZ) Bearings
• (12) plastic eyelets
• (13) “bearing_u_608”
• (10) “bearing_u_623”
• (2) “line_roller_anchor_half_tilt.stl”
• (2) “line_roller_anchor_half_tilt_mirrored.stl”
• (2) “line_roller_anchor_straight.stl”
• “tilted_line_deflector.stl”
• “tilted_line_deflector_mirrored.stl”
• “line_roller_double.stl”
• “line_roller_wire_rewinder.stl”
• “line_verticalizer.stl”
• “horizontal_line_deflector.stl”
1. Print the above components and follow the part inspection guidelines set in [59] or [60], [61].
2. Cap all 13 of the 608 bearings with each of the printed “bearing_u_608” part as shown in Fig. 11. To help press fit the bearing into the cap, use a vice or lightly tap the bearing with a rubber mallet.

**Fig. 11:**
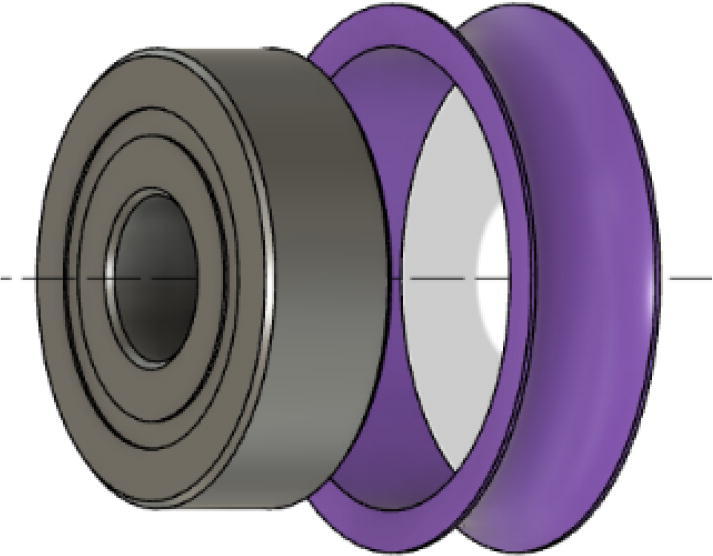
Capping 608 bearing with printed “bearing_u-608” part.
3. Fix 2 capped 608 bearings into each slit of the printed “tilted_line_deflector” part and secure them in place with the M8 bolt seen in Fig. 12*.* Secure the assembly with a M8 nut on the other end of the screw. Use a flat head screwdriver and adjustable wrench to tighten assembly.

**Fig. 12:**
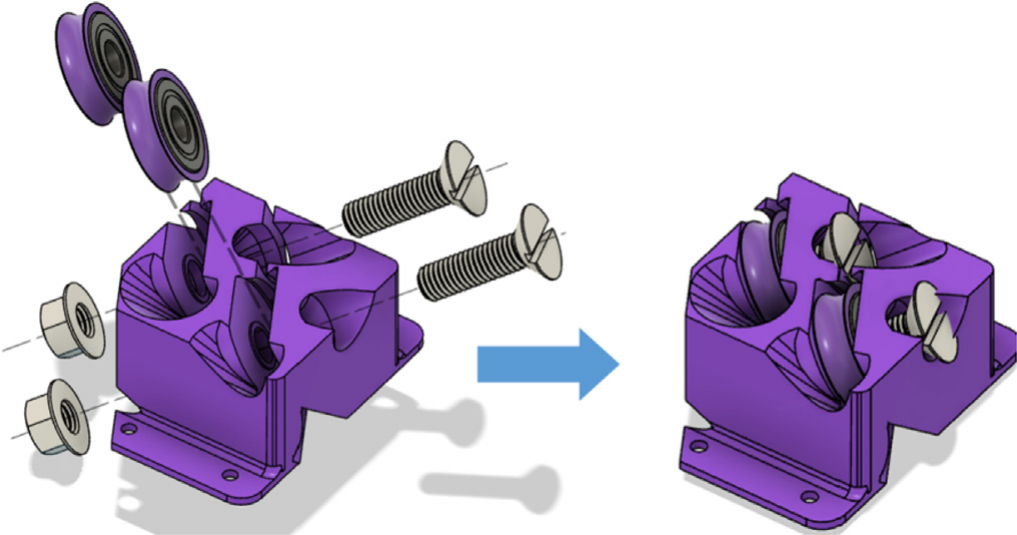
Assembly of ceiling mount tilted line deflector for B and C lines.
4. Repeat step 2 for the “tilted_line_deflector_mirrored” part. This completes the “tilted_line_deflector” and “tiled_line_deflector_mirrored” component assemblies for the B and C line routing on the ceiling mount.
5. Repeat step 2 for the printed “line_roller_double” part shown in Fig. 13. This completes the “line_roller_double” component assembly for the A line on the ceiling mount.

**Fig. 13:**
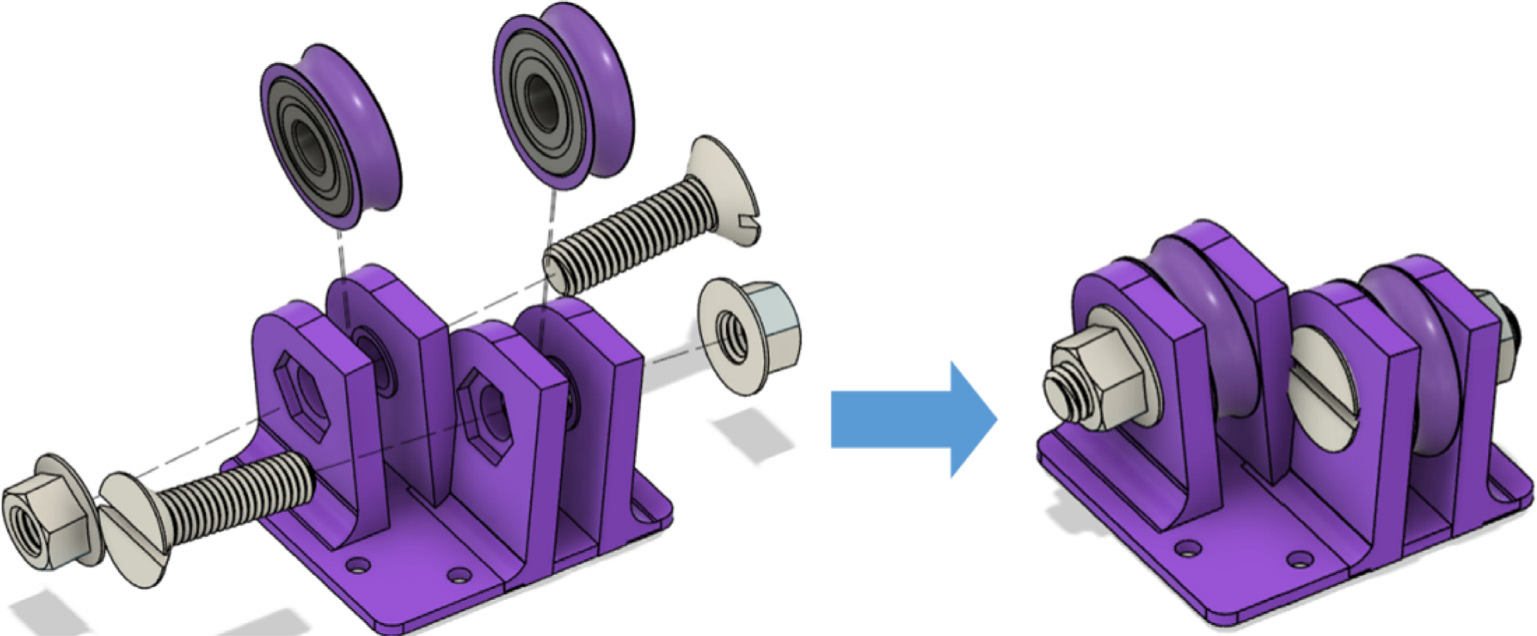
Assembly of ceiling mount doubled line rollers.
6. This completes the assemblies of the ceiling mount line routing components. Now assemble the line roller components on the anchors.

**Fig. 14:**
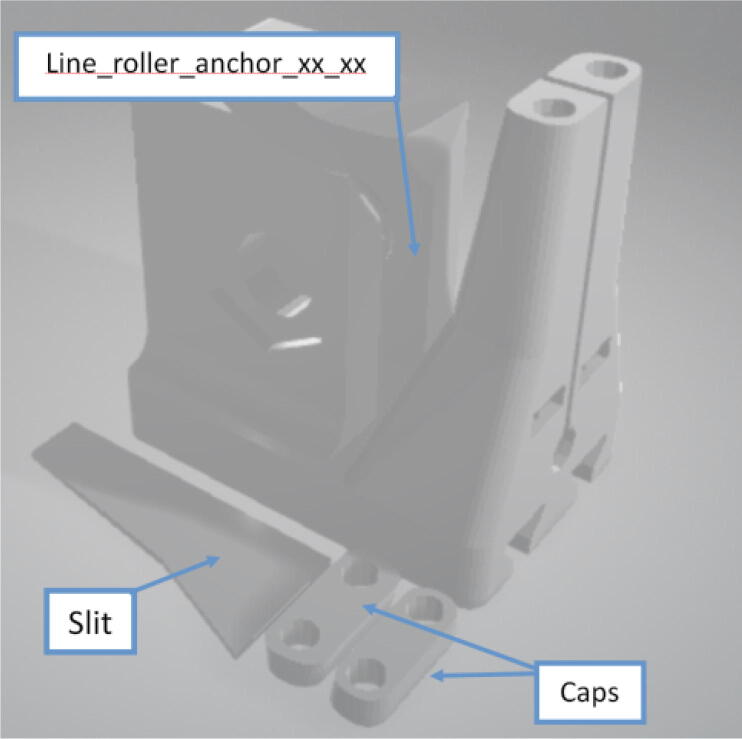
STL File constituents for all “line_roller_anchor_xx_xx” parts.

Note that all the above mentioned “line_roller_anchor_XX_XX” STL files include 3 smaller components (shown in Fig. 14) constitute the subassembly of the printed component. While they are assembled in this step, they are not used until the Section 5.1.3 “Building Ground Anchors” section below. These components experience high bending moments from the tension caused by the lines. As per Table 4, these components should be printed with PETG (or better mechanical polymer) for high durability.

7. For all “line_roller_anchor_xx_xx” parts, insert a capped 608 bearing into the large slit and secure it with the M8 bolt and as shown in Fig. 15*.* Tighten with a flathead screwdriver and adjustable wrench.

**Fig. 15:**
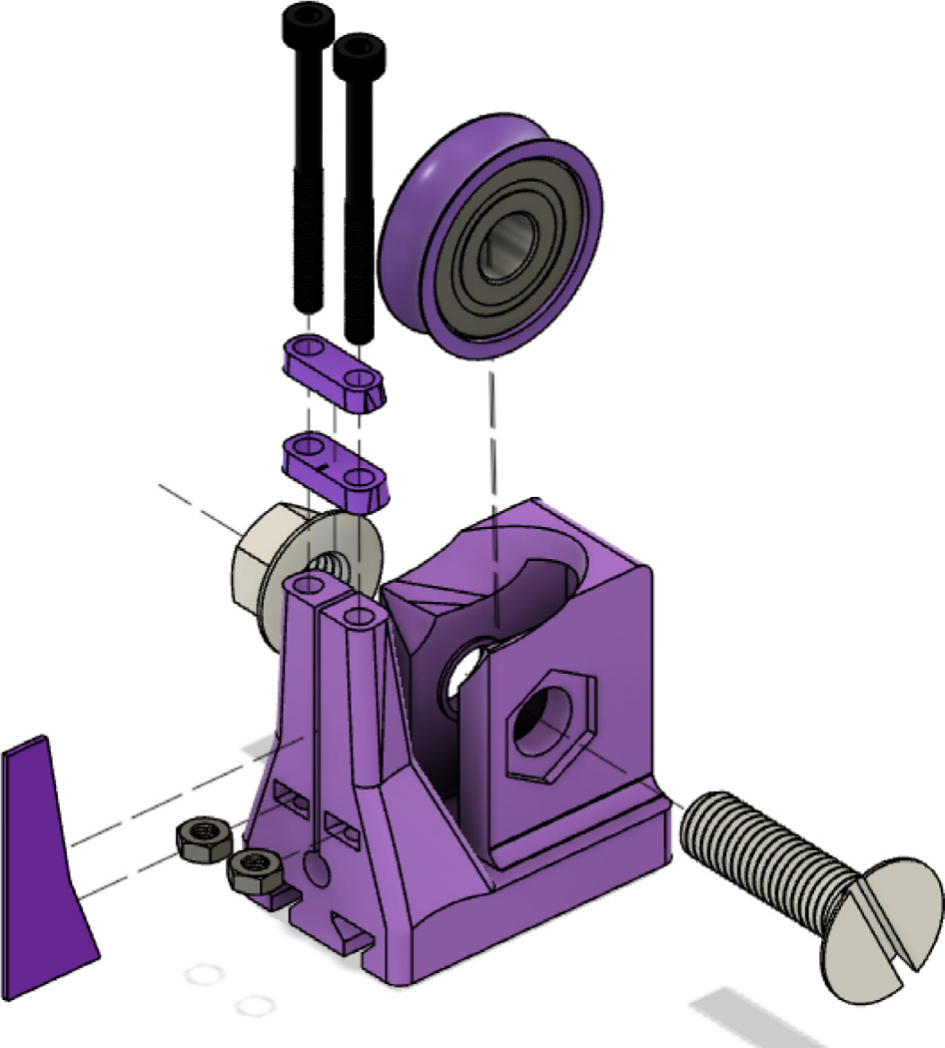
Assembling the “line_roller_anchor_xx_xx” style components.
8. Next, screw in the 2 caps using the 50 mm long M3 cap screws with a 2.5 hex driver as shown in Fig. 15*.* Insert two M3 nuts (one into each slit) near the bottom of the line roller anchor as shown in Fig. 15. Do this for all six-line roller anchor parts. For brevity, only one variation of the line roller anchor is shown here, but the assembly is identical for the other variations such as straight- and tilted-line roller anchors.
9. Like the 608 bearings, cap 623 bearings with a printed ‘bearing_u_623′ as shown in Fig. 16*.*

**Fig. 16:**
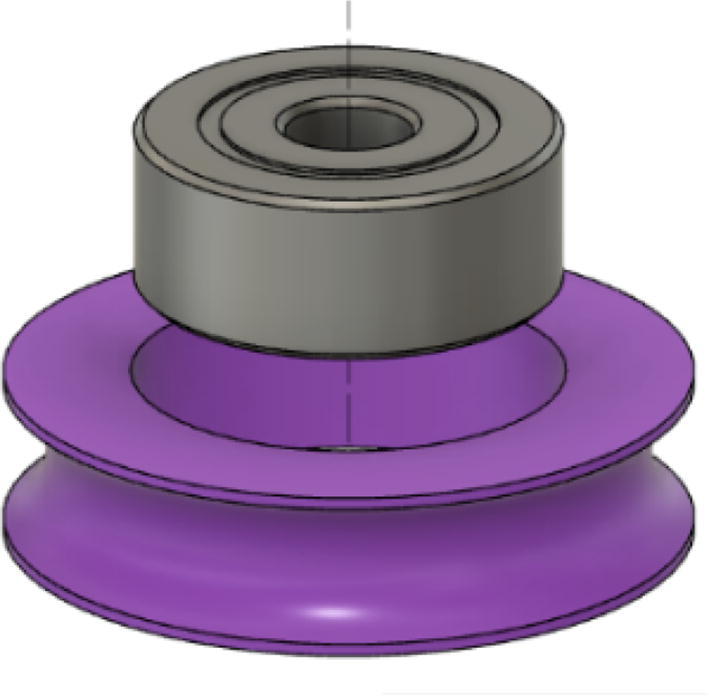
Capping 623 bearing with printed “bearing_u_623″ part.
10. Insert these capped 623 bearings into the respective slits of all “line_verticalizer” and “horizontal_line_deflector” and secure the bearings with the M3x16mm nylon hex screws and a M3x25mm nylon hex screw respectively. Tighten with corresponding M3 nuts as shown in Fig. 17 and with a flathead screwdriver.

**Fig. 17:**
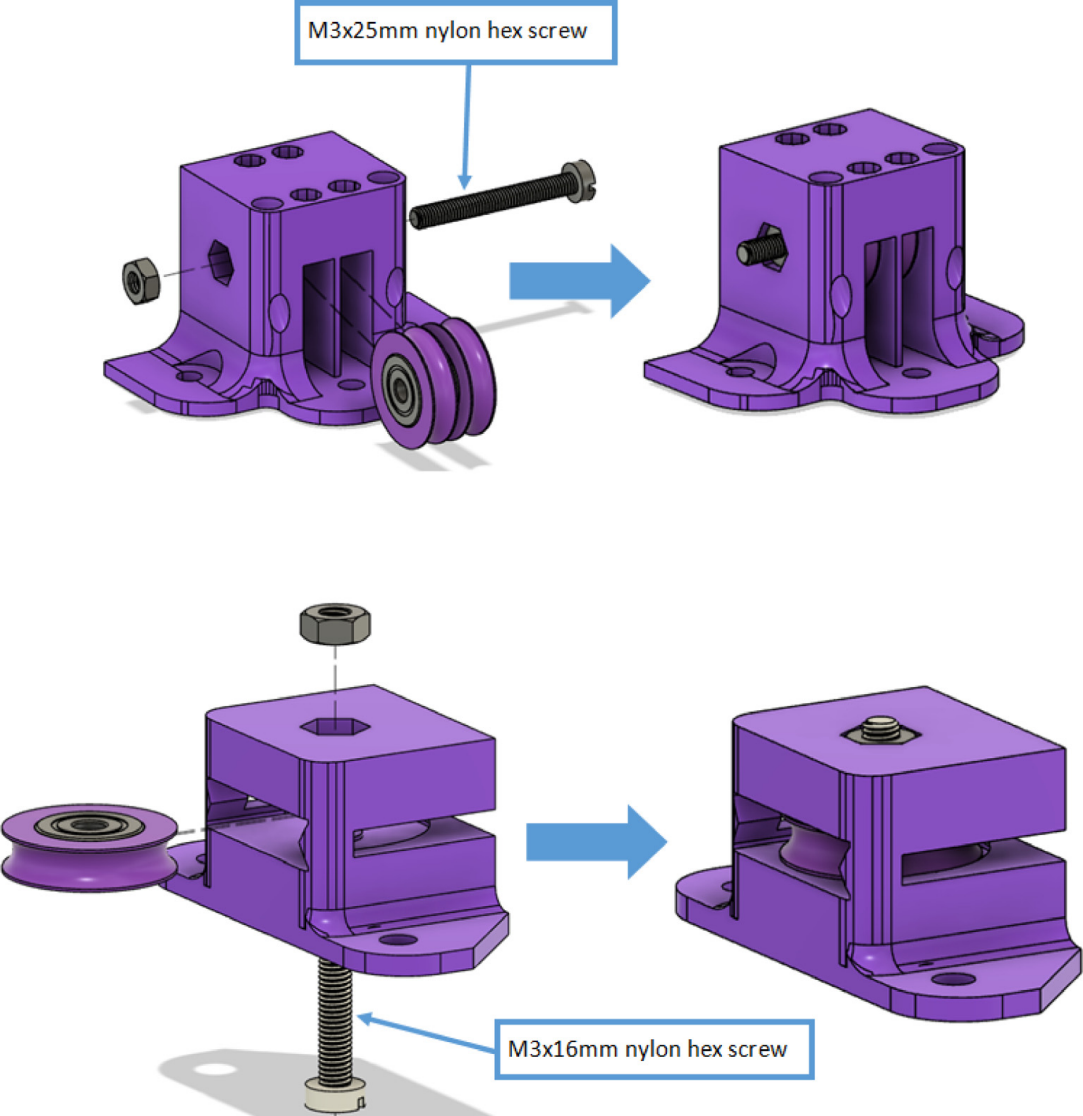
Assembly of Line Verticalizer and Horizontal Line Deflector.

This completes the component assemblies of all the line routing components that will be mounted on the ceiling and anchors. At this point, you should have completed assemblies of:

• (2) “line_roller_anchor_half_tilt”
• (2) “line_roller_anchor_half_tilt_mirrored”
• (2) “line_roller_anchor_straight”
  o “tilted_line_deflector”
• “tilted_line_deflector_mirrored”
• “line_roller_double”
• “line_roller_wire_rewinder”
• “line_verticalizer”
• “horizontal_line_deflector”

Save these parts and move on to the assembly of the spools and motor bracket assemblies below.

#### Building the ground anchors

With the anchor line rollers (informally called snails due to their shape) assembled, they are now to be fastened onto a wooden beam which will then be later attached to the ground via weights and a strong double-sided tape. These line roller anchors are fastened to these wooden beams via 4 wood screws which the base of the line anchor rollers slide into with their built-in channels.

Parts needed:

• (6) assembled line_roller_anchor parts along with their included caps and slits from Fig. 14
  o (2) “line_roller_anchor_half_tilt.stl”
  o (2) “line_roller_anchor_half_tilt_mirrored.stl”
  o (2) “line_roller_anchor_straight.stl”
• (1) “line_roller_anchor_template.stl”
• (3) 2″ by 4″ by 8′ SPF dimensional lumber plank
• (24) #5x3/4 flat head wood screws
1. To produce the wooden beams that the line roller anchors will mount on, measure and mark 3 lines, each spaced out 25.16″ along the length of the 2″x4″ lumber plank. Make cuts along these lines using a circular saw or any other appropriate saw.
2. Print all of the “line_roller_anchor_xx_xx” parts and inspect for any deformities or errors according to the inspection method outlined in [59] or [60], [61].
3. Print the “line_roller_anchor_template” part. This is used to help with the alignment and spacing of the mounting holes for the line roller anchor parts.
4. On each of the cut 2″x4″ wood planks (laid out horizontally), position the “line_roller_anchor_template” part on the wood plank according to Fig. 18*.* The units shown are in millimeters (mm).

**Fig. 18:**
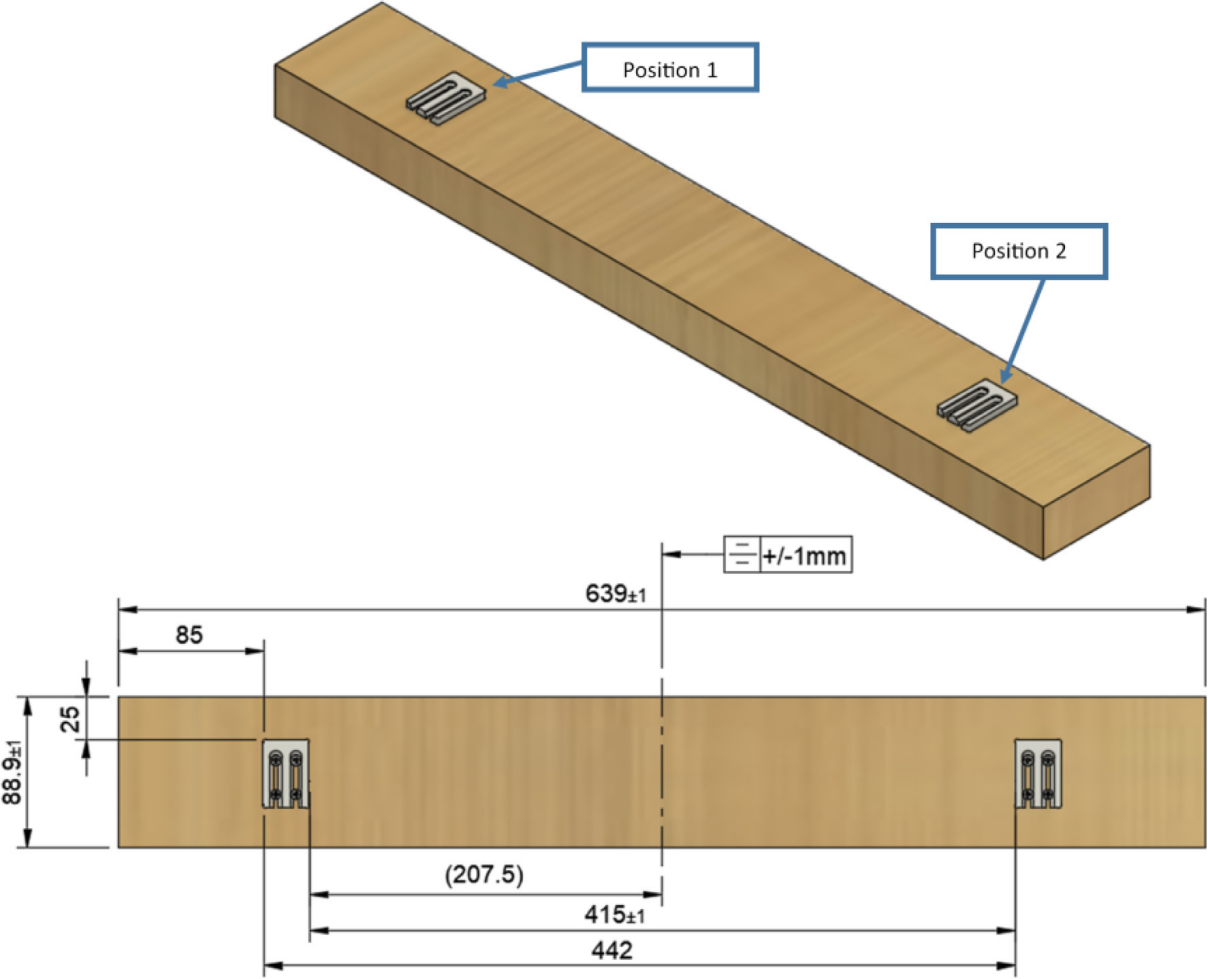
Positioning the “line_roller_anchor_template” on wooden plank.
5. Using a marker, mark 2 dots spaced out approximately 1.2 cm – 2 cm vertically inside each slot of the “line_roller_anchor_template”. Do this for both positions shown in Fig. 19*.* Ensure that the slots of the template piece are vertical and that the top-hole markings should be made so that the head of the screw is coincident with the top arch of the slot.

**Fig. 19:**
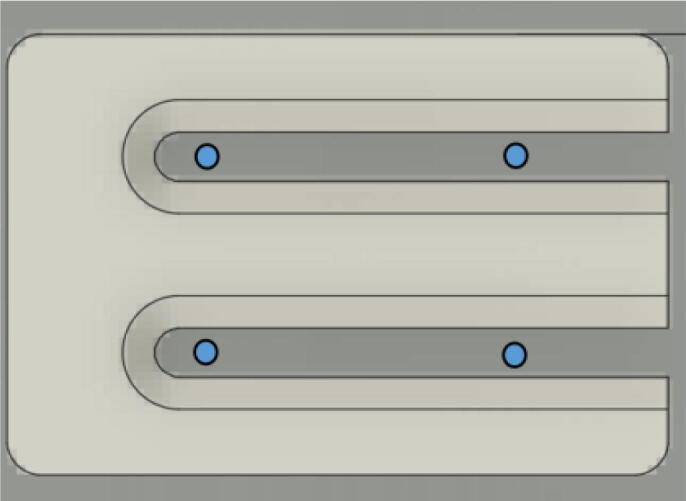
Guiding screw hole markings.
6. Using a screwdriver or drill, screw in the wood screws in the marked locations from the previous step. Ensure that the head is approximately 0.5 cm above the top face of the wood plank as shown in Fig. 20. This is so that the line roller anchor’s channels can slide under the screws and be secured into place.

**Fig. 20:**
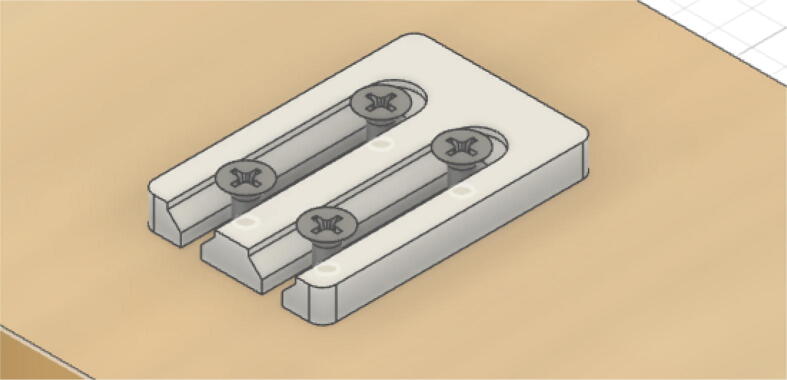
Using the line roller anchor template to insert wood screws.

**Fig. 21:**
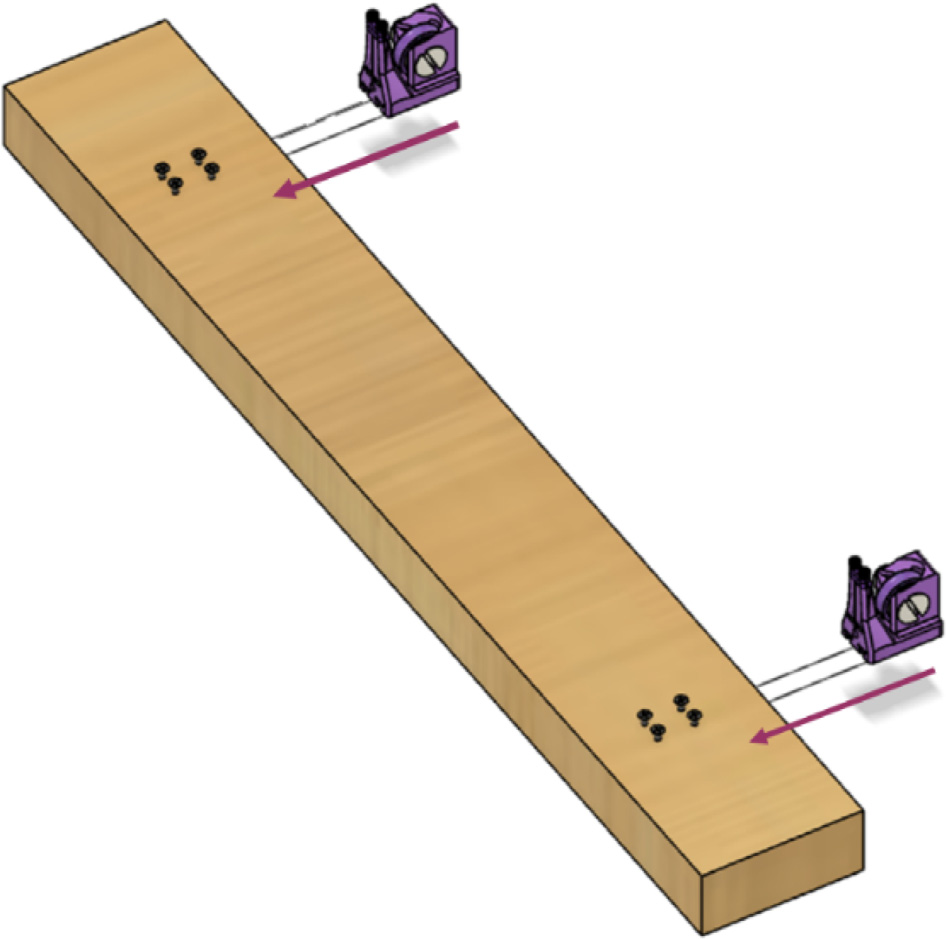
Attaching the line roller anchors to the wood beam to create complete anchor.
10. Repeat step 8 for anchor B but use the “line_roller_anchor_tilted_mirrored” assembly.
11. Repeat step 8 for anchor C but use the “line_roller_anchor_tilted” assembly.

*Note: Depending on the nature of the assembly and angle of the lines coming from the ceiling to the floor anchors, a different kind of line roller may be needed (*e.g.*, straight in place of tilted line roller anchors for the B and C anchors). Print more of these as needed.*

7. Once the holes on all 3 wood beams are made, the “line_roller_anchor_template” part is not needed and can be discarded or stored.
8. Somewhere on the wood plank, label each with the letters “A”, “B”, and “C” respectively. These represent the A B and C line anchors.
9. For anchor A: Line up the channels of the “line_roller_anchor_straight” assembly with the screws and wood plank. Then slide the anchor into place as shown in Fig. 21.

Do not fix these anchors to the ground yet. Placement of these anchors relative to the ground are shown in the respective “Operating Instructions” section.

#### Assembling the hangprinter chassis

The hangprinter chassis consists of simple wooden beams cut from MDF board and 3-D printed corner clamps and bearings fastened together by screws and nuts. Six 623 capped bearings are inserted on each side of the corner clamp to route lines from the triangle to the ground anchors. As a design alternative, the user can also choose to 3-D print these beams using the provided.STL file from PLA, PETG or equivalent material.

Parts required:

• (3) “corner_clamp.stl”
• (18) “bearing_u_623.stl”
• (30) “ziptie_tensioner_wedge.stl”
• (18) 623 (623ZZ) Bearings
• (21) M3 nuts
• (6) M3x16mm Slotted Hex nylon screws,
• (6) M3x 50 mm screw from M3 Hex Socket Cap Screw Kit
• (3) M3x25mm Slotted Hex nylon screws
• (24) eyelets (2) 1″x36″ Square Poplar Wood Dowel
1. Print 3 “corner_clamp.stl” components and insert the eyelets into the locations shown below in Fig. 22. There should be 8 eyelets per “corner_clamp” piece. These are used to ensure the line routing is smooth and the string does not rough against the rough edges of the printed component.

**Fig. 22:**
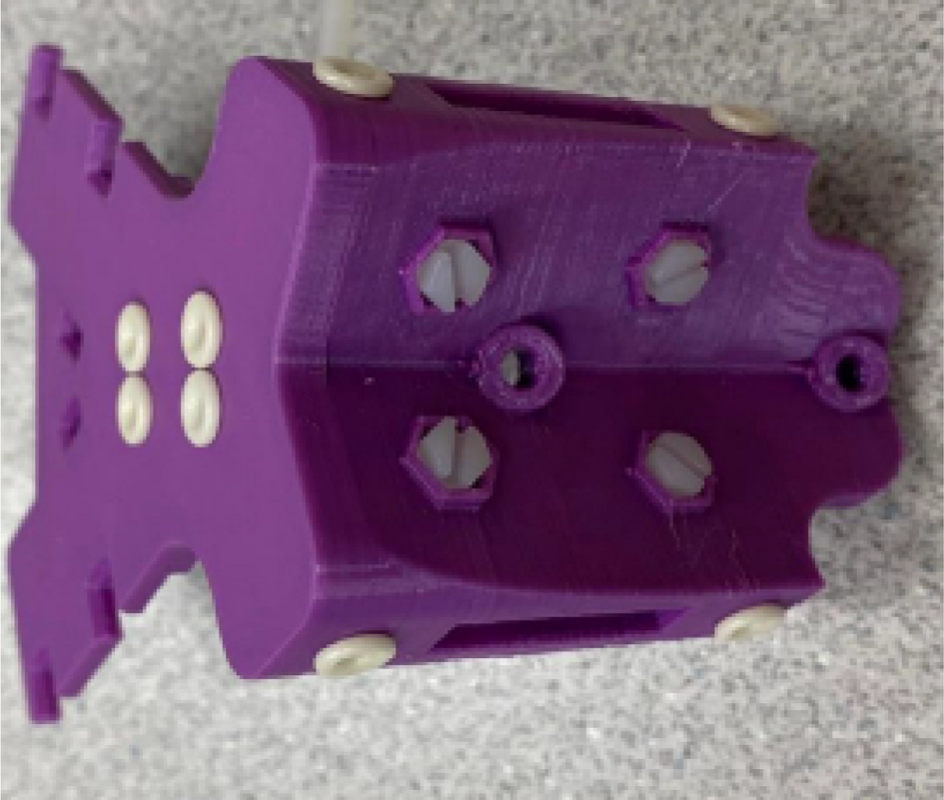
Printed “corner_clamp” part with eyelets inserted.
2. Insert 6 capped 623 bearings into the slots of each “corner_clamp.stl”. Then insert the M3 screws to secure the bearings in place as shown in Fig. 23*.*
  1%2 For the top left and rght corners, the longer M3x50mm hex socket screws should be used as these will be the fasteners for joining the wood beams that make up the triangular hangprinter chassis. Secure two M3 nuts on each of the hex socket screws. The first nut goes all the way towards the head of the screw. Leave the other M3 nut anywhere along the screw. This will be used to secure the wood beam that will be fixed on the other end of the screw.
  2%2 Use the M3x16mm slotted hex screws for the bottom left and right holes. Secure with two M3 nuts.
  3%2 Use the M3x25mm slotted hex screw for the hole on the middle slit as shown in Fig. 23*.* Secure with a M3 nut.
  4%2 Tighten the slotted and hex head screws with a flathead and hex driver bit screwdriver respectively. Be sure to not screw the long M3x50mm hex cap screws in all the way as they need to be drilled into the chassis wood beams in the steps below.

**Fig. 23:**
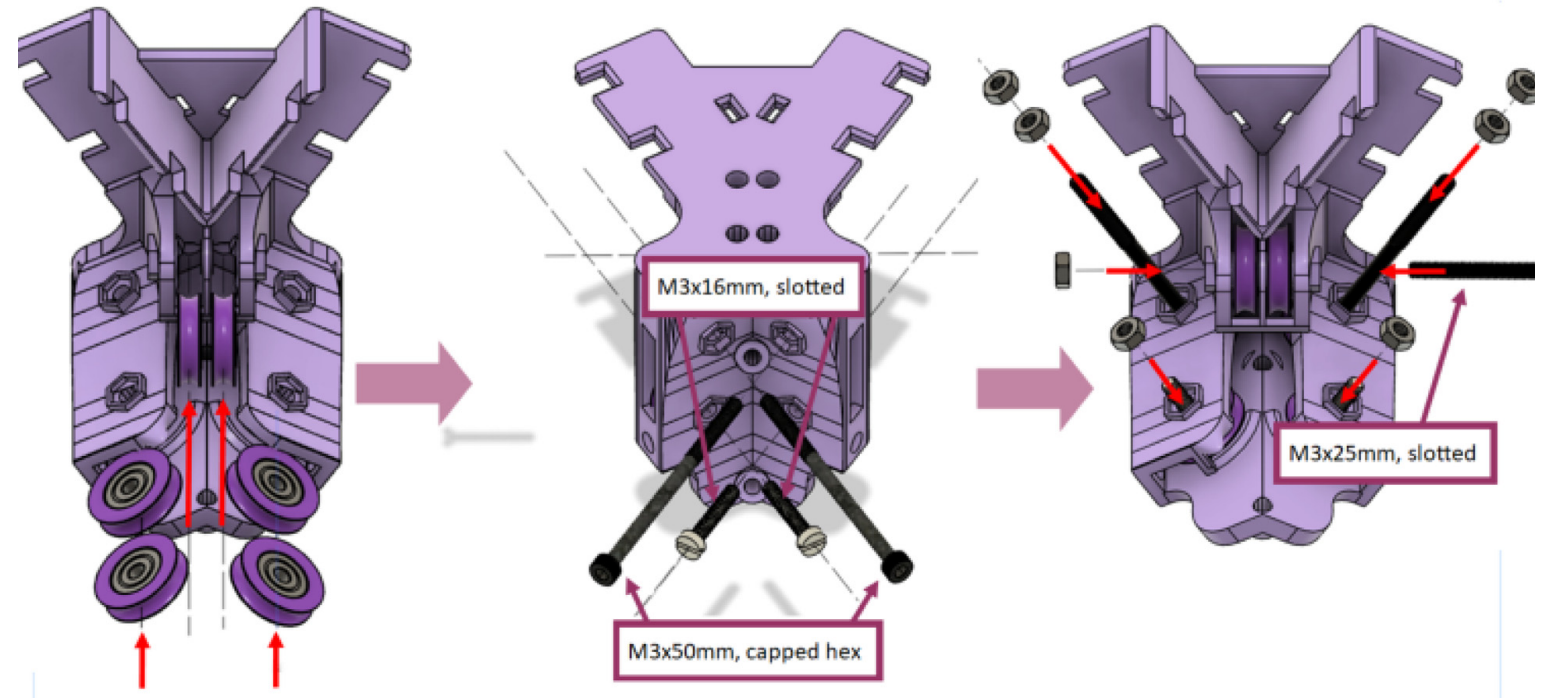
“corner_clamp” component assembly.
3. Repeat step 2 for all 3 “corner_clamp” components.
4. Using a circular saw, cut three 1″x1″x15.748″ (25.4 mm × 25.4 mm × 400 mm) wood beams from the 1″x36″ square dowel as shown in Fig. 24 and Fig. 25. Using a 1/8″ (3 mm) drill bit, drill a hole on both ends of the wood beam about 4 cm deep as shown in Fig. 24*.* The full drawing can be found in the “Electrical Schematics and Drawings” folder in [47].

**Fig. 24:**
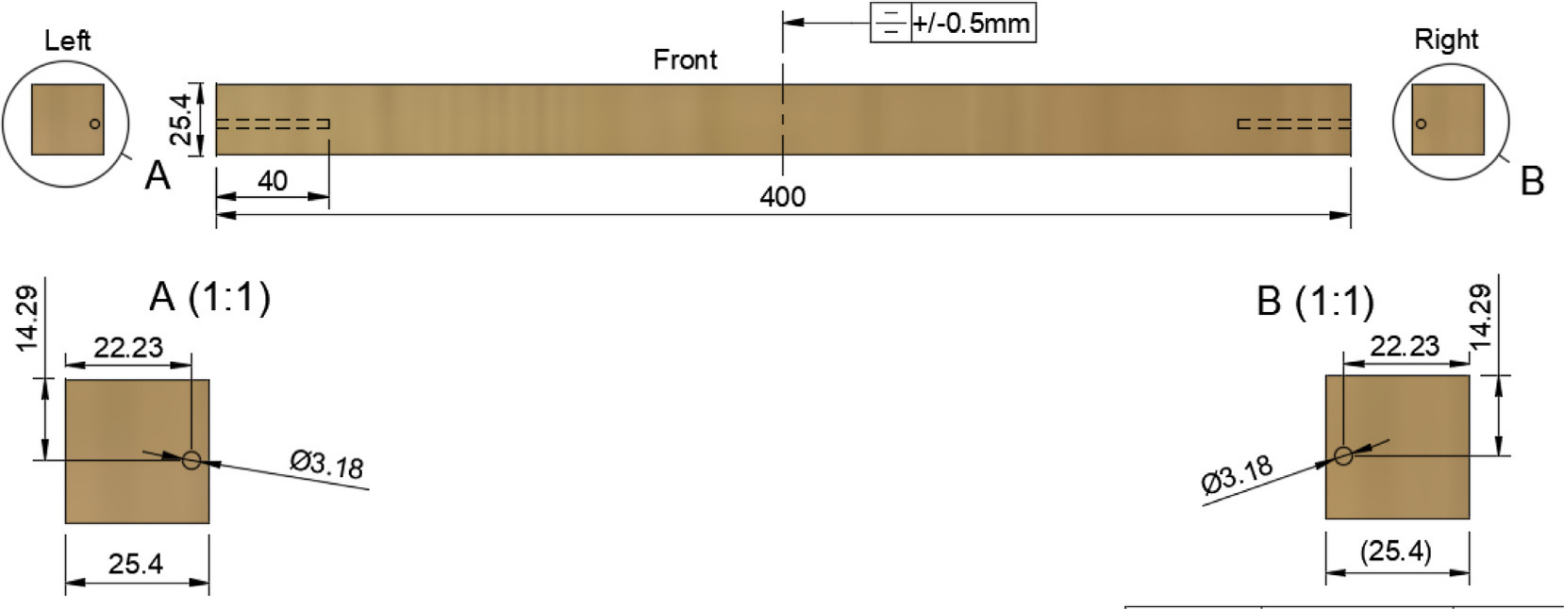
Dimension of cut wood beam (mm).

**Fig. 25:**
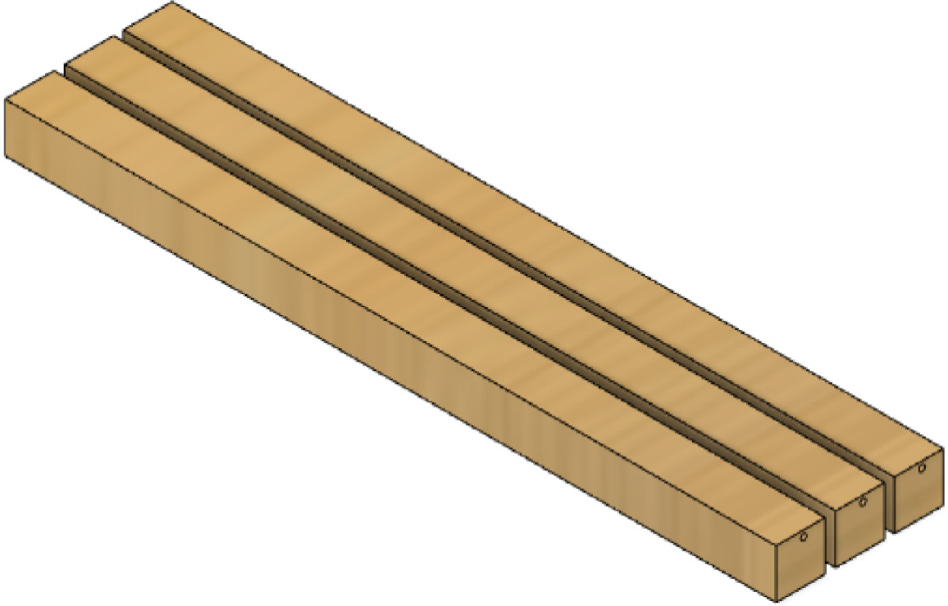
cut wood beams for the Hangprinter chassis.
5. Attach the wood beams to the “corner_clamp” assembly by sliding the wood beam along the “corner_clamp’s” channels as shown in Fig. 26. Once in place, secure the wood beam by screwing in the 50 mm long, M3 hex socket screws into the corresponding holes in the wood beam. Adjust the second M3 spacer nut on the socket screw so that it is fastened against the end face of the wood beam as shown in Fig. 27*.*

**Fig. 26:**
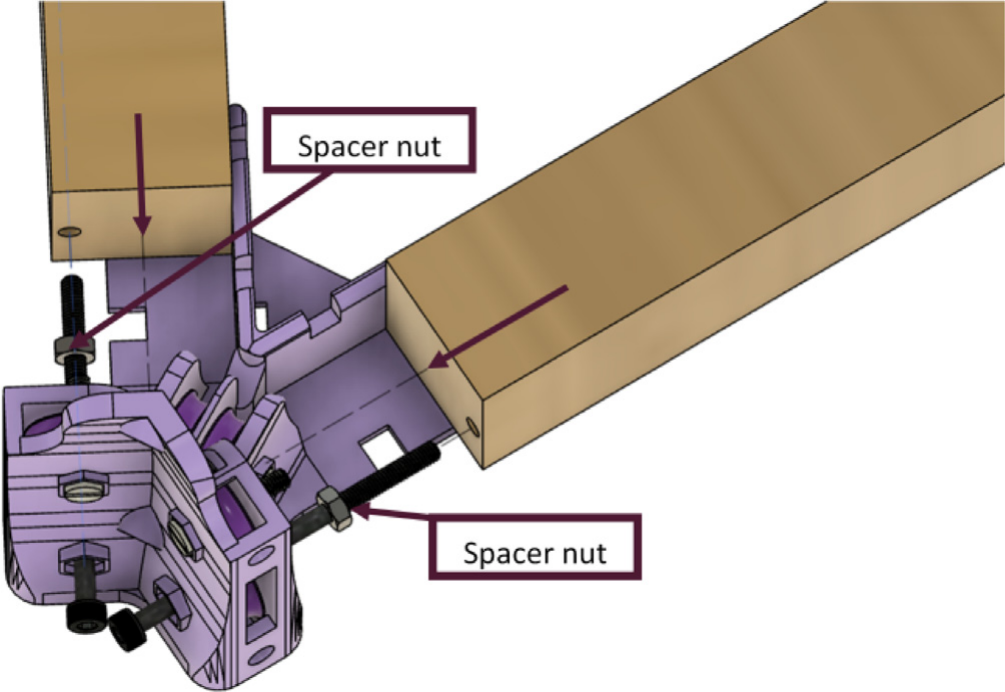
Corner clamp assembly to wood beam.

**Fig. 27:**
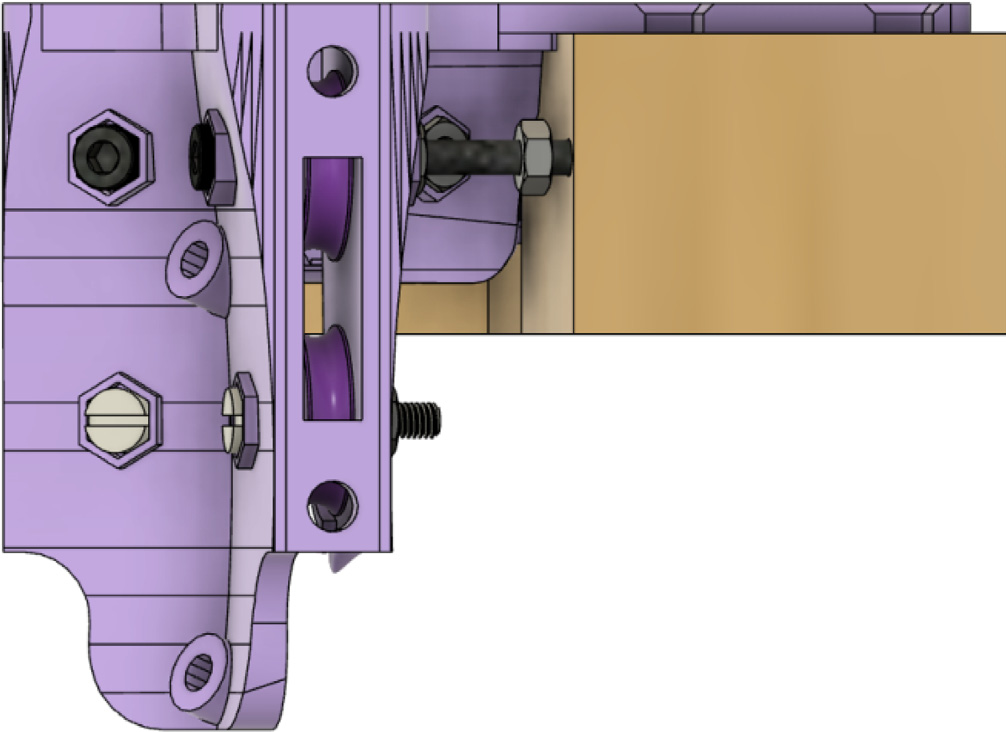
Assembled corner clamp and wood beam.
6. Repeat step 4 for all three of the “corner_clamp.stl” parts resulting in a triangular frame shown below in Fig. 28*.*

**Fig. 28:**
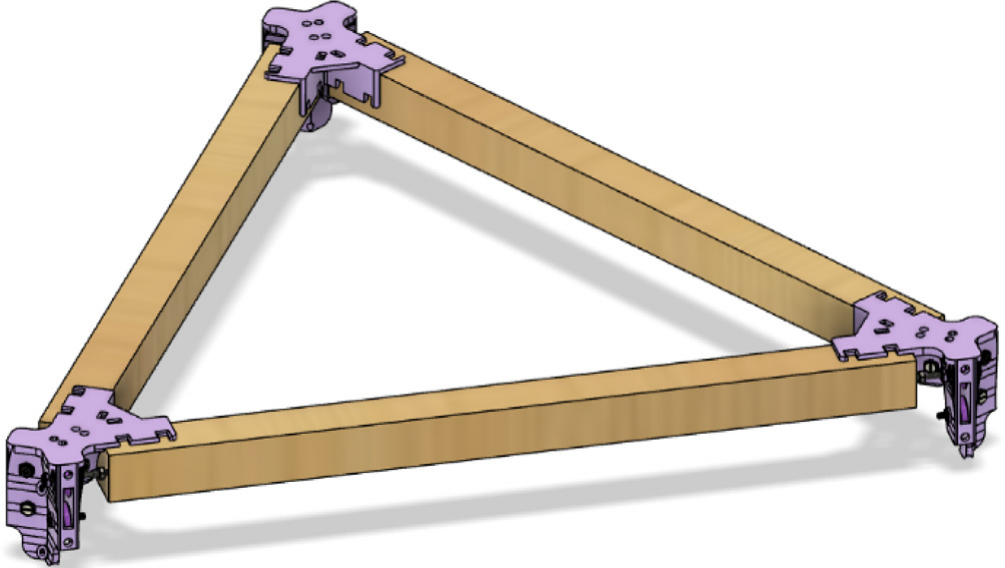
Fully assembled hangprinter chassis frame.

#### Assembling motor mounts and spools

**Fig. 29:**
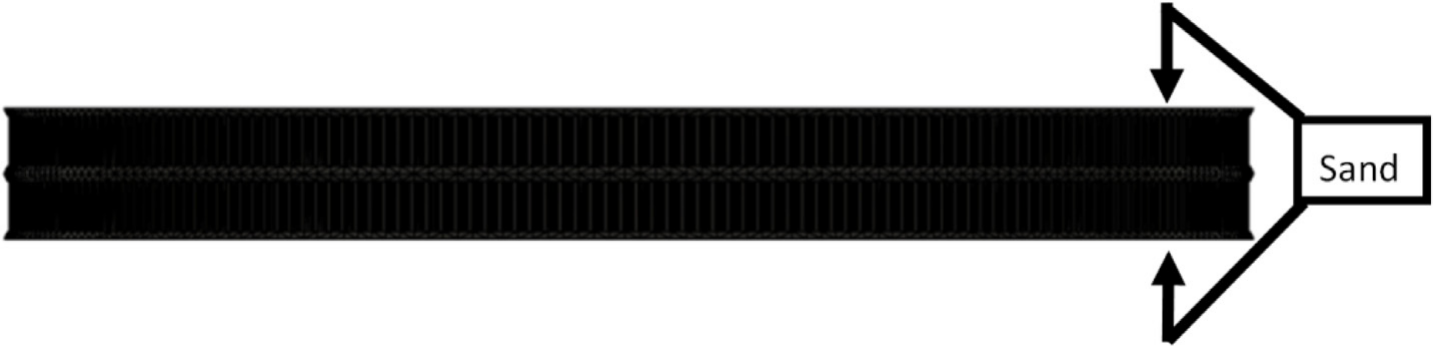
Areas to sand “GT2_spool_gear_double” part.

In this section, the assembly of the motor mounts and spools are shown. Modification of the motor mounts and spools have been made to accommodate for four Nema23 stepper motors rather than O-drives and BLDC motors used in the Hangprinter v4 [49]. The main design decision for the use of Nema 23 stepper motors is due to their (Fig. 29):

• Ease of use and integration with the DUET3 controller without the need of knowledge in python scripting and CAN communication.
• popularity in the 3-D printing community.
• low cost and high availability.

Furthermore, the end effector of this system is heavier than the filament extruder head used on the original hangprinter v4 design thus making Nema23 stepper motors a better choice than official v4′s BLDC motors for torque. The motor mount and spool assembly consist of 3D printed parts and off the shelf components such as gears, bearings, and screws.

**Fig. 30:**
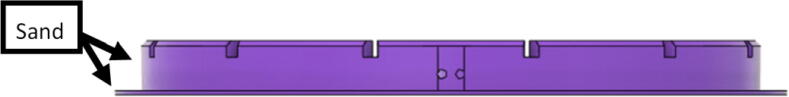
Areas to sand on “spool.stl” part.
• (1) “dleft_spool.stl”
• (1) “dright_spool_top.stl”
• (1) “dright_spool_bottom.stl”
• (3) “spool_cover.stl”
• (3) “spool_cover_mirrored.stl”
• (1) “dleft_spool_cover.stl”
• (1) “dright_spool_cover.stl”
• (4) “GT2_spool_gear_double.stl”
• (3) “ABC_motor_mount_spool.stl”
• (1)”D_motor_mount_spool.stl”
• (8) 608 (608ZZ) Bearings
• (16) 623 (F623ZZ)
• (16) M3x16mm Hex nylon screws
• (16) M3x50mm hex cap screw from M3 30–50 mm hex socket cap screw set
• (12) M3x35mm hex cap screw from M3 30–50 mm hex socket cap screw set
• (44) M3 nuts
• (1) 8 mm Steel Rod (650 mm length)
• (8) 20 tooth GT2 Gear
• (6) spectra line cut to size *l_ABC_* using Table 7 or
• (3) spectra line cut to size *l_D_* using or Table 7
  1. Print all of the above-mentioned spool components and inspect each part according to the guidelines outlined in [59] or [60], [61]. It is imperative that all the surfaces that the line will be in contact must be smooth for proper performance [49]. This includes the “GT2_spool_gear_double’s” inside edge. Do the same for the “spool” parts. Use a medium grit (80) sandpaper. For further details, read the “Areas That Must be Smooth” section in [49].
  2. Assemble the two “spool” parts to the “GT2_spool_gear_double” part as shown below in Fig. 31. Ensure that the inner teeth of the spool and the GT2 spool gear mesh together well as shown in Fig. 31. An audible tick or snap should indicate successful assembly. Insert two 608 bearings (one on each end) to the center holes of each spool. Test the flatness (or presence of warps) by inserting the 8 mm shaft into the bearing and rotating the spool on a shaft. If there is significant wobbling while spinning, the part is warped and thus should be reprinted. For more details, see “Make Sure Spools Rotate Without Wobble” section in [49].

Parts required:

• (6) “spool.stl” (Fig. 30)

**Fig. 31:**
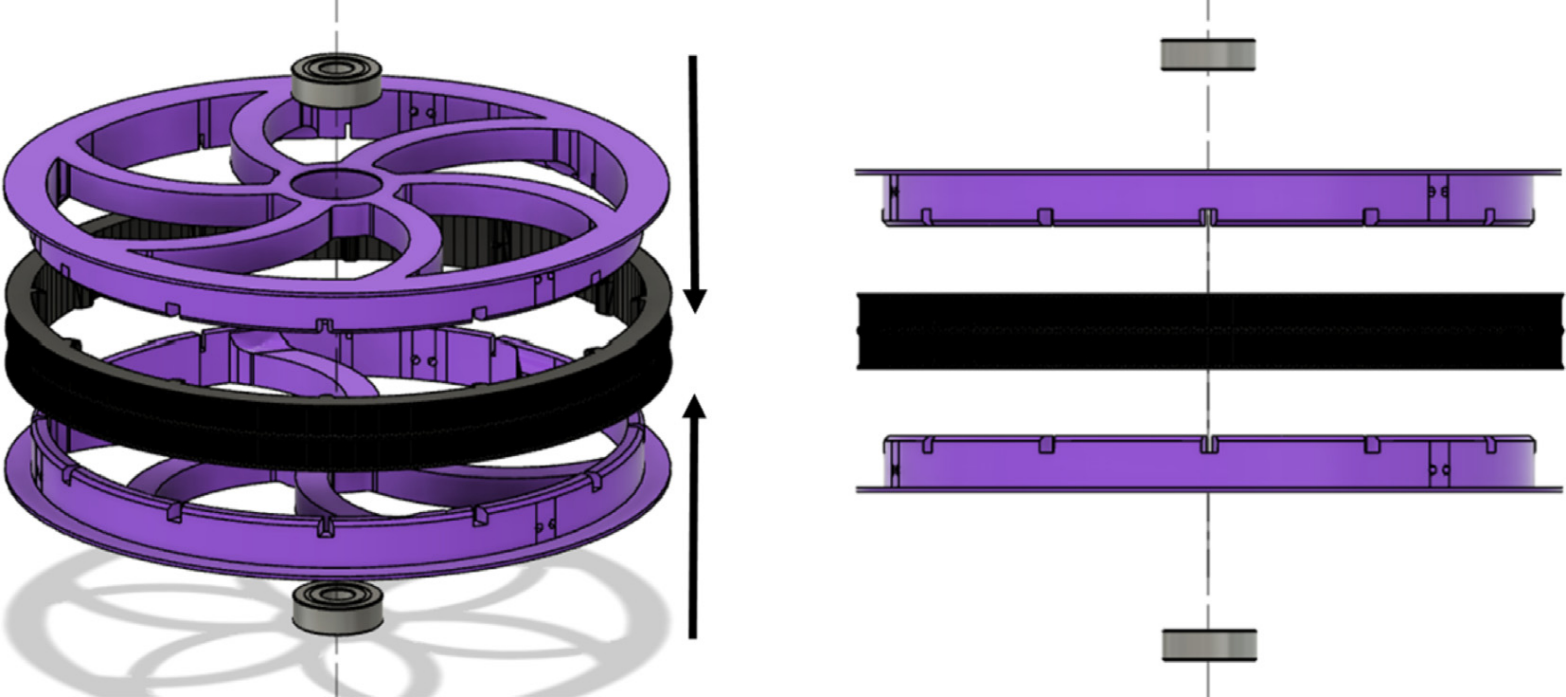
ABC Spool Assembly.
  3. Lightly apply super glue to the spool and GT2 gear interface to ensure the assembly does not come apart during operation. Sanding and filing of the teeth on the spool or GT2 gear may be required for successful assembly.
  4. Once assembled, attach two sets of spectra line, one on each spool half. Bring the line through one of the holes on the spool as shown in Fig. 32 and in Fig. 33.

**Fig. 32:**
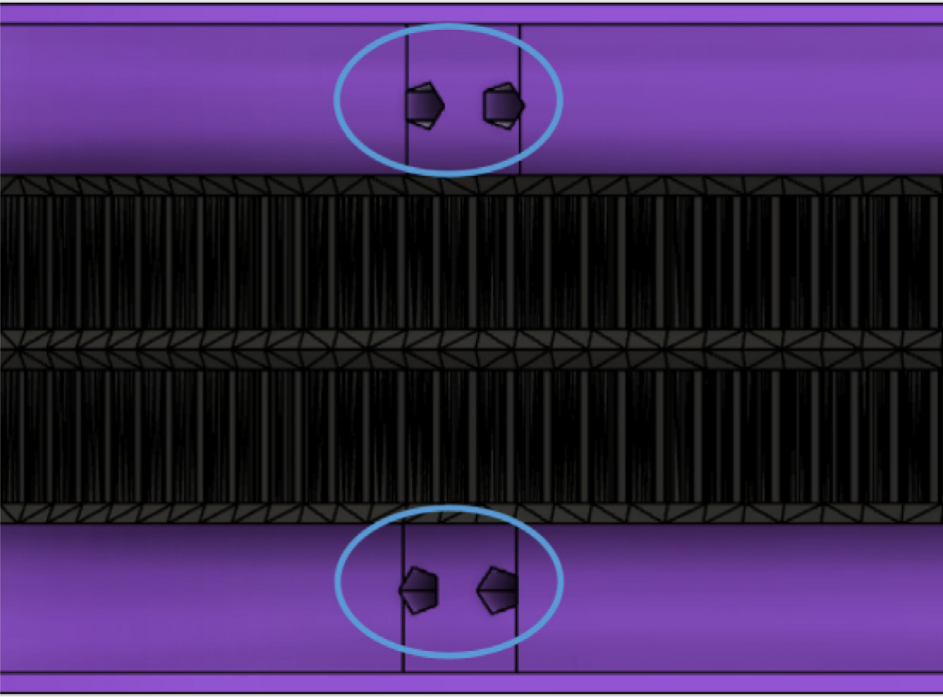
Knot location for spool lines. Bring line through hole and tie a knot inside the spool.

**Fig. 33:**
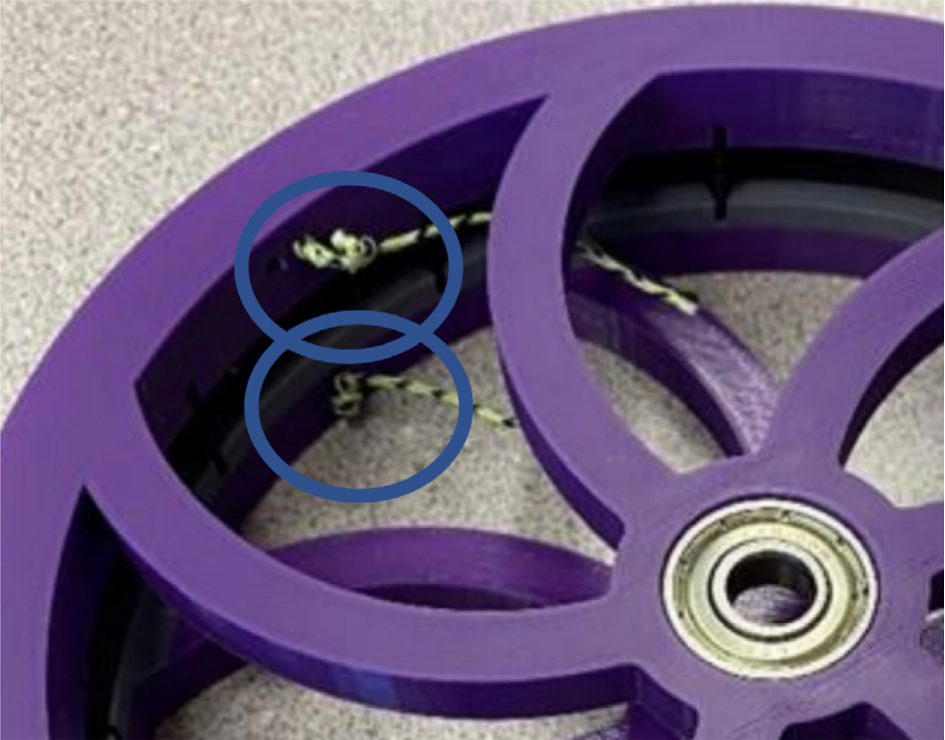
Knot Tied in Spool.
  5. Wrap the spectra line around the spool counterclockwise on one side, and clockwise on the other (with part flipped over), ensuring both exit the spool following the same path as seen, the A, B and C spools should each have 2 lengths of line as seen in Fig. 34*.* For each of A, B, C spools, there should be about 24 windings.

**Fig. 34:**
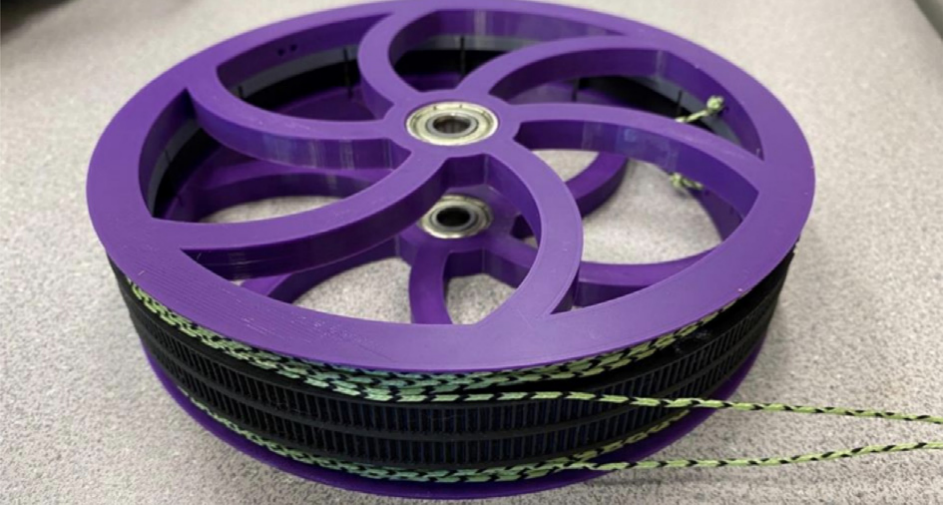
Wrapping spectra line around the spool.
  6. Repeat the above steps 1–5 for each A, B C spool.
  7. For the D spool, perform steps 1–5 using “dleft_spool”, “dright_spool_bottom” and “dright_spool_top” parts. Due to the D spool having 3 lines, there is a unique left and right spool. Two additional parts to be inserted are the “sep_disc” and “dleft_spool”. Assemble these according to Fig. 35*.*

**Fig. 35:**
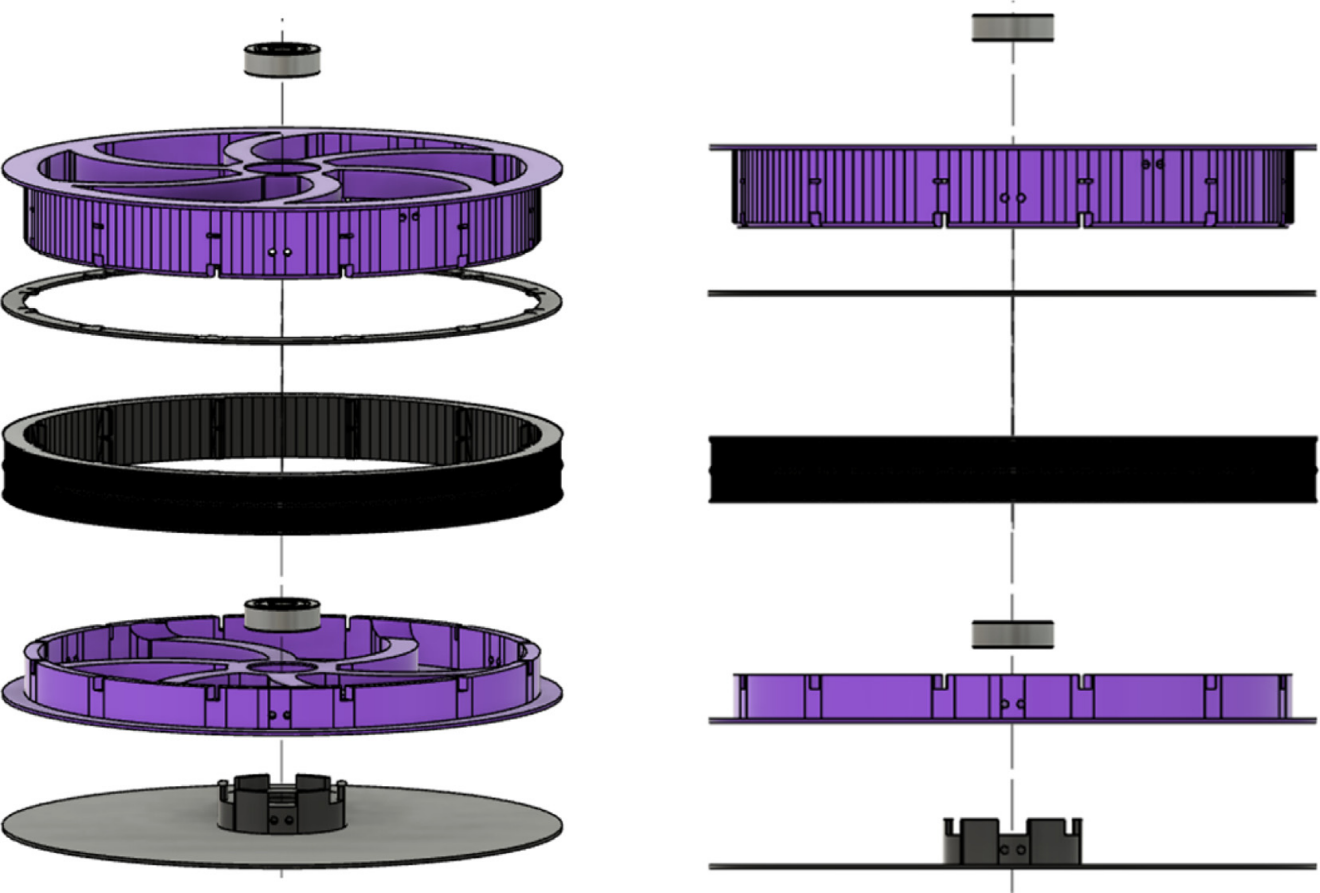
D spool assembly.
  8. Wrap the respective spectra line sized for the D spool similar to the way shown above for A, B and C spools with the addition of a third line. The D spool should have about 36 windings (Fig. 36).

**Fig. 36:**
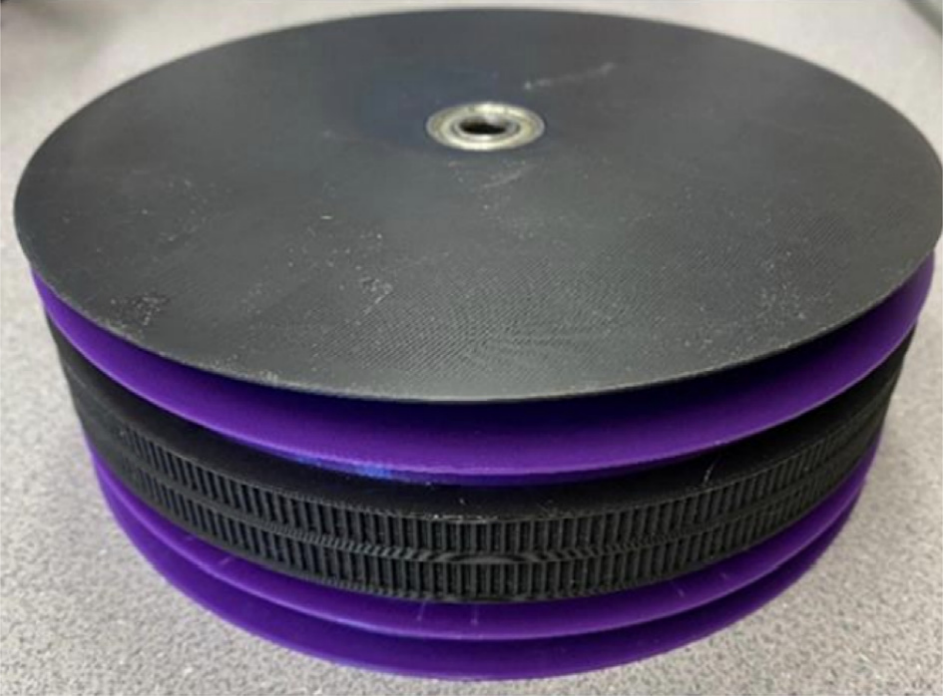
Assembled D spool.
  9. For all A, B, C, D spools, ensure that the spectra line is wrapped tight and apply some temporary tape to prevent the line from unravelling.
  10. Next, cut the 650 mm steel rod according to the following lengths and quantities:
  • Four 50 mm long shafts – for use on Nema23 motor and G2 20 teeth gears (Fig. 37).

**Fig. 37:**
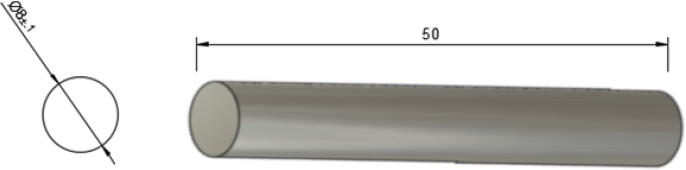
50 mm steel shaft cut.
  • One 100 mm long shaft – for use on D spool (Fig. 38).

**Fig. 38:**
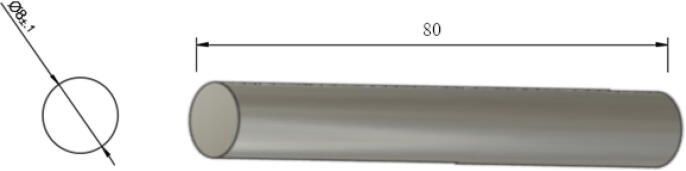
80 mm steel shaft cut.
  • Three 80 mm long shaft – for use on A, B, and C spools (Fig. 39).

**Fig. 39:**
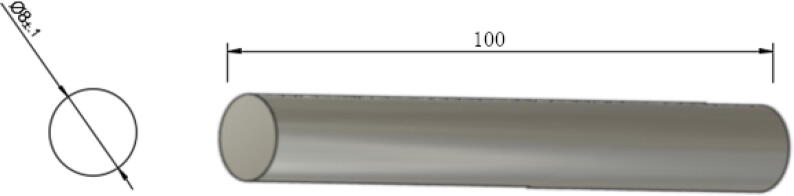
100 mm steel shaft cut.
  11. Assemble two GT2 20 tooth gear and tighten with 2 mm Allen key. Position them roughly in the center of the shaft to Fig. 40. Next, insert and secure the 8 mm to 10 mm shaft coupling on one end of the shaft. Tighten the screws with a 2 mm and 3 mm Allen key. Do this for all four 50 mm shafts.

**Fig. 40:**
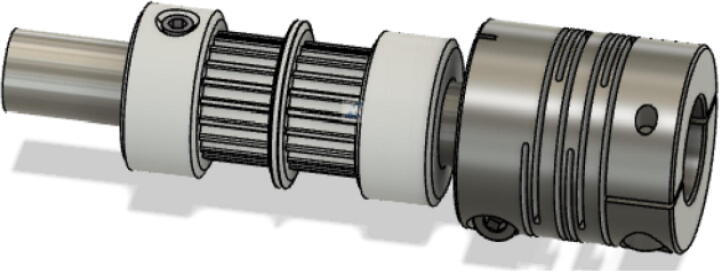
G2 gear arrangement on 50 mm shaft.
  12. Affix the Nema23 motor to the “ABC_motor_mount” and secure with four M3x16mm slotted nylon screws as shown in. Secure with M3 nuts. Insert a 608 bearing in its respective hole location opposite the motor holding plate. Finally, slide the “motor_holder” part along the Nema23 motor. Note that this “motor_holder” screws into the ceiling mount thus simply rest this component on the motor for now with some tape (Fig. 41).

**Fig. 41:**
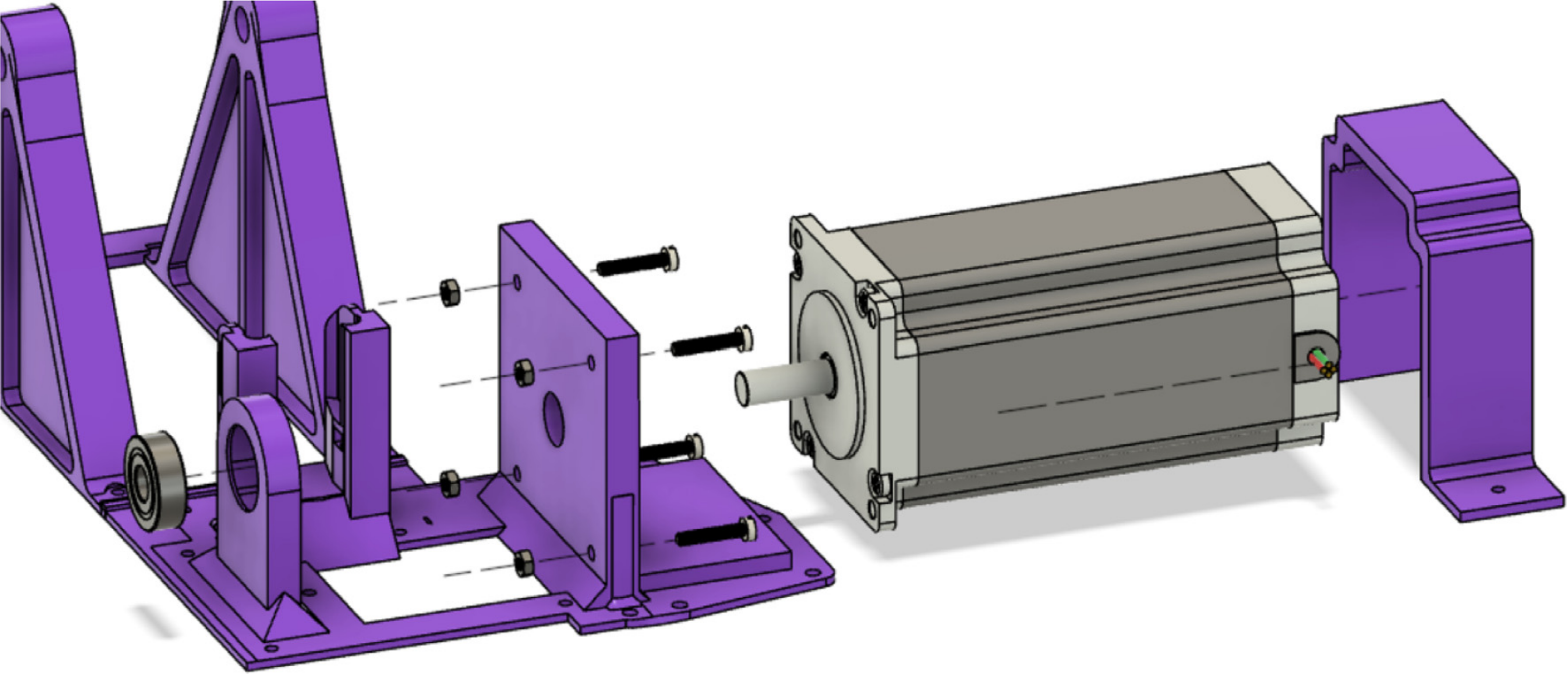
Nema23 motor assembly.
  13. Insert the “belt_roller_insert_extended” to the motor bracket as shown in. First insert the M3 nuts at the bottom of the belt roller walls. Then insert two M3x35mm hex cap screws as shown in Fig. 42. Position the four flanged F623ZZ bearings as shown in Fig. 42. To allow each belt to be contained within the flanges of each bearing pair. Slide a third M3x35mm hex cap screw through the belt roller insert and bearings and secure with a M3 nut on the other end.

**Fig. 42:**
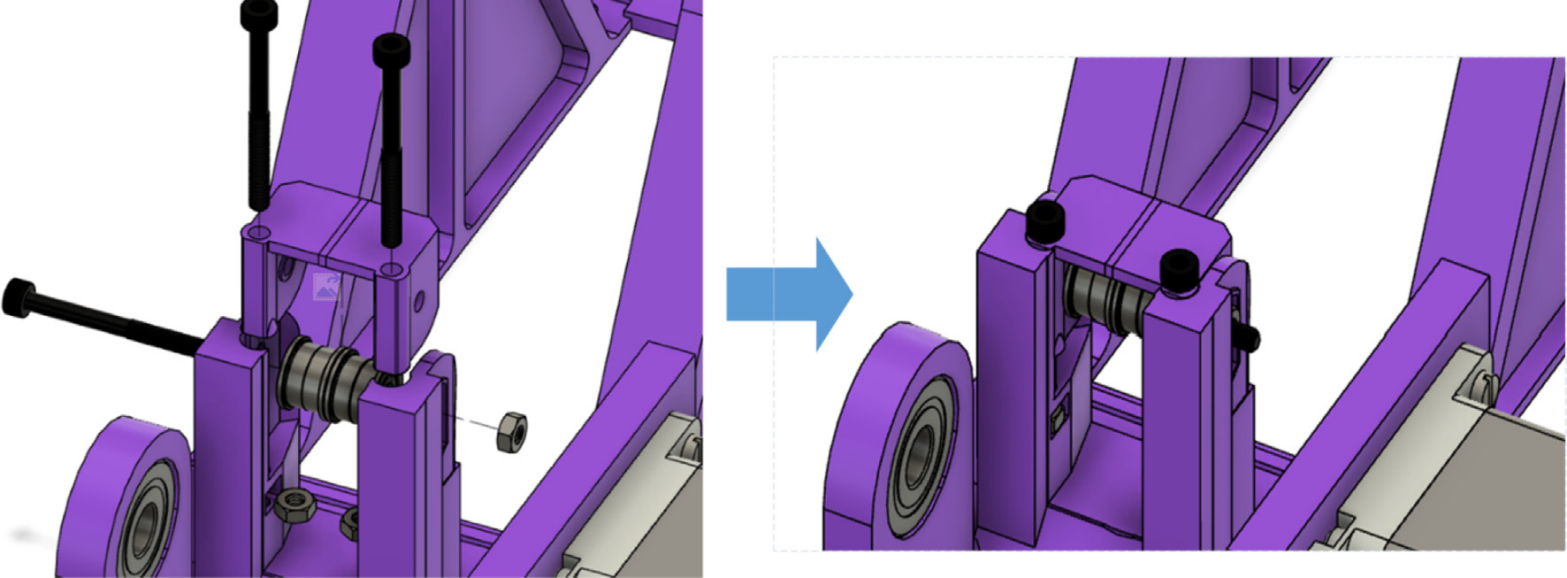
Belt roller insert assembly.
  14. Wrap the GT2 belt around the ABC spool assemblies made in step 2 according to Fig. 43. Then assemble the “spool_cover” and “spool_cover_mirrored” to the spool assembly using four M3x50mm hex cap screws and four M3 uts as shown in Fig. 44*.* Also ensure that the line is still contained in the spool and has not unraveled.

**Fig. 43:**
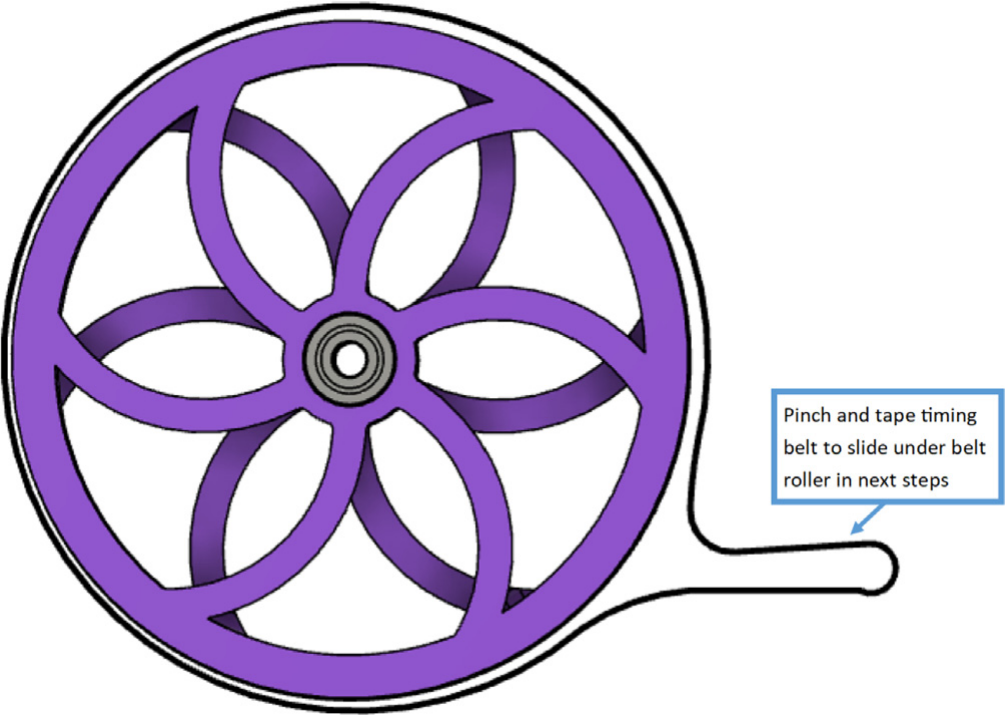
Wrapping timing belt around spool assembly.

**Fig. 44:**
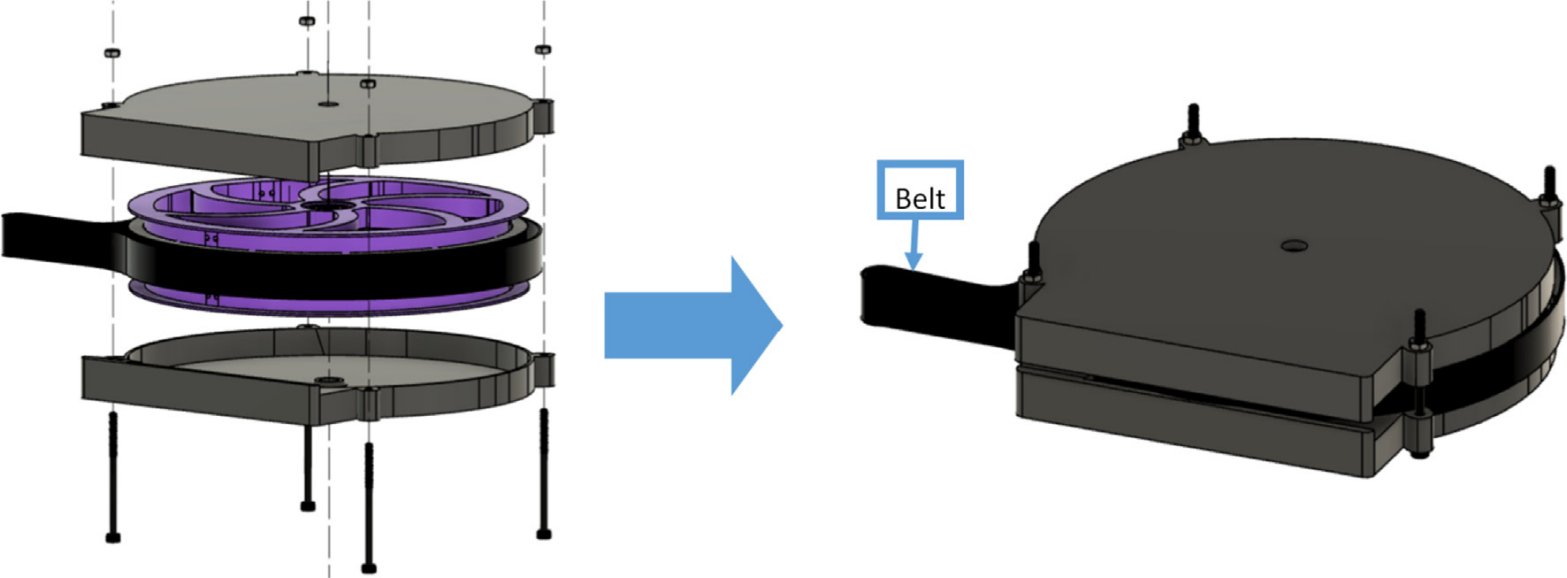
ABC spool cover and belt assembly.
  15. Insert the ABC spool assembly into the motor mount as shown in Fig. 45. Pull and pinch the belt as much as possible and slide it under the 623 flanged bearings under the belt roller assembly. Then using the 50 mm shaft assembly from Step 10, insert it through the two GT2 timing belts and secure one end of the into the shaft coupling and the other into the 608 bearing as shown in Fig. 45*.* Ensure that the teeth of the timing belt and gear teeth mesh properly and are separate by the flanges of the GT2 gears.

**Fig. 45:**
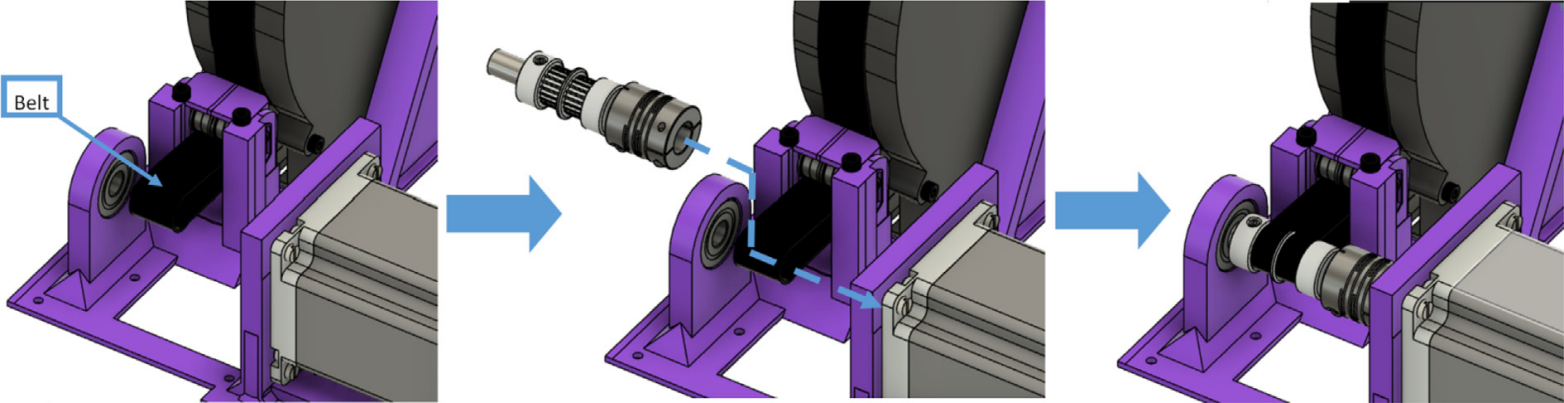
Connecting motor to spool via shaft and timing GT2 timing belt.
  16. Lastly, insert the 80 mm cut steel shaft into the spool and spool holder of the motor bracket to complete the entire ABC spool assembly as shown Fig. 46. Note that it will require the user to potentially pull the spool back in order t line up the holes of the spool and spool holder to freely slide the shaft in.

**Fig. 46:**
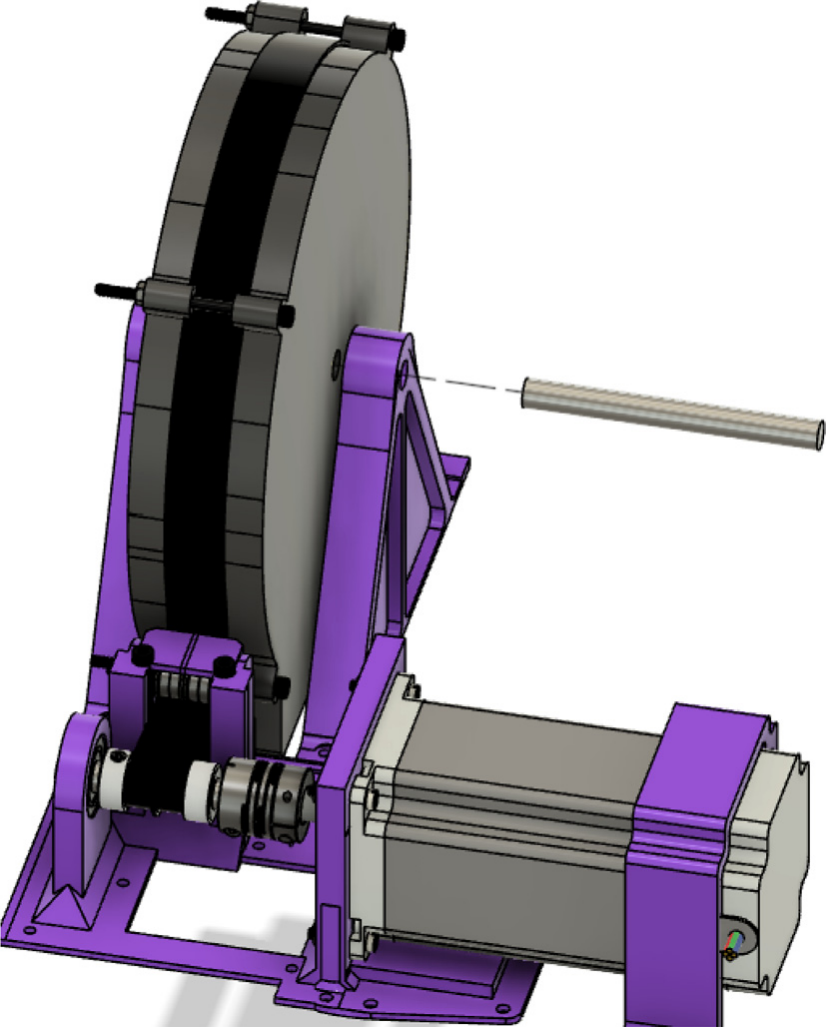
Completing the ABC spool assembly.
  17. Repeat steps 11–16 for the B, C and D anchors respectively. However, for the D anchors, be sure to use the corresponding D spool assembly, “dleft_spool_cover”, “dright_spool_cover”, “D_motor_mount and the 100 mm steel shaft instead of the 80 mm steel shaft.

**Fig. 47:**
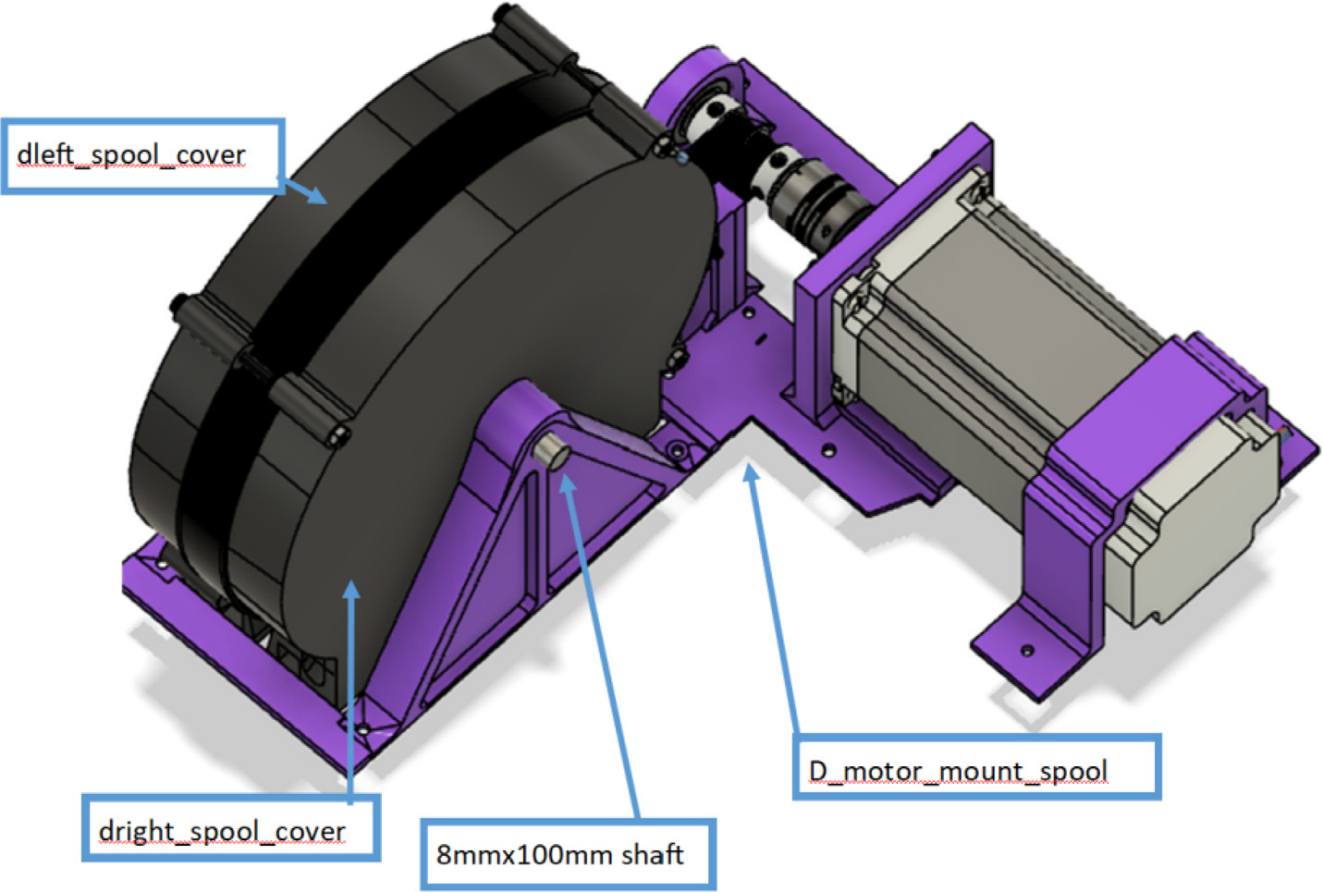
x1 D motor and spool assembly.

**Fig. 48:**
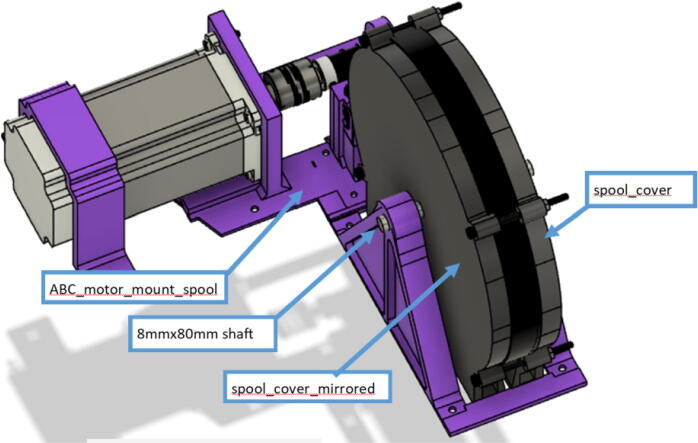
x3 ABC motor spool assembly.

A summary of the differences of the A,B,C and D motor and spool assemblies are shown below in Fig. 47 and Fig. 48.

#### Ceiling mount assembly

**Fig. 49:**
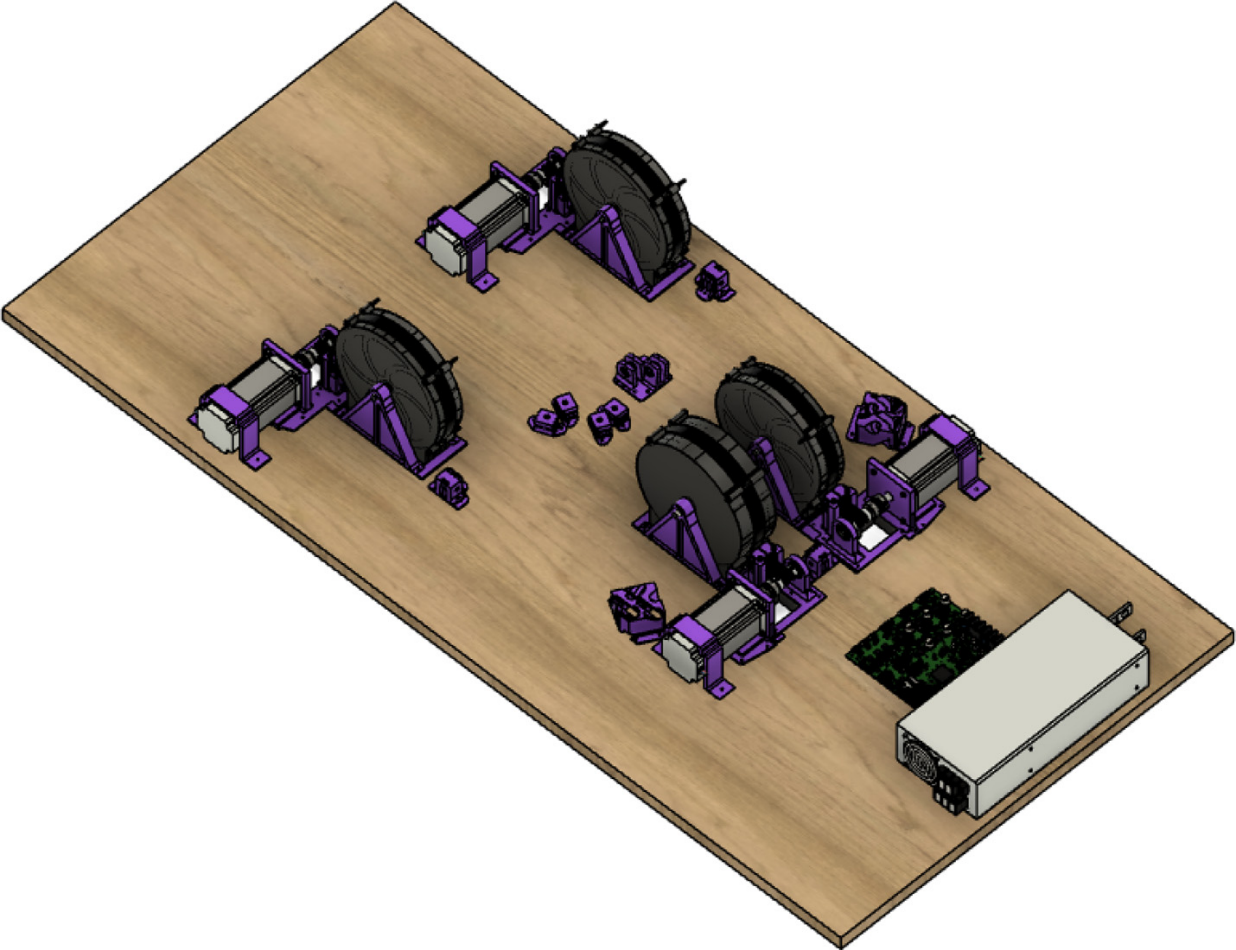
Hangprinter and electronic components layout on ceiling mount.

The ceiling mount comprises of all the printed and assembled hangprinter components as well as all the control electronics and power supply. Placement of these components are shown in Fig. 49.

Parts needed:

• MDF Board 2′x8′
• ABC motor mount assemblies
• D motor mount assembly
• assembled line roller and line roller anchor assemblies from section 5.1.2
• MEANWELL SE-1000–24 power supply
• DUET3 6HC Mainboard
• (>50)#8 × 5/8-inch round head wood screws
• #5x3/4 Flat Head Wood Screw
1. Reduce the length of the 2ft × 8ft MDF to 2ft × 4.5ft by securing the board on a flat tabletop with clamps. Mark a line 4.5ft down the length of the board and cut using a circular saw. Make sure to wear safety glasses and protective gloves.
2. To help make it easier for the user, a layout drawing has been prepared and can be printed to use as a template. The template can be found in [47].

**Fig. 50:**
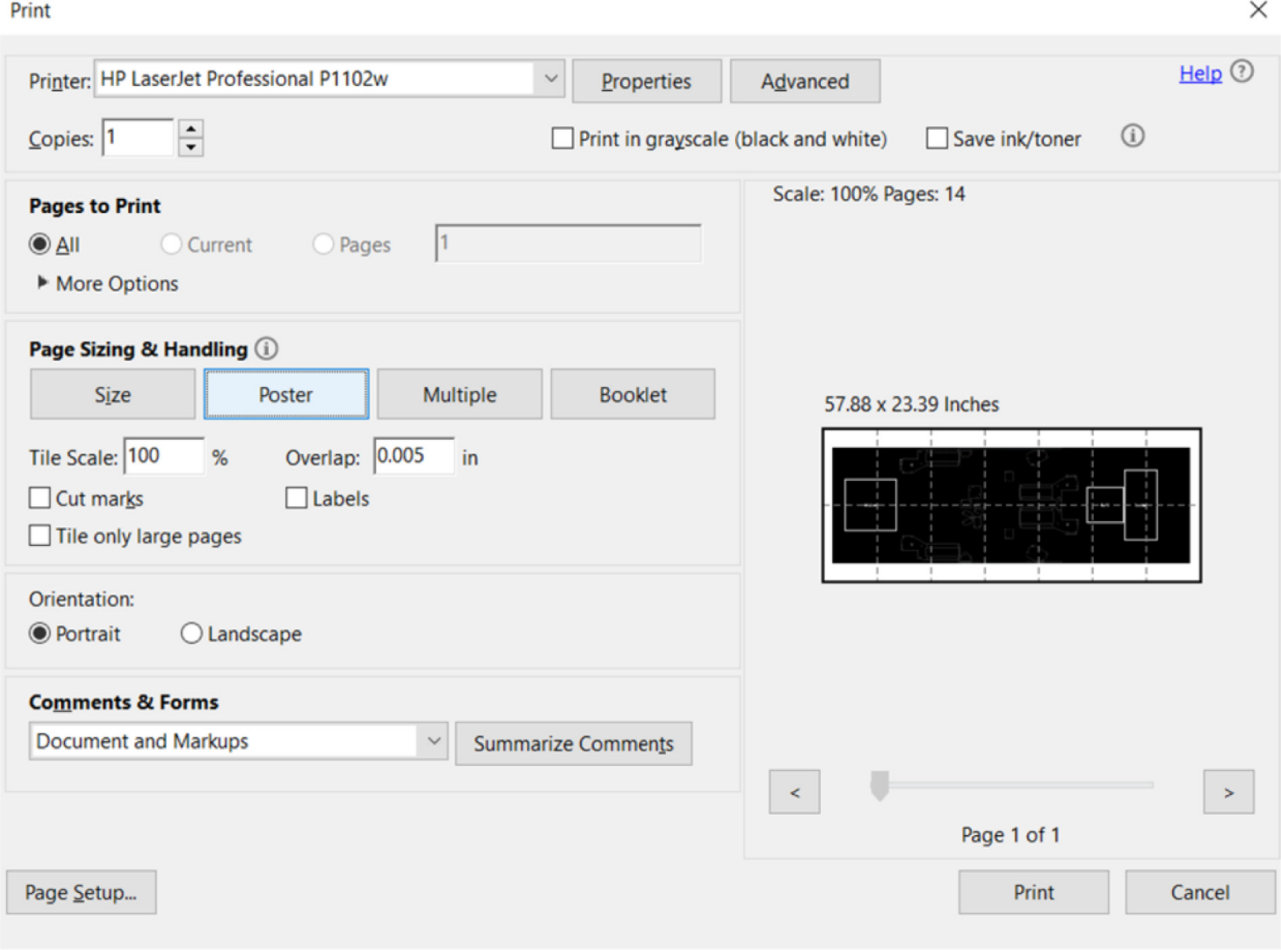
Print settings for poster printing template.

**Fig. 51:**
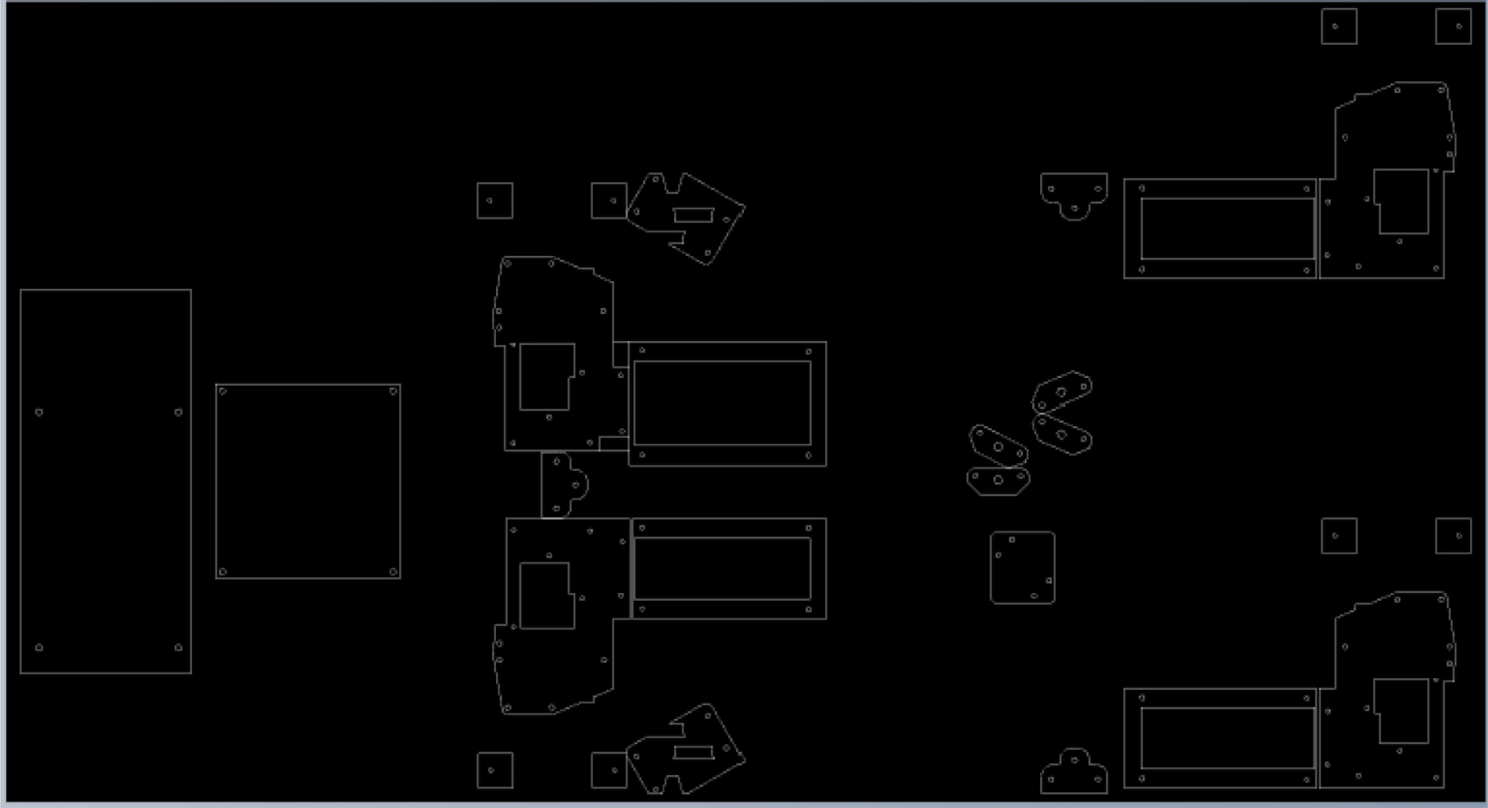
Ceiling_Mount_Layout template.

To do this, download the ‘Ceiling_Mount_layout. Open the pdf and select the ‘Poster’ option (Fig. 50: Print settings, this command splits a single image into multiple pages. Print out the layout into separate pages). Once the pages have been printed, staple each page in its respective location onto the MDF board. From here parts can be drilled right into the paper where holes for each respective part are allocated without the need for dimensioning (Fig. 51).

3. Mount all the hangprinter and electronics onto the ceiling mount ion their respective locations using the #8 × 5/8-inch round head wood screws for all printed components and for all the rest.

#### Line routing and attaching chassis

Tips:

• Do not fix anchors to the ground as they will be fixed during the calibration process.
• Unspool a large amount of line from each spool to ensure enough slack during routing.
  o For d-line routing, bring the Hangprinter chassis triangle end effector all the way to the ground.

Routing the d-line:

1. Unwound the spectra line from the d-spool assembly on the ceiling mount.
2. Route the three d-lines through their respective “horizontal line deflector” components on the ceiling mount as shown in Fig. 52*.*

**Fig. 52:**
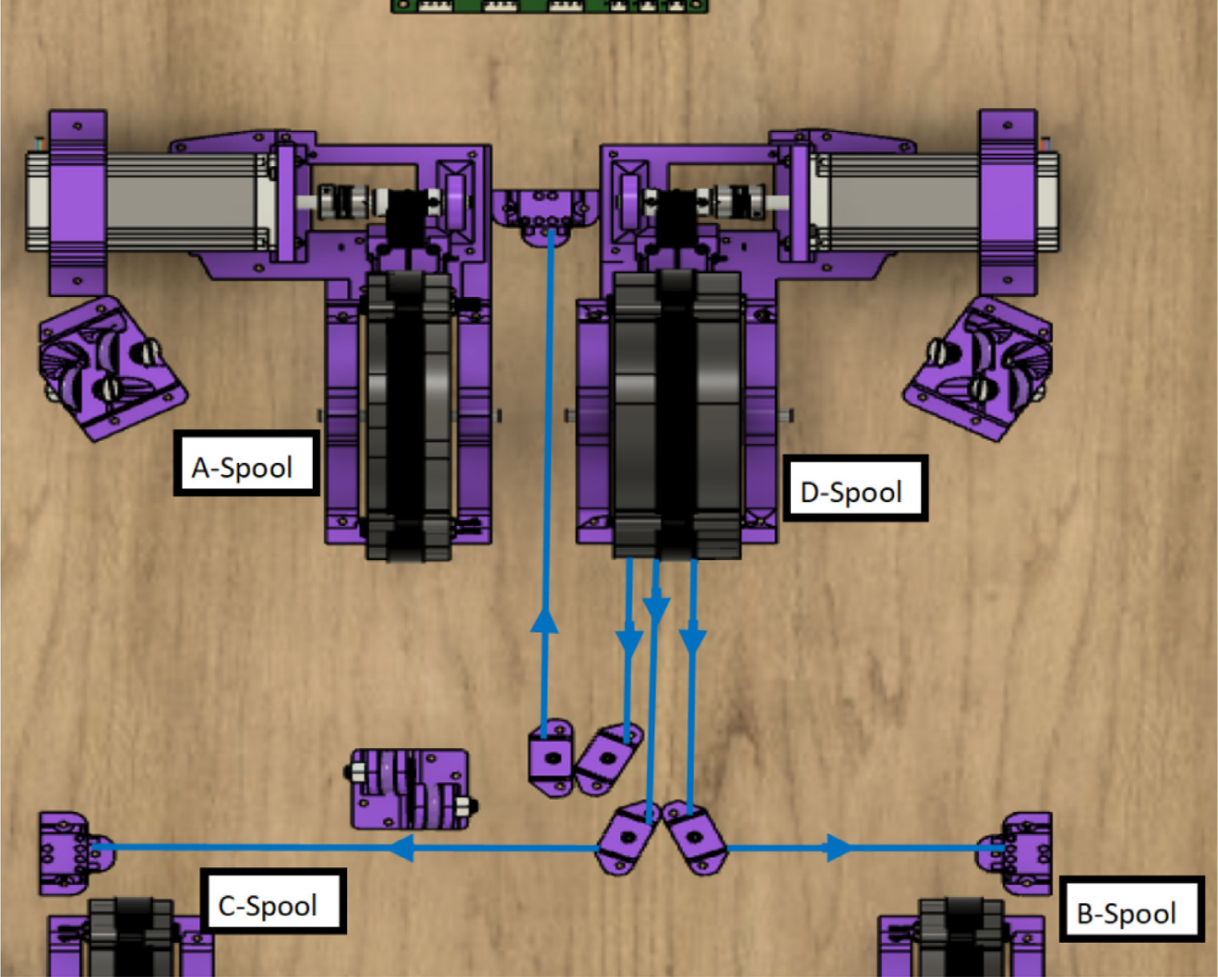
d-line ceiling mount routing to “horizontal line deflectors”.
3. D line connections to the chassis are made by routing each line through the ‘line_verticalizer’ to its respective ‘corner_clamp’ and back twice, being terminated against the board by a wood screw and washer as shown in Fig. 52*,* Fig. 53*,* Fig. 54*,* Fig. 55 and Fig. 56.
  a. By pulling the line from this end and adjusting the screw, the level of the Hangprinter chassis can be adjusted.
  b. Markers in the figures below are as follows (X = 1,2,3)
    i. DXA- first line from d-spool to Hangprinter chassis via “line verticalizer”.
    ii. DXB- from chassis “corner clamp” bearing back to “line verticalizer”.
    iii. DXC – second wound from “line verticalizer” to second chassis “corner clamp” bearing.
    iv. DXD – from second “corner clamp” bearing to “line verticalizer” and terminated on ceiling mount via screw and washer.

**Fig. 53:**
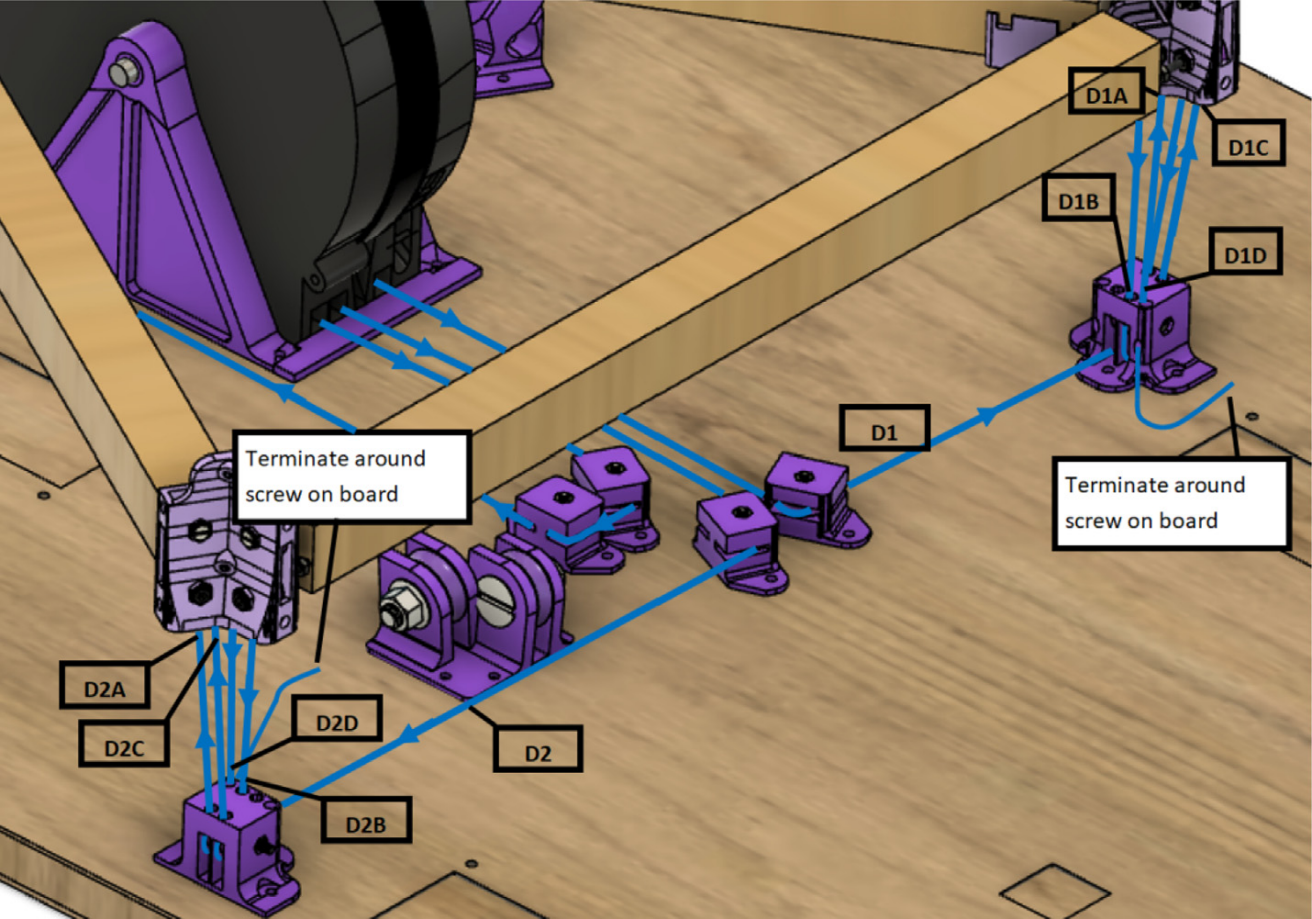
d-line routing from “line verticalizers” to chassis 1.

**Fig. 54:**
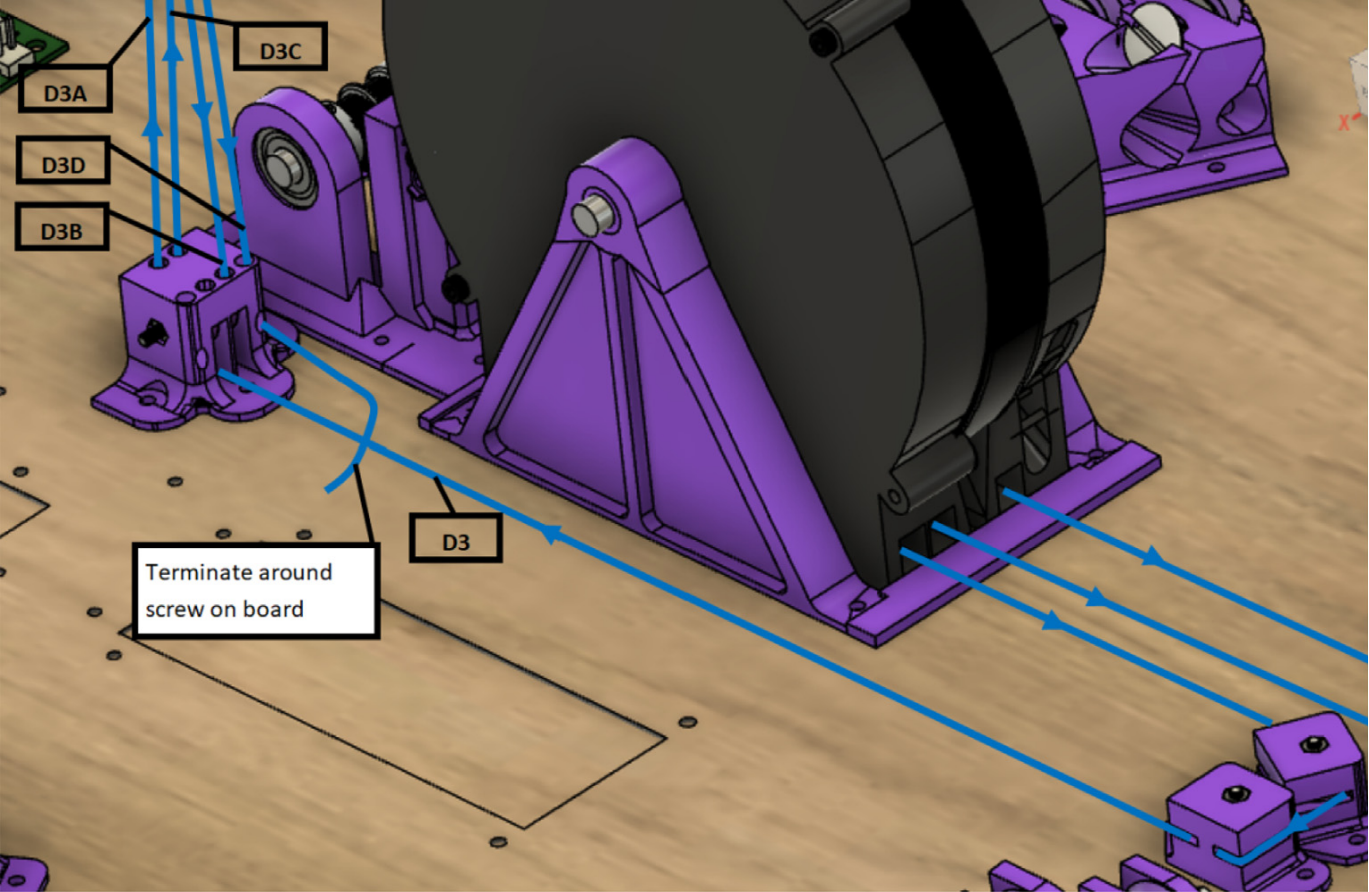
d-line routing from “line verticalizers” to chassis 2.

**Fig. 55:**
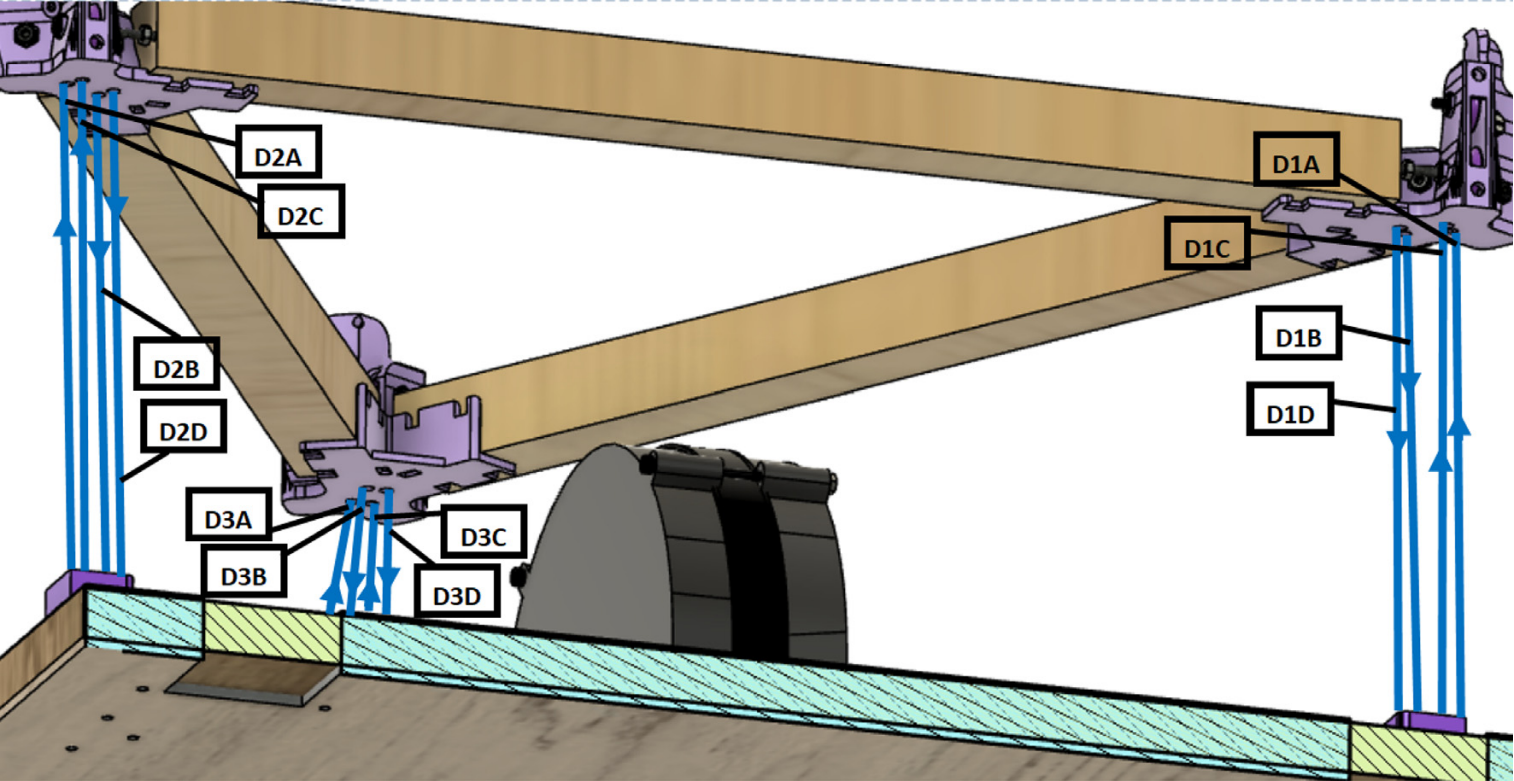
d-line routing from “line verticalizers” to chassis from chassis side.

**Fig. 56:**
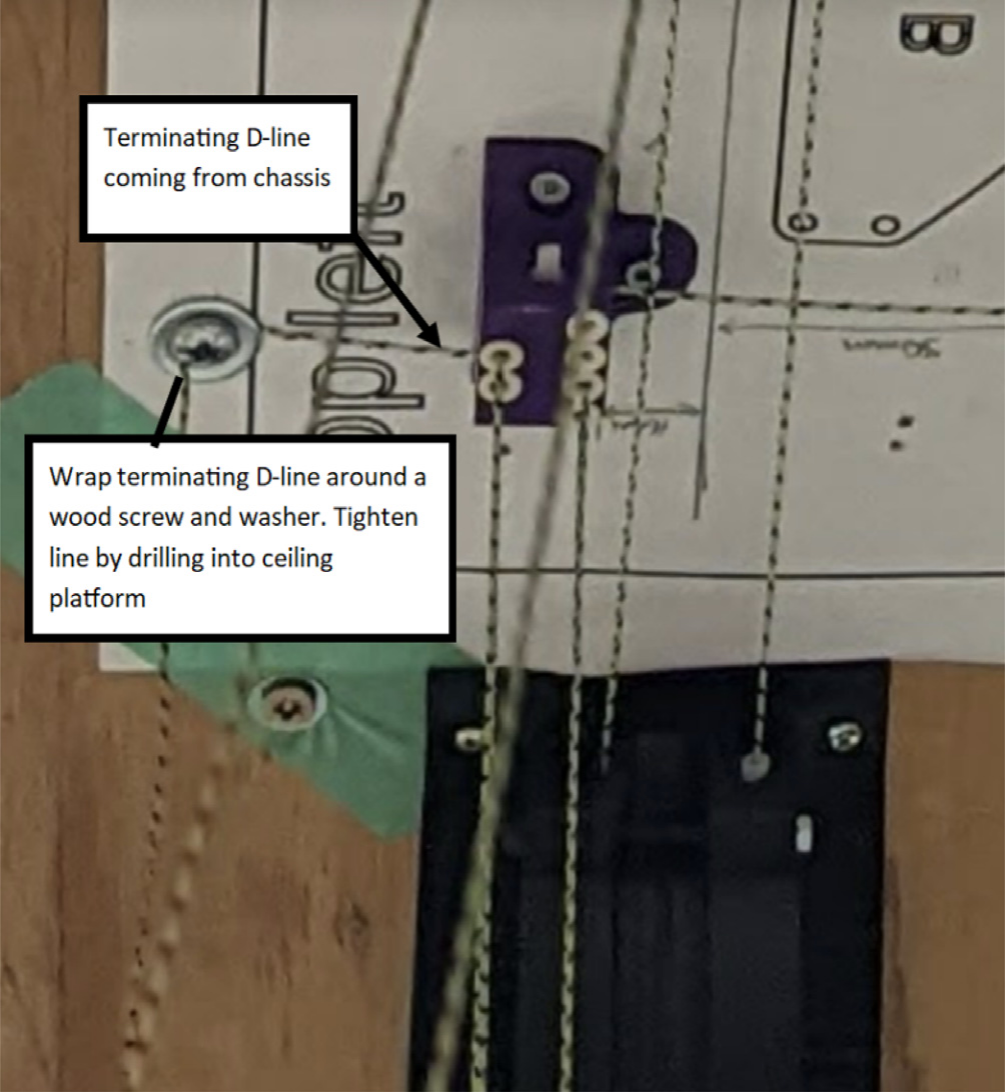
Tightening terminal end of routed d-line around screw on ceiling mount.

Routing the A-B-C-lines:

1. Route the A, B and C lines over the bearings of the tilted line deflector and line roller double components as shown in Fig. 57*.*

**Fig. 57:**
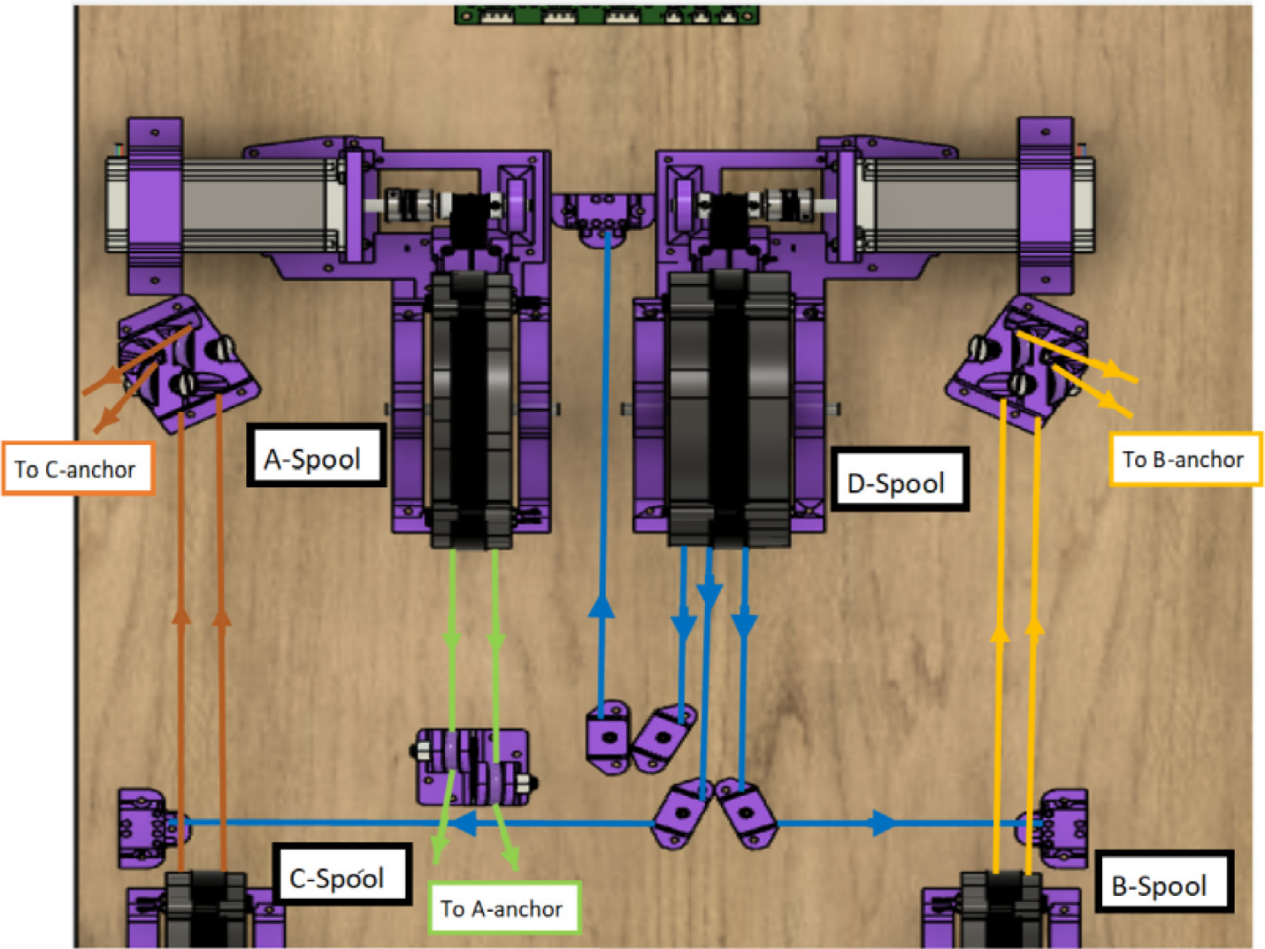
Ceiling mount side line routing for ABCD lines.
2. Route the lines through the line roller anchor and to the chassis with the end of the line terminating back at the top-of-the-line roller anchor as shown in Fig. 58*.*

**Fig. 58:**
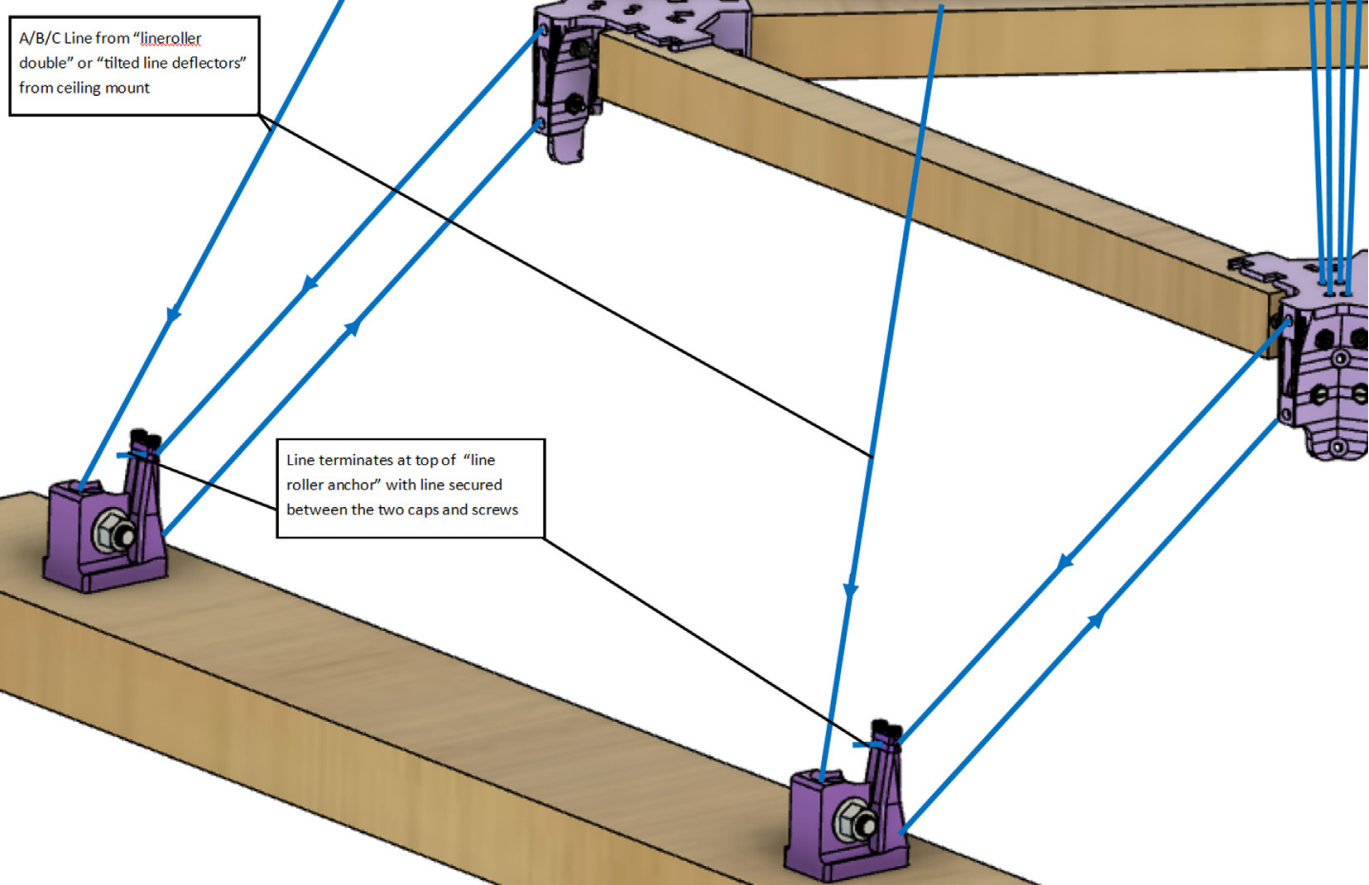
Routing ABC lines from anchor to chassis.

Note: The clamp on top of the printed “line roller anchor” component on the ground anchor is a safety mechanism. In presence of a sudden large force on the line (i.e. someone tripping on a line), the clamp should release the line. Thus, it is important that the line protruding on the back of the clamp is short.

The line routing of this hybrid hangprinter’s A, B, C and D lines are similar to that of the Hangprinter 4 and 3 versions. For more information or background on line routing, the user can visit the following resources:

• “Hangprinter Line routing: Quick, Silent Overview” [64]
  o Very similar routing to this project however note that the spool layouts of the B and C motors are different due to larger NEMA 23 stepper motors.
  o Positions of all line routing components on the ceiling mount are the same.
  o In the video, the ABC lines are tied together at the anchor points, however this is outdated; lines are now terminated at the anchor via the “line_roller_anchor” parts.
• “HangPrinter v3 Part 1 - The Build - Chris's Basement” [65]
  o Similar concept for line routing in V3 but single lines and different style of termination and spooling.

#### Print bed

Any large platform can be used as a print bed for the Hangprinter. Consequently, this item and its construction are excluded from the project scope. For this specific instance, a 42″ by 30″ tabletop was detached from its legs and used as the print bed. 4 nuts and bolts were used in the 4 corners of the bed to adjust its level. Alternatively, the user can also choose to treat the ground as the print bed. It is recommended to cover the print area with painters’ tape or masking tape unless a specialty bed is being created.

This completes the mechanical component of the Hangprinter assembly. See below sections for assembly of the pellet extruder and pellet feed system.

### Pellet extruder

The pellet extruder consists largely of off the shelf and 3-D printed components however there are two primary components that use subtractive manufacturing methods such as milling, turning and tapping. More on these components is explained further below. For information on the 3-D printed components, refer to Table 1. Furthermore, there are two off the shelf components (nozzle adapter and 2 mm nozzle) that also require some simple machining operations. Instructions for these are also described at the end of the section below.

#### Machining the components for the barrel assembly

The components of the barrel assembly consist of an aluminum heat dissipation block, 304 steel barrel, a 304 steel nozzle adapter and a 2 mm standard printer nozzle. The heat dissipation block (Aluminum_Heatsink_Block) and the barrel (Steel_Barrel) for this specific project were fabricated from a 3rd party machining service. Use the drawings provided in [47] in the “Electrical Schematics and Part Drawings” section to provide to a metal fabrication shop or to use as reference if machining on your own. While off the shelf options were explored for these components, nothing was found that would suit the desired auger size and thermal insulation thus, the design strategy was to design a custom heat dissipation block and barrel around the size of other components such as common heating band sizes and nozzle adapter thread sizes. Also, to make the design modular and easy to disassembly for cleaning, the threads on each of these parts made for easy disassembly.

Due to variance in rates/quotes for outsourcing the fabrication of these 2 components, Xometry is used to provide a universal, base quotation for both parts. Xometry is a custom fabrication service that offers both additive and subtractive manufacturing capabilities and instant quoting [66]. Follow the steps below to obtain an instant quote and order the fabrication of these parts.

Steps for Steel_Barrel:

1. Go to Xometry.com [66] and upload the “Steel_Barrel.sldprt” file found in [47] using the “get your instant quote” button.
2. Click on “Modify Part”.
3. Select the CNC machining option under the “Process” tab. Change material to 304 Stainless Steel under the “Material” tab.
4. Under “Part Features”, specify the number of “Threads and Tapped Holes” to 2. A message will appear to upload a technical drawing of the part. Upload the “Steel_Barrel.pdf” drawing found in [47] in the “Electrical Schematics and Part Drawings” section.
5. Leave every other setting as is and click the “Save Properties” button.
6. The preview section of the quote should look like Fig. 59*.*

**Fig. 59:**
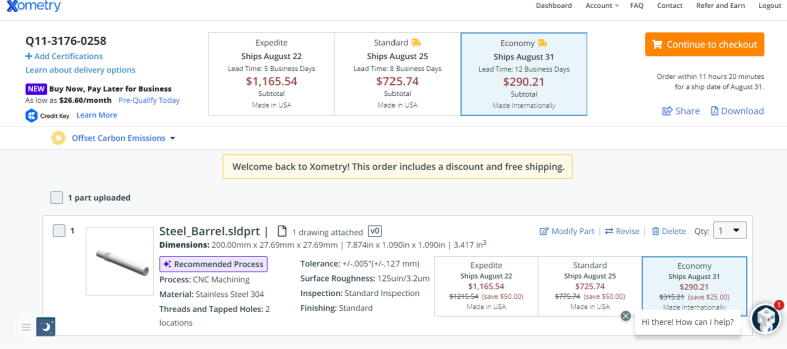
Xometry instant quotation for Steel_Barrel part.
7. To reduce shipping costs, select the Economy option as shown in Fig. 59*.*
8. Click checkout and follow the onscreen instructions to order.

Steps for Aluminum_Heatsink_Block:

1. Go to Xometry.com[66] and upload the “Aluminum_Heatsink_Block” file found in [47] using the “get your instant quote” button.
2. Click on “Modify Part”.
3. Select the CNC machining option under the “Process” tab. Change material to 6061 Aluminum under the “Material” tab.
4. Under “Part Features”, specify the number of “Threads and Tapped Holes” to 5. A message will appear to upload a technical drawing of the part. Upload the “Aluminum_Heatsink_Block.pdf” drawing found in [47] in the “Electrical Schematics and Part Drawings” section.
5. Leave every other setting as is and click the “Save Properties” button.
6. The preview section of the quote should look like Fig. 60*.*

**Fig. 60:**
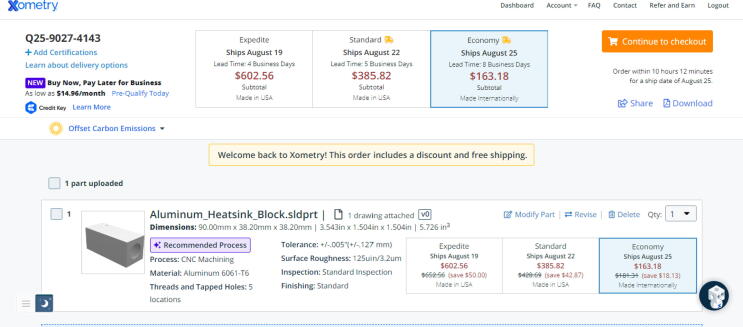
Xometry instant quotation for Aluminum_Heatsink_Block part.
7. To reduce shipping costs, select the Economy option as shown in Fig. 60*.*
8. Click checkout and follow the onscreen instructions to order.

**Fig. 61:**
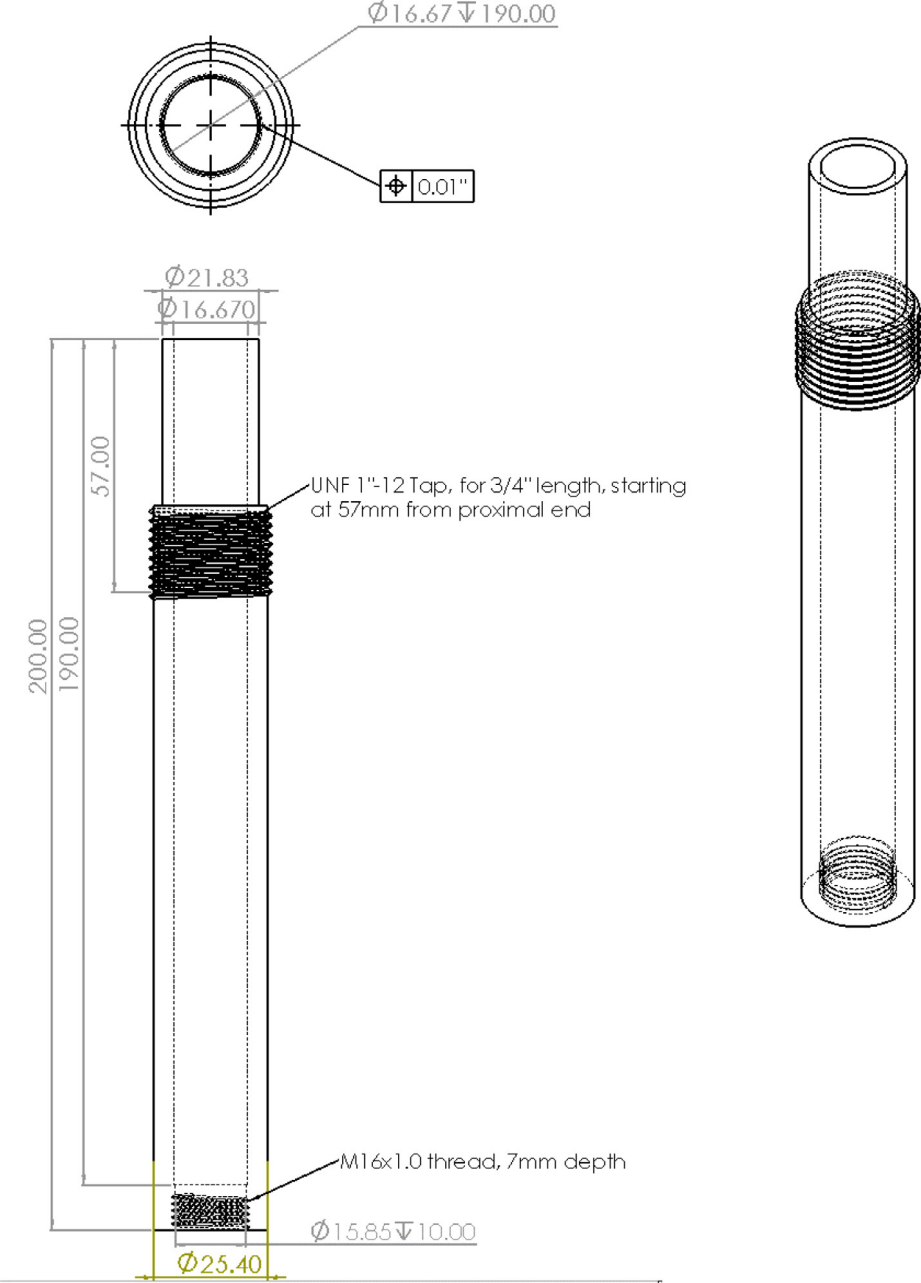
Features and Dimensions for Steel Barrel Part.

**Fig. 62:**
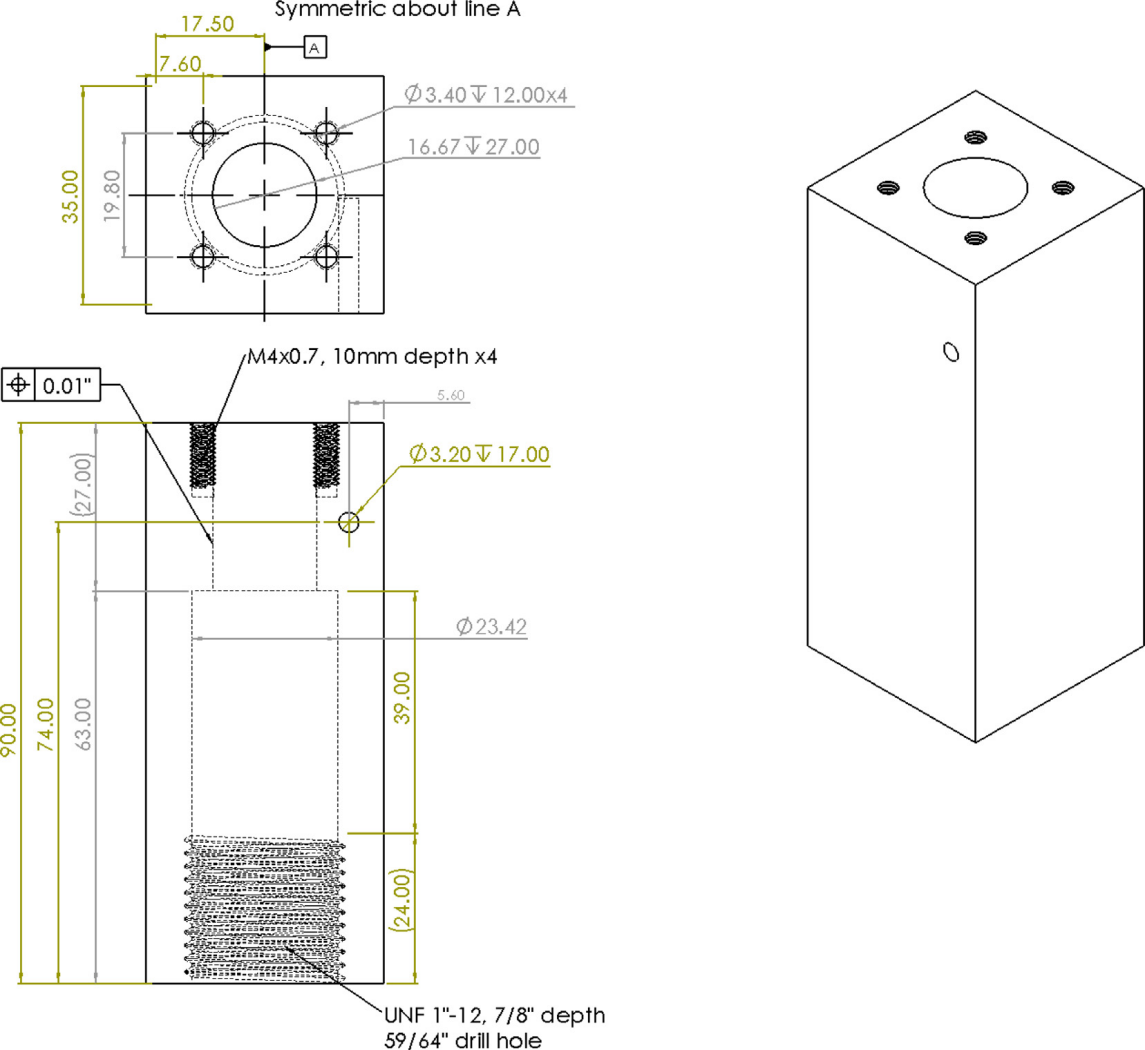
Features and Dimensions for Aluminum_Heatsink_Block Part.

Alternatively, these two parts can also be fabricated manually either by the appropriate machines such as a milling machine, drill press and lathe. If no access to a milling or lathe, some additional tools are needed to produce the features of the parts shown in Fig. 61 and Fig. 62. To produce these features by hand, the following parts are needed (as per drawings found in [47]):

• 304 Stainless Steel Round Tube, 0.188″ Wall Thick, 1ft - stock for “Steel_Barrel”
• Aluminum Square Bar 6061 T6511 1.500, 4″ Length -stock for “Aluminum_Heatsink_Block”
• M6 × 1.0 Female to M16 × 1.0 Male Nozzle Adapter
• 2 mm steel or copper 3d printer nozzle
• M4x0.7 thread tap -for step 9 on “Aluminum_Heatsink_Block) [67]
• M16x1.0 thread tap – for step 5 on “Steel_Barrel” [67]
• 1″-12 UNF thread tap – for step 12 on”Aluminum_Heatsink_Block” [67]
• 1″-12 UNF thread die – for step 4 on “Steel_Barrel” [67]
• 9/64″ (3.4 mm) Titanium-nitride coated drill bit [67]
  o For drilling clearance hole for M4x0.7 tap on “Alumnum_Heatsink_Block”
  o For drilling RTD hole in nozzle adapter and expanding nozzle orifice
• 21/32″, 12″ long Black Oxide HSS Drill bit [67]
  o For step 1 and 6 below
• 15/16″ diameter, 3″ Cutting Depth, H.S.S. Annular Cutters – for cutting clearance hole for 1″-12 UNF thread in steps 10 and 11 [68]
• Vise
• Tap and die handle

Tips for fabrication if no access to lathe or milling machine (skip if you have a lathe and milling machine with appropriate tools):

• Use a vise for proper work holding.
• Wear safety glasses and protective gloves.
• Instructions on how to tap internal and external threads can easily be found on the internet.
• Turning operations such as step 3 below can be recreated on a drill press and using a sharp file and special work holding setup for those who do not have access to a lathe using this resource [69].
• Use a drill press and corresponding drill bit for the boring operations listed above for both the steel tube stock and aluminum stock.
• If using a drill press and the 21/32″ black oxide HSS drill bit for step 2 and 7 below, drill at a slow RPM and use coolant. Drill using multiple passes to remove chips. Ensure that work piece is tightly clamps down on the work table or secured in a vise.
• If using a drill press and the 15/16″, 3″ deep annular cutter for step 10, cut at a slow RPM with the workpiece rigidly secured on the worktable or vise. Use coolant. Drill using multiple passes to remove chips.
  o Annular cutters require less cutting force than twist drills as they only cut the diameter and not the material inside, leaving a slug/coupon of cylindrical material inside.
  o If the thickness of the annular cutter results in a slug/coupon with a diameter less than the resulting bore from step 7, the slug/coupon should simply fall off.
  o If the resulting slug/coupon is bigger than the bore from step 7, then subsequent machining steps to remove to slug/coupon will need to be done. Boring with an intermediate drill bit size corresponding to the slug/coupon diameter should remove it.

Order of operations for Steel_Barrel:

1. Cut the 304 round steel tube to a length of 200 mm (7.87″).
2. Bore/expand the inner diameter of the stock steel tube from 15.8 mm (0.624″) to 16.67 mm (0.656″) at a depth of 190 mm (7.48″).
3. From the top of the tube, machine down the outer diameter of the tube from 25.4 mm (1″) to 21.83 mm (55/64″).
4. Starting from the top of the tube machine down the outer diameter to 23.62 mm (0.930″) and 37.95 mm (1.494″) deep to allow for external thread feature to be created.
5. Create an external thread starting at 37.95 mm (1.494″) b”) below the top of the tube. The specification is a UNF 1″-12 tap at a length of 19 mm (0.75″).
6. Create a M16x1.0, 7 mm deep internal thread at the bottom of the tube (unbored internal diameter).

Order of operations for Aluminum_Heatsink_Block:

7. Drill/mill a 16.67 mm (0.656″), 27 mm (1.06″) deep hole in the center of the top face of the block.
8. Drill four, 3.4 mm (0.134″), 12 mm (0.472″) deep holes according to the dimensions shown in Fig. 62*.*
9. Drill a 3.2 mm (0.134″),17 mm deep hole on the face adjacent to the top face as shown in Fig. 62*.*
10. Using a M4x0.7 tap, thread the holes from step 6 to a depth of 10 mm.
11. On the bottom face (opposite to the face in step 5) of the block, drill/mill a 23.42 mm (59/64″) hole at a depth of 63 mm (2.48″). The opening of the 16.67 mm hole from step 5 should be visible at this point. File/de-burr any rough edges.
12. Using a UNF 1″-12 tap, thread the interior of the hole at a depth of 24 mm (7/8″).

The last two machined components are the nozzle adapter and 2 mm printer nozzle. The nozzle is more specifically a M6 × 1.0 female to M16 × 1.0 male stainless-steel adapter that is available online for purchase thus does not need to be machined from stock. Therefore, manually tapping a M16 thread on the steel barrel is required so that the adapter can be screwed in. Both the nozzle adapter and the 2 mm printer nozzle need to have a 3 mm (0.125″) hole drilled into them. The hole on the nozzle adapter is for inserting a PT1000 RTD to monitor the nozzle and barrel temperature. For the nozzle itself, the 2 mm orifice needs to be expanded to 3 mm due to the project requirements. Both operations can be done on a drill press and work holding tool.

**Fig. 63:**
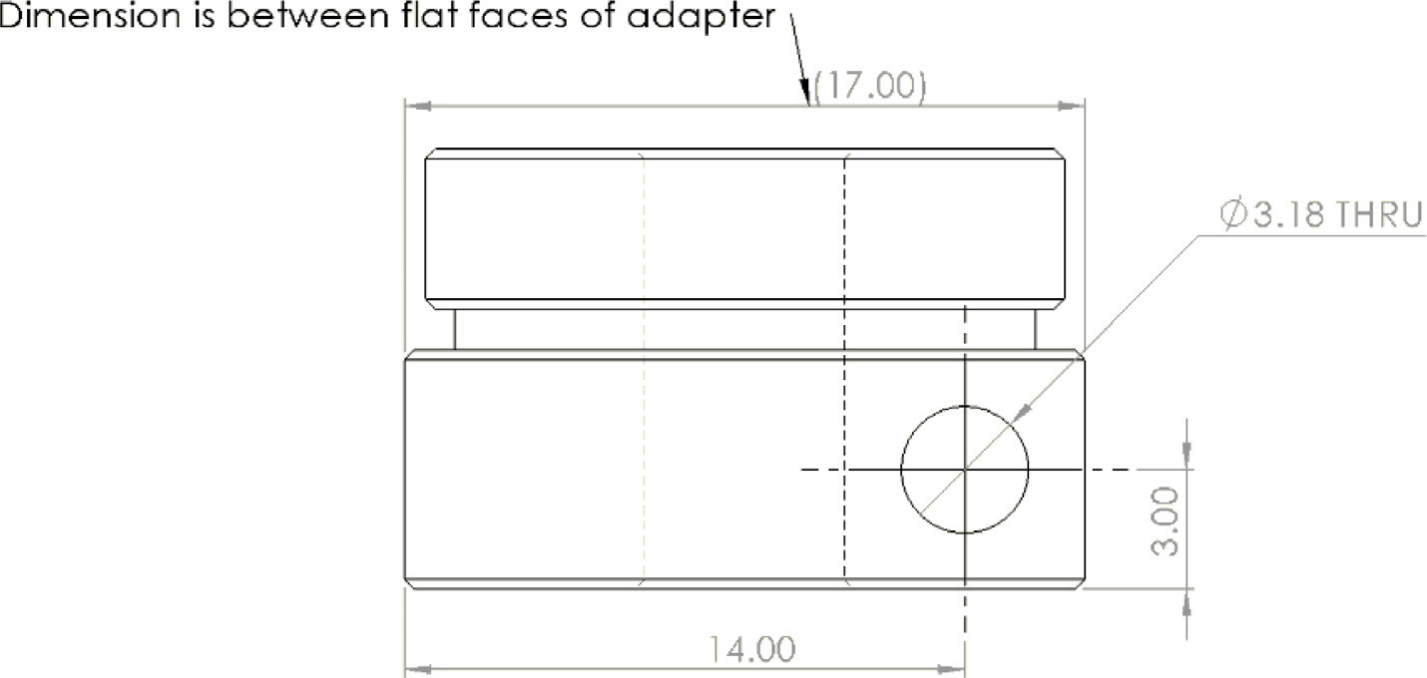
RTD Hole Location on Nozzle Adapter.

Nozzle Adapter:

1. Secure the nozzle adapter in a vise or on the worktable of a drill press.
2. Insert a 9/64″ (3.4 mm) drill bit into the chuck. A cobalt or titanium-coated drill bit is suitable for cutting through hard steel.
3. Position the tool head and work piece according to Fig. 63 and Fig. 64*.*

**Fig. 64:**
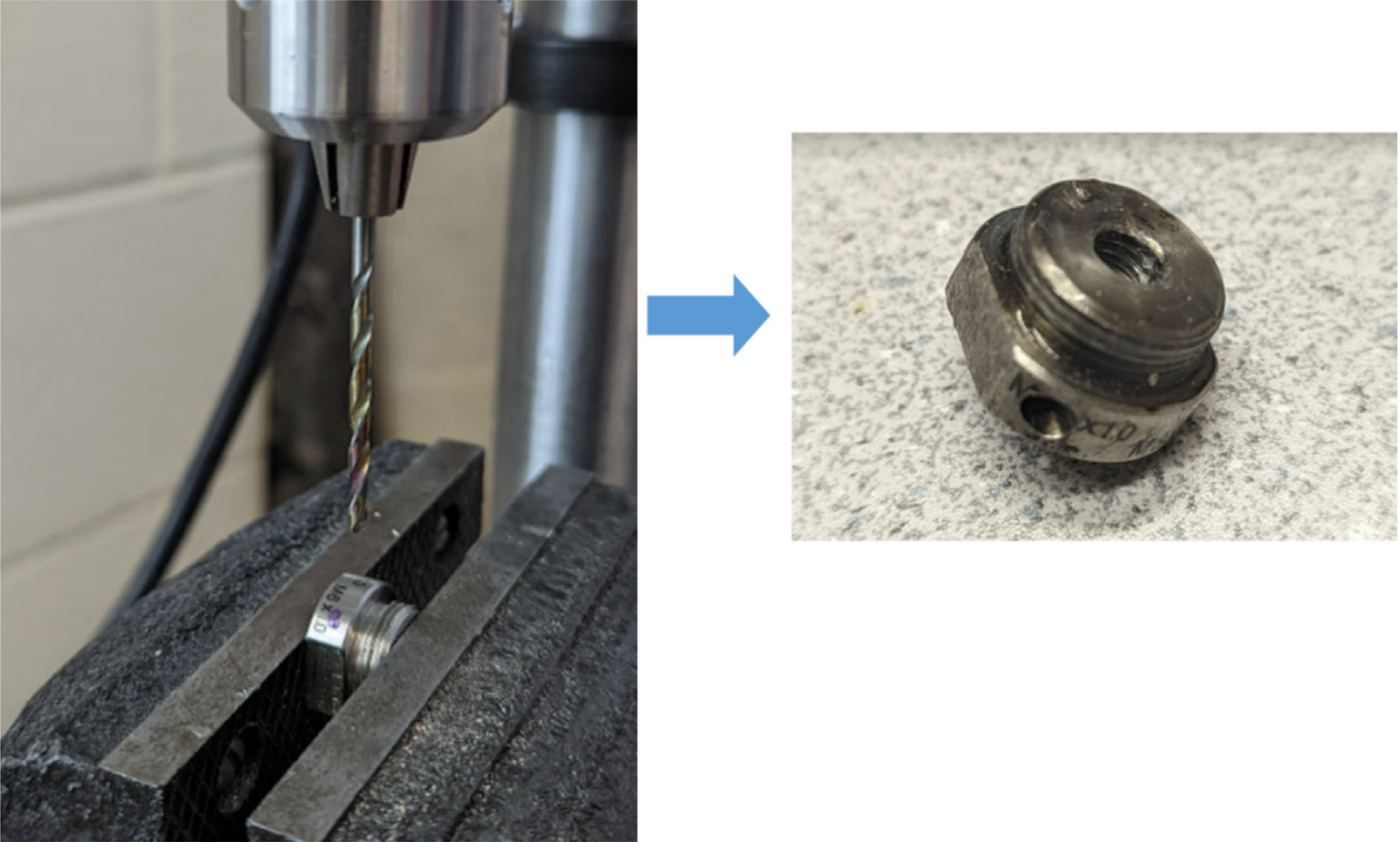
Work holding and drilling 3 mm hole on nozzle adapter.
4. Slowly bring the drill up and down to the hole location ensuring that the drill bit does not bend while drilling into the curved surface.

**Fig. 65:**
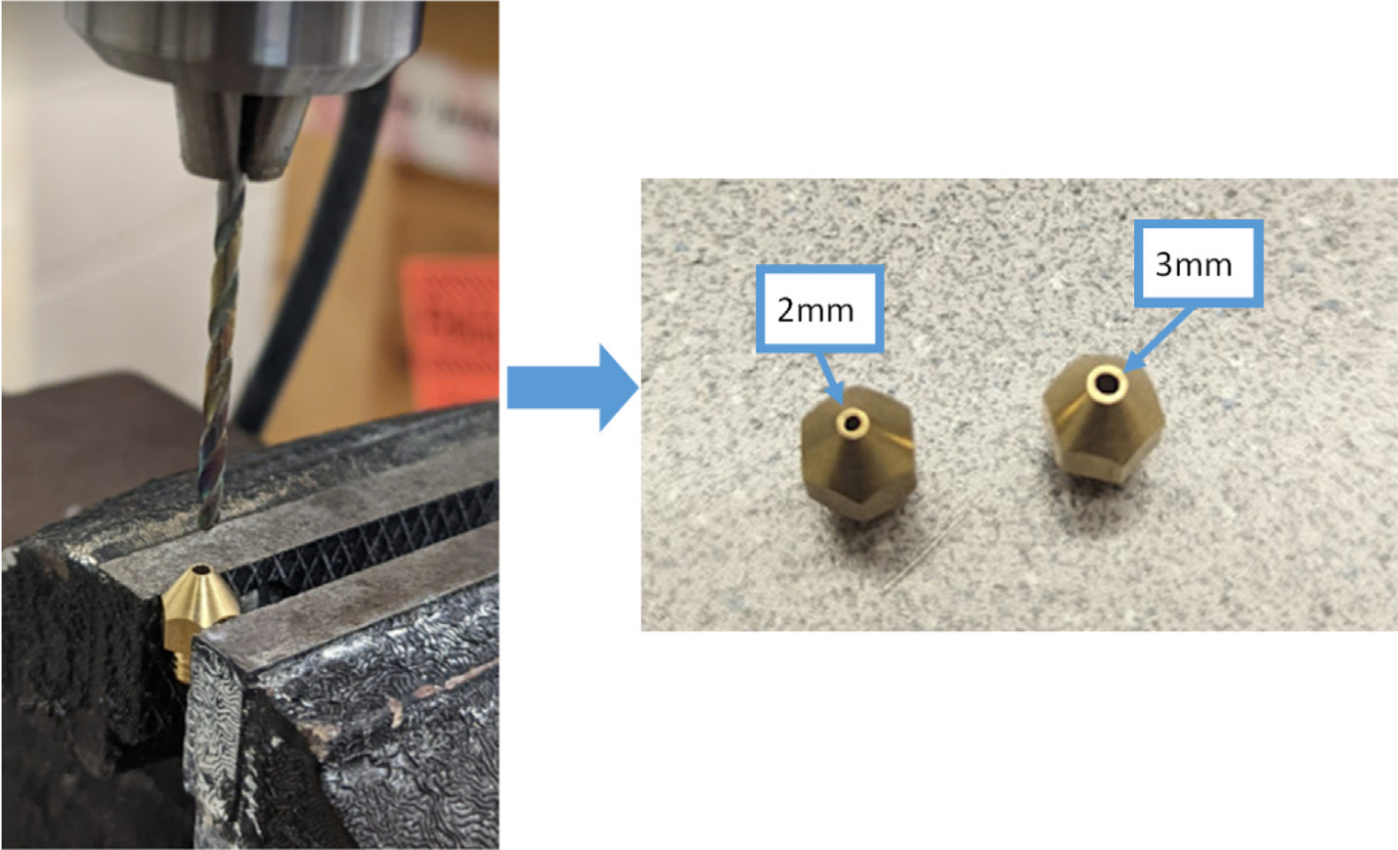
Work holding and drilling 3 mm orifice on 2 mm nozzle.

*Note: Material used here is a copper nozzle however a steel nozzle can also be used and is recommended due to its higher durability and is less prone to fractured threads when swapping nozzles.*

Nozzle:

1. Perform the same steps above with the same drill bit but drill through the existing hole to widen orifice. Ensure that the orifice diameter is close to 3 mm as shown in Fig. 65.

#### Barrel assembly

**Fig. 66:**
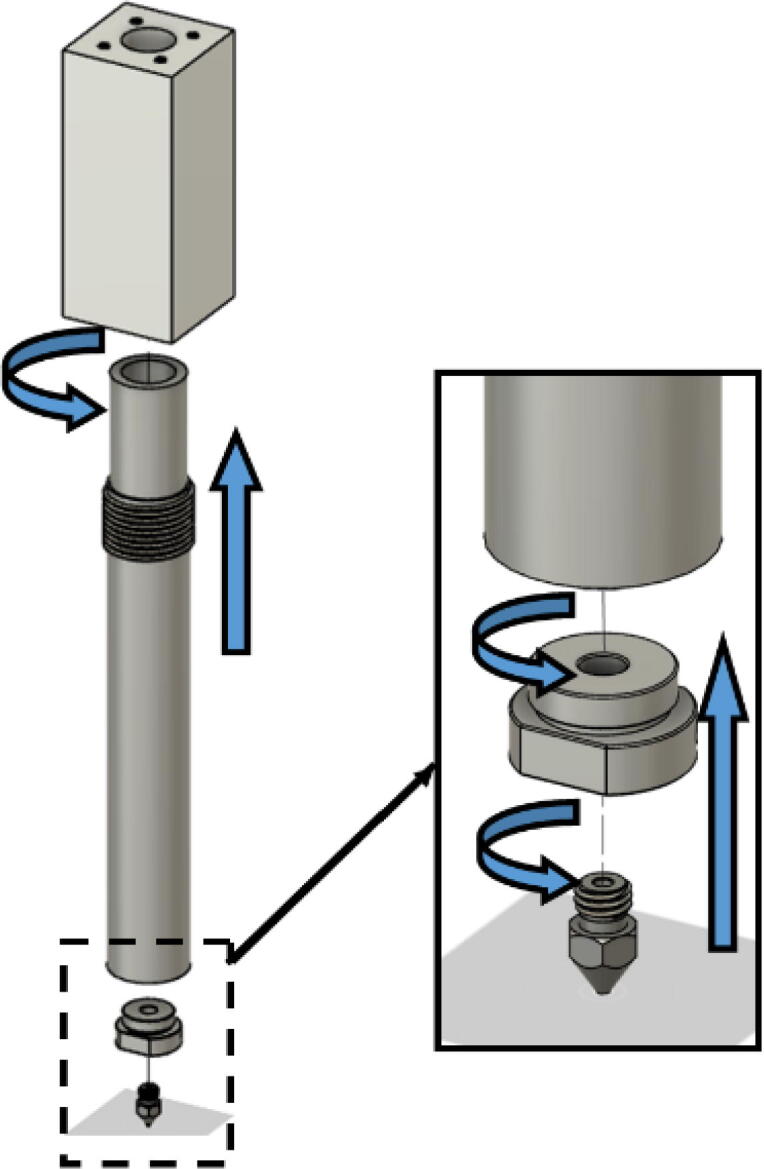
Barrel Metal Assembly.

With modularity in mind, the aluminum heat sink block and steel barrel were designed to be easily screwed and unscrewed togethr for easy cleaning and storage. With the barrel and heatsink block parts machined, screw in the steel barrel into the aluminum heat sink block as shown in Fig. 66. Then screw in the steel nozzle adapter on the bottom of the steel barrel and screw in a nozzle.

#### Full extruder assembly

**Fig. 67:**
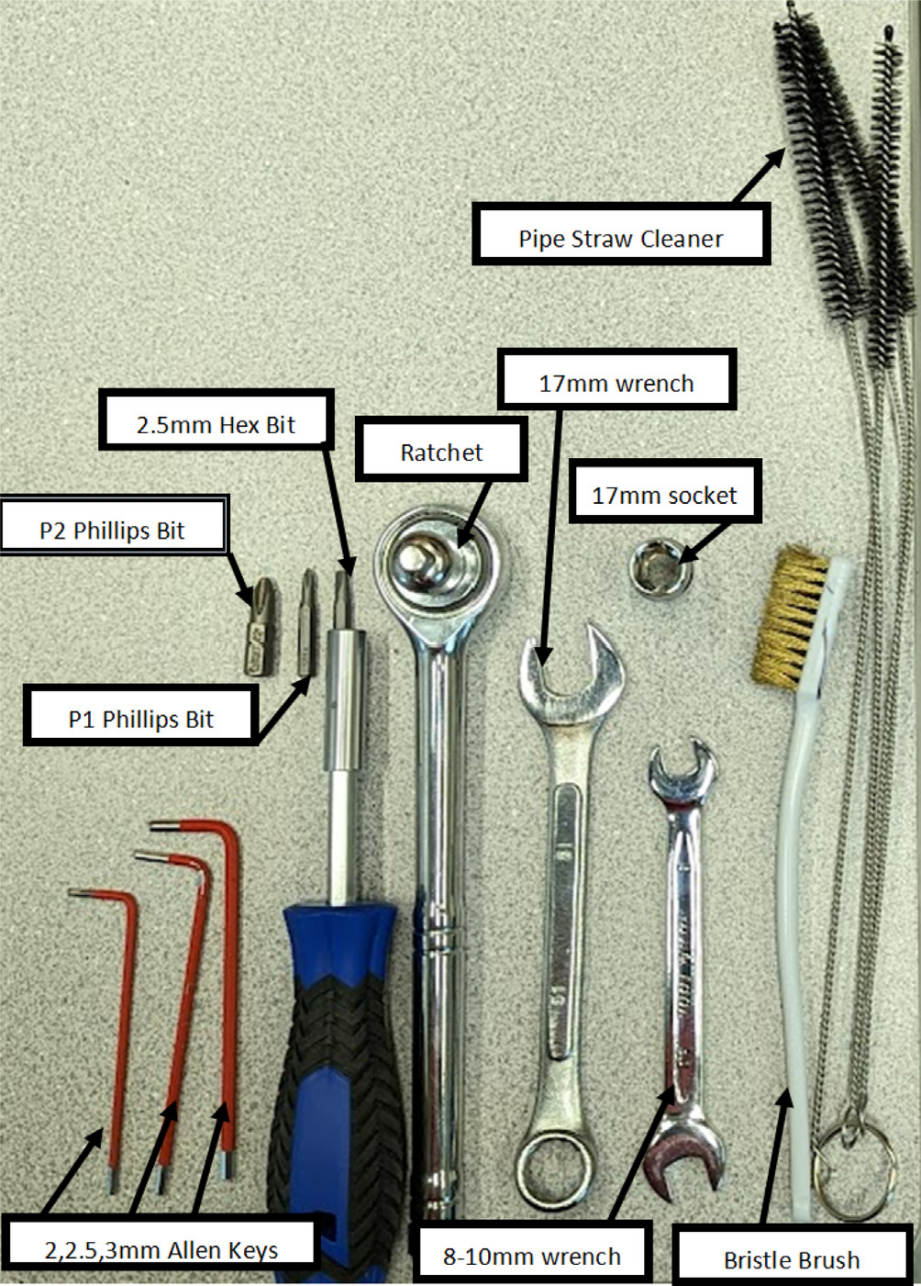
Tools for Extruder Assembly and Cleaning.

The assembly of the extruder consists of a specific order of operations due to the nature of the assembly and space/shape requirements. The tools required for this section are shown below in Fig. 67. Print all corresponding STL file parts listed below according to Table 4 and follow the guidelines set out in [59] or [60], [61] for ensuring good print quality. Ensure that the “Barrel Bracket Base.stl” part is printed out of a strong material that can handle temperatures up to 60 °C near the aluminum heatsink block (i.e., ABS or PETG).

Parts required:

• (1) “Motor Mount Square Bracket.stl”
• (1) “IntakeTube.stl”
• (1) “IntakeTube_Hopper.stl”
• (1) “IntakeTube_Extension.stl”
• (1) “Barrel Bracket Base.stl”
• (1) “Barrel Bracket Wing 1.stl”
• (1) “Barrel Bracket Wing 2.stl”
• (2) “Blower Fan Plate.stl”
• (2) “Blower fan mount.stl”
• (1) Assembled metal barrel assembly from section 5.2.1
• (1) 5/8″ x 18″ HCS Auger Bit
• (1) M6 Copper 2 mm Nozzle
• (6) Heat sinks
• (1) 1/8″x24″x48″ hardboard handy panel
• From M2 M3 M4 Phillips Pan Head Screws Kit:
  o (4) M3x16mm
  o (2) M3x12mm
  o (6)M3x8mm
  o (18)M4x20mm
• (4) 5/16″-18 × 1″hex drive bolts
• (4) 5/16″-18 × 1″ hex coupling nut
• (4) 5/16″-18 Oval head Screws
• (4) 5/16″-18 hex nuts
• Thermal tape
• (2) RTD Pt1000
• (1) 120 V, 900F,1″OD TEMPCO BAND HEATER
• (2) 6015 blower fan
• (2) 5015 axial fan
• Nema 17 Geared 51:1 Stepper Motor

**Fig. 68:**
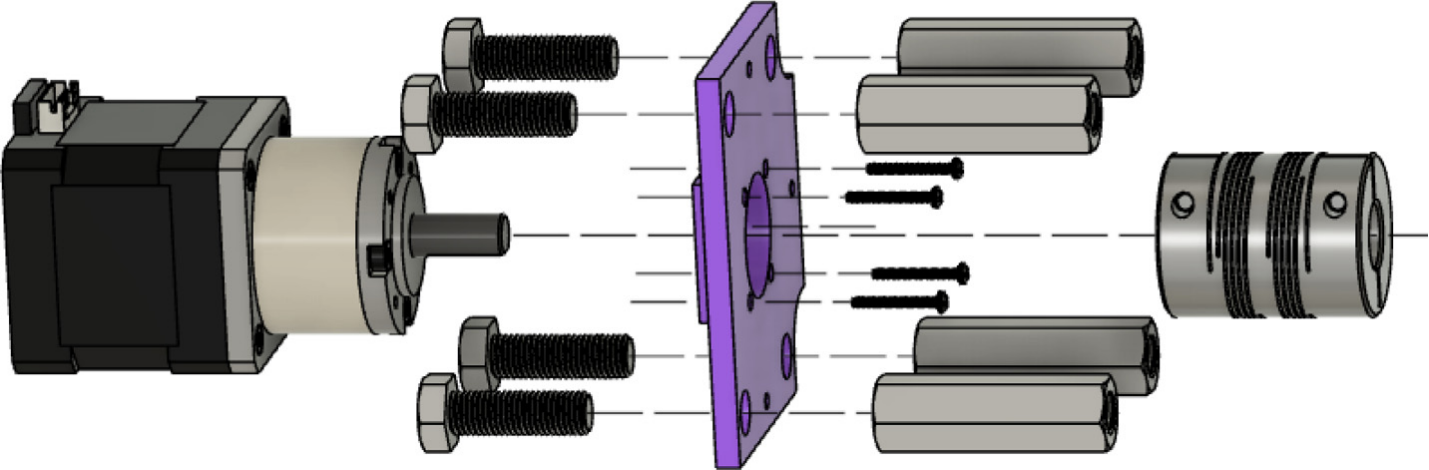
Assembling motor bracket assembly of extruder.

**Fig. 69:**
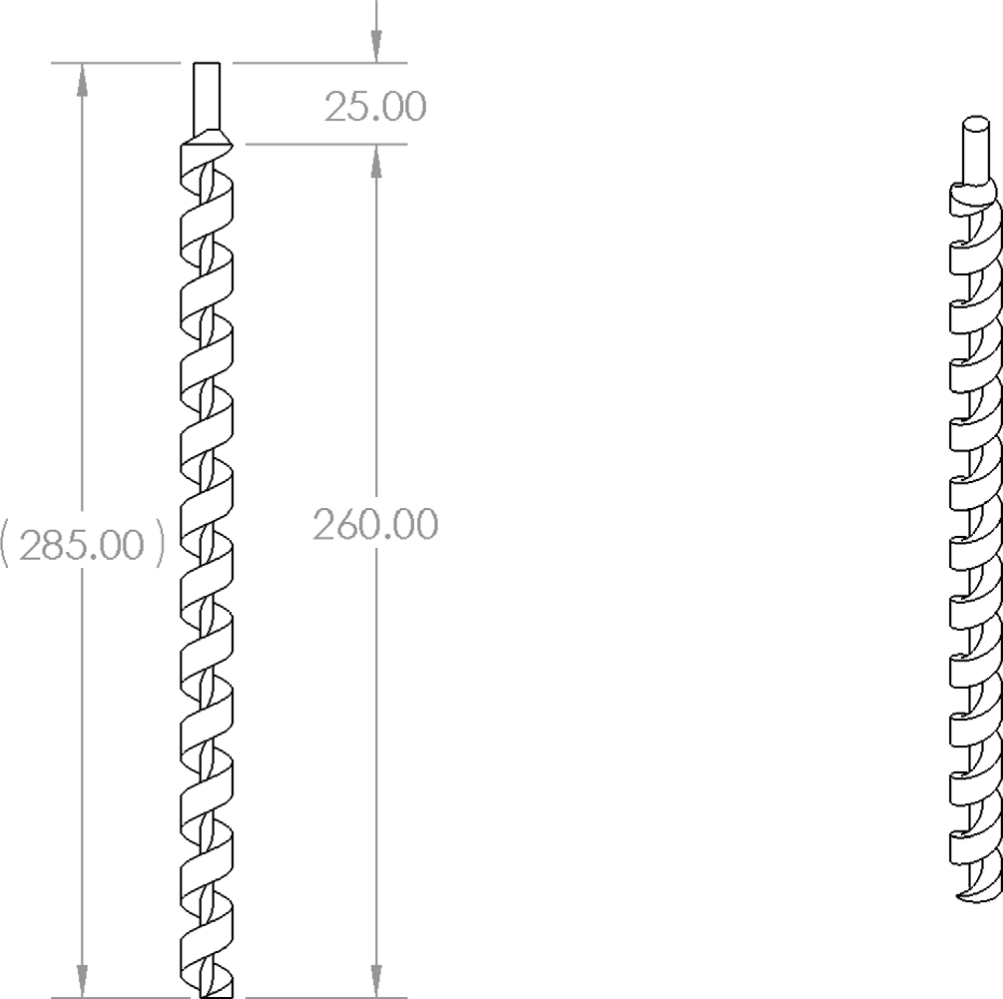
Auger dimensions after cutting.

**Fig. 70:**
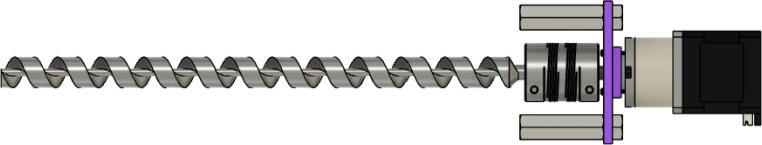
Auger inserted into shaft coupling and motor.

**Fig. 71:**
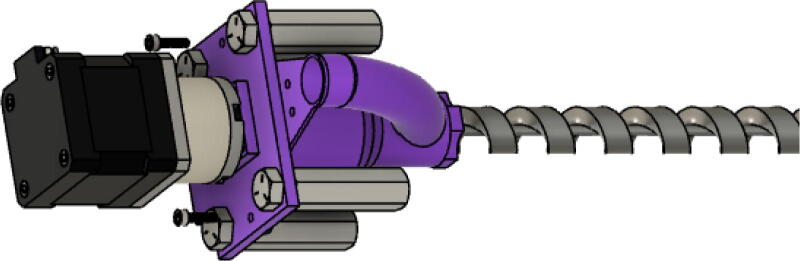
Attaching the IntakeTube.

**Fig. 72:**
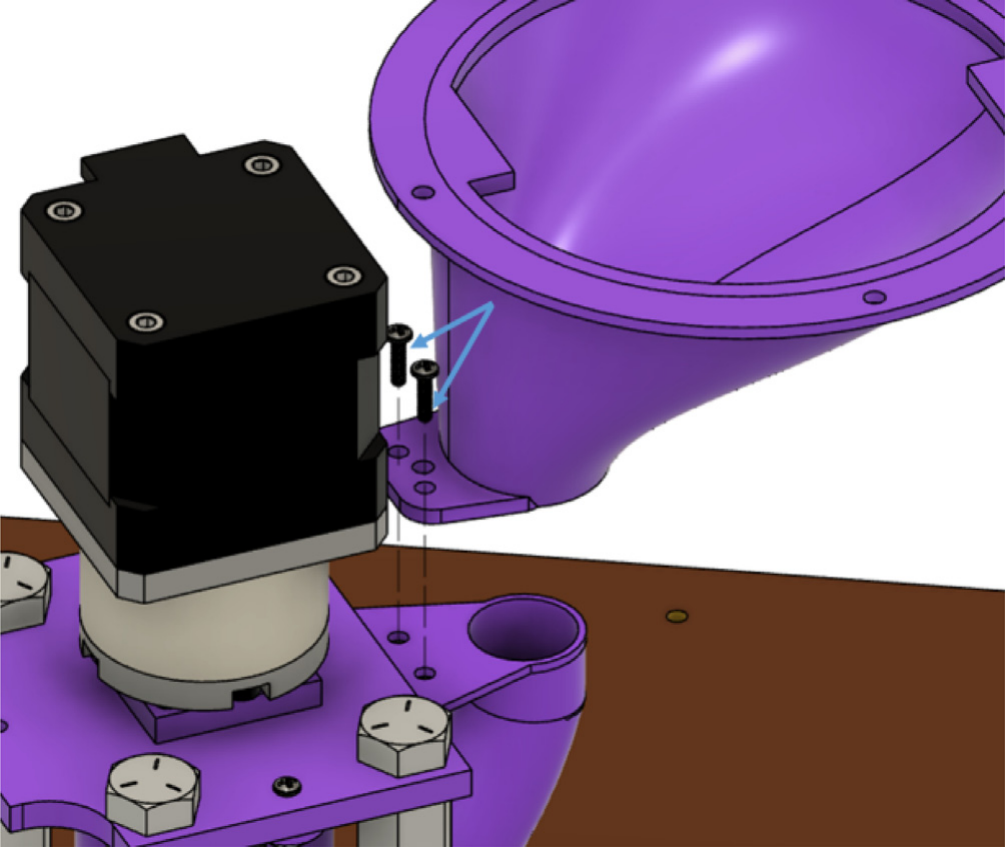
Mounting the IntakeTube_Hopper.

**Fig. 73:**
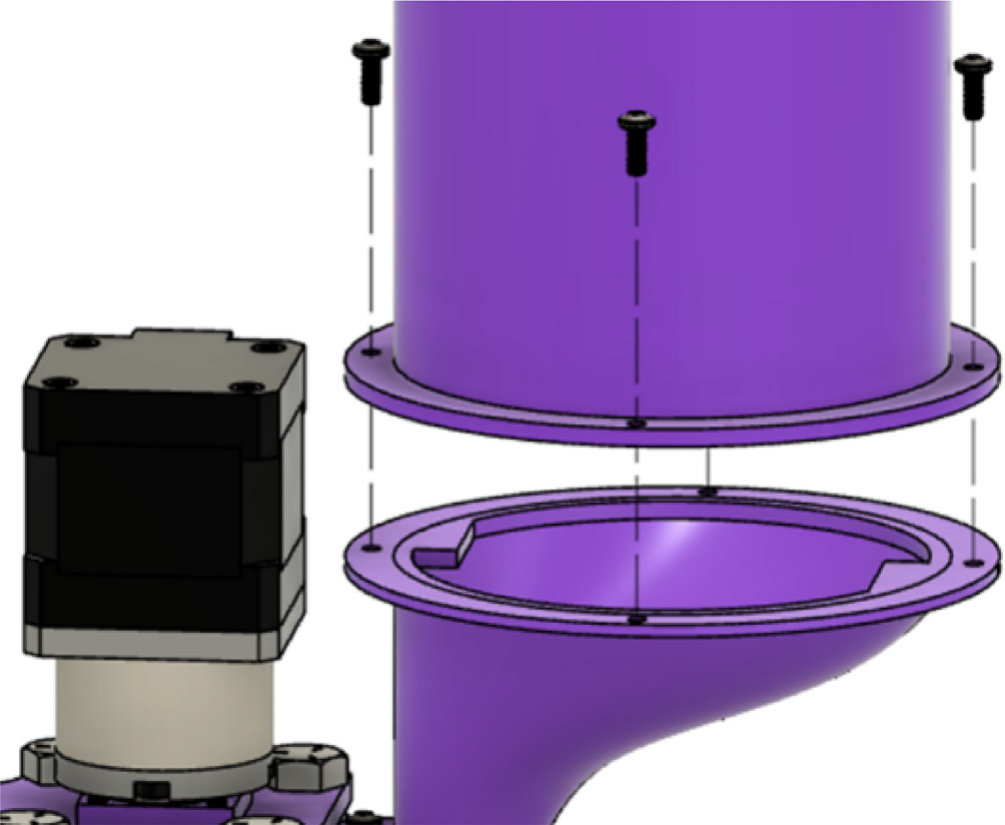
Mounting the IntakeTube_Extension.

Motor and Auger Assembly:

1. Insert the NEMA17 motor through the motor mount bracket as shown in Fig. 68. Use four M3x16mm Phillips screws to secure the “Motor Mount Square Bracket” to the motor with a Phillips bit screwdriver. Next, fit one end of the shaft couple to the shaft motor and tighten its setscrews with a M2 and M3 Allen key.

2. Place the four 5/16in-18. × 1in. hex bolts into the corners of the end of the “Motor Mount Square Bracket” from the top. On the other side, secure each hex bolt with a 5/16in-18 × 1in. hex coupling nut as shown in Fig. 68 using a 8–10 mm wrench*. Note: Do not tighten these hex shaft coupling.*

3. Using an angle grinder with a cutting disc for steel, cut the auger from its initial length to the dimensions shown Fig. 69*.* Secure the auger in a vise and use safety glasses and protective gloves will cutting.

4. Insert the 8 mm hole side of the shaft coupling into the shaft of the NEMA17 stepper motor. Insert the cut auger into the other end (12 mm) of the shaft coupling as shown in Fig. 70*.* Tighten the set screws with an M2 and M3 allen key.

5. Slide the “IntakeTube” up the auger and to the “Barrel Bracket Base” component. Screw in two M3x12mm screws in the locations shown in Fig. 71*.*

6. Attach the “IntakeTube_Hopper” with two M3x8mm screws as shown in Fig. 72*.*

7. Attach the “IntakeTube_Extension” to the hopper with four M3x8mm screws as shown in Fig. 73. This cylindrical extension serves to maintain a steep slope for the flexible tube for pellets to freely flow from the feeding system to the extruder. Additionally, it ensures that there is no slack on the tube thus preventing any bridging of the tube which would otherwise block the pellets.

Fan Panel Assembly:

8. Slide the both “Blower Fan Plates” into their respective “Barrel Bracket Wing 1” and “Barrel Bracket Wing 2” *T*-slots shown in Fig. 75*.* If there is a tight clearance, use a hammer or mallet and lightly tap on the edge of the part to complete the T slot assembly. Perform additional sanding to mating edges if necessary.

**Fig. 74:**
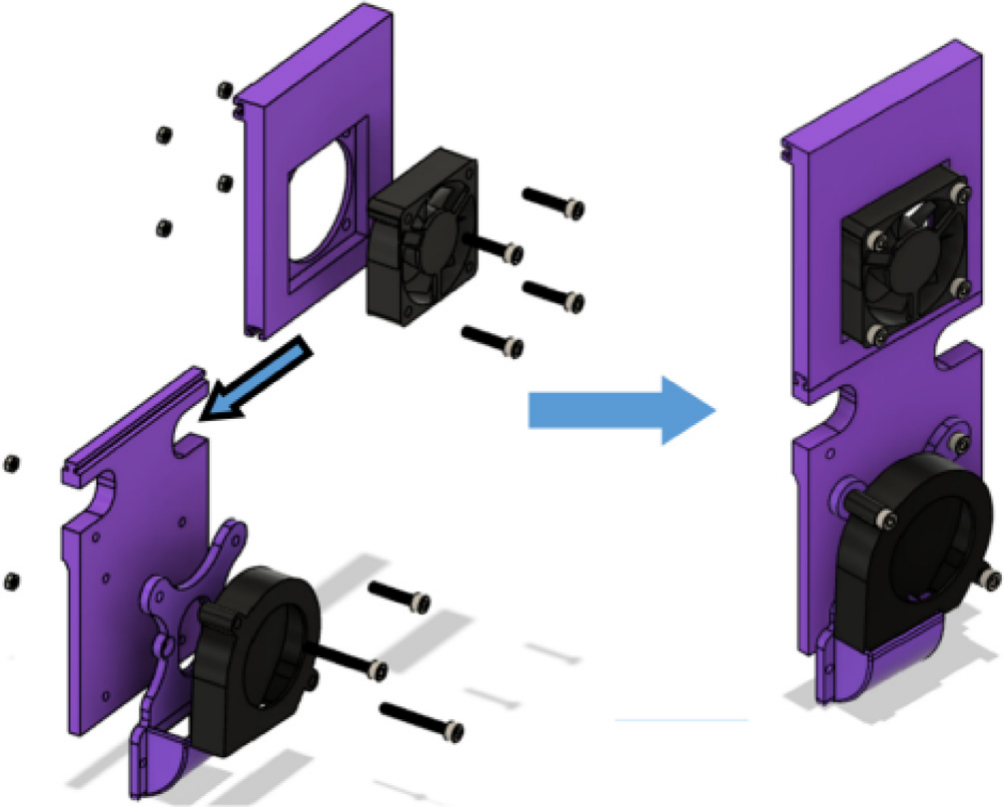
Right fan panel assembly.

**Fig. 75:**
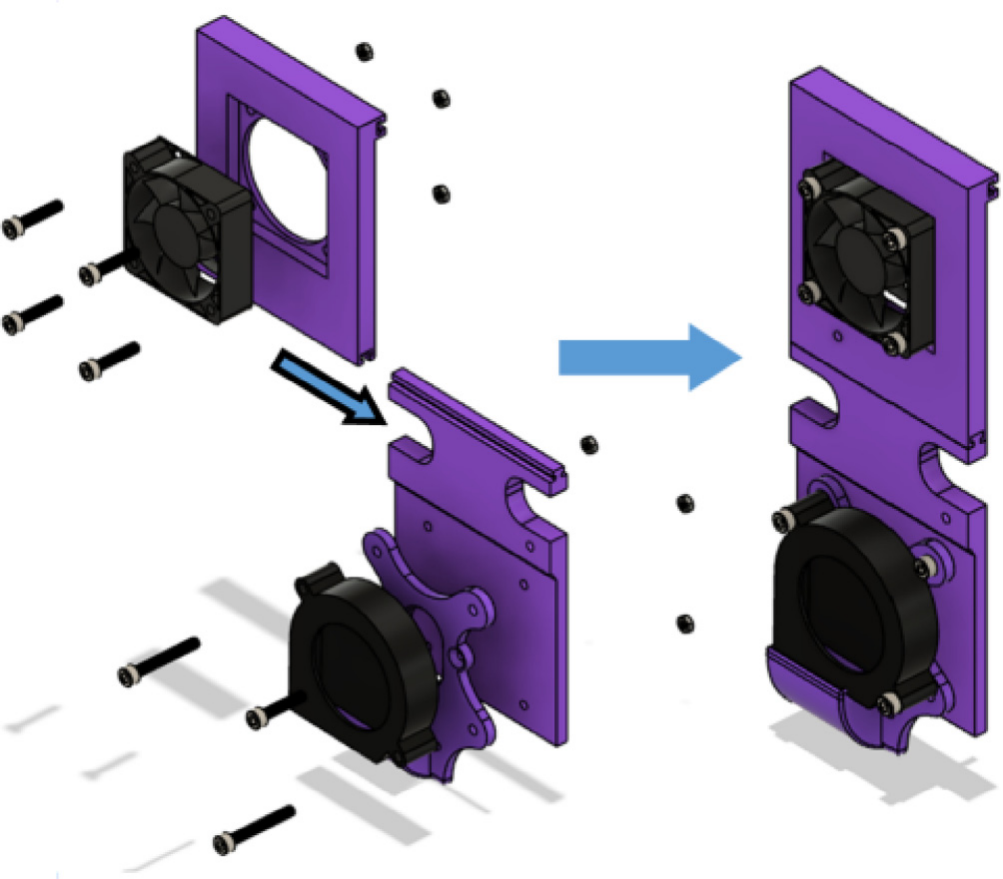
Left Fan Panel Assembly.

Insert the 5015 axial and 6015 blower fans into their corresponding locations shown in. Fig. 74 secure fans using M4x20mm Phillips screws and M4 nuts

**Fig. 76:**
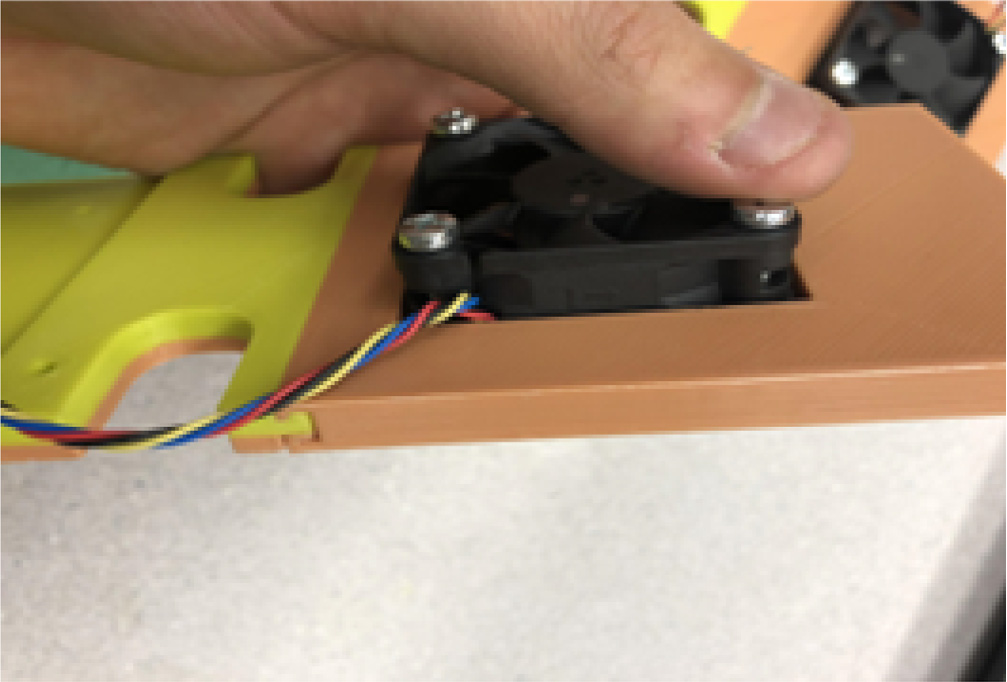
5015 axial fan orientation.
• “Blower fan mount” is attached together with the blower fan.

• Four M4x20mm screws for each 5015 fan and three M4x20mm screws for each 6015 blower fan.
• Ensure that the axial fan is set to drive air inwards towards the aluminum heatsink block (Figs. 56 and 76).

**Fig. 77:**
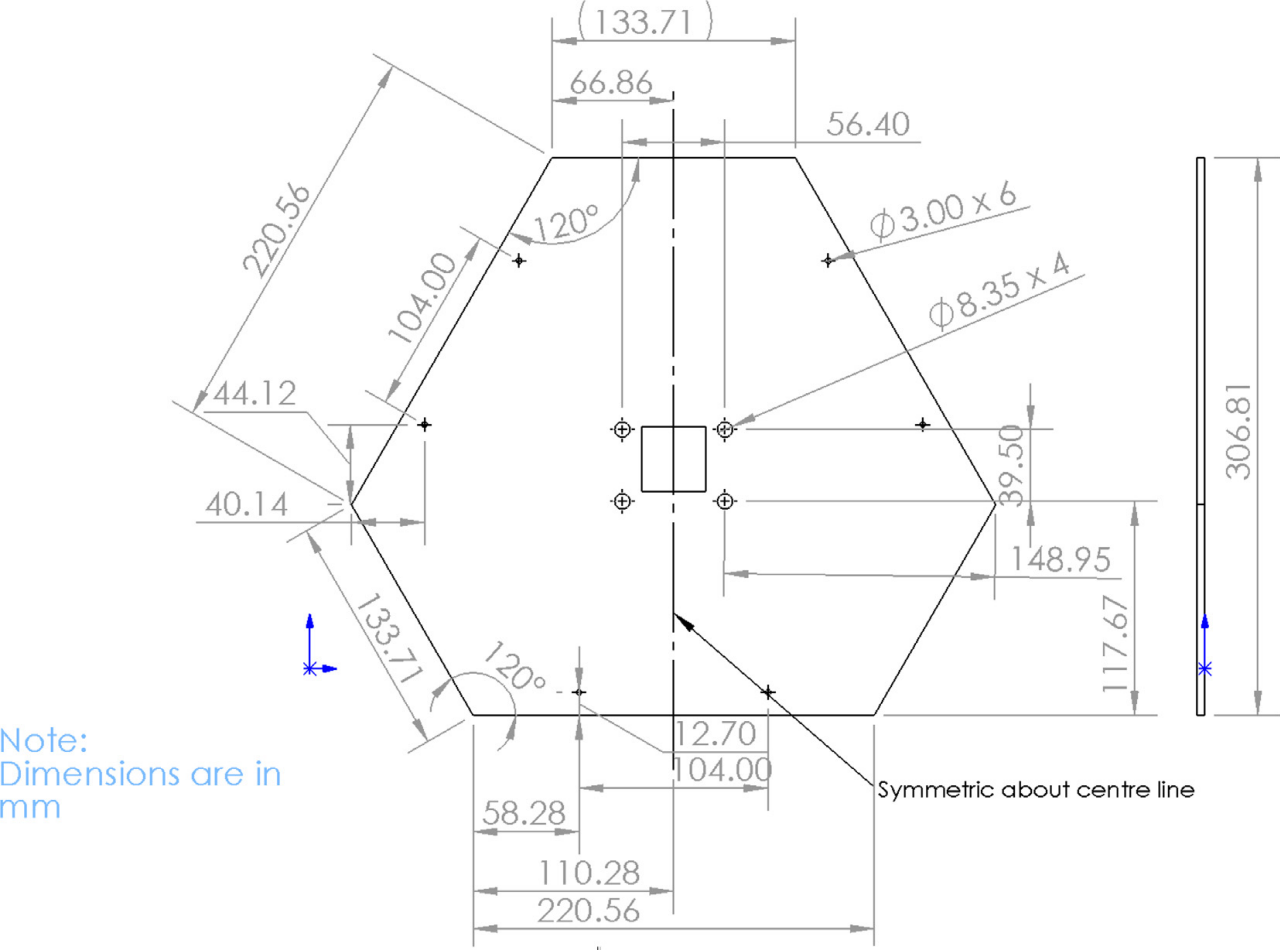
Chassis Frame Mount dimensions.

**Fig. 78:**
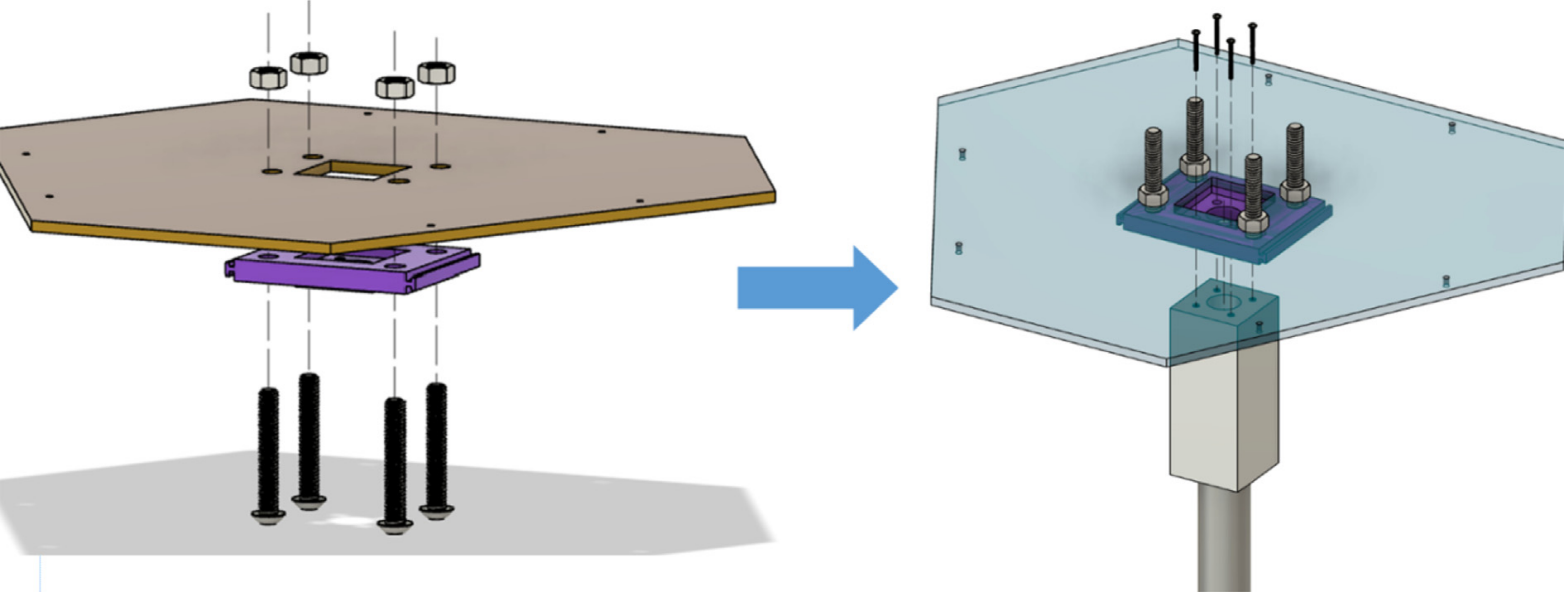
Barrel Bracket and lower extruder assembly.

**Fig. 79:**
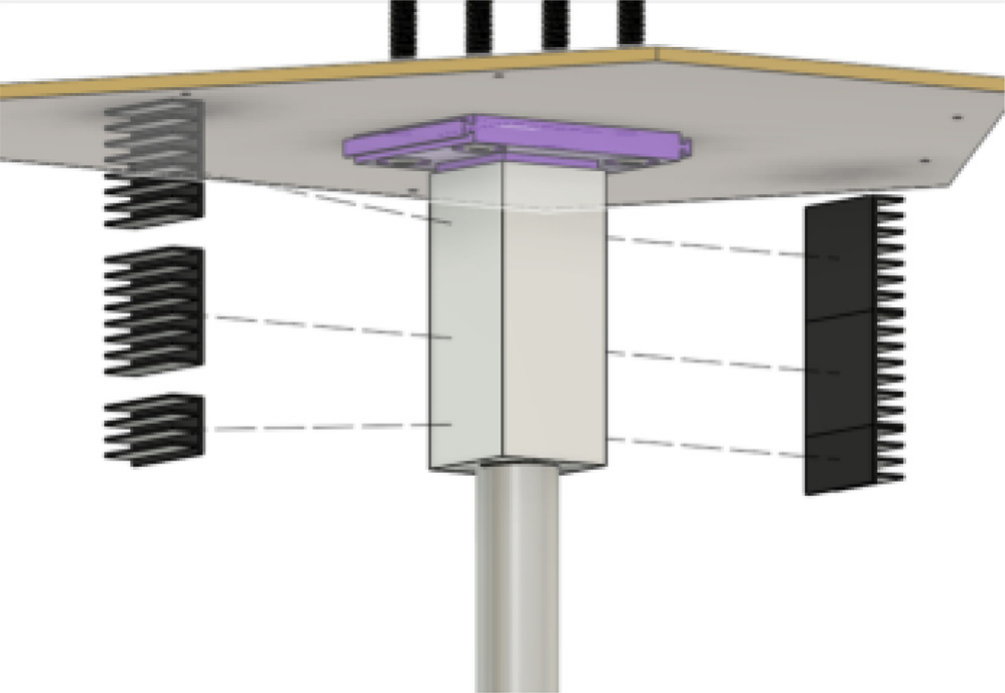
Mounting heatsink array to Aluminum Heatsink Block.

**Fig. 80:**
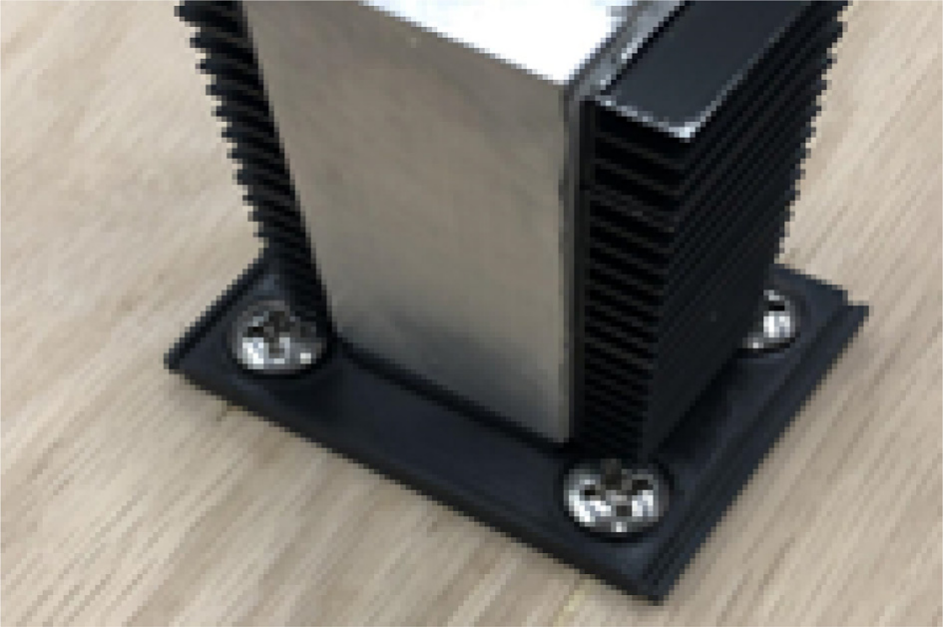
Mounted heatsink array orientation.

Assembling the Barrel and Motor Frame Assembly:

10. Produce the “Chassis Mount Frame” from the 1/8”x24”x48” hardboard handy panel according to the dimensions shown in Fig. 77. Cut using a band saw or table saw. Alternatively, this component can also be laser cut by using the provided 1:1 scale DXF file in [47].

11. Assemble the “Barrel Bracket Base” and “Chassis Mount Frame” using four 5/16“-18 oval head screws and corresponding four 5/16”-18 nuts as shown in Fig. 78.

12. Next, assemble the barrel assembly to the “Barrel Bracket Base” using four M4x20mm Phillips screws.

13. Using the thermal tape, attach the heatsinks to the Aluminum Heatsink Block according to Fig. 79*.* The heatsink array on both sides of the aluminum block consist of two full heatsink parts and one cut to size so that it is flush with the bottom face of the aluminum block.

14. Press the full heat fin array, on either side of the heat sink body. Ensure that each array is set horizontally along the thermal tape to push air out the sides. Align the heat sink with the base of the Barrel Bracket. The result is shown in Fig. 80.

**Fig. 81:**
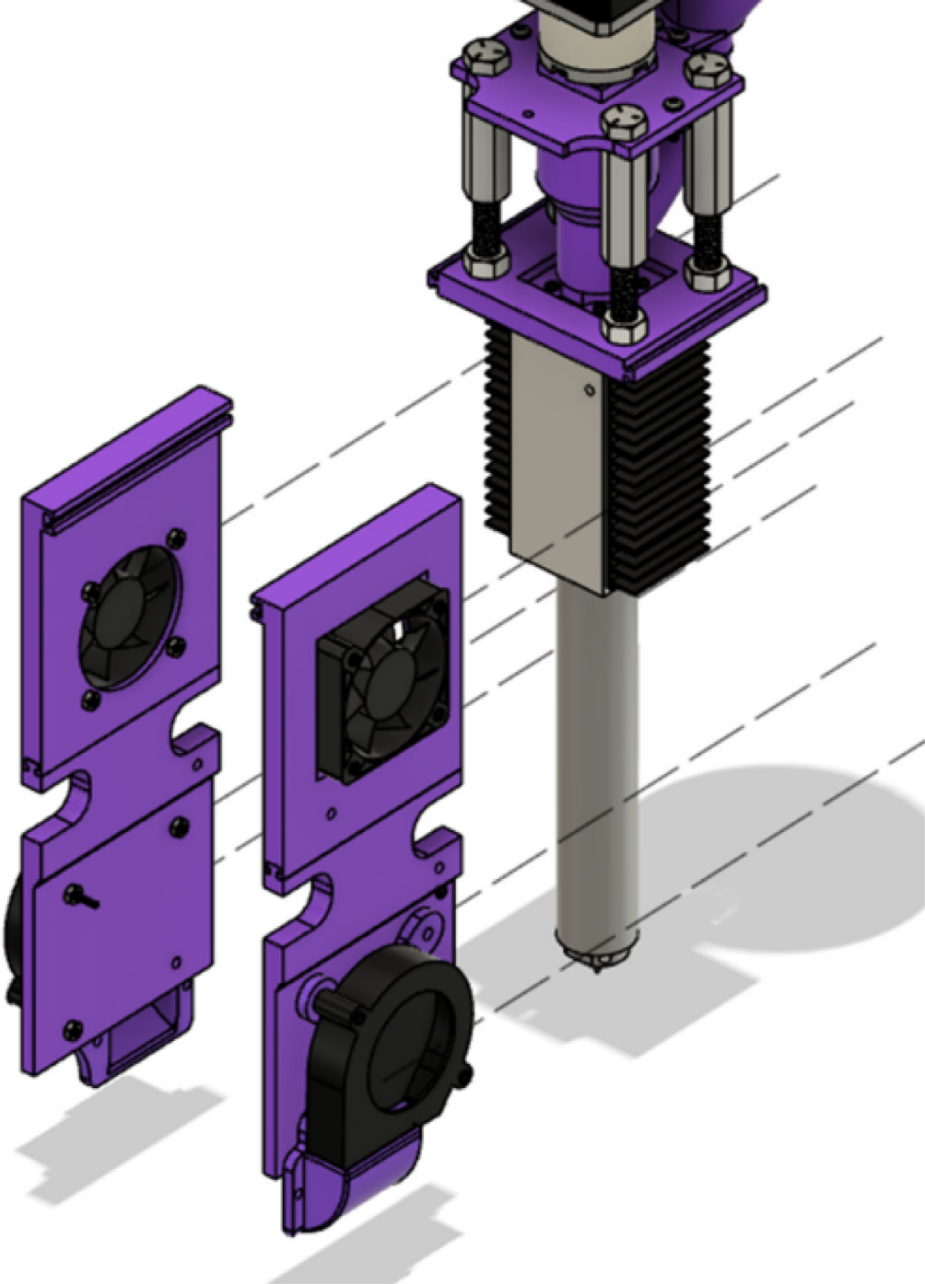
Assembling fan panels to complete extruder assembly.

Slide both assembled fan panels into their respective *T*-slots on the “Barrel Bracket Base” part as shown in Fig. 81. Note: The “Chassis Mount Frame” component is omitted for clarity.

**Fig. 82:**
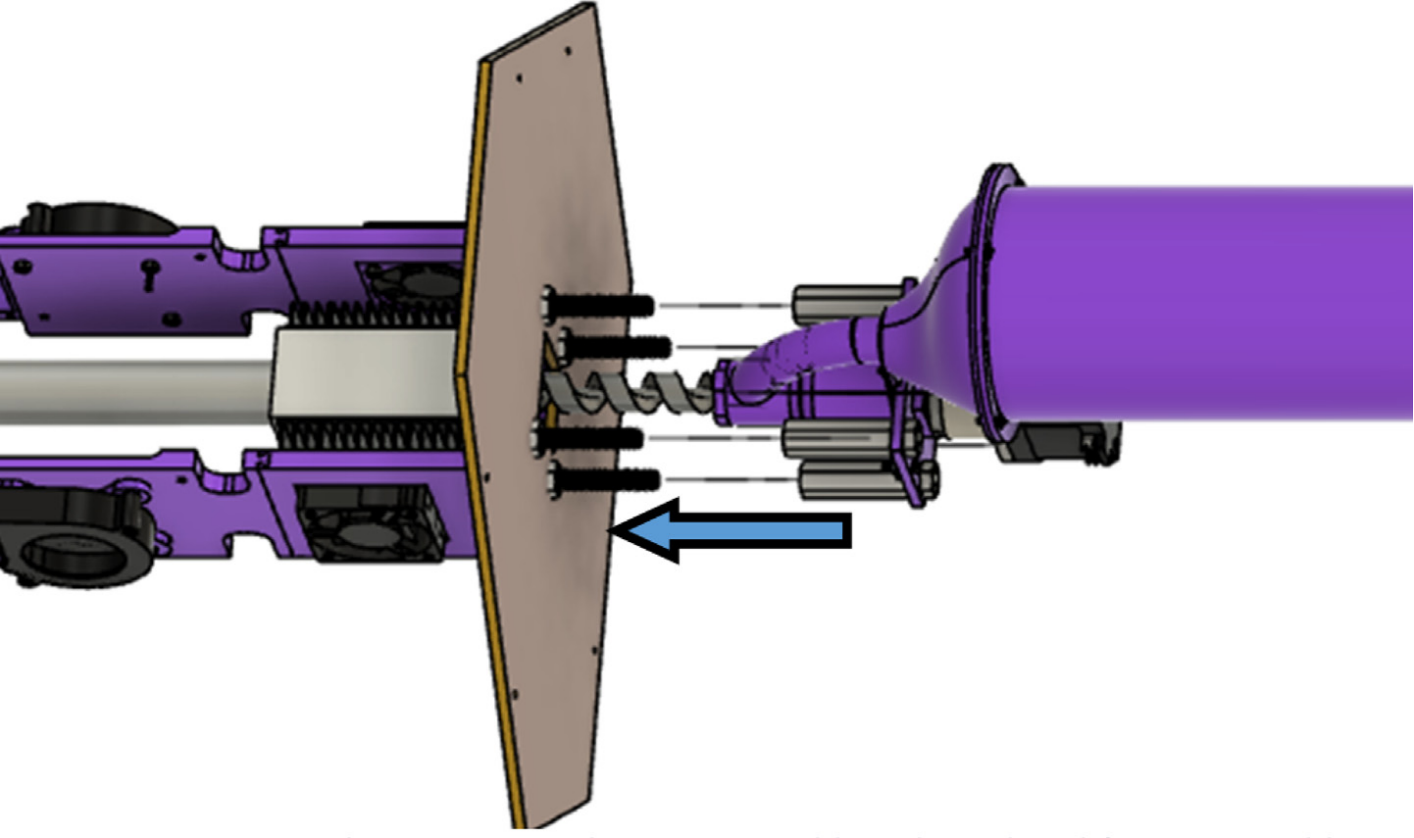
Attaching motor and auger assembly to barrel and frame assembly.

**Fig. 83:**
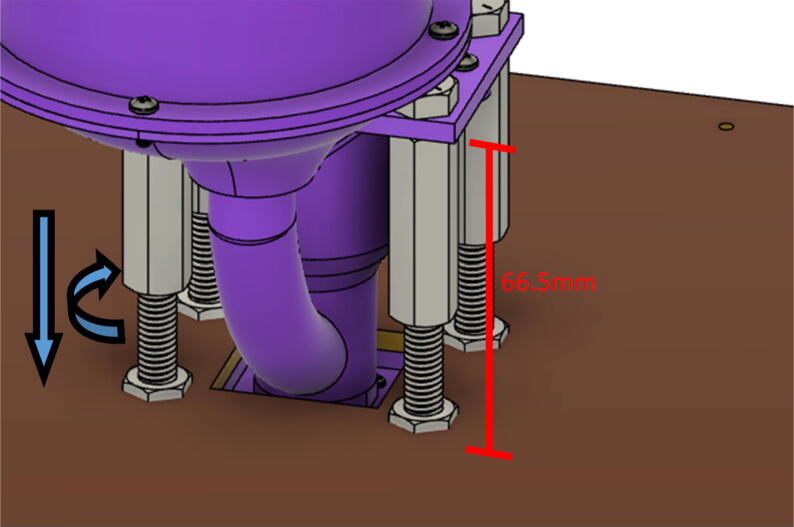
Adjusting motor bracket and chassis mount clearance.

16. Mount the standoffs from the motor and auger assembly to the bolts on the chassis frame as shown in Fig. 82. Rotate and adjust each standoff so that the distance between the top face of the “Chassis Mount Frame” and bottom face of “Motor Mount Square Bracket” is 66.5 mm +/- 0.1 mm as shown in Fig. 83. Measure with calipers.

17. This distance is to ensure that the bottom of the intake tube forms a closed seal on the barrel bracket so that no interference is created when the auger is conveying pellets down the extruder.

18. Tighten the hex bolts to secure the “Motor Mount Bracket” to the rest of the assembly.

**Fig. 84:**
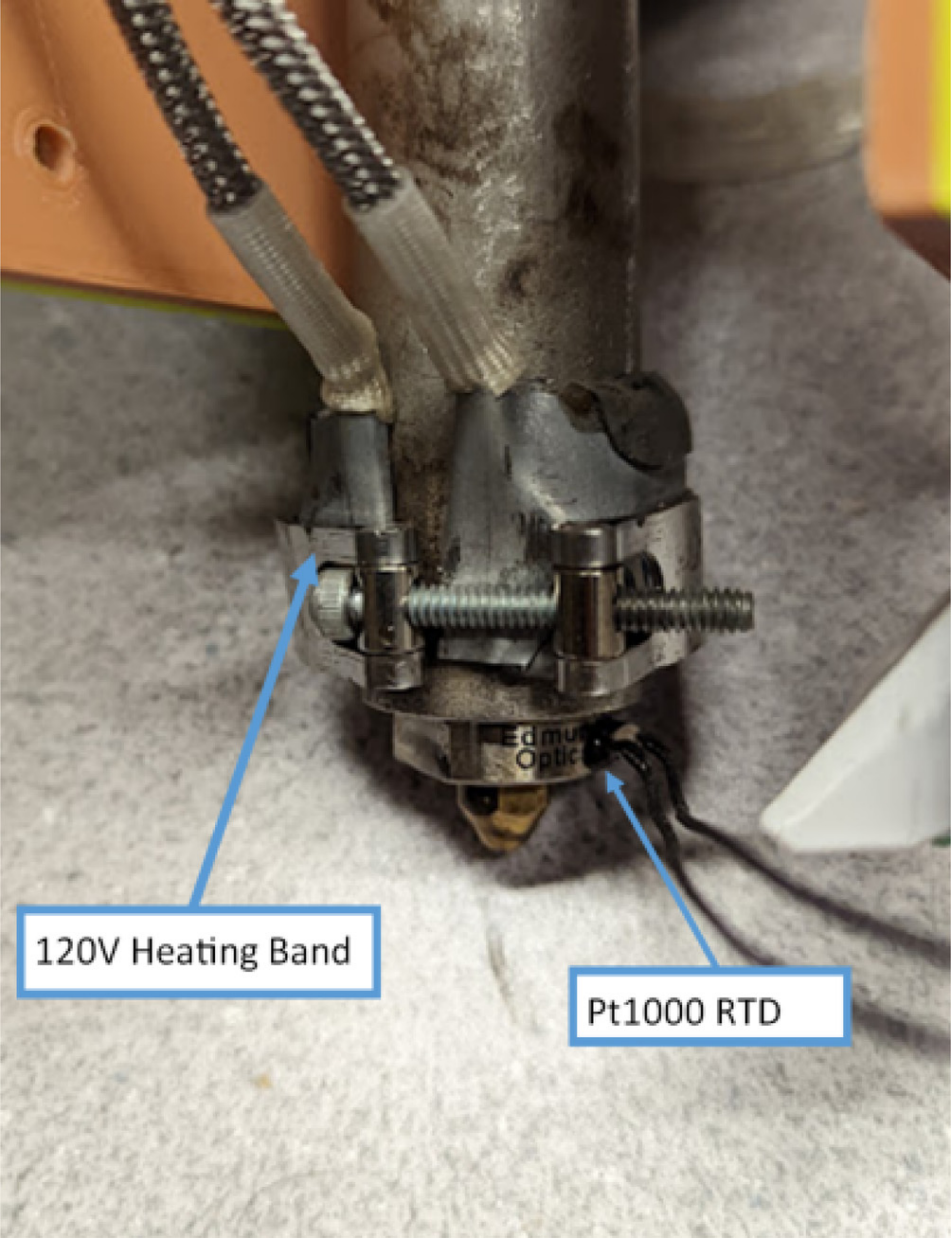
Heating band and RTD sensor attachment.

Slide the 120 V band heater to the “Steel_barrel” as shown in Fig. 84. Insert a pt1000 RTD in the hole drilled into the nozzle adapter.

**Fig. 85:**
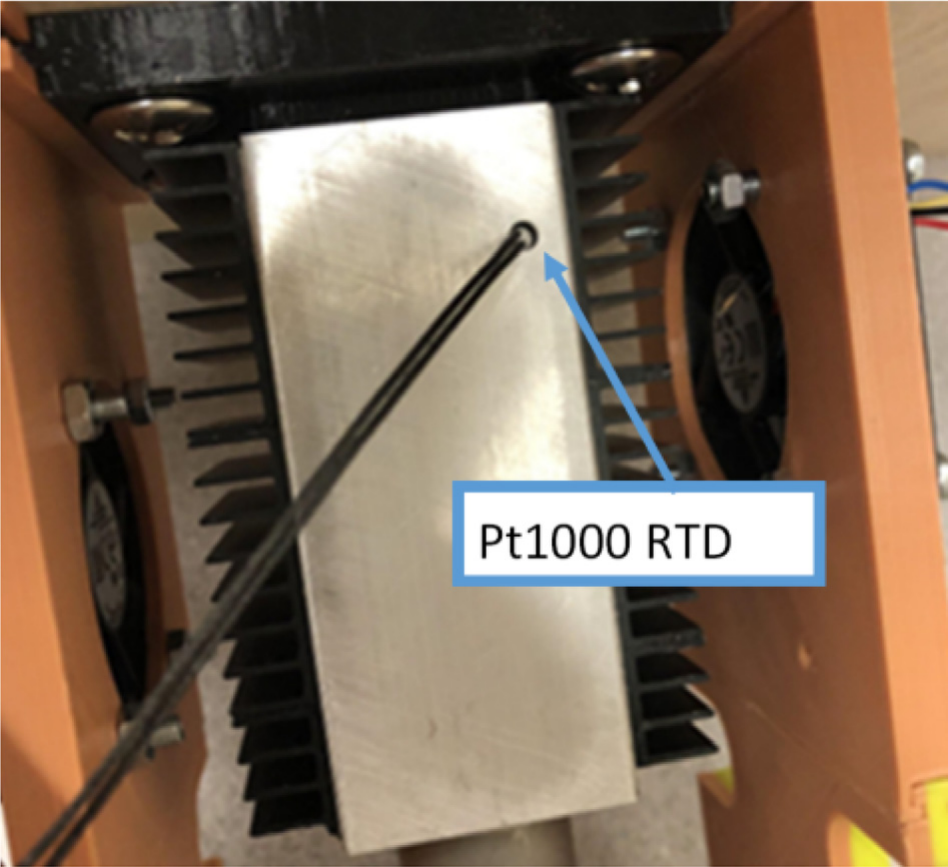
Optional RTD mount for monitoring upper barrel temperature.

20. Insert another Pt1000 sensor in the hole location on the aluminum heatsink block according to Fig. 85. Note: Inserting the sensor here is optional and only for monitoring/debugging purposes as explained in the sections below.

**Fig. 86:**
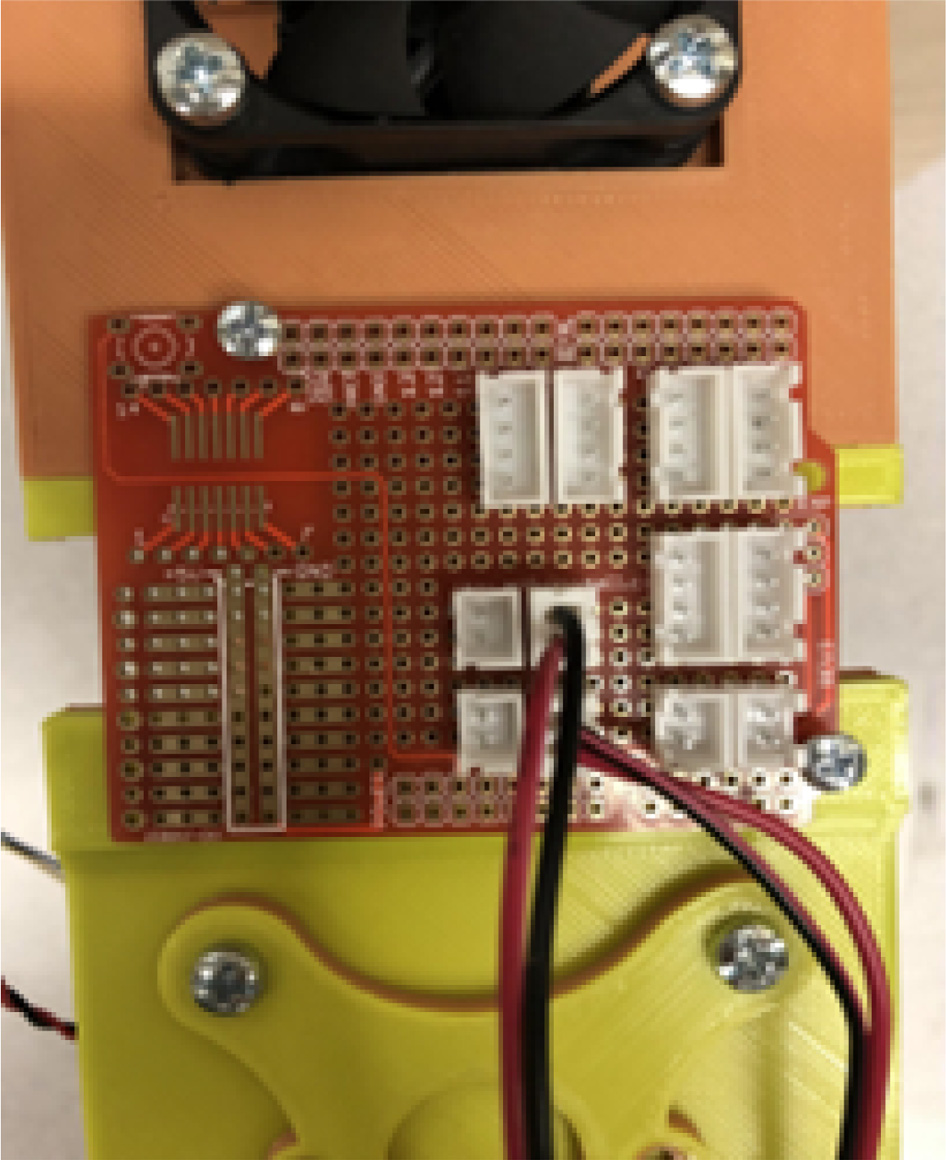
Breakout board mounting.

Position the holes of the breakout board between the holes of either fan panel assembly and fasten the board using two M3x16mm screws and M3 nuts as shown in Fig. 86.

**Fig. 87:**
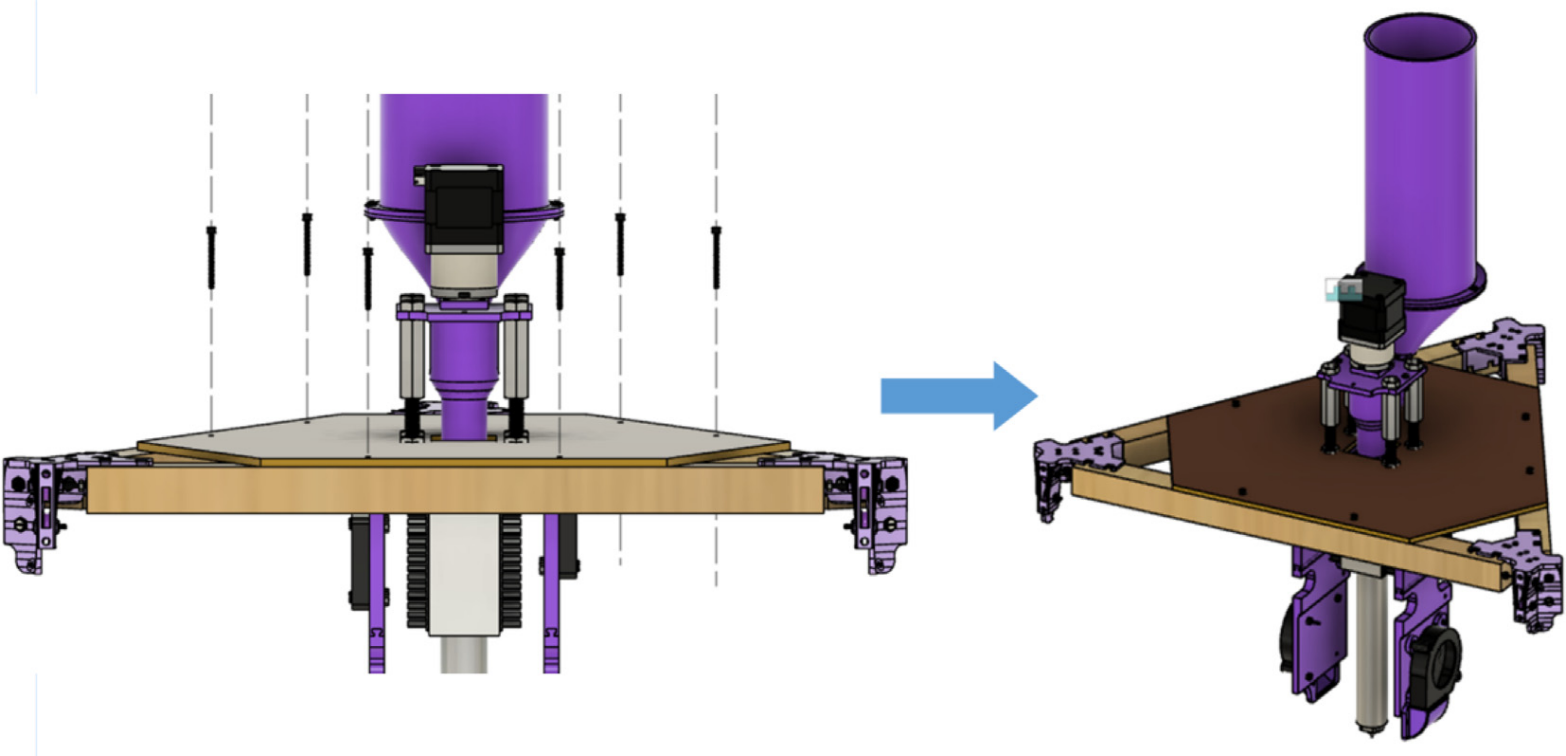
Assembling extruder to Hangprinter chassis.

This completes the mechanical assembly of the pellet extruder. Assemble the extruder onto the Hangprinter by drilling the “Chassis Frame” with 6 M3x30mm hex cap screws onto the Hangprinter chassis as shown in Fig. 87 below. Note: It is recommended to mount the extruder on the chassis after the necessary calibration steps have been performed in the sections below.

### Pellet feeding system

The pellet feeding system stores a large quantity of pellets and releases them to the extruder at a controlled rate via a flexible tube. The hopper is mounted on the ceiling mount and can slide in and out of place via aluminum extrusions and screws sliding the T slots. All the components of this system can be assembled using common hand and power tools and 3-D printing methods.

#### Hopper and feed mechanism assembly

Parts used:

• (1) “Feeding Tube Coupler.stl”
• (1) “Feeding gear.stl”
• (1) “Feeder Outlet.stl”
• (1) “Feeder Motor Enclosure.stl”
• (1) “Feed Gear Enclosure.stl”
• (8) “Hopper Corner Bracket.stl”
• (1) 4′x8′x1/8′ Utility Panel
• (1) 2″by4″by8′ SPF Dimensional Lumber
• (1) 4x4 square wood beam
• (1) 11/16-in × 3–1/2-in × 8-ft MDF/Particle Board
• (1) 20mmx20mmx914mm aluminum extrusion
• (1) 12″x12″x1/8″ Acrylic sheet
• (1) PEX 3/4″x10′ waterline pipe
• (5) 2″x2″x1.89″ L bracket
• (4) 2″x5/8″ l-corner brace
• (14) 2″ x 5/8″ Mending Platel -Zin
• (1) 8 mm to 8 mm Shaft Coupler
• (1) 8 mm steel shaft
• (1) 3 in. × 20 ft. Vinyl Flexible Hose
• (32) #6 1/2″ flat head wood screw
• (16) #5x3/4 Flat Head Wood Screw
• From M2 M3 M4 Phillips Pan Head Screws Kit:
  o (34) M4x16mm
  o (34) M4 nuts
• Nema 17 Stepper Motor, 5:1 Gearbox
1. Print the STL file parts above according to Table 4 and use the part inspection process laid out in [59] and or [60], [61].
2. Cut 10 pieces of the 4′x8′x1/8′ utility panel in the quantities and shapes below. Detailed drawings of these pieces can be found in the schematics and drawings folder of [47].
  a. Two 390 mm*250 mm rectangles (Rectangle 1).
  b. Two 240 mm*250 mm rectangles (Rectangle 2).
  c. Two at 230 mm*240 mm rectangles (Rectangle 3).
  d. Two at 50 mm*180 mm rectangles (Rectangle 4).
  e. Two symmetrical trapezoids (top is 40 mm, bottom is 390, and the distance from top to bottom is 150 mm).
3. With the two “Rectangle 1” and two “Rectangle 2” pieces, assemble a box frame as shown in Fig. 88. Glue four printed triangular “Hopper Corner Bracket” pieces with one on each corner. Leave the glue to dry for 1–2 h.

**Fig. 88:**
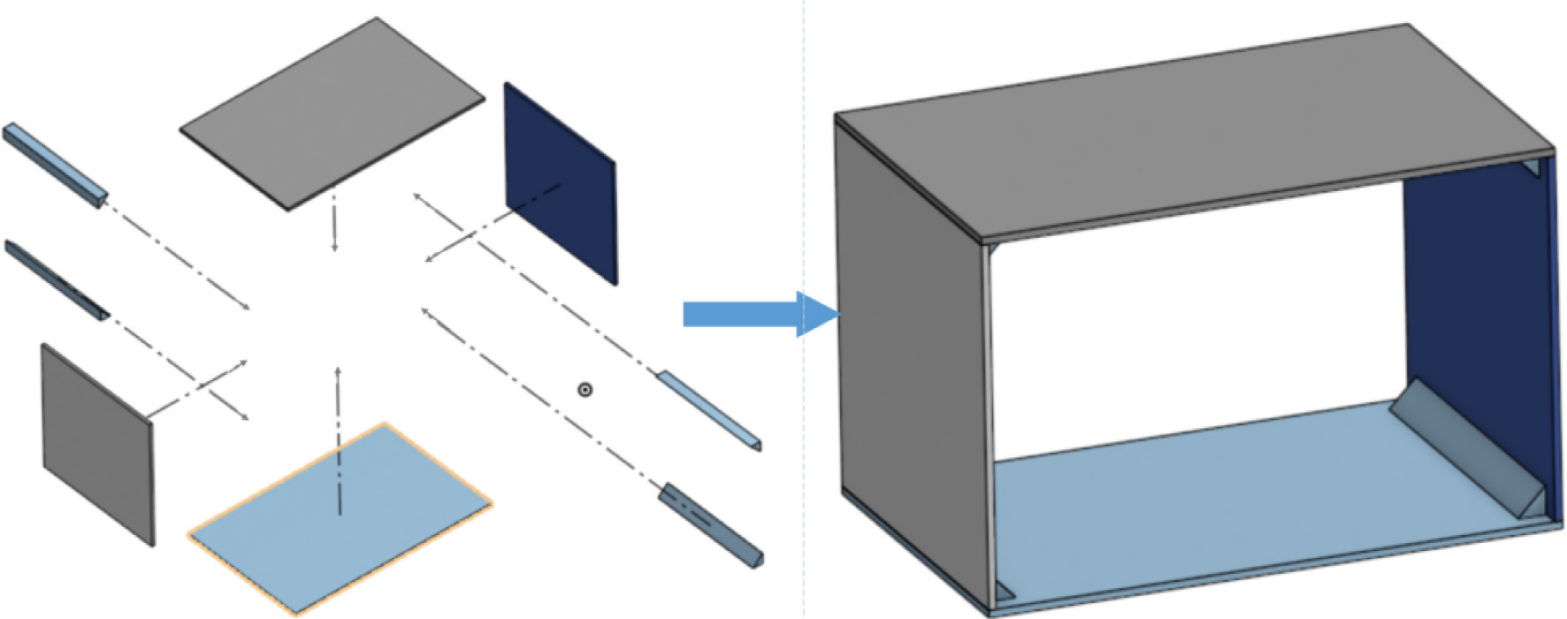
Upper hopper assembly.
4. Once the glue has dried, use 16(4 for each corner) #6 1/2″ flat head wood screws to fasten and secure each of the corners of the box as shown in Fig. 89. Exact location is not important but ensure screws are spread evenly and screw into the triangular “Hopper Corner Bracket” part on the inside corner of the upper hopper assembly.

**Fig. 89:**
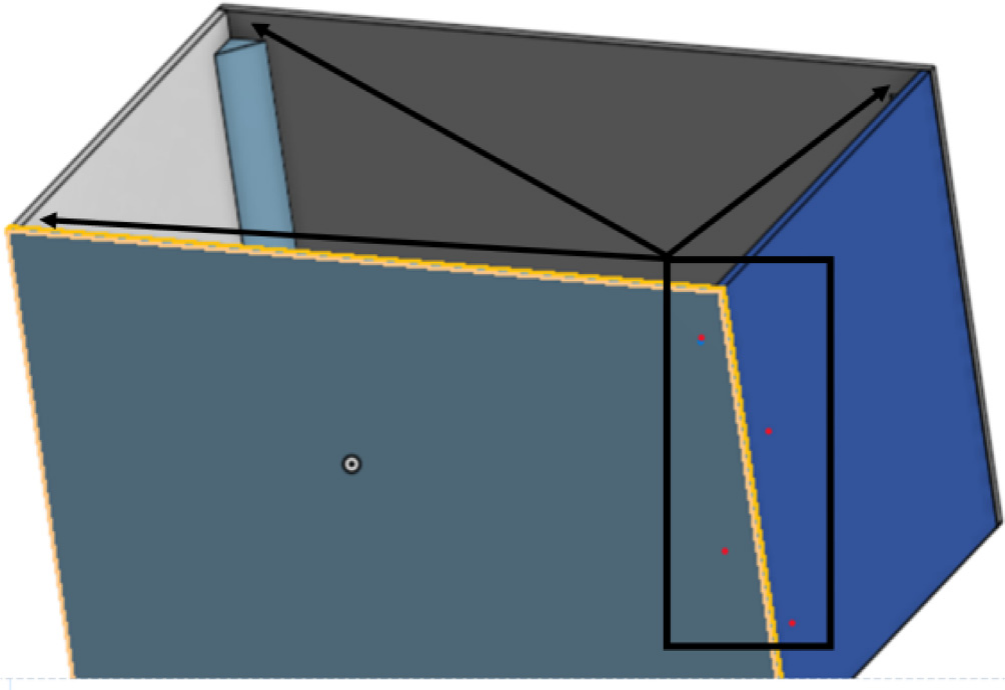
Wood screw locations for upper hopper assembly.
5. Using glue, assemble the lower portion of the hopper, assemble the two trapezoidal panels and the two “Rectangle 3” panels together with the remaining four triangular “Hopper Corner Brackets” shown in Fig. 90. Join the upper and lower halves of the hopper by attaching a “Hopper Panel Bracket” to the rectangular and trapezoidal panel interface on both sides.

**Fig. 90:**
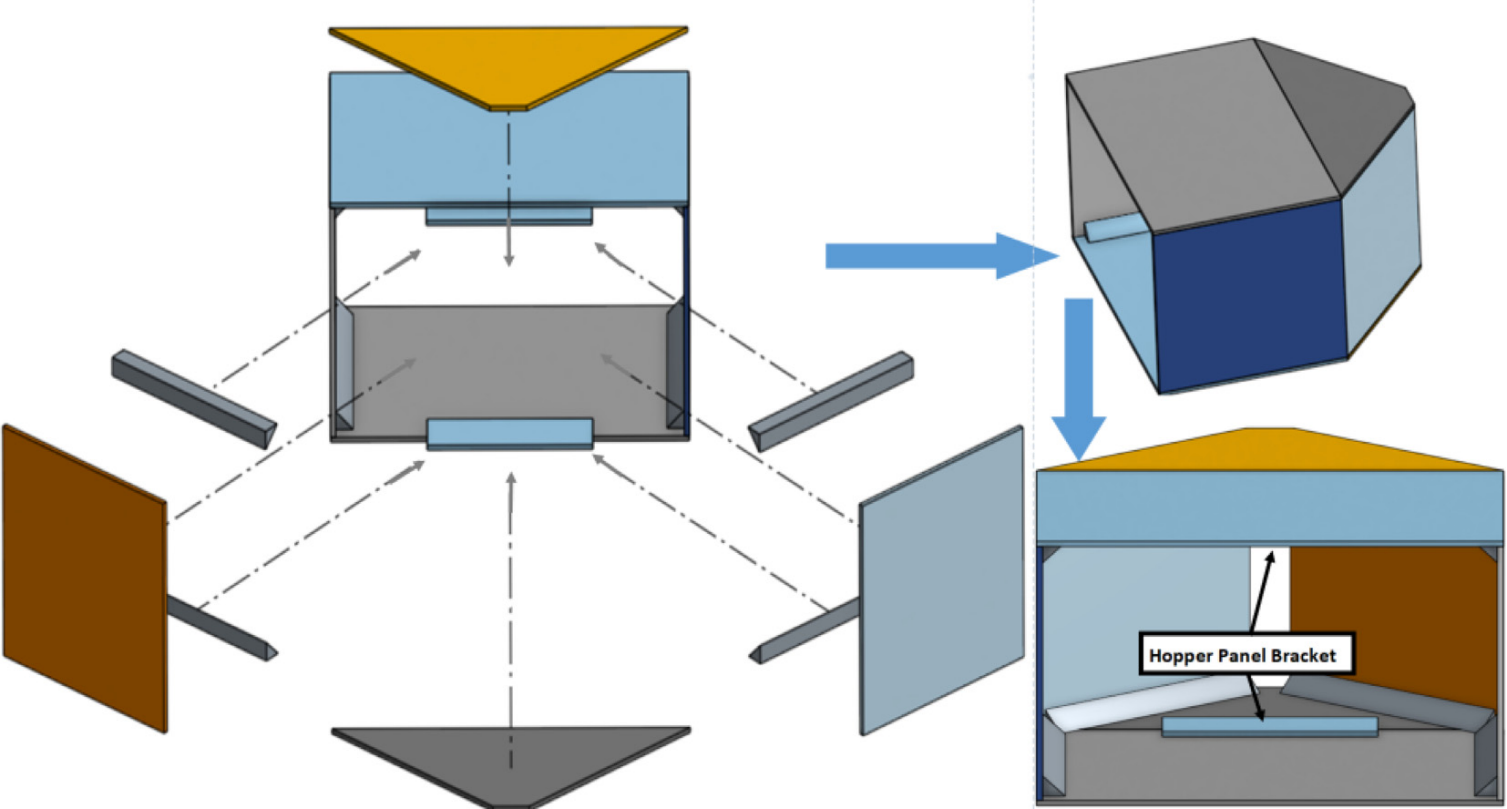
Upper hopper assembly.
6. Repeat step 4 but for each corner of the bottom half of the hopper shown in Fig. 91*.*

**Fig. 91:**
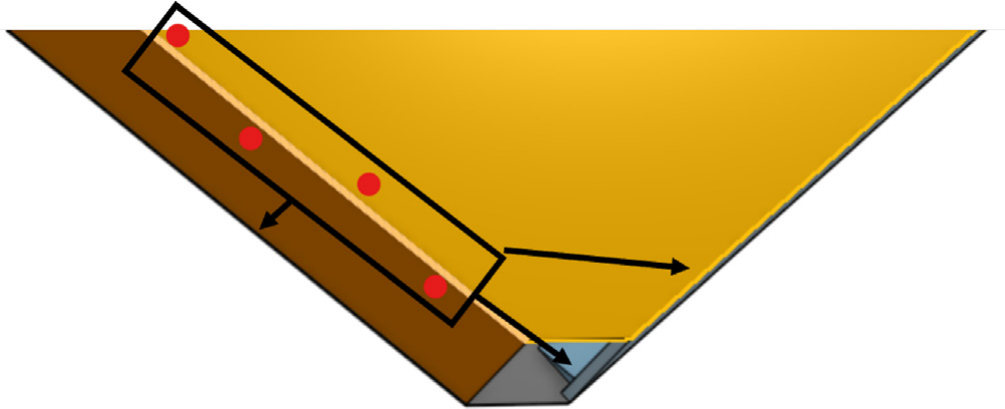
Wood screw locations for bottom half of hopper.
7. Use the four mending plate brackets and eight #6–1/2″ wood screws to affix the two pieces of the hopper on both sides (2 on each side) shown in Fig. 92.

**Fig. 92:**
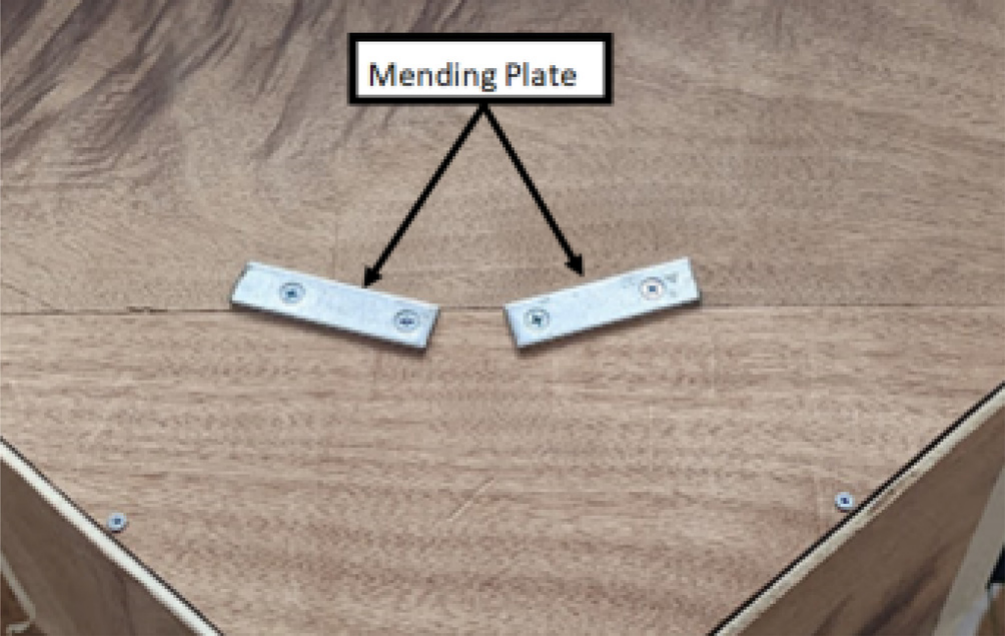
Connecting hopper halves with mending plates on the outside.
8. Next, connect the printed “Feeder Outlet” and “Feed Gear Enclosure” together, as shown in Fig. 93, using super glue or JB

**Fig. 93:**
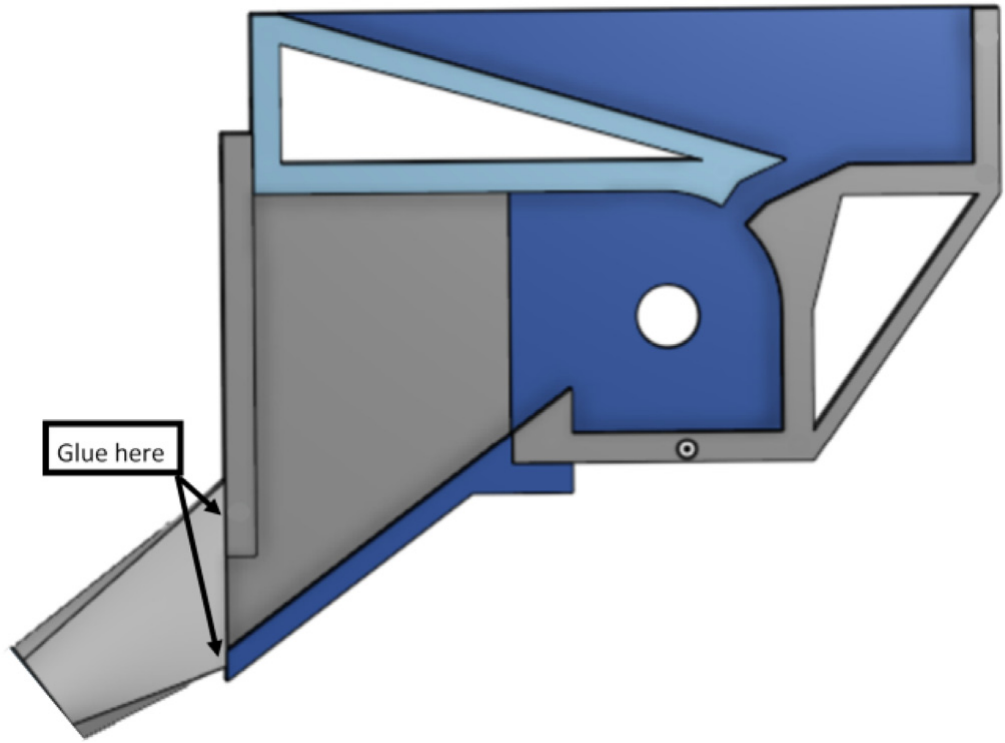
Assembling “Feeder Outlet” to “Feed Gear Enclosure”.
9. Drill 10 holes using a 5/32″ drill bit and drill to fit te M4x16mm Phillips screws in the locations shown below in Fig. 94. These holes are for mounting the acrylic cover that will enclose the printed assembly.

**Fig. 94:**
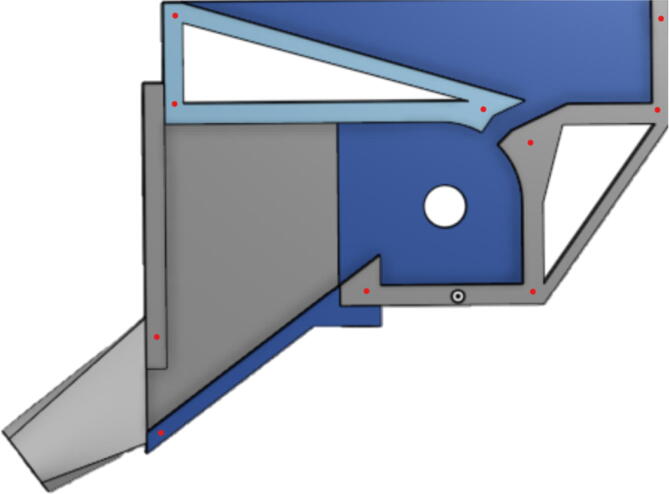
Acrylic cover mounting hole locations.
10. Cut the 8 mm shaft down to 50 mm with an angle grinder.
11. Attach the 8 mm shaft to the back of the feed gear as per Fig. 95

**Fig. 95:**
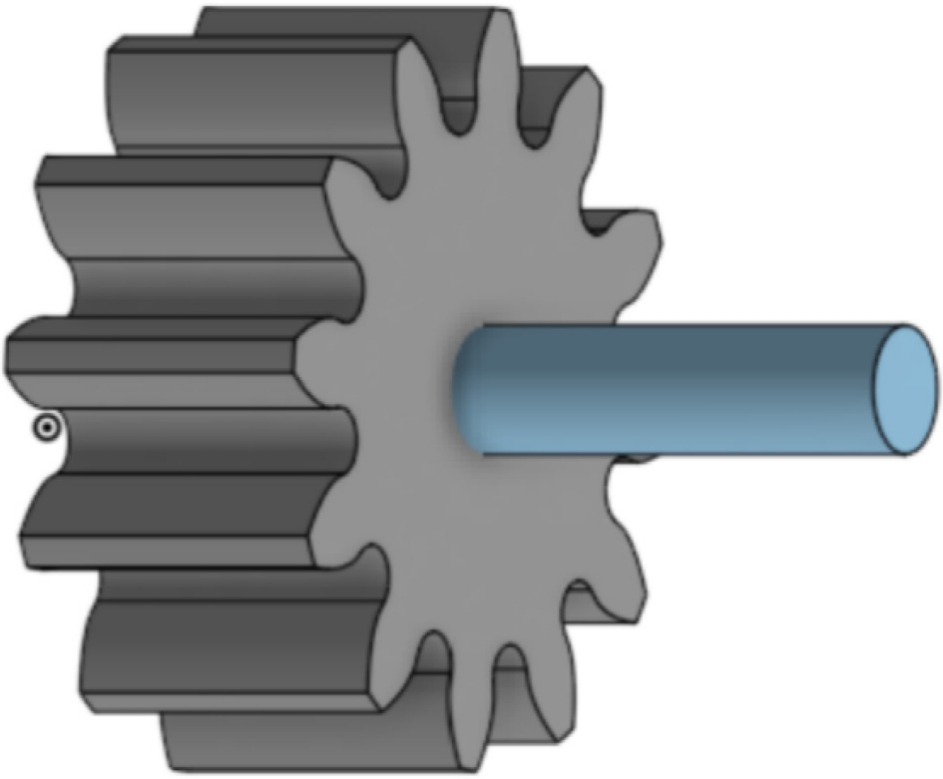
Feed gear and shaft.
12. Affix the 608ZZ bearing to the inside of the hole on “Feed Gear Enclosure” as shown in Fig. 96. This ensures smooth ration between the motor and gear within the enclosure assembly

**Fig. 96:**
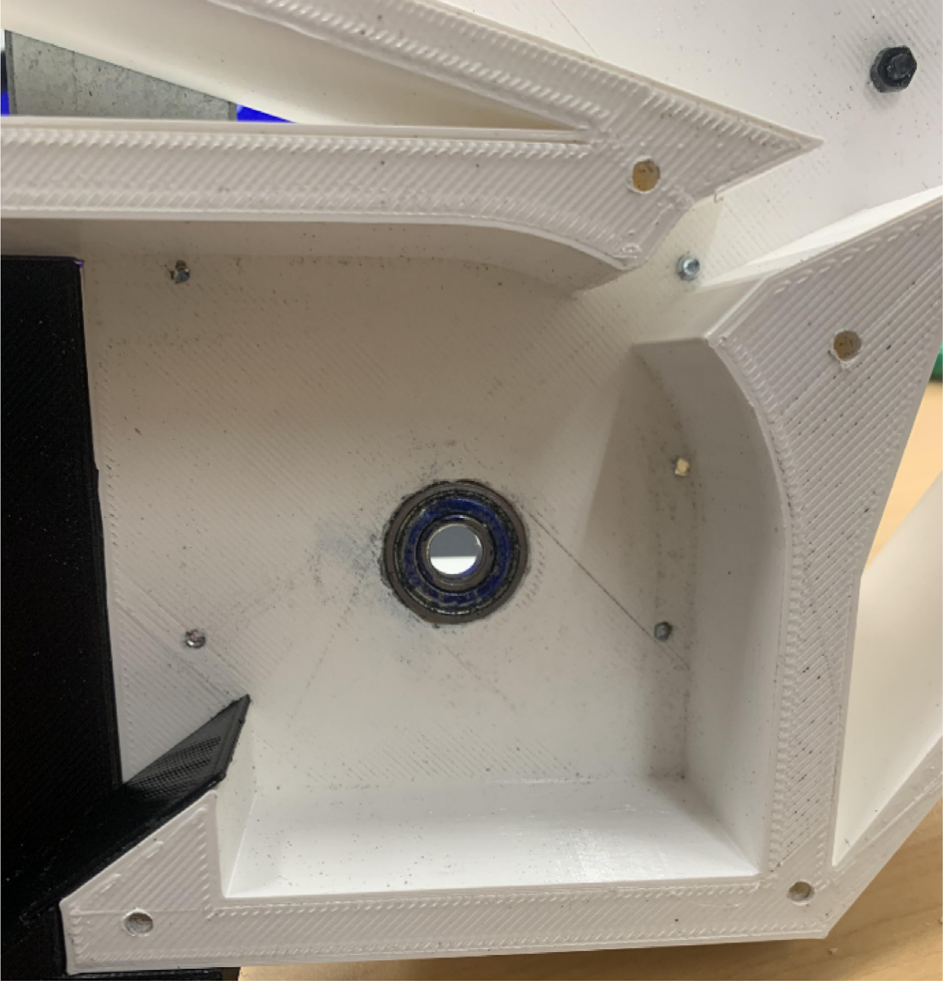
608 Bearing insert.
13. Drill four holes on the back of the “Feed Gear Enclosure” assembly and affix the printed “Feed Motor Enclosure” that will hold the motor, with four M4x16mm Phillips screws and nuts as shown in Fig. 97. Use a 5/32” drill bit to make the holes for the screws.

**Fig. 97:**
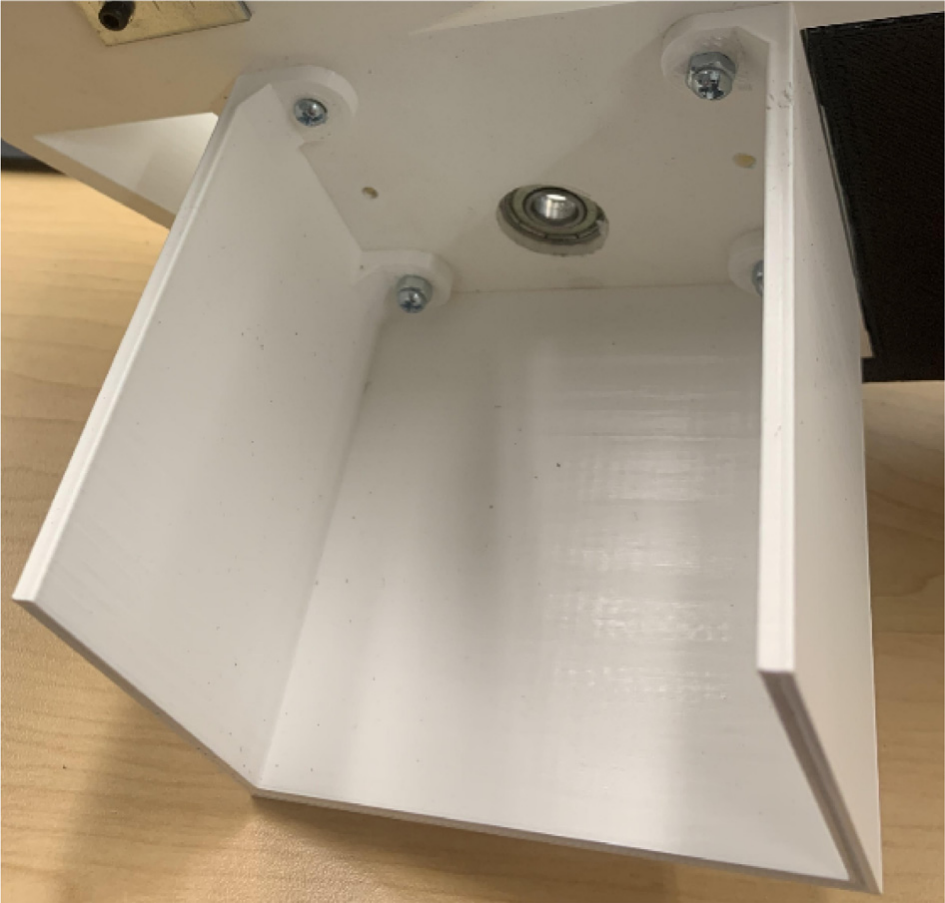
Assembling “Feed Motor Enclosure” to “Feed Gear Enclosure”.
14. Insert shaft and gear to the feed gear enclosure. Then, attach the 8mmx8mm shaft coupler to the other end of the shaft nd connect the shaft couple to the shaft of the NEMA17 geared stepper motor shown in Fig. 98.
  a. Make sure the motor will line up to be able to sit on the platform.
  b. Ensure the feed gear is flush with the outer edge of the “Feed Gear Enclosure” assembly.

**Fig. 98:**
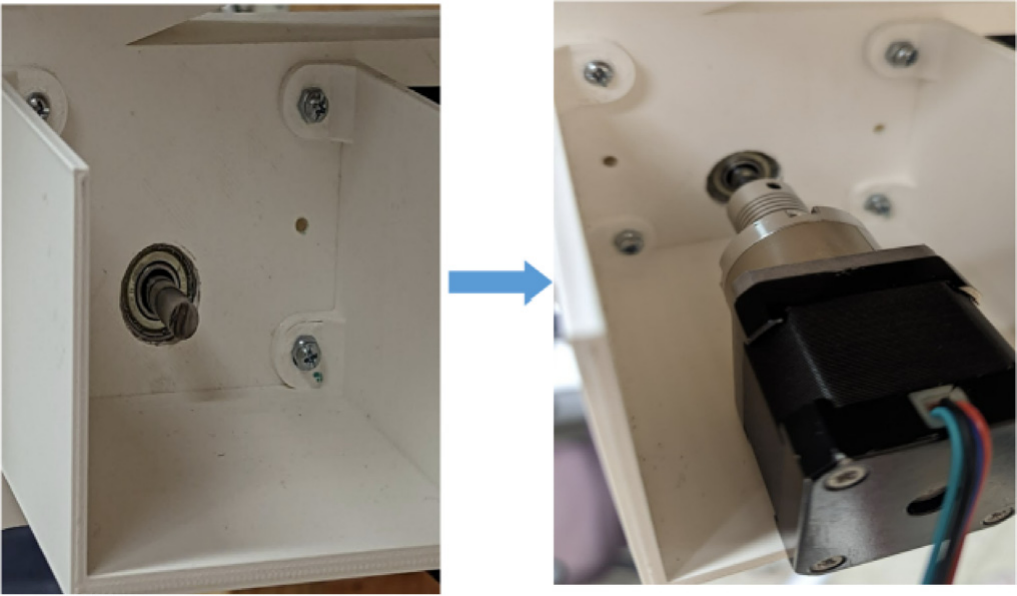
Feed motor assembly.
15. Cut out a piece of the 12″x12″x1/8″ acrylic plastic sheet to make the cover for the feeding assembly by tracing an outline of the “Feed Gear Enclosure” assembly. Cut using a bandsaw or table saw.
16. Use 10 M4x12mm Phillips screws and nuts to attach the cover to the feeding assembly as shown below in Fig. 99.

**Fig. 99:**
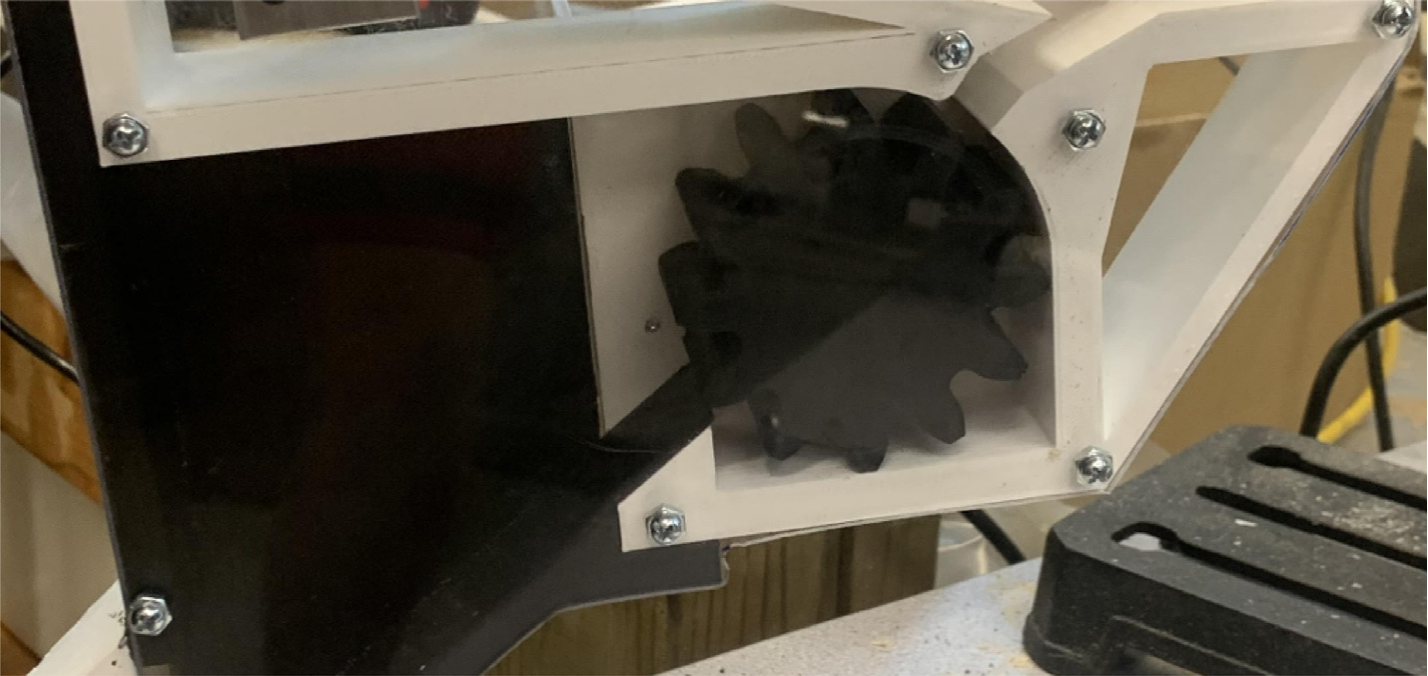
Acrylic cover for feeder enclosure.
17. Take five 2″x2″x1.89″ L brackets and bend them in a vice from 90°to about 135°or until they sit flush with both the feeding assembly and the hopper. Then using ten M4x16mm Phillips screws, screw in the L brackets to the positions shown below in Fig. 100. Locations do not need to be exact. Use M4 nuts to secure the brackets from the other side. Use a 5/32″ drill bit to make the holes for the screws.

**Fig. 100:**
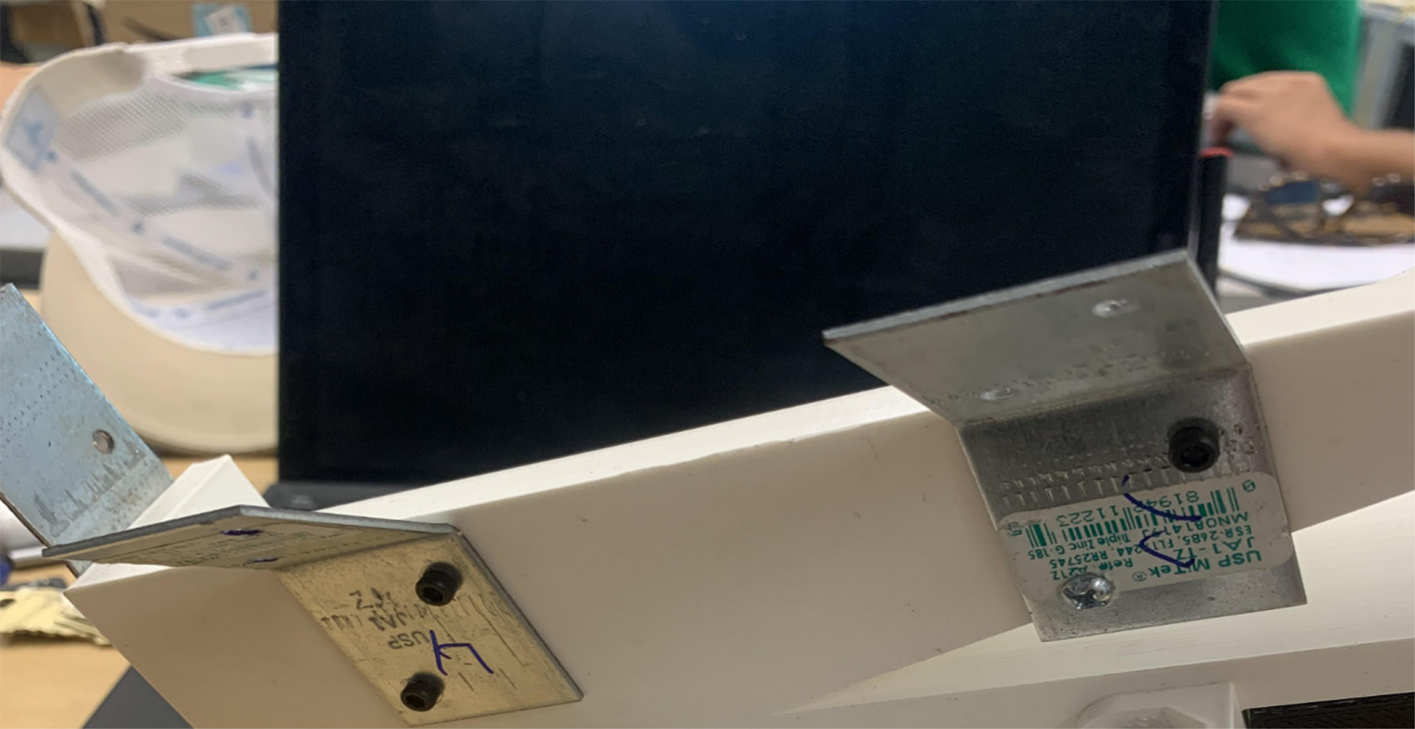
Fastening L brackets to feeder enclosure.
18. Affix the feeder enclosure to the hopper and mark out the spots for the brackets to be fixed to the hopper. Use ten more M4 Phillips screws and nuts to secure the hopper to the feed enclosure assembly as shown below in Fig. 101.

**Fig. 101:**
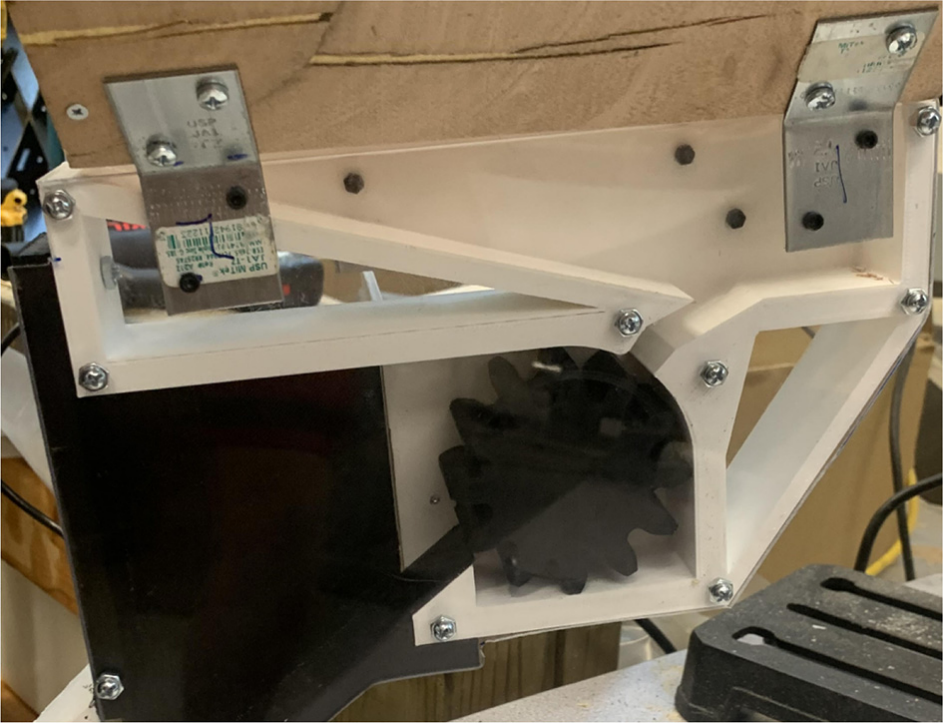
Assembled feed enclosure and hopper.
19. On the shorter widths of the hopper, drill 3 holes on each side with a 5/32″ drill bit according to the dimensions shown below in Fig. 102.

**Fig. 102:**
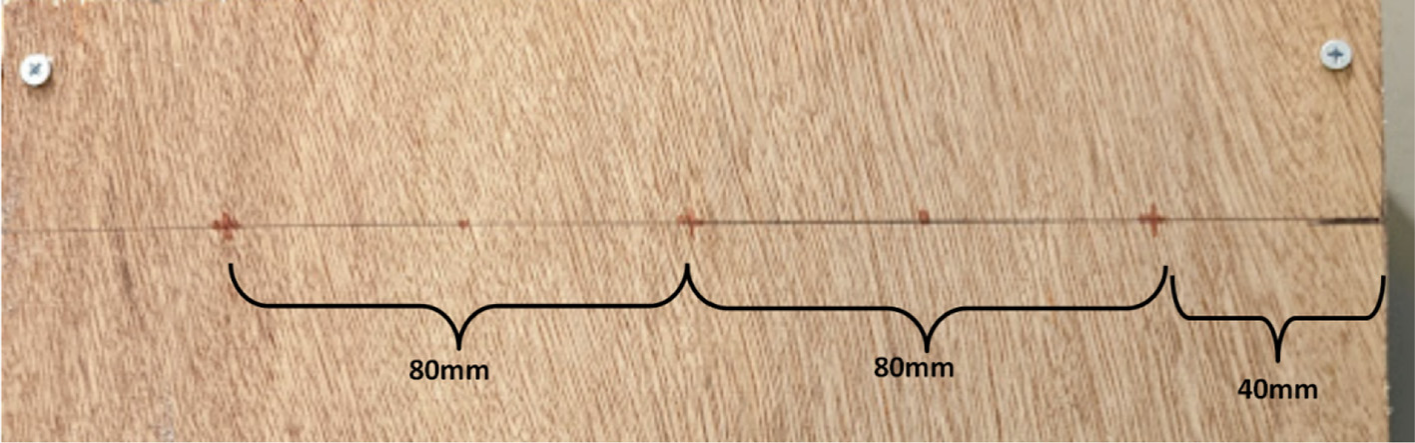
Dril hole locations for mounting screws on hopper.
20. Prepare six M4x16mm screws, M4 nuts and washers in the arrangement shown below in Fig. 103. Insert these screws into the sides of the hopper as shown in Fig. 104. Add six additional nuts on the other side of the hopper side panels to secure mounting screws.

**Fig. 103:**
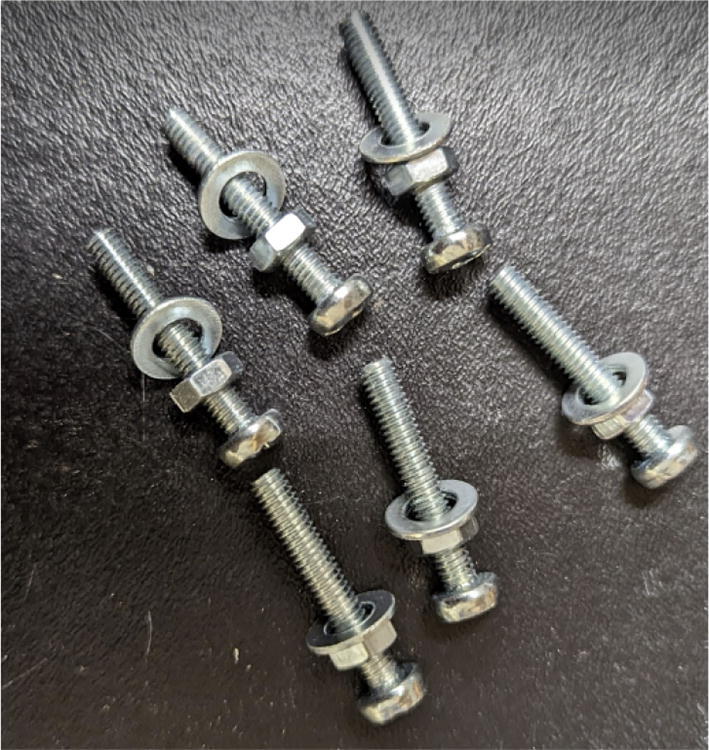
Hopper mounting screw arrangement.

**Fig. 104:**
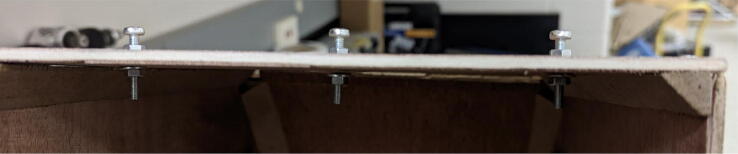
Hopper mounting screw installation.
21. Take the 11/16″x3-1/2″x 8′ particleboard plank and cut two pieces according to the dimensions below in Fig. 105. Then using eight #5x3/4 flathead wood screws and four 2″x5/8″ l-corner braces, fasten 2 L braces on each plank according to the dimensions shown in Fig. 105

**Fig. 105:**
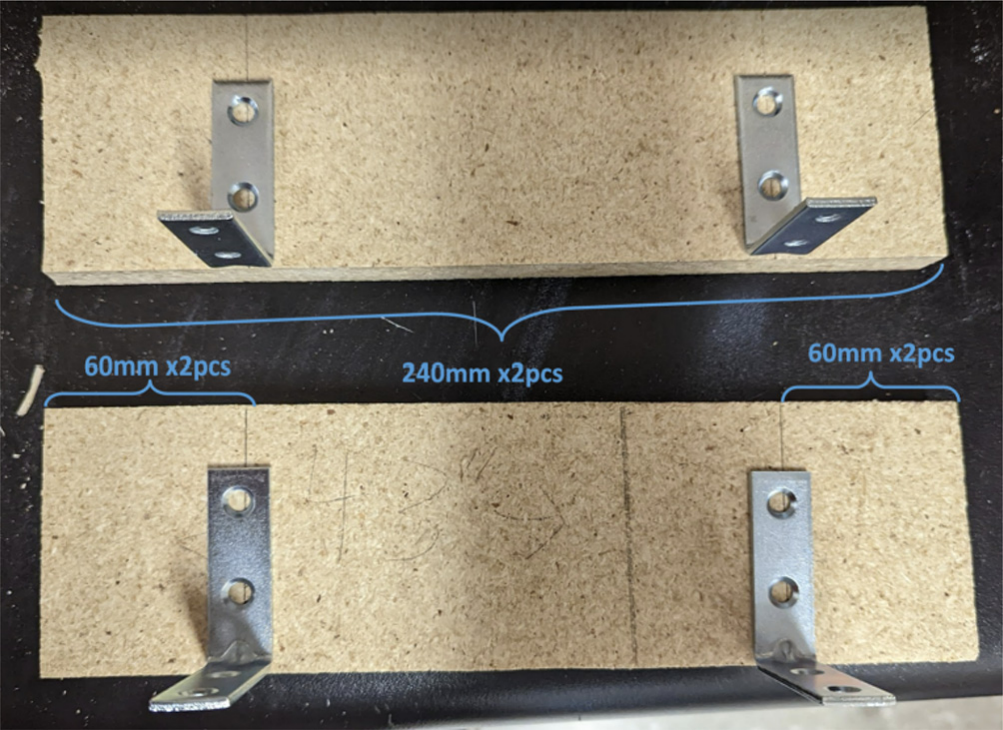
Hopper mounting bracket dimensions to cut and arranging L brackets.
22. Secure the 20mmx20mmx914mm aluminum extrusion in a vise and using a hacksaw(Fig. 106), cut two extrusions 240 mm in length as shown in Fig. 107.

**Fig. 106:**
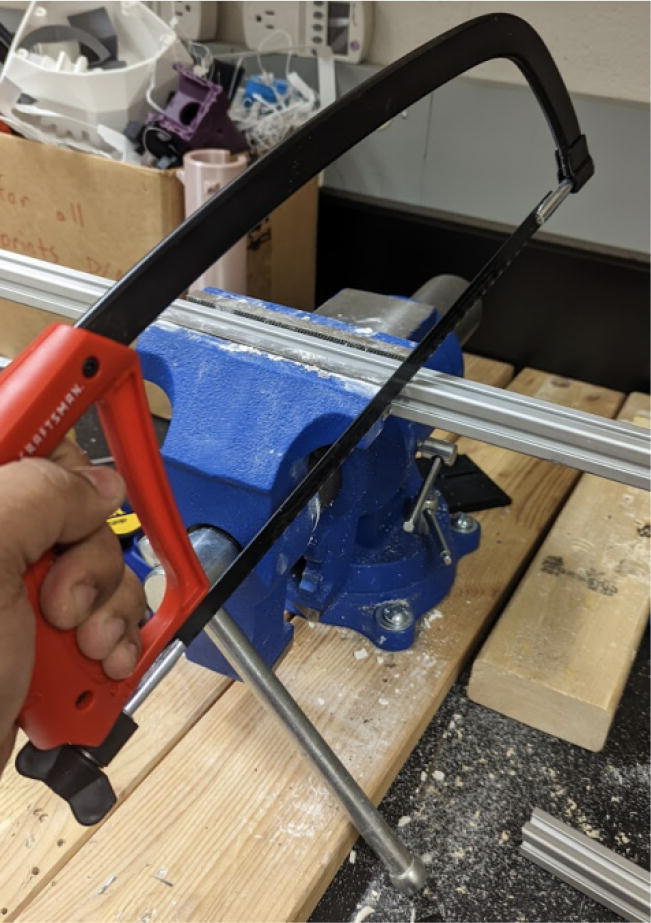
Work holding and cutting of aluminum extrusion.

**Fig. 107:**
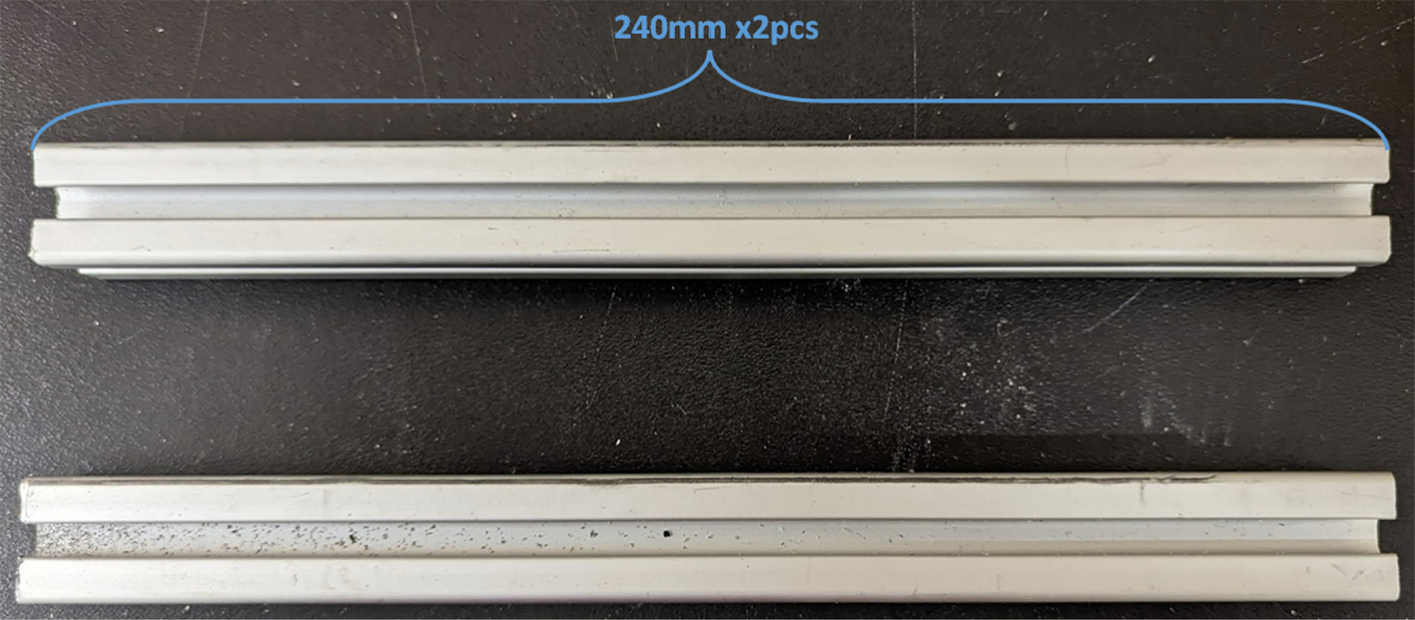
Cut aluminum extrusion dimensions.
23. Using a 5/32″ drill bit and drill, drill 2 holes on each of the cut extrusions 60 mm from the edges as per Fig. 108*.*

**Fig. 108:**
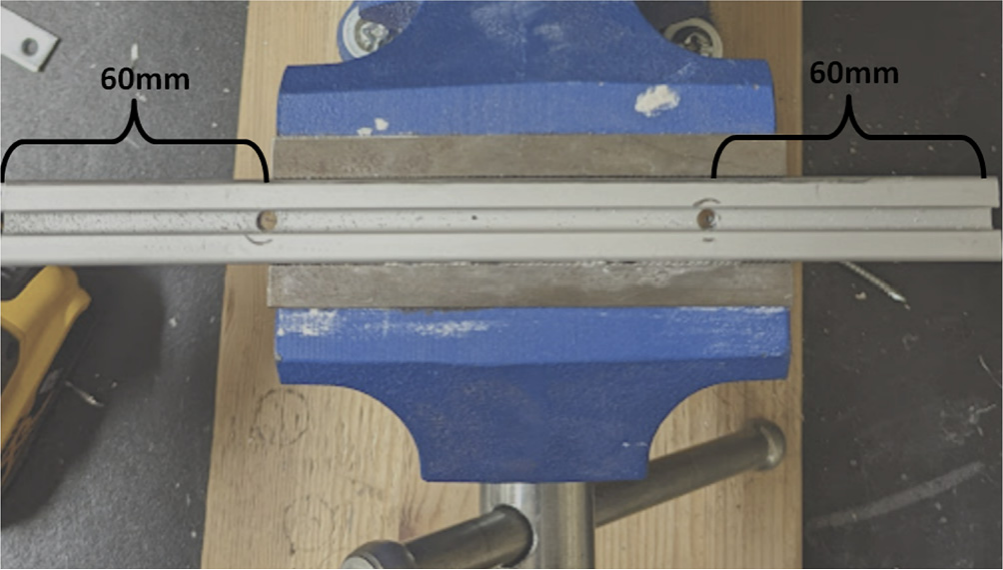
Drill hole locations on extrusion.
24. With the extrusion still in the vise, fasten 2 mending plates onto each cut extrusion using two M4x16mm Phillips screws shown Fig. 109. Secure the assembly with two M4 nuts on the other side of the extrusion for each extrusion shown in. Fig. 110.

**Fig. 109:**
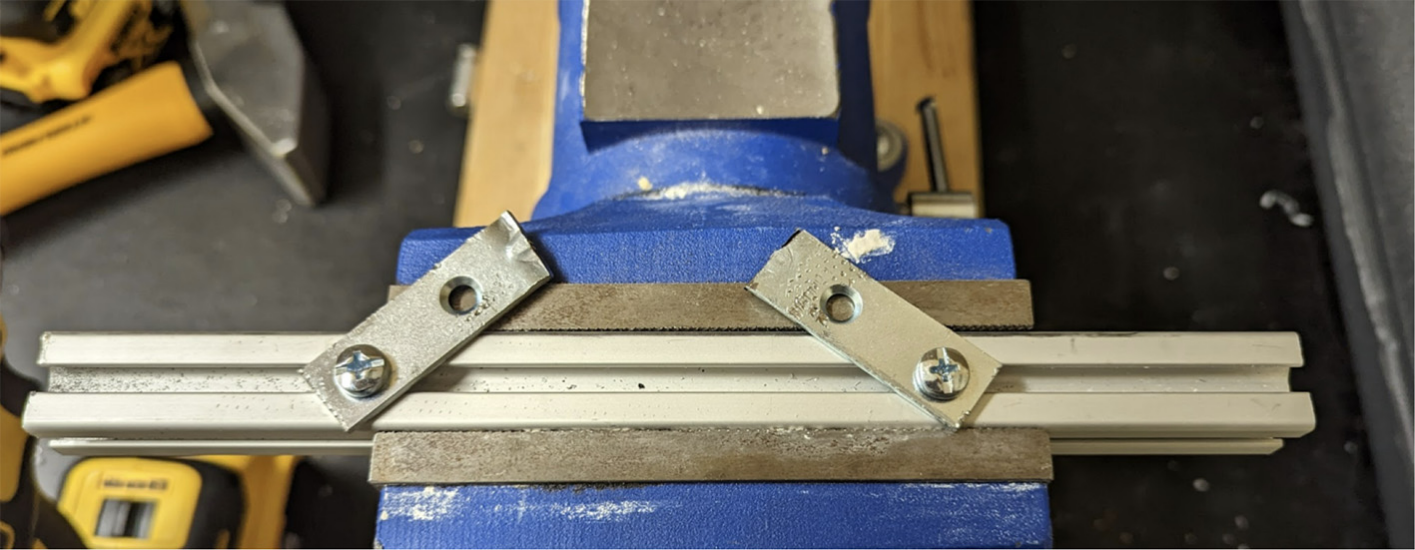
Fastening mending plates onto aluminum extrusion.

**Fig. 110:**
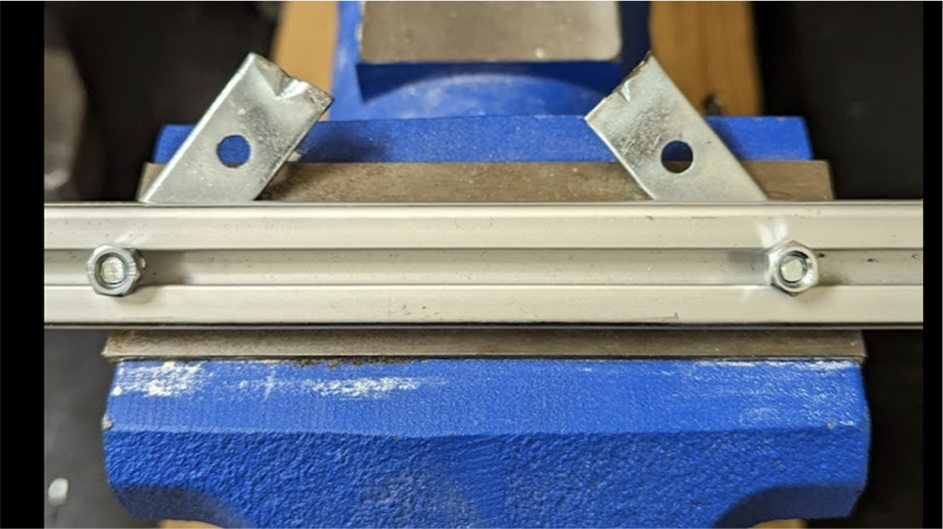
Fastening mending plateaus onto aluminum extrusion other side.
25. Insert the cut particle board clamp into the vise along with the extrusion and align the edges so that the top edges are flush, and the side edges are centered as much as possible shown in Fig. 111. Fasten the other end of the mending plates with 4 (2 for each plank) #5x3/4 flathead wood screws. This completes the two mounting brackets for the hopper.

**Fig. 111:**
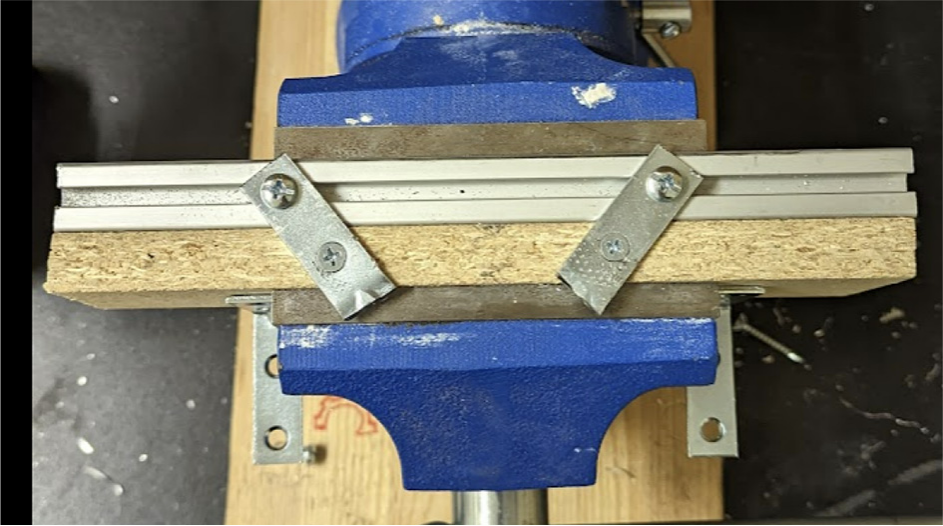
Assembling particle board and extrusion vie mending plate.
26. Mount the assembled hopper mounting brackets to the Hangprinter ceiling mount shown in Fig. 112 using eight #5x3/4 flathead wood screws with the brackets spaced out approximately 430 mm apart. Try to align the feeder outlet of the printed feed enclosure with the center of the two A anchor lines that span from the ceiling mount to the ground anchors.

**Fig. 112:**
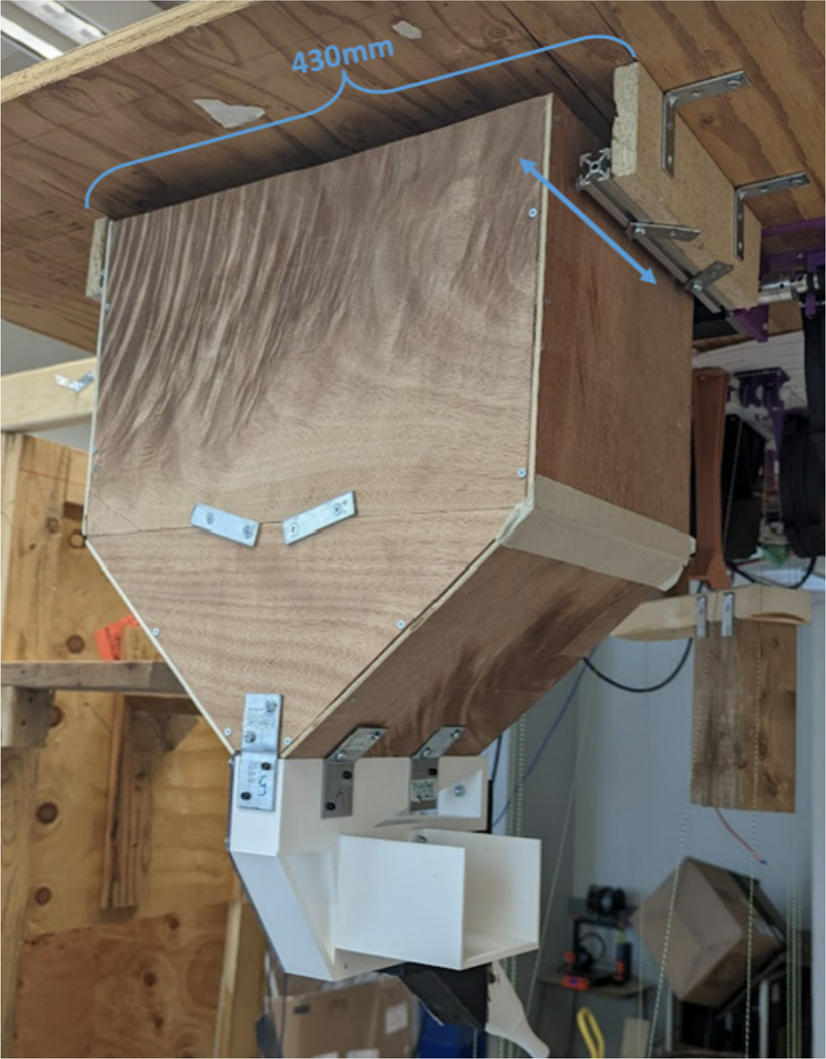
Mounting hopper to ceiling mount.

This completes the Feeding system and hopper assembly on the ceiling mount. The steps below illustrate how to connect the feeding system to the pellet extruder via a flexible hose. These steps are suggestions and certain lengths, configurations and other quantities will vary depending on the users set up. Thus, it is up to the user to decide how to best mount the flexible tube on the ceiling mount and join the feeding system and flexible tube together.

**Fig. 113:**
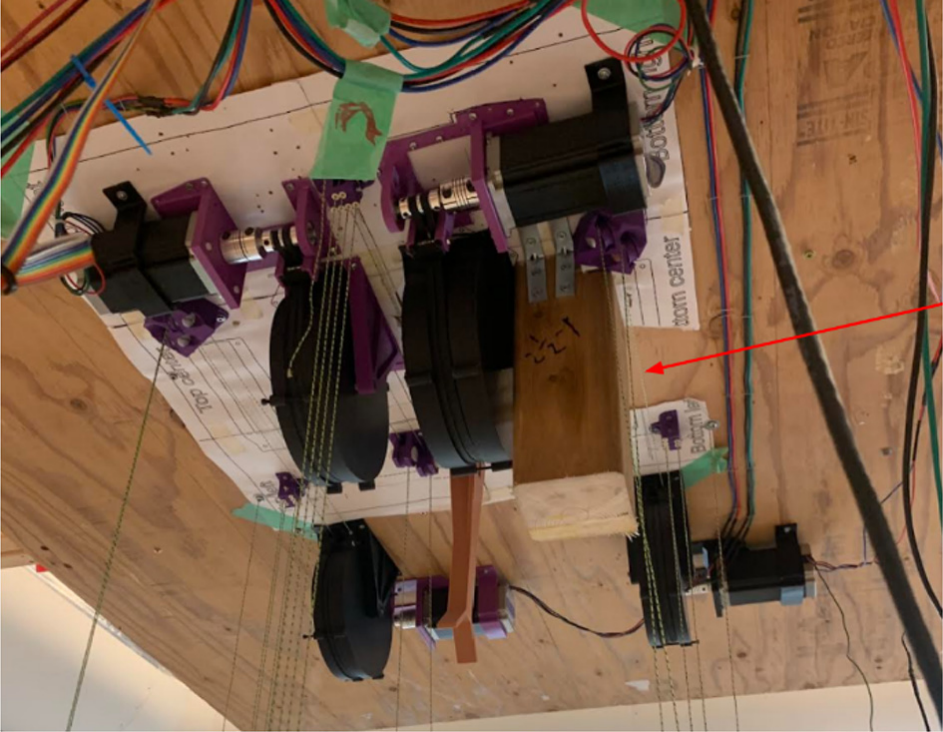
Fixing 4x4 wood block to ceiling mount.
Use 6 mending plates and 12 #6 1/2″ flat head wood screws to fasten the 40 cm square block and 30 cm 2x4 wood block directly over the approximate centre of the hangprinter chassis as shown in Fig. 114. Screw in two additional #6 1/2″ flat head wood screws to secure the wood block indicated by the red arrows in Fig. 114.

Cut a 4x4 wood beam down to a length of 30 cm and another at 40 cm.
Cut a 2x4 piece of wood at 30 cm
Fasten the 30 cm piece of wood to the ceiling using four 2″x5/8″ l-corner braces and four #6 1/2″ flat head wood screws as shown in Fig. 113.

**Fig. 114:**
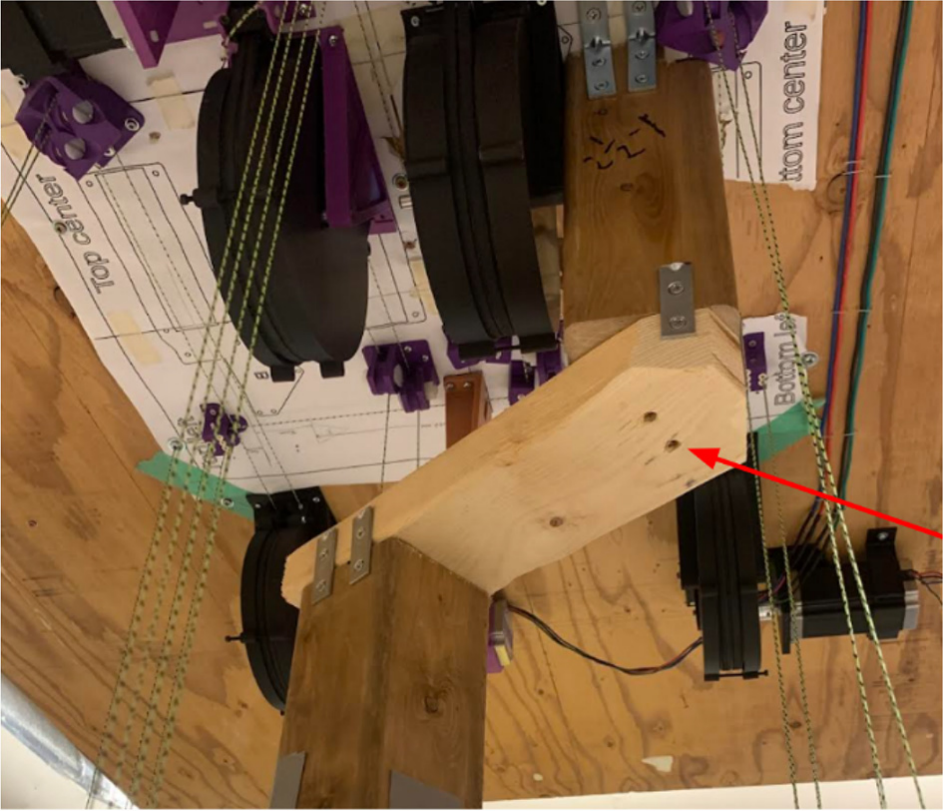
Installing mount for flexible tube onto 4x4 wood block.
Twist the printed “Feeding Tube Coupler” onto the flexible tube as shown in Fig. 115.

**Fig. 115:**
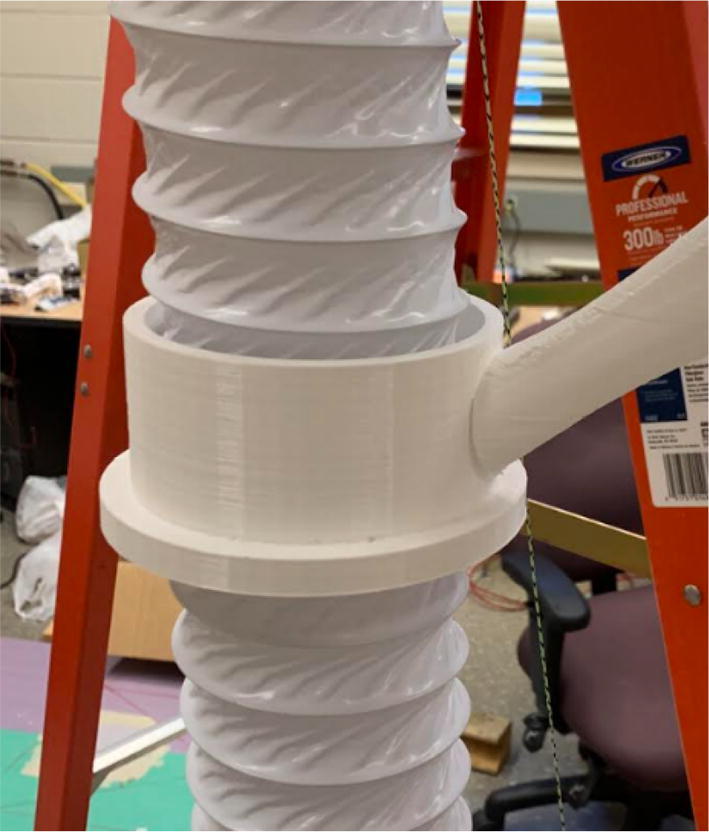
Fitting the “Feeding Tube Coupler” onto the flexible tube.
Attach the white flexible tube to the bottom of the wood block with two #5x3/4 flathead wood screws long washers. Then wrap with duct tape as shown in Fig. 116.

**Fig. 116:**
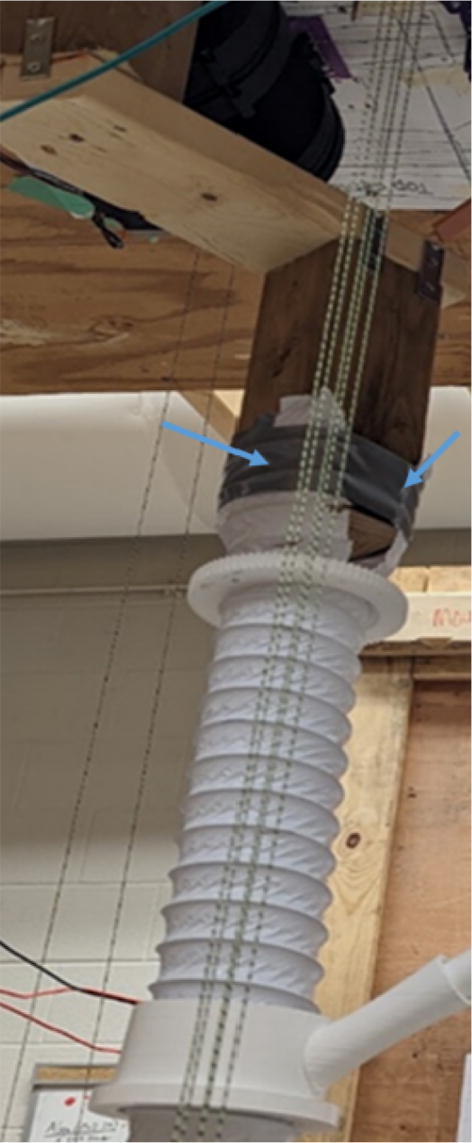
Installing flexible tube onto 4x4 wood block support.
Once the coupler is in place, cut out a hole in the flexible tube about the same diameter as the inlet of the coupler. Take the PEX 3/4″x10′ waterline pipe and cut it down to approximately 30 cm in length. Attach between the coupler and hopper as shown in Fig. 117.

**Fig. 117:**
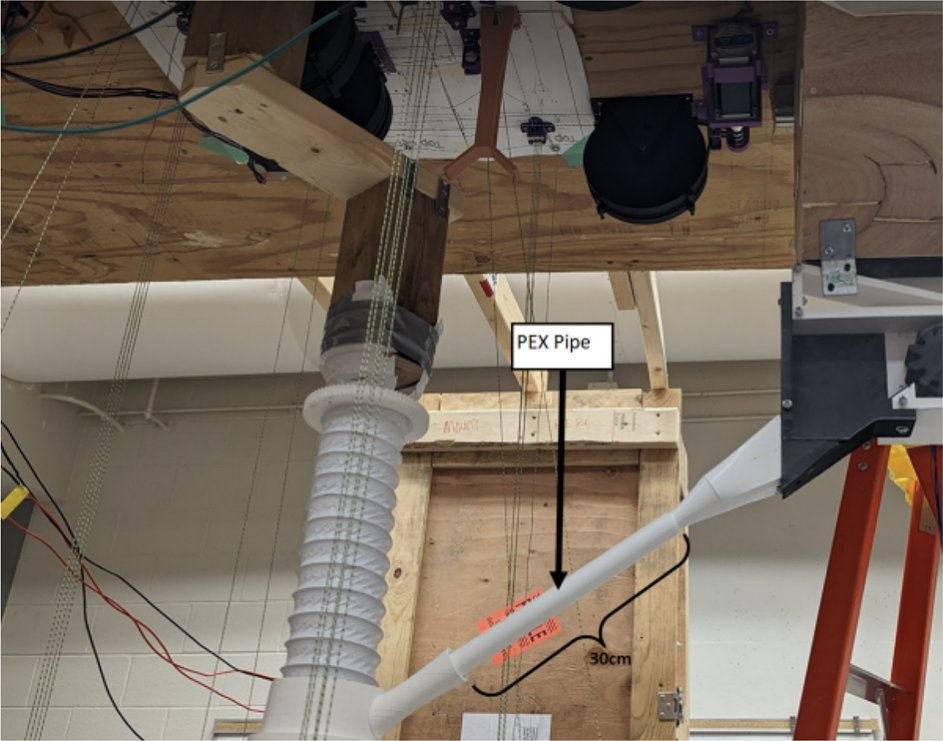
Joining the flexible tube and feeding system assembly.

### Wiring the electronics

#### Establishing pellet extruder to DUET3 board connections

*Break-out board and extruder component wiring harness crimping:*

The extruder electronics connect to the DUET3 controller on the ceiling mount via an intermediate breakout board on the extruder. This allows cease of removal and storage. The tools required for wire the breakout board and wire harness are listed in Table 8. The wiring of components on the pellet extruder connects to an intermediate breakout board and then a separate set of wire harnesses connect the breakout board to the DUET3 Controller. Only the pellet extruder side of the wire harnesses are shown in this section. The connectors and crimping involved in the DUET board connections are shown in the Hangprinter and Overall System section.

**Table 8:** Electrical components and tools needed for pellet extruder to breakout board wiring.

**Item /Tool **	**Quantity**	**Image **
4 Pos JST Female Socket w/ pin connectors (FROM JST KIT) (PES 003)	5	
2 Pos JST Female Socket w/pin connectors (FROM JST KIT) (PES 003)	4	
4 Pos JST Male Plug (FROM JST KIT) (PES 003)	5	
2 Pos JST Male Plug (FROM JST KIT) (PES 003)	3	
Spade crimp connector from space connector kit	2	
UEETEK Ribbon Cable (PES 001)	1	
Assorted 14-16AWG Wire Spools	1	Red, Black, Green, Blue, and White
Universal Perf Board	1	
Nema17 51:1 Stepper Motor	1	
6015 Blower Part Cooling Fan	2	
5015 Axial Heatsink Fan	2	
PT1000 Sensor and Wire Harness Included	2	
TEMPCO Heating Band (PES 002)	1	
Crimping Tool	1	
Wire Stripper	1	

*Process:*

**Fig. 118:**
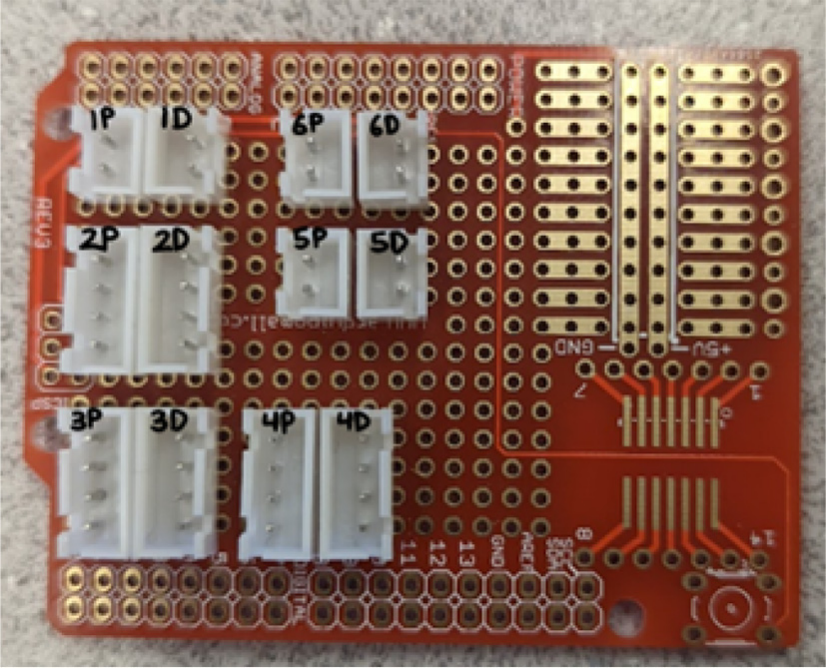
Breakout board top view.

**Fig. 119:**
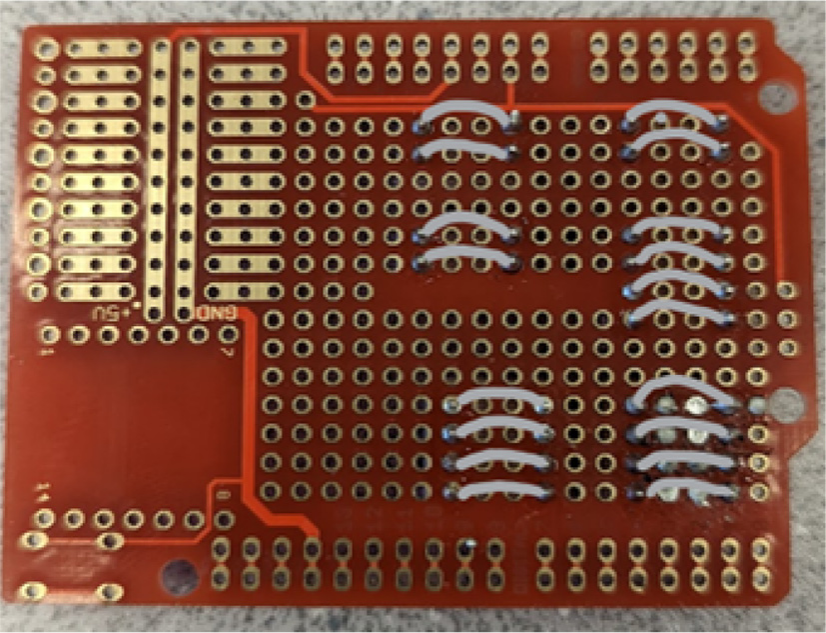
Breakout board bottom view.

The breakout board is shown in Fig. 118 and Fig. 119. Solder the male 2pos and 4pos JST male plugs according to the picture below. Solder jumper wires to join both adjacent connectors together. The left-side of the connectors (i.e., pellet extruder side) are for wiring pellet extruder components to the board. The adjacent right-side connectors (i.e., Duet3 Side) connect the wire harnesses for the respective components from the board to DUET3 on ceiling mount.

**Fig. 120:**
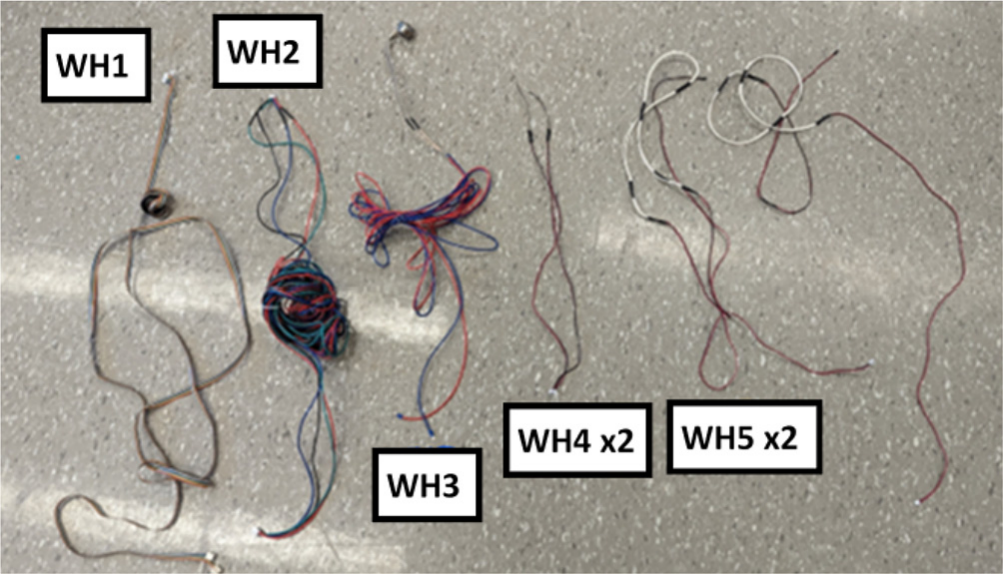
All Wire Harnesses Running from Pellet Extruder to Ceiling Mount/DUET3 Controller.

The necessary wire harnesses for connecting pellet extruder components to the DUET3 controller are shown below in Fig. 120 and Table 9. NOTE: all wire harnesses from extruder to ceiling mount are approximately 2.5 m. Depending on the target print height users will need to size accordingly.

**Table 9:** Description of wire harnesses from pellet extruder to ceiling mount/DUET3 controller.

**WH# **	**Quantity**	**Description **
WH1	1	Rainbow Ribbon cable connects the 2 heatsink fans and 1 part cooling fan to the ceiling mount (full size)
WH2	1	16-18AWG black, green, blue, red wire for extruder NEMA17 motor to DUET3 on ceiling mount
WH3	2	18AWG black, red wire for heating band to SSR on ceiling mount
WH4	2	22AWG wire harness (included with sensor) for Pt1000 sensor to breakout board
WH5	2	22AWG wire harness w/ extension for Pt1000 sensor from breakout board to DUET3 on ceiling mount

*Crimping Heat Sink Fan Wiring Connections:*

**Fig. 121:**
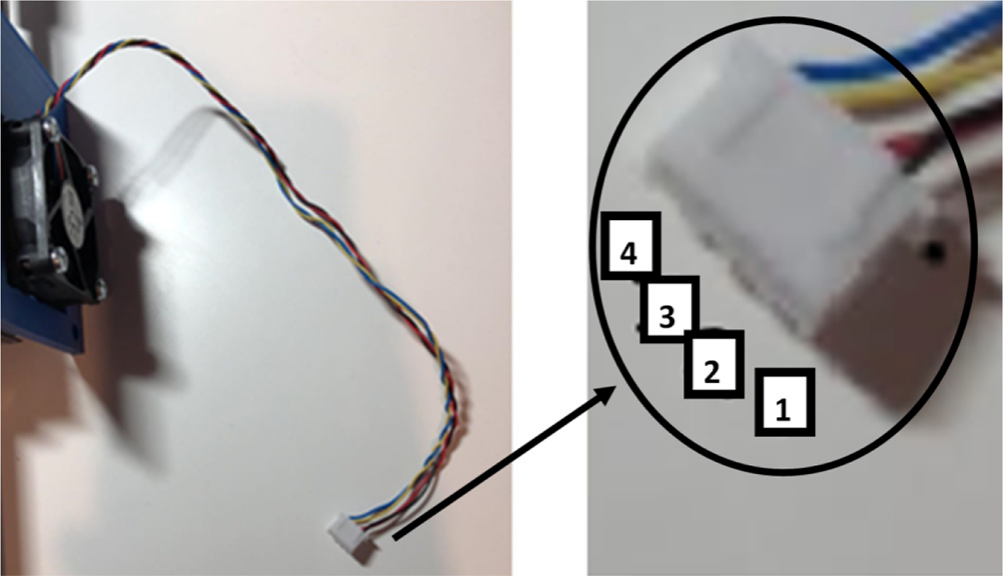
Connector on Heat Sink Fans 1 & 2.

**Fig. 122:**
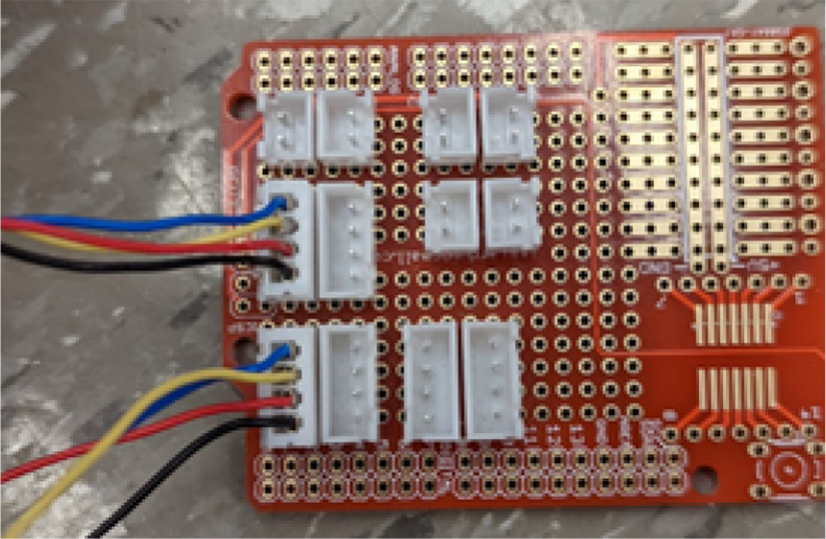
Connecting Heatsink Fan Connectors to Breakout Board.

Crimp all wires using the crimp pins included in the JST-XH Connector Kit with the crimping tool. Strip wires as needed.
Insert into the mating 4 pos female JST socket according to Fig. 121, Fig. 122 and Table 10 Note orientation of connecter and order of wires.

**Table 10:** Heatsink fan (axial Fan) wire color and connector positions.

**Fan Wire **	**Connector Position according to**Fig. 121
GND (black)	1
+12 V/Vcc (red)	2
Tach output (blue)	3
PWM (yellow)	4
Do this for both heat sink fans

*Crimping Part Cooling Fan Wiring/Connection:*

**Fig. 123:**
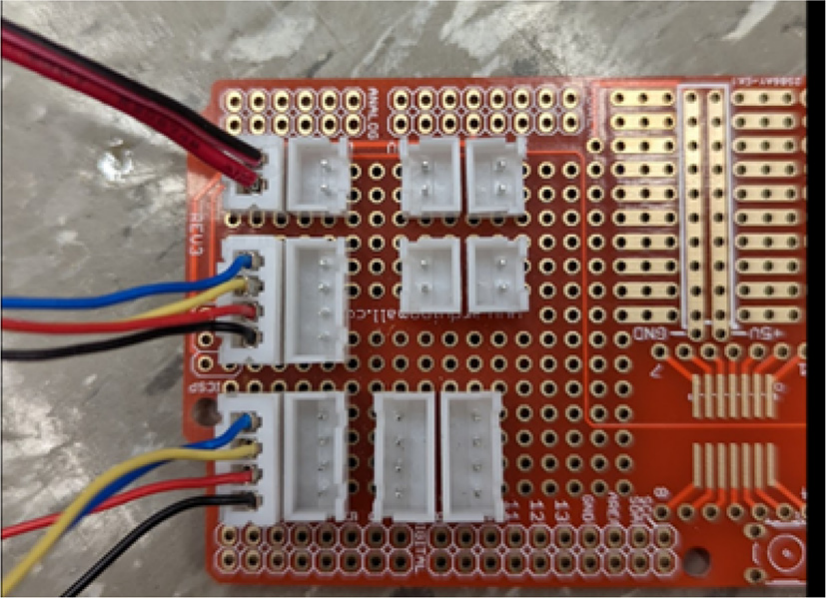
Connecting Part Cooling Fan Header to Breakout Board.

1. Join the red(12 V) of both cooling fans and black (GND) of both fans together.
2. Crimp the red and black wires with the included crimp pins in the JST connector kit and insert into a 2 pos female connector.
3. Connect the part cooling fan connector to the breakout board as shown in Fig. 123 Table 11.

**Table 11:** Part cooling fan (blower fan) wire colors and connector position.

**Fan Wire **	**Connector Position according to**Fig. 123
+Vin (red)	2
-Vin/GND (black)	1

Note: Wire colors may vary, refer to fan data sheet to identify wires. If using different fans for heatsink or part cooling, be sure to refer to its datasheet. Order of wires on breakout board wire harness must be in accordance with order of pins for fan connector on DUET3 board. Refer to following website to learn how to connect fans to DUET3 in more detail [70].

*Crimping NEMA17 Extruder Motor:*

**Fig. 124:**
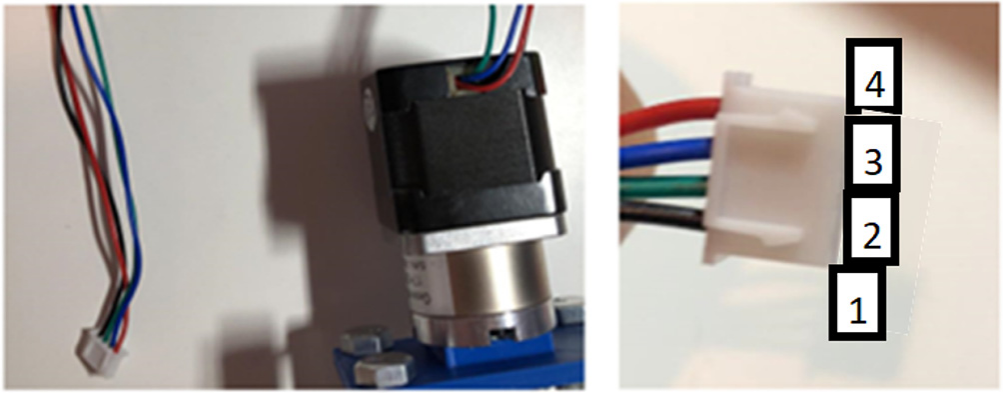
Extruder Motor Pin Connections.

Crimp all wires using the crimp pins included in the JST-XH Connector Kit with the crimping tool (strip wires as needed).
Insert female JST socket according for Table 12 and Fig. 124 Note orientation of connecter and order of wires.

**Table 12:** Stepper motor color and corresponding connector positions.

**Coil**	**Wire Color**	**Connector Position**
A+	Black	1
A-	Green	2
B+	Red	3
B-	Blue	4

***NOTE: Stepper motor wire color may vary. To identify coil pairs, touch different pairs of wires until motor becomes hard to spin. Caution: Mixing the phases up on the 4-pin connector can result in damage to the stepper driver. Be sure to remember order of wires on connector when connecting wire harness from break out board to DUET3 Controller.

**Fig. 125:**
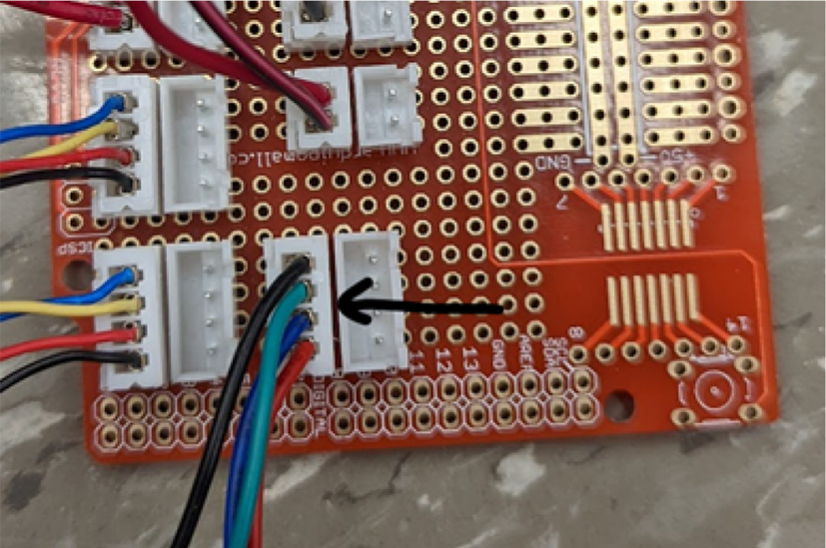
Stepper connector on breakout board.

Connect the stepper motor wires to the breakout board as shown in Fig. 125*.*

*Rainbow Ribbon Cable Wire Harness (WH1):*

**Fig. 126:**
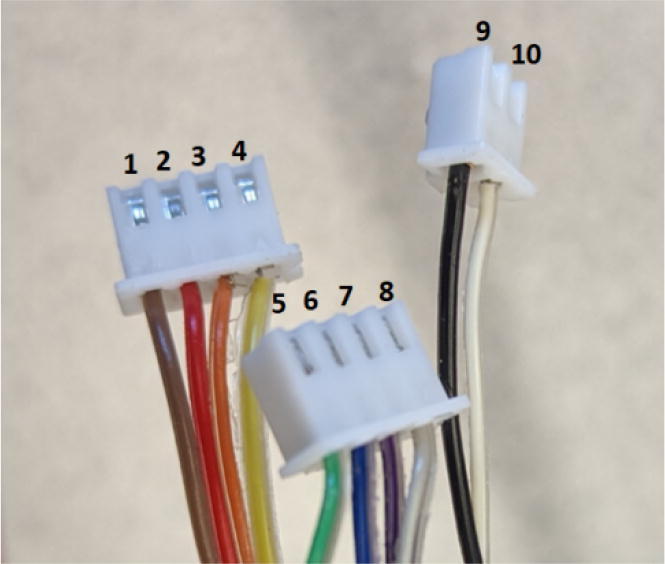
Fan Connectors Wire Harnesses on Breakout Board Side to DUET3 Board.

The 10-wire ribbon cable (PES 001) is used to connect the two 4-wire axial heatsink fans as well as the 2 wire blower fans for part cooling to the DUET3 Controller. Crimp the 4 pos and 2pos Female JST sockets and pin connectors as shown in Fig. 126,Table 13, Table 14 and Table 15. Note the orientation of the connector and order of colors on the connectors. Leave the other side of the ribbon cable empty for now.

**Table 13:** Heatsink fan 1 wire colors and breakout board connections.

**Fan Wire **	**Rainbow Connector Wire/position**	**Break Out Board Connector Number **
GND (black)	Brown (1)	2P
+12 V/Vcc (red)	Red (2)	2P
Tach output (blue)	Orange (3)	2P
PWM (yellow)	Yellow (4)	2P

**Table 14:** Heatsink fan 2 wire colors and breakout board connections.

**Fan Wire **	**Rainbow Connector Wire **	**Break Out Board Connector Number **
GND (black)	Green (5)	3P
+12 V/Vcc (red)	Blue (6)	3P
Tach output (blue)	Purple (7)	3P
PWM (yellow)	White (8)	3P

**Table 15:** Part cooling fan wire colors and corresponding connections.

**Fan Wire **	**Rainbow Connector Wire **	**Break Out Board Connector Number **
+Vin (red)	White (10)	1D
-Vin/GND (black)	Black (9)	1D

NOTE: Color may be different if using different fans or manufacturer. Refer to Table below to ensure proper connections.

**Fig. 127:**
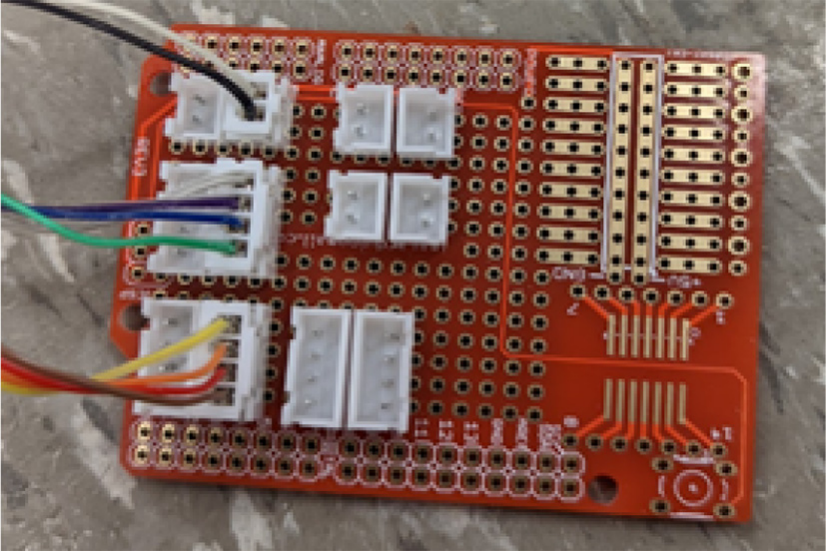
Ribbon Cable Wire Harnesses connected to DUET3 Side Connectors of Breakout Board.

Connect the ribbon cable connectors to the breakout board according to Fig. 127. It does not matter which 4pos male connector the 2 connectors for the heatsink fans are connected.

*Extruder motor Wire Harness (WH2):*

**Fig. 128:**
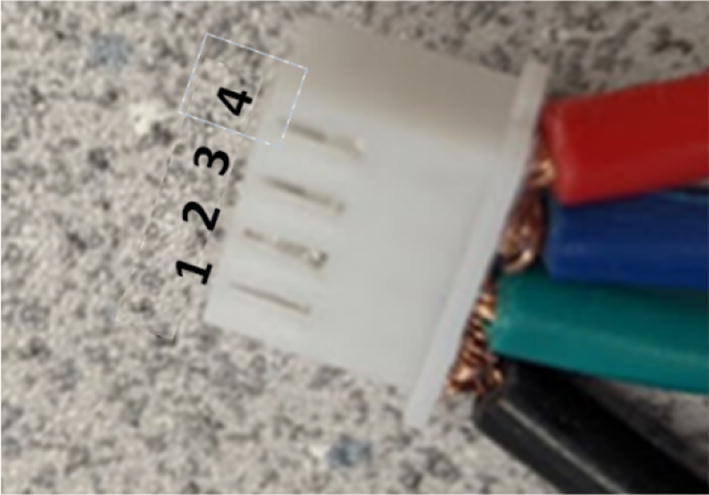
JST Connector Positions on Extruder Motor for Connecting to Breakout Board.

**Fig. 129:**
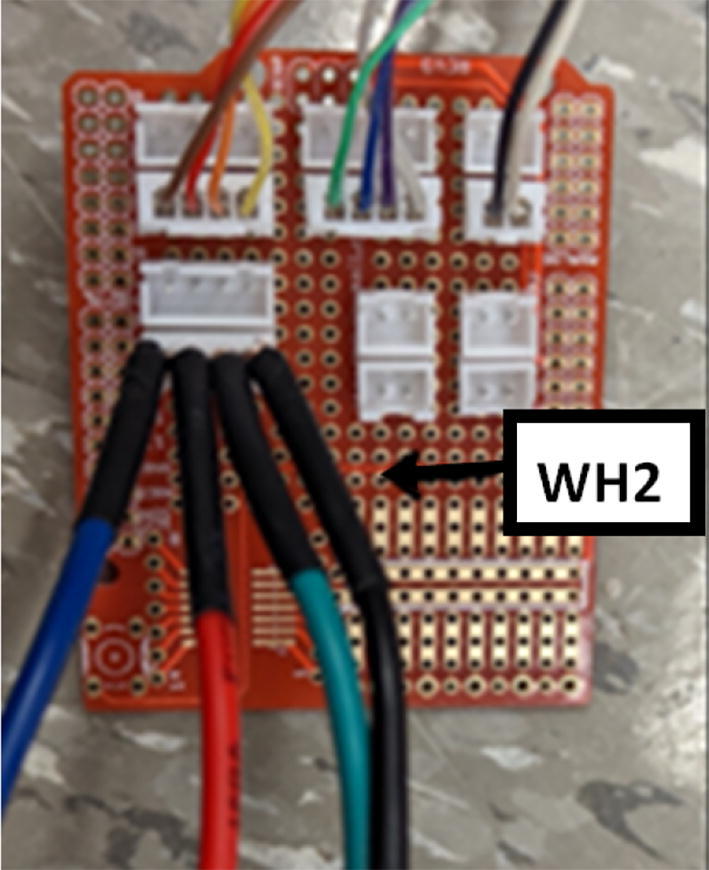
Stepper Motor Harness (WH2) connected to DUET3 Connector Side of Breakout Board.

Using the 16-18AWG assorted spools of wire, cut approximately 2.5 m of black, green, blue and red wire. Strip both ends of wire. Using pin connectors and one 4pos JST Female Socket, crimp wires to the connector according to Figs. 128, 129 and Table 16. Leave the other side of the wires bare for now. Use some heat shrink tubing to create a bundle of the 4 wires.

**Table 16:** Extruder motor wire harness colors and corresponding connector positions.

**Stepper Motor Coil**	**Wire Color**	**Connector Position**
A+	black	1
A-	green	2
B+	blue	3
B-	red	4

NOTE: Stepper motor wire color may vary. To identify coil pairs, touch different pairs of wires until motor becomes hard to spin. Caution: Mixing the phases on the 4-pin connector can result in damage to the stepper driver. Be sure to remember order of wired on connector when connecting wire harness from break out board to DUET3 Controller.

Connect the crimped side of the wire harness WH2 to the breakout board according to Fig. 129.

*Band Heater Wire Harness (WH3):*

**Fig. 130:**
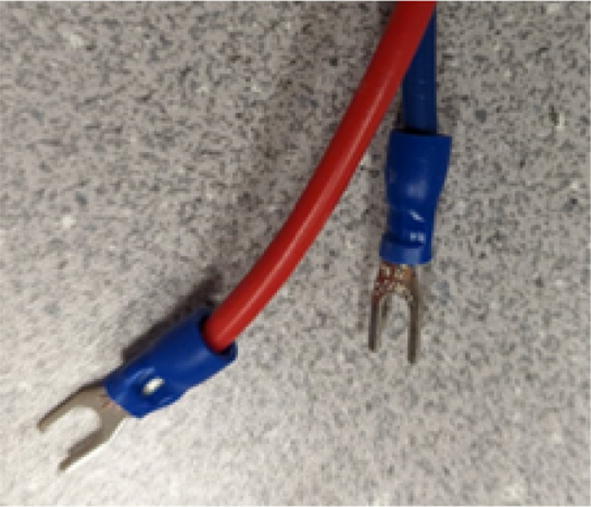
Band Heater Crimping.

**Fig. 131:**
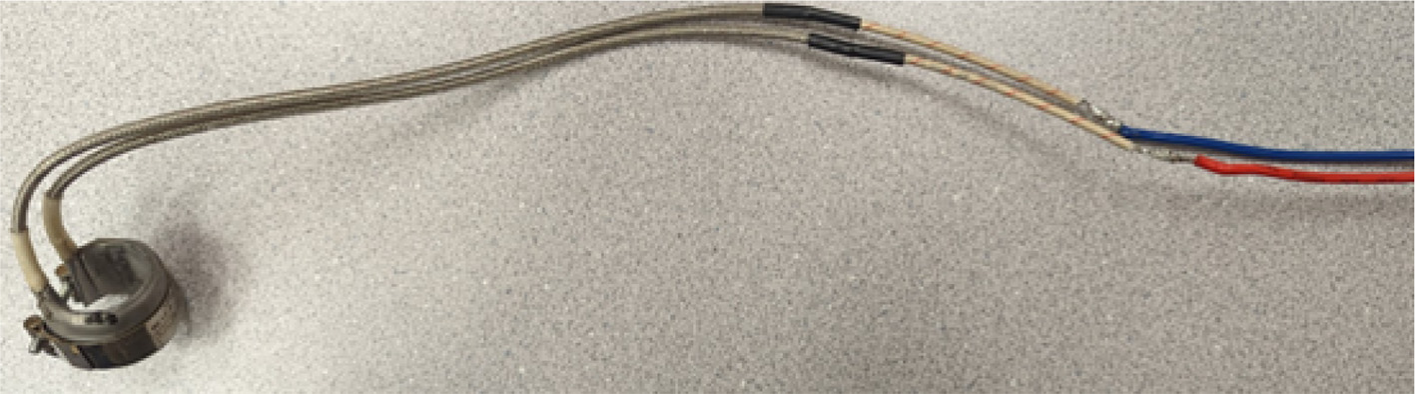
Band Heater and Wire Connection via Solder Joint.

**Fig. 132:**
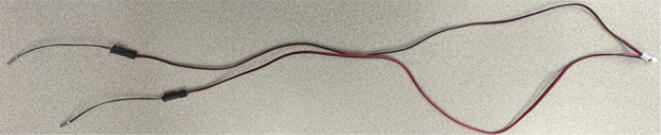
Pt1000 Sensor Wire Harness connecting to Breakout Board.

Using the 16-18AWG assorted spools of wire, cut approximately 2.5 m of blue and red wire. Strip both ends of wire and crimp spade connectors on both wires ( Fig. 130). Solder the other ends of the wire harness to the leads of the band heater. Order does not matter as this is a resistive heating element. Use some heat shrink to cover and insulate the solder joints ( Fig. 131). Do this for both heaters and their respective wire harnesses. This wire harness does not connect to the breakout board and runs directly to an SSR mounted on the ceiling as it is driven by 120VAC (Fig. 132).

*Pt1000 Sensor Wire Harness to Breakout Board (WH4):*

**Fig. 133:**
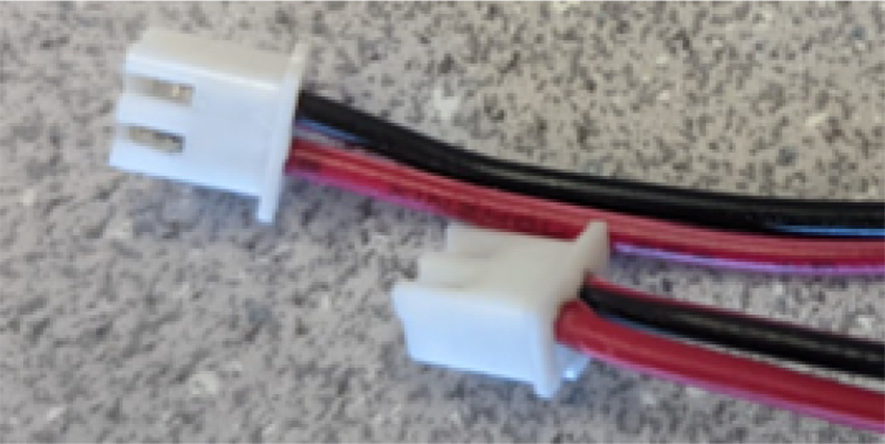
Pt1000 Sensor Wire Harness connecting to Breakout Board.

Use the included 22AWG extension wire included in Pt1000 sensor packaging and cut it in half. Crimp and connect 2pos JST female sockets to the cut end of the wire. The black connected on the other end that connects to the sensor is already pre crimped. (Fig. 133).

**Fig. 134:**
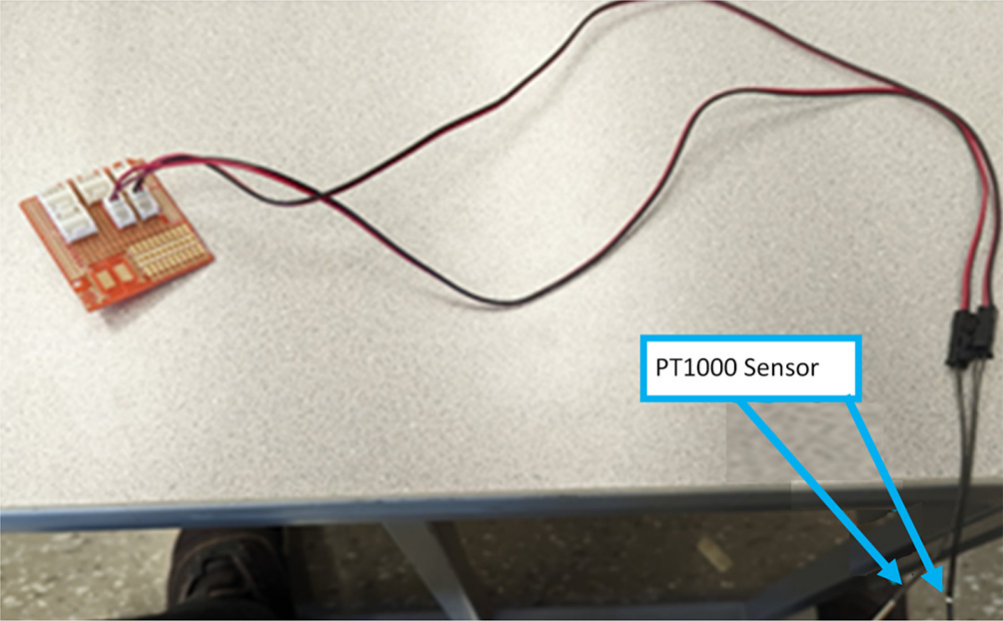
Pt1000 Sensor connection to breakout board.

Connect the wires to the breakout board. Note that the other connectors previously listed are omitted for clarity (Fig. 134).

*Pt1000 Sensor Wire Harness from Breakout Board to DUET3/Ceiling Mount (WH5):*

**Fig. 135:**
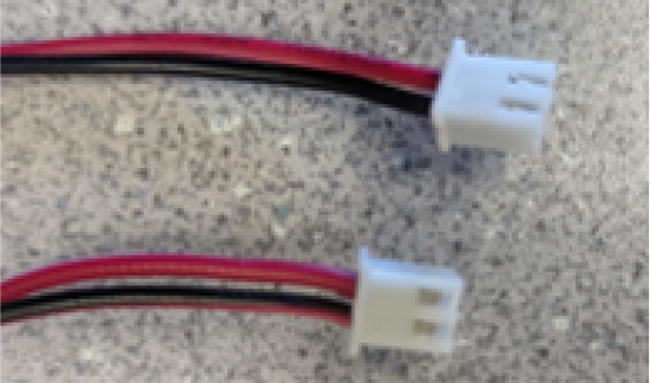
Pt1000 sensor connectors from breakout board to DUET3 board.

**Fig. 136:**
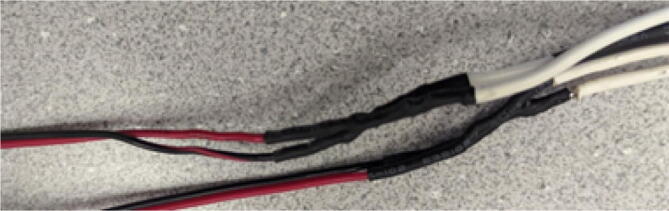
Solder Joint between Pt1000 Harness and White Wire.

**Fig. 137:**
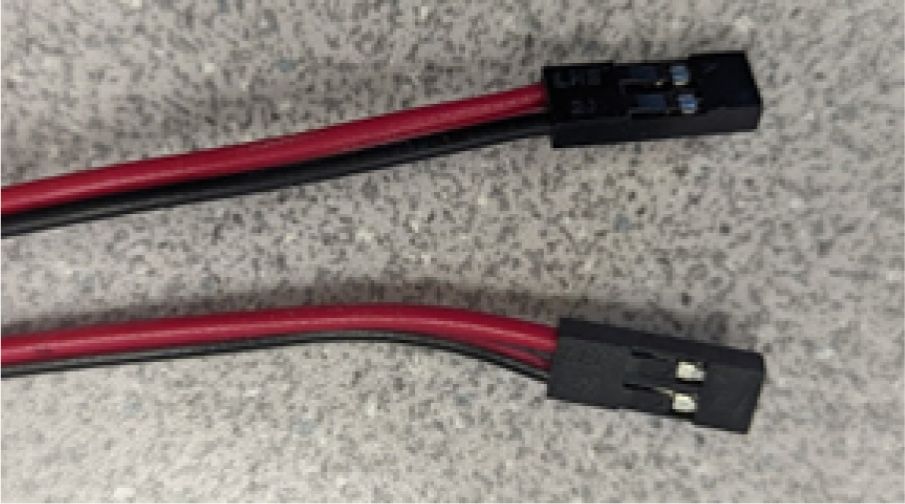
Pre-Crimped DUET3 Side Connector.

**Fig. 138:**
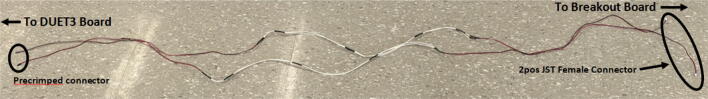
Wire Harness (WH5) for Both Sensors.

Cut two pairs of approximately 1.5 m of white 22AWG wire and solder them to the remaining wire harnesses included in the Pt1000 sensor packaging. Solder the remaining wire harnesses included in Pt1000 packaging to the white cable. For both wire harnesses (1 for each sensor), crimp and connect a 2 pos female JST socket for connecting to the breakout board. Leave the pre-crimped connector on the other side of the harness for connecting to the DUET3 controller. See Fig.134, Fig. 135, Fig. 136, Fig. 137 and Fig. 138.

**Fig. 139:**
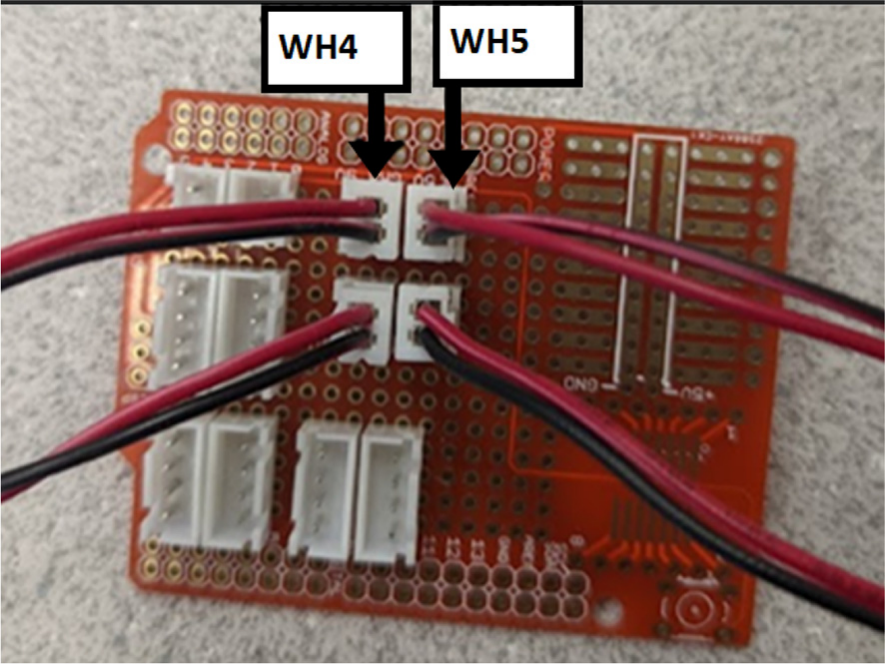
WH5 connected to Breakout Board along with WH4.

Alternatively, fabricators can use two pairs of approximately 2.5 m long 22AWG wire and use the 2 pos female JST connectors to connect one end to the breakout board and the other to the DUET3 controller (if extra wire harnesses not provided in Pt1000 packaging). Connect the WH5 (Pt1000 breakout board to DUET) harness to the breakout board as shown in Fig. 139.

Table 17 provides a summary of connecting wire harnesses and extruder components to breakout board.

**Table 17:** Wire harness and extruder components to breakout board summary.

**Connector **	**Pellet Extruder Component **	**Wire Harness **
1P	2 wire blower fan (PES 014) connector	
1D		WH1(black and white)
2P	4 wire axial fan connector (PES 013)	
2D		WH1(green, blue, purple, white)
3P	4 wire axial fan connector (PES 013)	
3D		WH1(brown, red, orange, yellow)
4P	NEMA17 Stepper (PES 000)	
4D		WH2
5P	WH4 #1(Pt1000 to Breakout board)	
5D		WH5 #1
6P	WH4#2 (Pt1000 to Breakout board)	
6D		WH5 #2

**Fig. 140:**
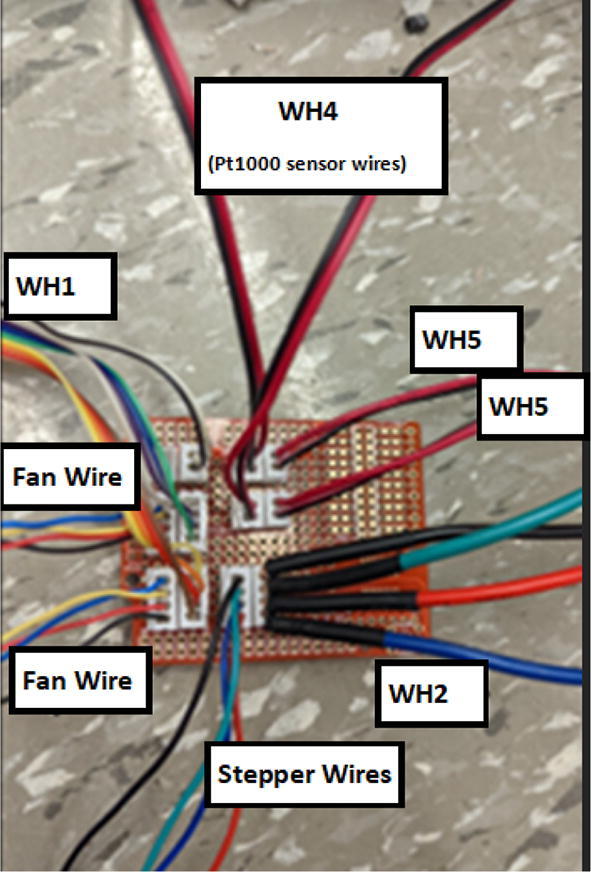
Overall Extruder to Breakout Board Wiring.

Once all wire harness and components are connected to the breakout board, it should look like Fig. 140.

#### Establishing Hangprinter motor to DUET3 board connections

This section shows the termination of the wire harness on the DUET3 side as well as where to connect the components on the DUET and wiring the power supply. Table 18 is a list of components extracted from the master parts list above needed for the full electrical wiring.

**Table 18:** List of electrical components needed to complete hangprinter wiring and pellet extruder connections.

**Component**	**Quantity**	**Image**
DUET3 MBHC Controller and its included connectors	1	
Inkbird SSR	2	
Nema23 Stepper Motors	4	
MeanWell Power Supply	1	
Fully Assembled Pellet Extruder with wire harnesses WH1-WH5	1	See Fig. 120 above
2.1A Fuse	2	
Fuse holder	2	
Terminal Strip	2	
Assorted 14-16AWG Wire Spools	1	Red, black, green, blue, white,
Spade Crimp Connector Kit	1	
Wire stripping tool	1	
Wire Crimping tool	1	

#### Terminating/Crimping DUET3 side of extruder wire harness

**Fig. 141:**
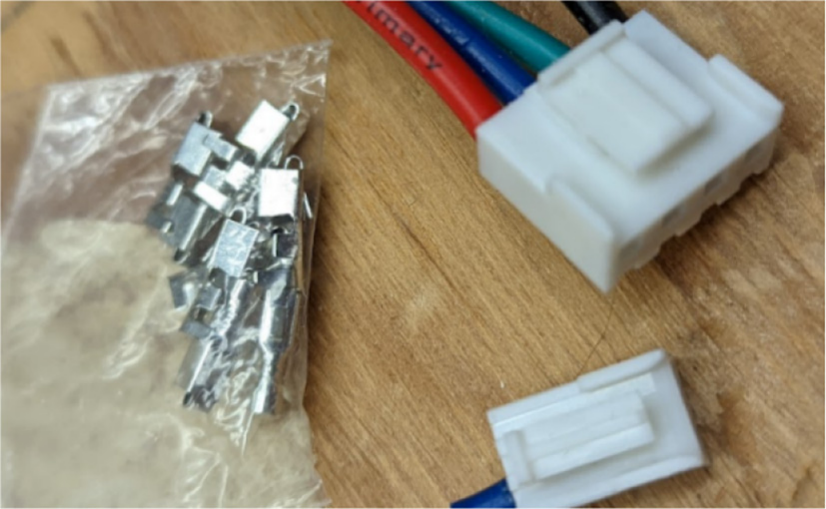
White Molex Connector with Larger Crimp Pins for Stepper Motors and Heaters.

**Fig. 142:**
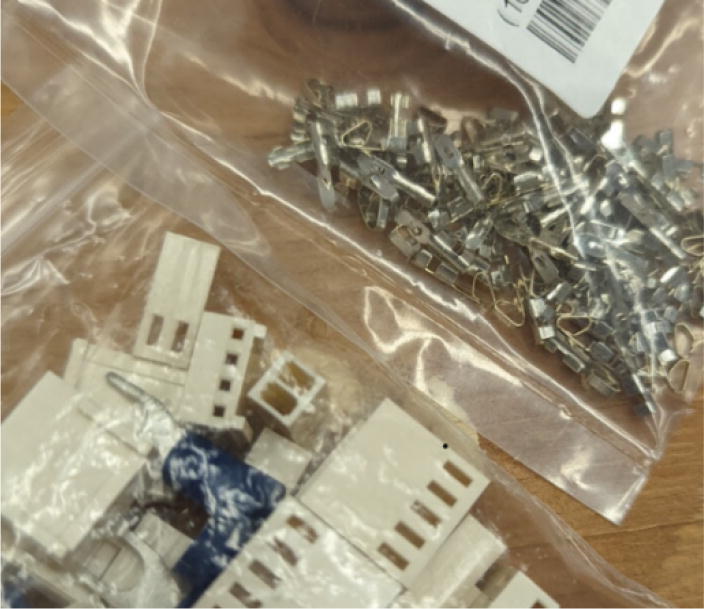
Beige Connector with Smaller Crimp Pins for Fans and Pt1000 Sensors.

To crimp the other side of wire harnesses WH1-WH5 with the exception of WH3 and WH4, use the included connectors and crimp pins included in the DUET3 Box. There are 2 sizes shown in Fig. 141 and Fig. 142.

#### Ribbon cable (WH1) for part cooling and heatsink fans

**Fig. 143:**
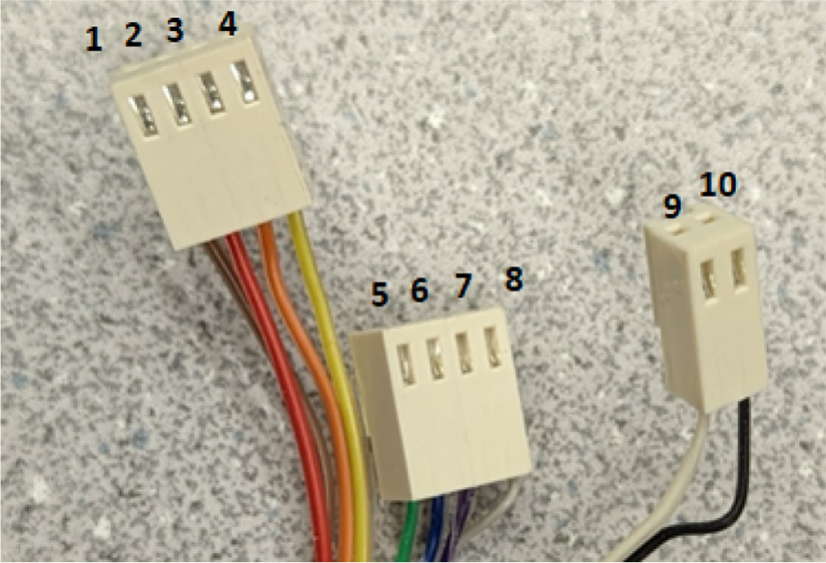
WH1 on DUET3 end.

On the other side of the ribbon cable, crimp and connect the beige 2 and 4 pin connectors provided from the DUET3 box as shown in Fig. 143.

#### Extruder motor (WH2) cables

**Fig. 144:**
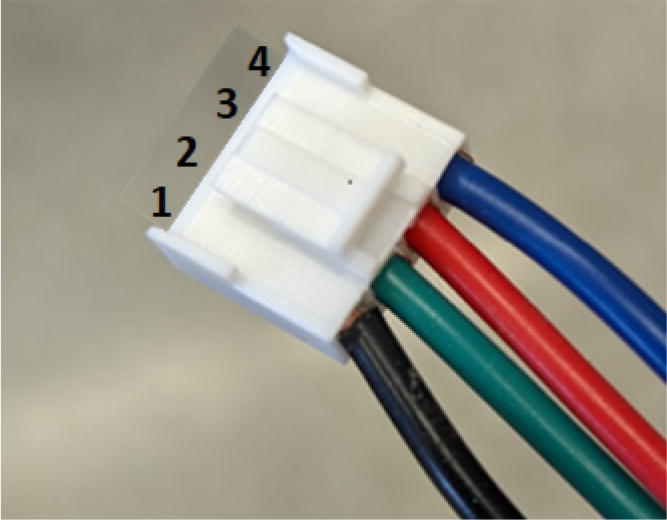
WH2 on DUET3 end.

On the other side of the extruder motor cable wire harness, crimp and connect the larger, white 4 pin connector provided from the DUET3 box as shown in Fig. 144.

#### Pt1000 sensor (WH5) cable

On the other side of the temperature sensor wire harness coming from the breakout board, crimp and connect two 2 beige pin connectors as shown in Fig. 175. These may also already come pre-crimped depending on supplier.

*Note: Ensure that the breakout board side connectors and DUET sides connectors of the wire harness are consistent with the order of wires for the fans and stepper motors. Order for temperature sensor wire harness does not matter.*

#### Connecting the extruder and hangprinter hardware to the DUET3 controller

**Fig. 145:**
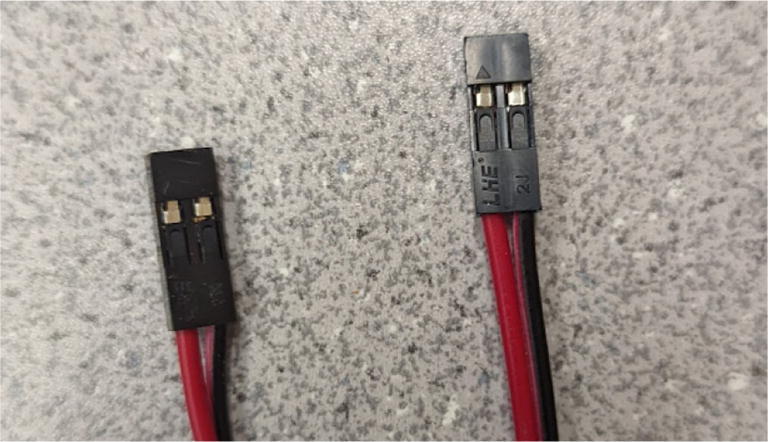
WH5 on DUET3 end.

**Fig. 146:**
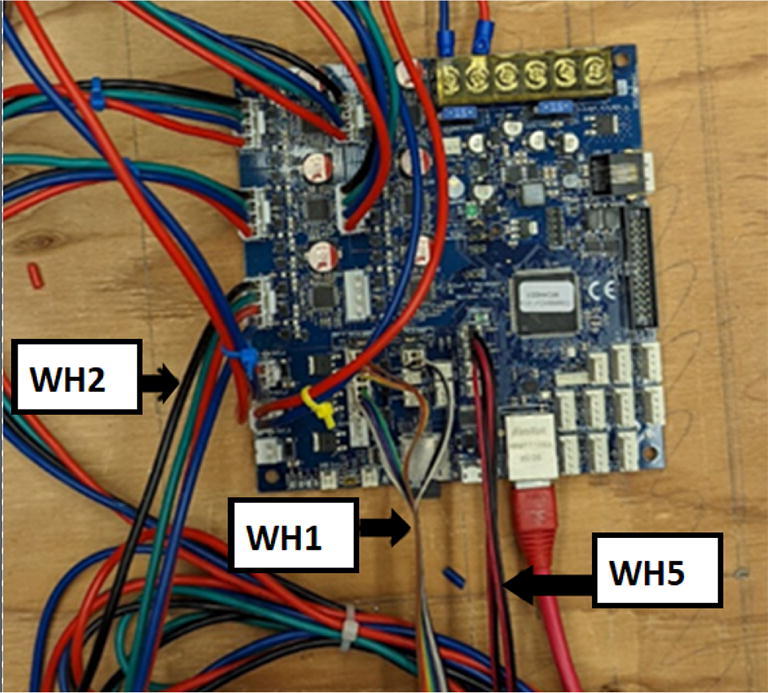
Wire Harness Connections to DUET3 from Breakout Board.

**Fig. 147:**
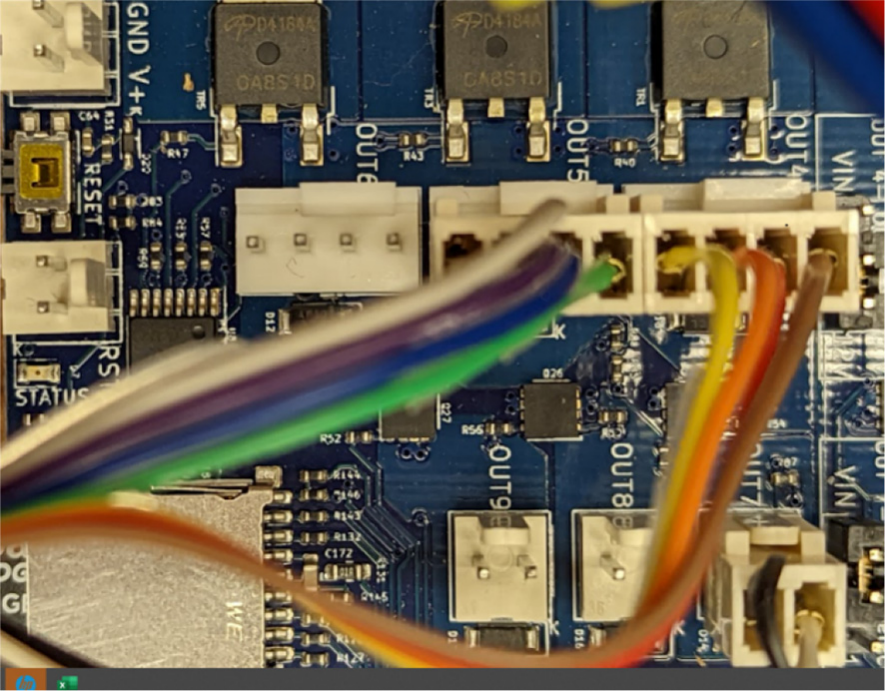
Rainbow Cable (WH1) Connections.

**Fig. 148:**
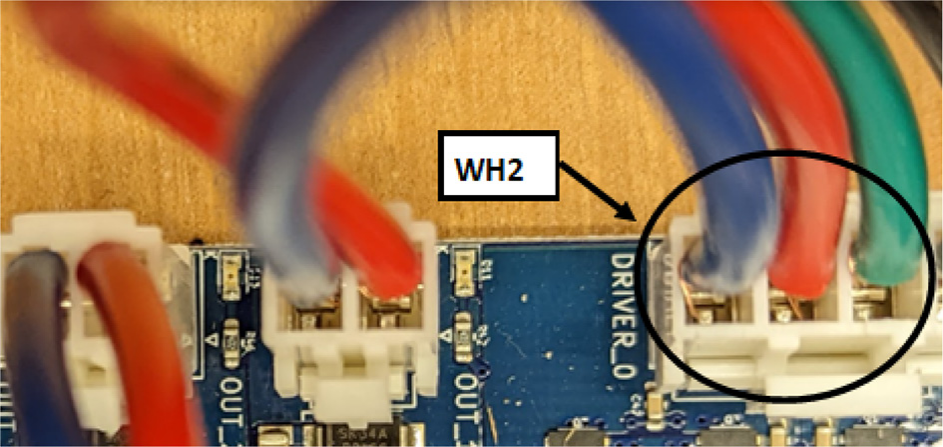
Extruder Motor Cable (WH2) Connections.

**Fig. 149:**
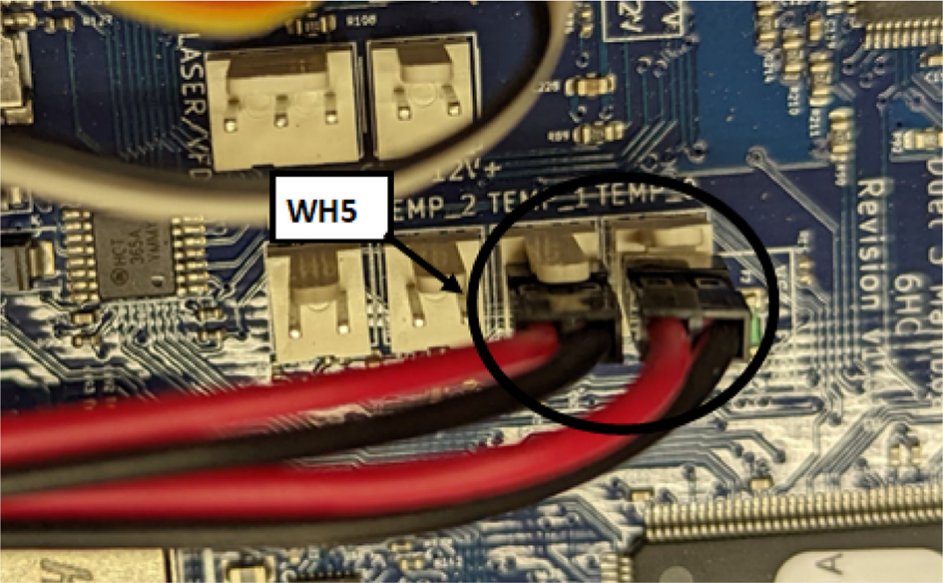
Pt1000 Cable (WH5) Connections.

Users should become familiar with the pinouts of the DUET3 Controller shown in [71]. Connect wire harnesses WH1, WH2 and WH5 according to Table 19 and Fig. 145, Fig. 146, Fig. 147, Fig. 148 and Fig. 149.

**Table 19:** Ribbon cable-DUET side connections.

**Wire Harness**	**DUET3 Connector Name (as it appears on the link provided above)**
Ribbon Cable (WH1) 4pos connector 1	OUT_0
Ribbon Cable (WH1) 4pos connector 2	OUT_1
Extruder Motor Cable (WH2)	DRIVER_0
Pt1000 Cable 1 (WH5)	TEMP0
Pt1000 Cable 2 (WH5)	TEMP1

#### Power supply, terminal strip and SSR/band heater wiring

**Fig. 150:**
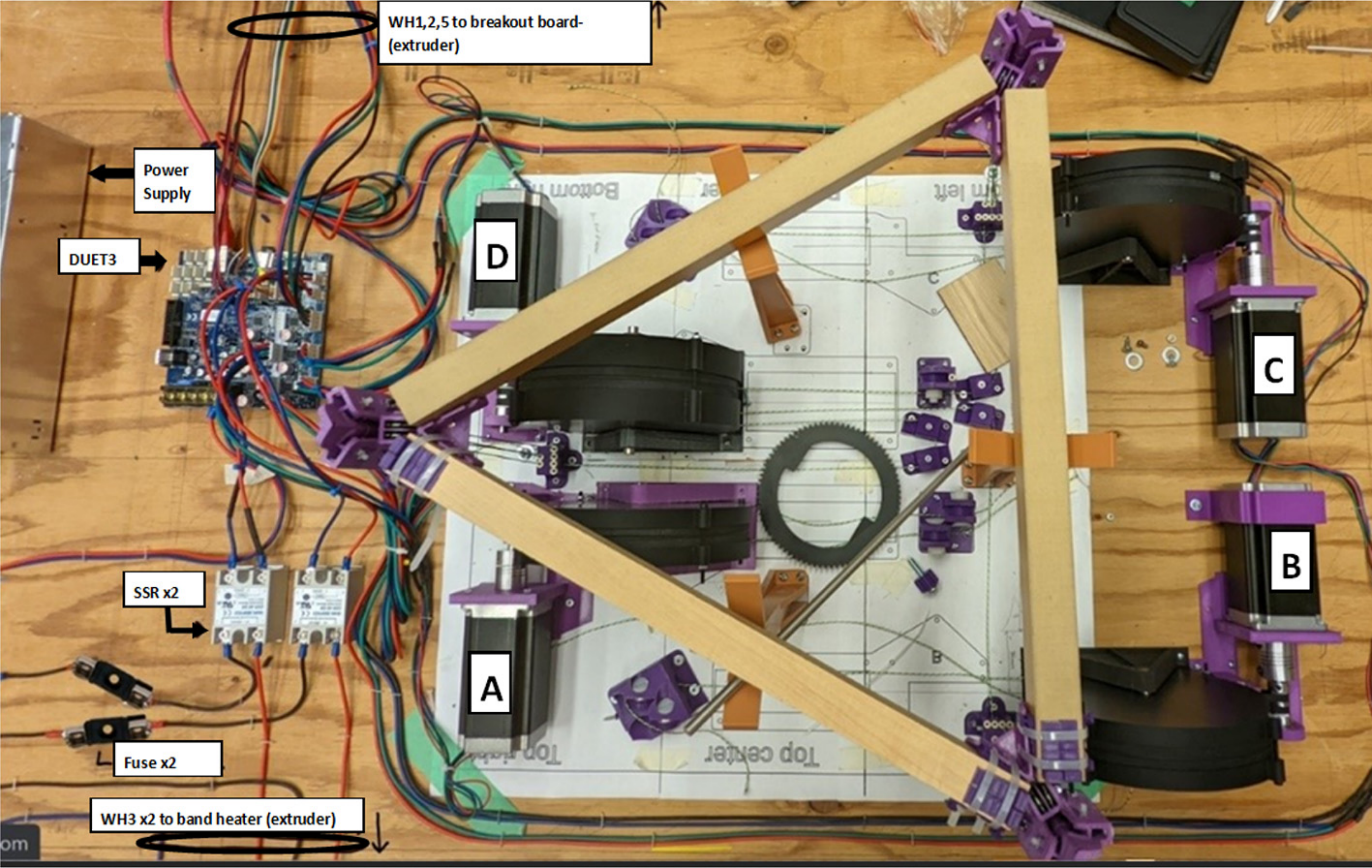
Layout of Hangprinter Electronics.

The overall layout of all electronics is shown in Fig. 150. As seen, there are 4 NEMA 23 stepper motors, 1 for each axis A through D. The stepper motor wires from each motor are routed to the DUET3 controller on the left. The wire harnesses WH1 through WH5 can be seen going from the DUET3 controller to the pellet extruder (not shown in picture). Two SSRs are connected to the DUET3 controller to drive the AC band heaters, which are connected to the opposite side of the SSR. One end of the relay is connected to a fuse to protect against overcurrent. The power supply can be seen on the left.

**Fig. 151:**
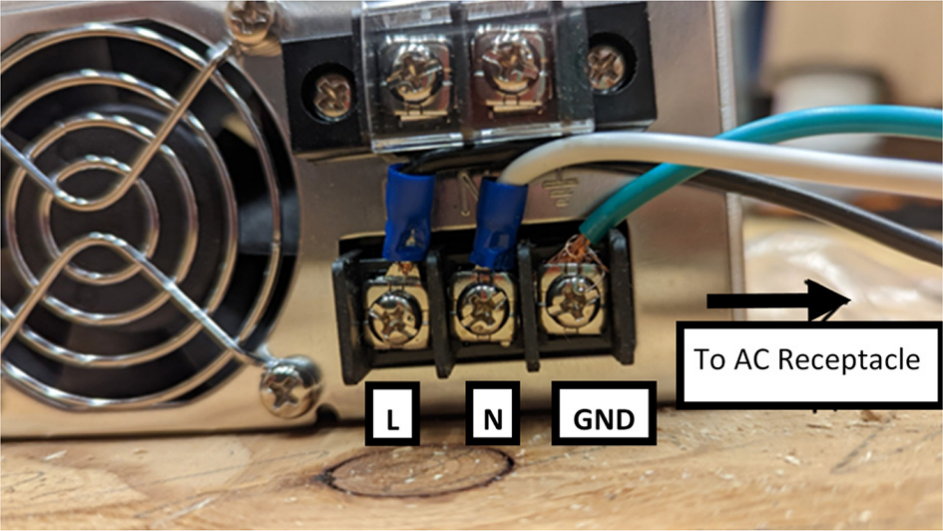
Main Connection to MEANWELL Power Supply.

The AC plug/wall cable will connect to the terminal block of the power supply as shown in Fig. 151.

**Fig. 152:**
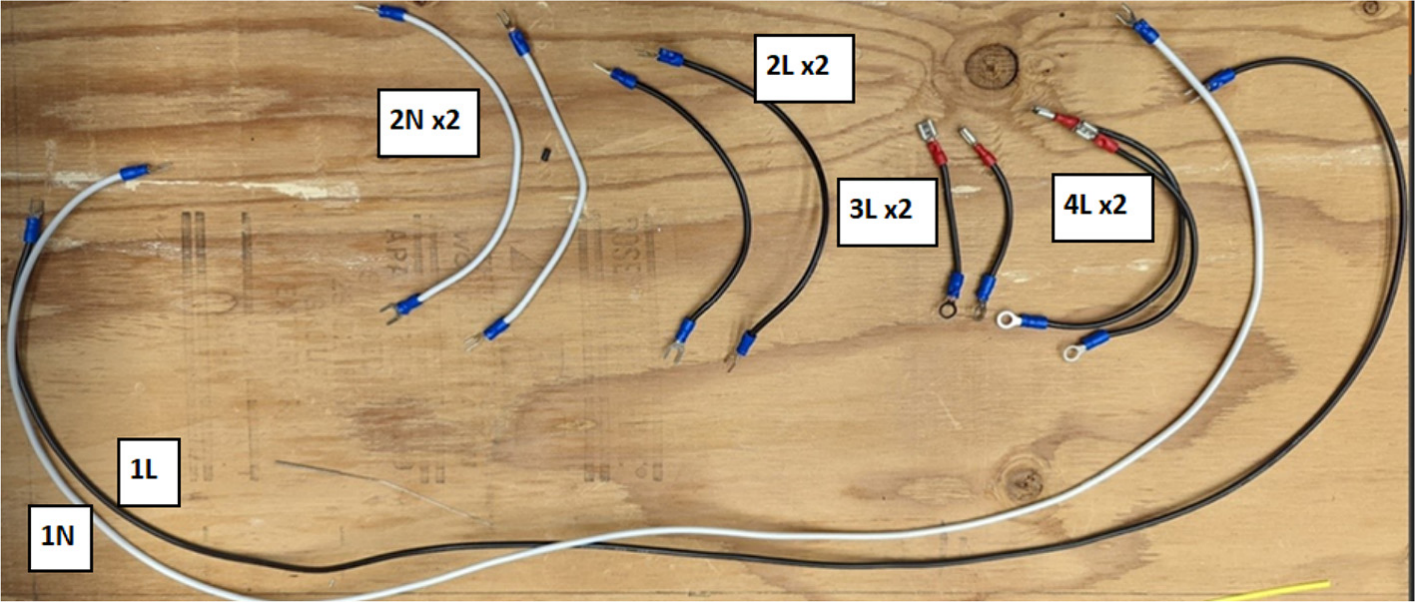
Power Supply Cables and Crimping.

To establish the AC side power supply and SSR to band heater connections, cut and crimp some 16AWG black and white wire to represent the line and neutral connections respectively shown in Fig. 152 and Table 20.

**Table 20:** AC power supply wire descriptions.

**Wire (L = Line, N = Neutral)**	**Qty**	**Approximate Length(ft)**	**Application**
1 N	1	4	Connect Neutral end of power supply to terminal block
1L	1	4	Connect line end of power supply to terminal block
2 N	2	0.25	Jumper 2 consecutive neutral terminals on terminal block
2L	2	0.25	Jumper 2 consecutive line terminals on terminal block
3L	2	0.08	Connects fuse holder to terminal block line
4L	2	0.15	Connects SSR to fuse holder

**Fig. 153:**
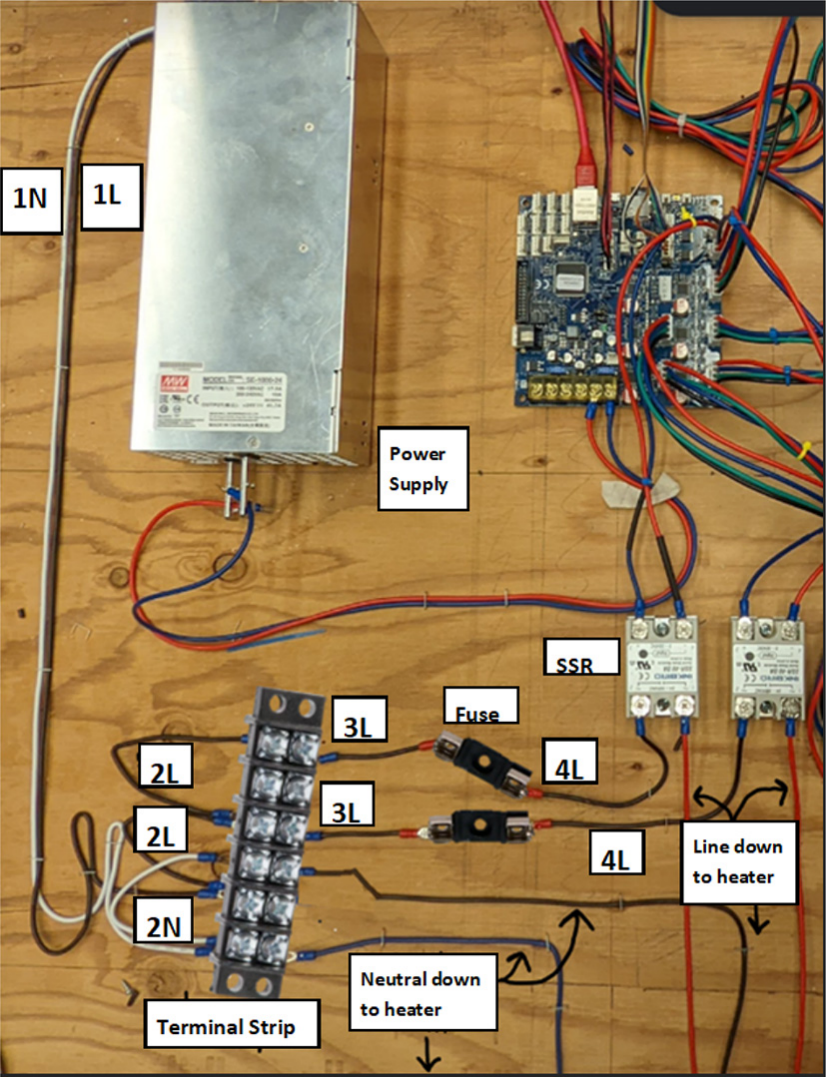
Wiring of Band Heater Circuit via SSR and AC Power Supply.

Establish the AC power supply connections between the relay, power supply and terminal block as shown in Fig. 153.

**Fig. 154:**
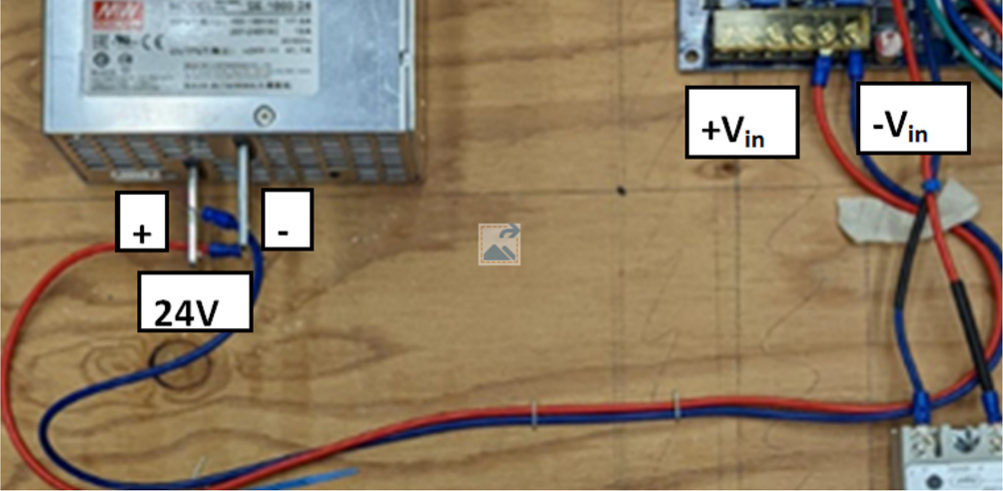
Connecting DC Power to VIN Terminals of DUET3 to Power Board and Components.

Next, wire the +24 V DC output of the power supply to the +/-VIN terminals of the DUET3 Board as shown in Fig. 154. Use approximately 3ft of 16AWG red and blue wire with spade connectors crimped on both ends of each wire.

**Fig. 155:**
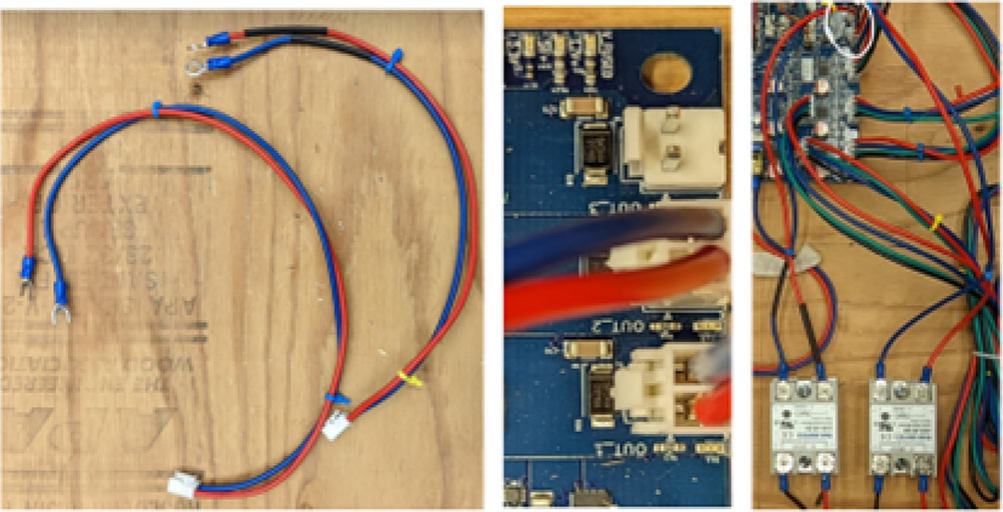
SSR to DUET Connection.

The above steps complete the power to the board and all its DC driven components as well as AC power to heaters controlled by SSRs. The next step is to hookup the SSRs to the DUET3s heater outputs and then from the SSRs to the band heater with AC lines. For this project, heater outputs OUT1 and OUT2 was chosen to drive the relay. Cut approximately 1–2 ft of blue and red 16AWG wire and crimp one end of both wires using a space crimp connector to connect to the relay side. Connect the other side of the red and blue wires using the DUET provided white, 2 pos connector. Refer to Fig. 155 and Table 21. Make note that the output signal to the SSR from the DUET has a polarity thus must be connected in proper order.

**Table 21:** Input side of SSR to DUET3 connections.

SSR #	SSR Inputs	Wire Color	DUET Port	DUET Port Pins
1	+	black/blue	OUT_1	V_FUSED
1	–	red	OUT_1	out1
2	+	black/blue	OUT_2	V_FUSED
2	–	red	OUT_2	out1

Note: All heater outputs (i.e., OUT1-OUt3) are active low, thus the PWM pin of the DUET is return point for current, thus it should be connected to the “-” lead of the SSR.

Next, connect the band heaters wire harness (WH3) to the AC output side of the relays. Follow the electrical schematic above for more detail. The black/blue wire of the band heater harness connects directly to neutral on the terminal block to the AC power source. The red wire connected to one end of the relay and the other is connected to the line connection on the terminal block via a 2A fuse block.

#### Connecting Hangprinter ABCD NEMA23 stepper motors to DUET3 controller

**Fig. 156:**
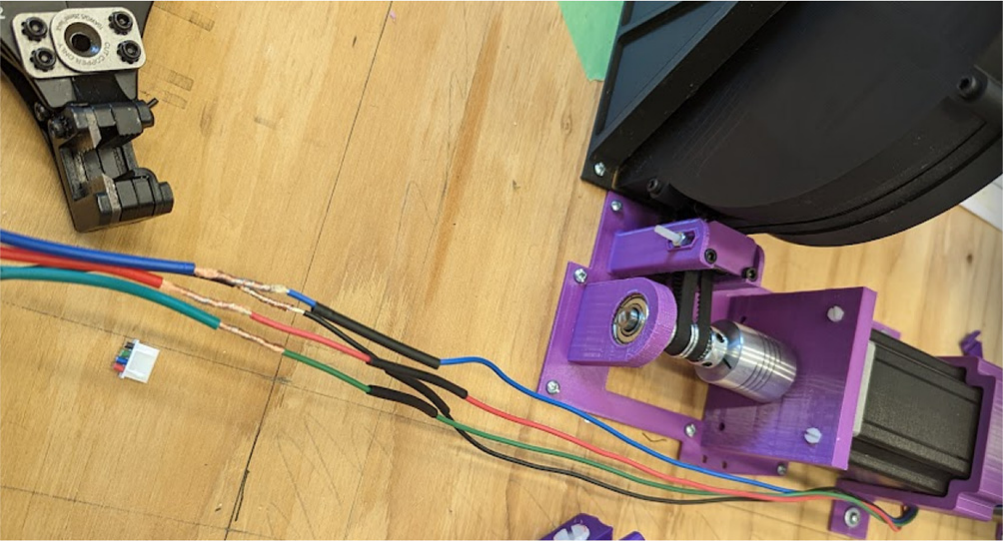
Stepper Motor Cable Extension.

The NEMA 23 stepper motors are used to drive the Hangprinter Chassis in the A, B, C, D local axis to produce movement in X, Y, Z cartesian coordinates. Their wires need to be extended using the 16-18AWG blue, red, green, and black wires and soldering them to their respective colored wire on the stepper motor like what is shown in Fig. 156. Use heat shrink tubing to cover the solder joints.

**Fig. 157:**
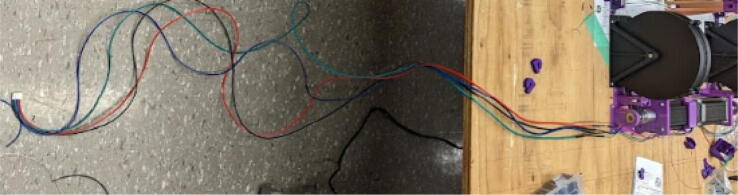
NEMA23 Extension.

To create the extension for the two stepper motors further back from the DUET3 controller, cut approximately 7-8ft of the 16AWG wire of black, blue, red, and green (Fig. 157).

**Fig. 158:**
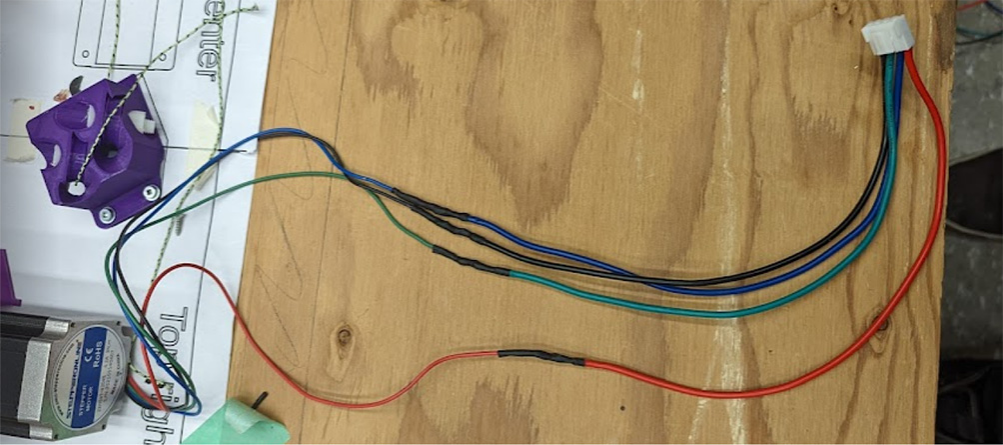
NEMA23 Extension 2.

To create the extension for the two stepper motors closer to the DUET3, cut approximately 1ft of blue, red, green, and black wire (Fig. 158).

**Fig. 159:**
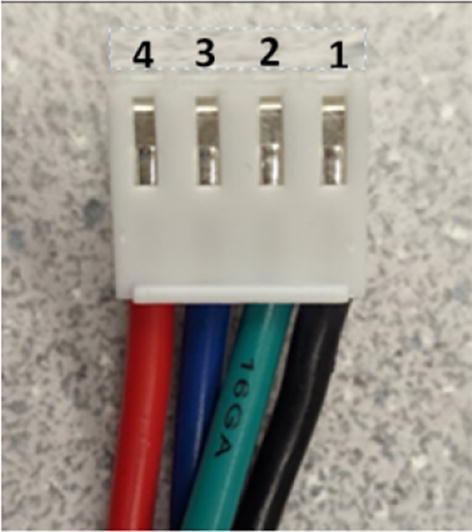
DUET3 Connecter Crimping-Stepper Motors.

Crimp the larger 4 pos, white connectors provided with the DUET for the 4 stepper motors as shown in Fig. 159. Note the color order and orientation.

Refer to Table 22 for order of wires on connectors.

**Table 22:** Stepper motor coil/color wires to corresponding connector positions.

**Stepper Motor Coil**	**Wire Color**	**Connector Position**
A+	black	1
A-	green	2
B+	blue	3
B-	red	4

NOTE: Stepper motor wire color may vary. To identify coil pairs, touch different pairs of wires until motor becomes hard to spin. Caution: Mixing the phases up on the 4-pin connector can result in damage to the stepper driver.

**Fig. 160:**
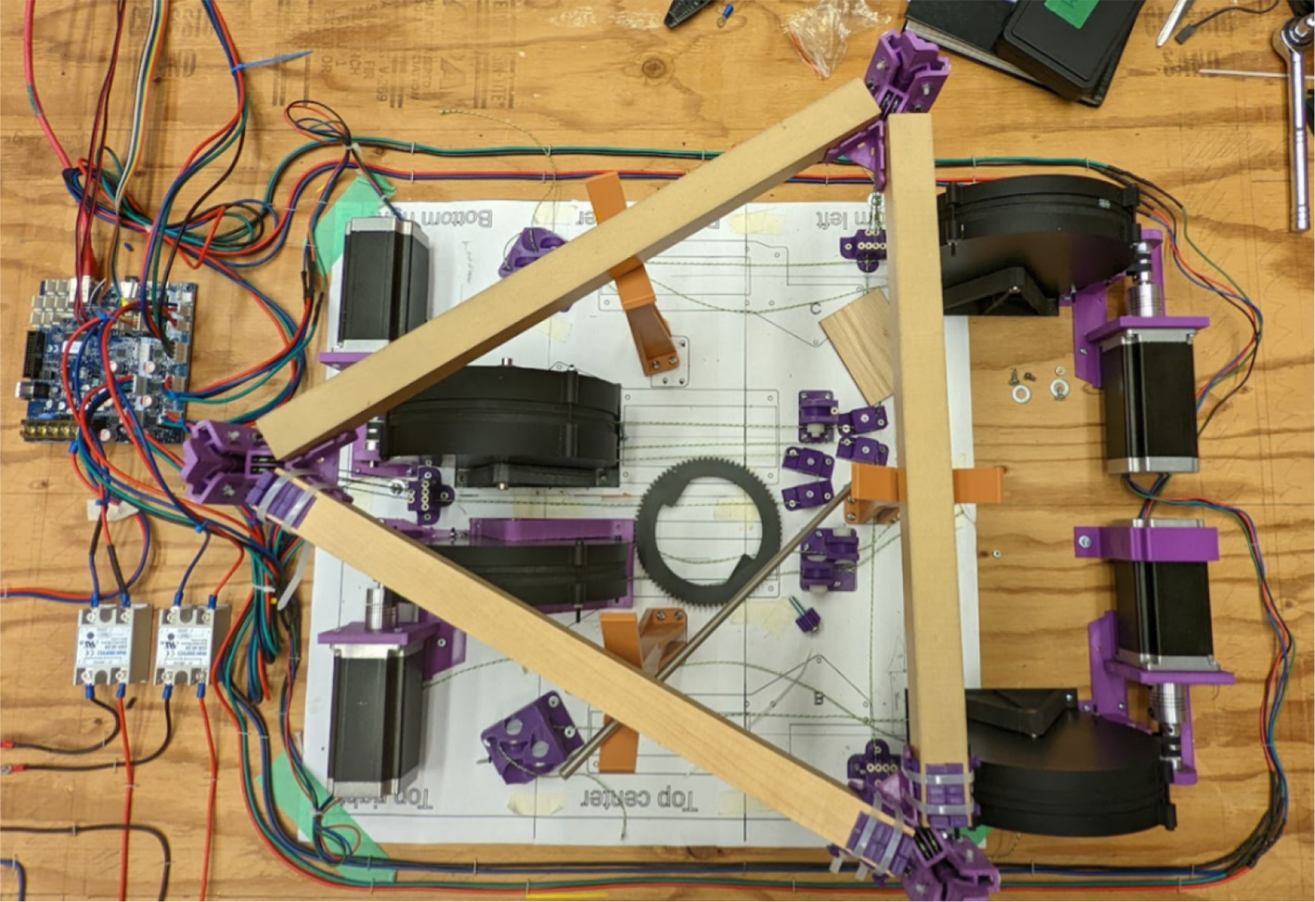
NEMA 23 Motor Wiring Layout.

**Fig. 161:**
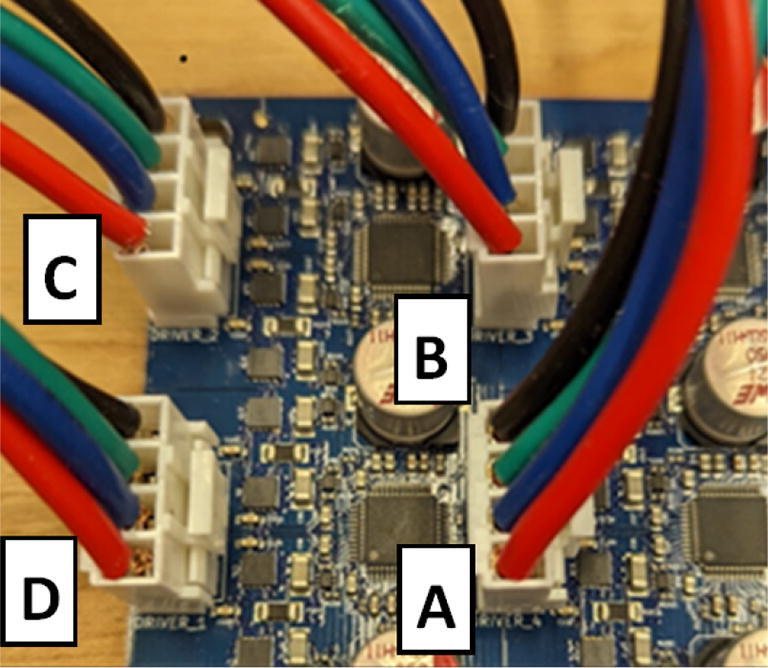
ABCD Motor Connections on DUET3.

Finally, route and connect the ABCD axis stepper motors to the DUET controller according to Fig. 160, Fig. 161 and Table 23.

**Fig. 162:**
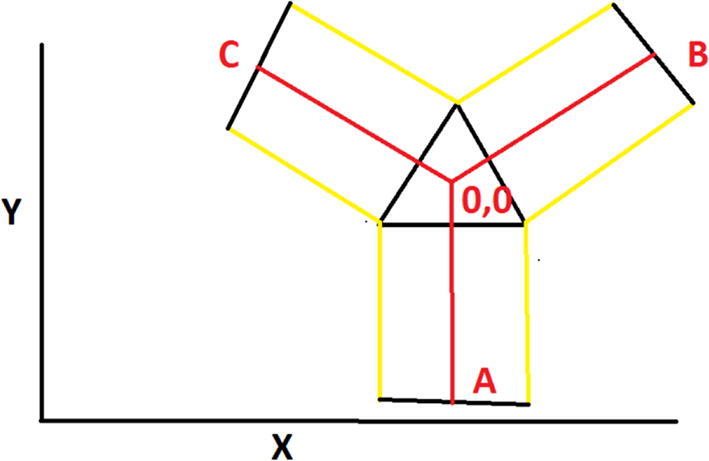
Anchors.

**Table 23:** A, B, C, D axis assignments to DUET3 stepper driver ports.

Motor/Axis	DUET3 Port
A	DRIVER_4
B	DRIVER_3
C	DRIVER_2
D	DRIVER_1
Extruder motor	DRIVER_0

By performing the above steps, the wiring of all ceiling mount components and connections between the pellet extruder and DUET3 should be complete.

## Operation instructions

Once the Hangprinter hybrid machine and its systems are assembled, the next step is to set up the hardware and software to enable printing. The following sections cover:

1. Setting up DUET3 controller and web interface.
2. Updating firmware on DUET3.
3. Configuration file set up.
4. Extruder and feeding system setup.
5. Hangprinter calibration.
6. Using the machine.
7. Cleaning and maintenance.
8. Safety.

### DUET3 mainboard setup, ethernet connection, web control interface (DWC) and firmware update

NOTE: The DUET3 controller is an ethernet only device - users will need a “crossing” ethernet cable.

There are multiple ways to establish DUET3 ethernet communication with a designated computer. The user is directed to [72], [73] setup connection between your DUET3 and computer. In the latter link, the user can opt to setup an ethernet connection directly to their computer or via a router.

The “Duet Web Control” is the web interface that is used to control functionality of the DUET3 board and to upload print jobs. While basic use of it is described further below in this document, the user is recommended to visit [74]. An official, complete list of G-code commands for the Duet 3 can be found here: [75].

Depending on the current firmware on the DUET3 board, the method of updating the DUET3 boards firmware may change. Thus, the user is directed here to follow instructions on how to update their firmware to the latest version, [76]. Please ensure that you only upgrade to the newest, most stable version, which at the time of publication of this document, is release 3.3 or onwards.

### Preparing the DUET3 configuration file

The DUET3 controller that is used in this project runs on a C based firmware called RepRap Firmware (RRF). This firmware accepts G-code commands from CAM programs (i.e., Slicers) or through terminal programs which then produces the proper response based on the G-code. Native support of the Hangprinter is supported in > RRF 1.2 thus for this project, no modifications in its firmware source code must be made. The “config.g” file, which contains G-code commands for the initialization of various setting and parameters, must be created. Only the basic settings used for this project are shown here and links to more detailed documentation regarding G-code on the DUET3 website are referenced.

#### Config.g file overview

1. Networking information

Networking comes disabled by default and must be enabled first through a serial communication program like YAT or PuTTY (see section above). Once the IP address has been set, enter it into your browser to access the DWC web interface. Click on the “System” button on the left tool bar. Then click on the “config.g” file in the file directory. The user can choose to edit the file directly in the DWC built in editor or choose to upload a new file to the SD card of the DUET3 by clicking the “upload” button at the top right of the file directory in the DWC. The YAT terminal program is only required once to initially set a temporary IP address to access the DWC web interface in your browser. Open the config.g file and erase all previous code as it is not needed. Enter the following lines of code at the top of the file:

; Enable network
M552 P192.168.50.2 S1
M550 P“hangprinter”; change the name from the IP address of 192.168.50.2

This is the minimum requirement to get Networking configured in the DUET3 board. For more information, visit [73].

2. General printer settings

General Machine Settings/Conventions

M699 K6; Configures the Duet 3 to identify as a Hangprinter

G21;work in mm

G90;absolute coordinates

M83;relative extruder moves

3. Heater and temperature sensor settings

There is one band heater used along with a pt1000 RTD. Another pt1000 RTD is used to monitor temperatures inside the Aluminum heatsink block but that is not attached to any heater.

; Heaters and Temp Sensor

M140 H-1; disable heated bed (overrides default heater mapping)

M308 S1 P“temp1″ Y”pt1000″ A“Extruder Sensor” configure sensor 1 as PT1000 on pin temp1

M950 H1 C“out1″ Q10 T1; create nozzle heater output on out1 and map it to sensor 1, limit pwm frequency to 10 Hz

M307 H1 B0 S1.00; disable bang-bang mode for heater and set PWM limit

M143 H1 S240; set temperature limit for heater 0 to 240C

308 S2 P“temp2″ Y”pt1000″ A“Barrel Sensor”;configure sensor 2 as PT1000 on pin temp2

*Note: Be sure to adjust the PWM frequency of the heater outputs. The SSRs used in this project are zero-crossing type thus, have longer cycle times. Use a PWM frequency of < 10 Hz.

4. Cooling fan settings

The pellet extruder is equipped with 2 heatsink cooling fans and a part cooling fan. The heatsink cooling fans are 4 wire axial fans while the part cooling fan is a 2-wire blower style fan. The cooling fan is for part cooling as is assigned by default, to fan0 in the firmware. This is because most slicer programs also treat fan0 as the part cooling fan. The 3-pin jumpers above each bank of fan connectors (OUT4 to OUT6, and OUT7 to OUT9) select the voltage for each bank of fans, of either VIN (10A fuse) or 12 V (800 mA, supplied by onboard 12 V regulator. You can supply a different voltage to the center pin of the 3-pin jumper, pin VOUTLCx, to run different voltages. For this project, the power to the fan will be supplied by the onboard 12 V regulator.

Fans are first defined and configured by **M950**, then operated by **M106**.The part cooling fan will be controlled by G-code command generated from the slicer to determine when cooling is needed for a particular layer.

M950 F0 C“out7″ Q500; create fan 0 on pin out7 and set its frequency

M106 P0 S0 H-1; set fan 0 value. Thermostatic control is turned off

5. Heatsink fan settings

The heatsink fan will be a thermostatically controller fan. This means that it will turn on and off as it needs to in order to prevent heat creep from occurring. Settings are shown below:

M950 F1 C“out4″ Q500; create fan 1 on pin out4 and set its frequency

M106 P1 S0 H-0; set fan 1 value. Thermostatic control is turned on

6. Extruder motor settings

Although the pellet extruder is not fed a filament, an “FDM equivalent” set of parameters is needed. The feed rate in RRF is capped at 9000 mm/min thus this is what the feed rate setting should be set at. The following equation (Equation (9)) gives the RPM of the motor (i.e., converts rpm to mm/s). It was found that using 224 steps/mm provided the best extrusion characteristics. This value however can be changed accordingly through physical experimentation.

(9) RPM=F(mm/min)∗E(step/mm)/3200(step/revolution)

M569 P0 S1;set driver0 to be extruder motor driver and drive CCW to extruder pellets down auger

M584 E0;map drive0 to extruder0

M203 E9000;set “FDM equivalent” feed rate to max. 9000 mm/min

M92 E224.6;steps/mm

7. Hangprinter kinematics and stepper motor settings

This project uses stock RRF firmware found directly from DUET3′s website. Stock firmware is used due to its official support by DUET3 and is well maintained. The configuration below are some basic settings in order to get the motors and their axes operational. Shown below in Fig. 162 is the layout and anchor distances that were used in this project. Note that the DUET3 RRF firmware uses a global co-ordinate system and the (0,0) coordinate is directly below the D anchor as shown in Fig. 193. This is set in firmware.

For this project, the Z position of the D anchor is the distance of the ceiling mount of the Hangprinter to the ground. For this project, Z = 2800 mm. By default, the X and Y coordinates of D anchor are set as (0,0), thus, the D anchor is located at (0,0,2800). The A anchor is 2438 mm from the D anchor (0 mm, −2438 mm, 10 mm). The B anchor is (2111.72 mm,1219.20 mm,10 mm). The C anchor is (-2111.72 mm, 1219.20 mm,10 mm). If you are using different anchor positions as determined in.

; Drives A,B,C,D Axis

M569 P0.0 S0; physical drive 0.0 goes forwards

M569 P0.1 S1; physical drive 0.1 goes forwards

M569 P0.2 S0; physical drive 0.2 goes forwards

M569 P0.3 S1; physical drive 0.3 goes forwards

M569 P0.4 S1; physical drive 0.4 goes forwards

M584 X0.0 Y0.1 Z0.2 E0.3 U0.4; set drive mapping

M669 K6 A0.0:-2438.0:0.0 B2111.72:1219.20:0.0C-1732.0:1000.0:0.0 D2000.0 P1000.0

M350 X16 Y16 Z16 E16:16 I1; configure microstepping with interpolation

M92 X80.00 Y80.00 Z80.00 E420.00 U80.00; set steps per mm

M566 X900.00 Y900.00 Z900.00 E120.00 U900.00; set maximum instantaneous speed changes (mm/min)

M203 X6000.00 Y6000.00 Z6000.00 E1200.00 U6000.00; set maximum speeds (mm/min)

M201 X500.00 Y500.00 Z500.00 E250.00 U500.00; set accelerations (mm/s^2)

M906 X1000 Y1000 Z1000 E800 U1000 I60; set motor currents (mA) and motor idle factor in per cent

M84 S30; Set idle timeout

Axis Limits

M208 Z-5 S1; set axis minima

M208 Z2000 S0; set axis maxima

8. Miscellaneous settings

Define a tool in the firmware. Doing so will generate a “tool” in the DWC interface from which heaters, fans, etc. can be controlled directly from the interface.

; Tool Definitions

M563 P1 S“Pellet Extruder” D0(extruderdrive) H1 F0:!(fans);assign extruder(T1), drive(E1),Heater(h1) to tool1

When the config file is done, upload it to the DUET3 SD card via DWC by clicking the “upload” button in the code editor on the DWC. The hybrid Hangprinter should now be ready to print. To upload a print job to the DUET controller, simply click on the system tab in the left-hand tool bar and click on upload files and upload to the appropriate file directory. For more details on Hangprinter configuration, visit [77].

Shown below are some common G code commands used in this project in Table 24 and Table 25. It is also recommended to refer to the G-code dictionary [75]**.**

**Table 24:** Common g- and m-commands for Hangprinter.

**Command**	**Description**
G91(relative)/G90(absolute)	Enter absolute or relative mode -printing usually done in absolute mode -individual motor movement and homing usually done in relative mode
G1 H2 Xx Yy Zz Uu Ff (linear advance)	**-**override hangprinter kinematics and individually move each anchor motor by x,y,z,u mm at feedrate f mm/min -X → A axis motor -Y → B axis motor -Z → C axis motor-Useful when lines need to be tightened individually and during calibration and homing (in G91 mode)
G1 Xx Yy Zz Ff (linear advance)	**-**use Hangprinter kinematics to move × mm, y mm and z mm in cartesian space at feedrate of mm/min -used for print moves in relative or absolute mode
G1 Ee Ff	-extrude e mm of plastic at feedrate f mm/min -extruder moves always relative specified by M83
G92 X0 Y0 Z0	-set home position of Hangprinter when nozzle is at center of print bed
M92 e0:e1	-adjust e-steps/mm of extruder(e0) and feeding system(e1)
M567 EX:Y	-adjust mixing ratio of feeding system release to extrusion as a % of total extrusion. Keep extruder % as 1 and feeder as a % of the extruder rate and needed to achieve consistent pellet feed

**Table 25:** G1 travel examples.

**Command**	**Behavior (orange arrows mark direction of movement)**	**Description**
G91 G1 X- 10 F2000		-Linear advance move in negative X direction by 10 mm @2000 mm/min via Hangprinter kinematics (relative mode) -To move in opposite direction, use G1 X10 F2000 -Similar behavior for movement in Y and Z directions -can combine with Y and Z movements
G90 G1 X- 10 F2000		-Linear advance move to X coordinate (-10,0,0) @2000 mm/min via Hangprinter kinematics (absolute mode)-To move in opposite coordinate (10,0,0) , use G1 X10 F2000 -Similar behavior for movement in Y and Z directions -can combine with Y and Z movements

### Hangprinter parameters and calibration

**Fig. 163:**
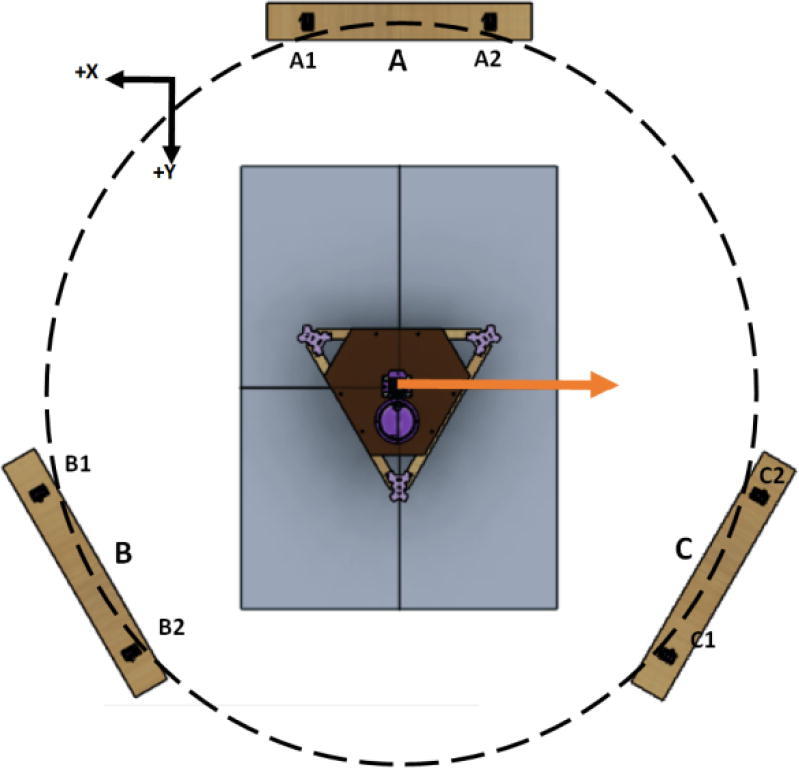
Hangprinter coordinates and anchor norms.

This section covers the process of positioning and grounding the anchors, measuring, and entering all physical parameters into firmware to calibrate the Hangprinter. With the lines routed, the anchors can now be fixed to the ground. As mentioned before, it is up to the user how far they want to position the anchor if the following conditions are met:

1. The anchor beam is parallel to the corresponding edge of the triangle end effector chassis.
2. The lines between the anchor and triangular chassis are perpendicular to the edge of the triangle.
3. There is sufficient line length.
4. Coordinates and sign conventions are according to Fig. 163.

**Fig. 164:**
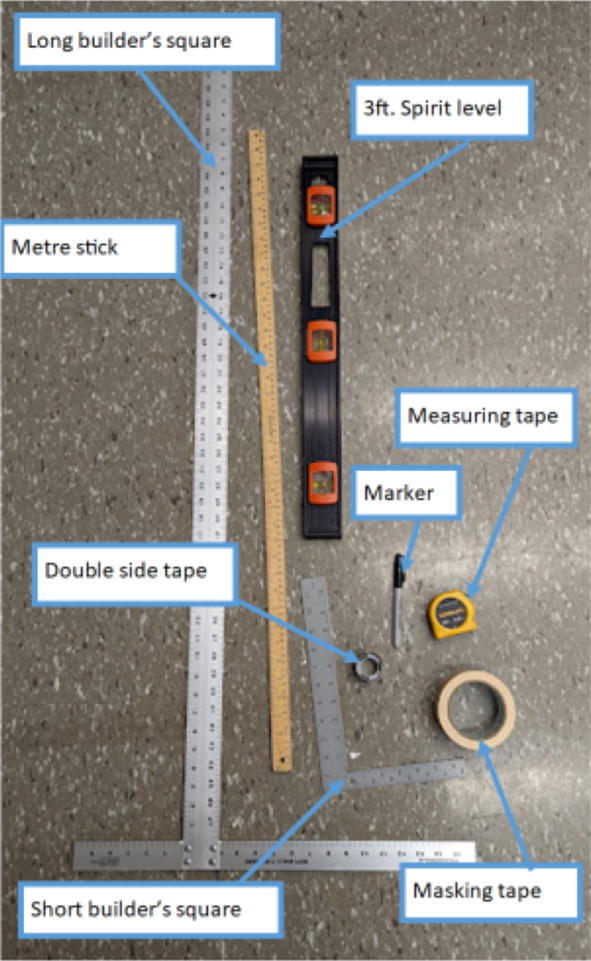
Tools for Hangprinter Calibration.

Tools needed for this section are shown below Fig. 164.

**Fig. 165:**
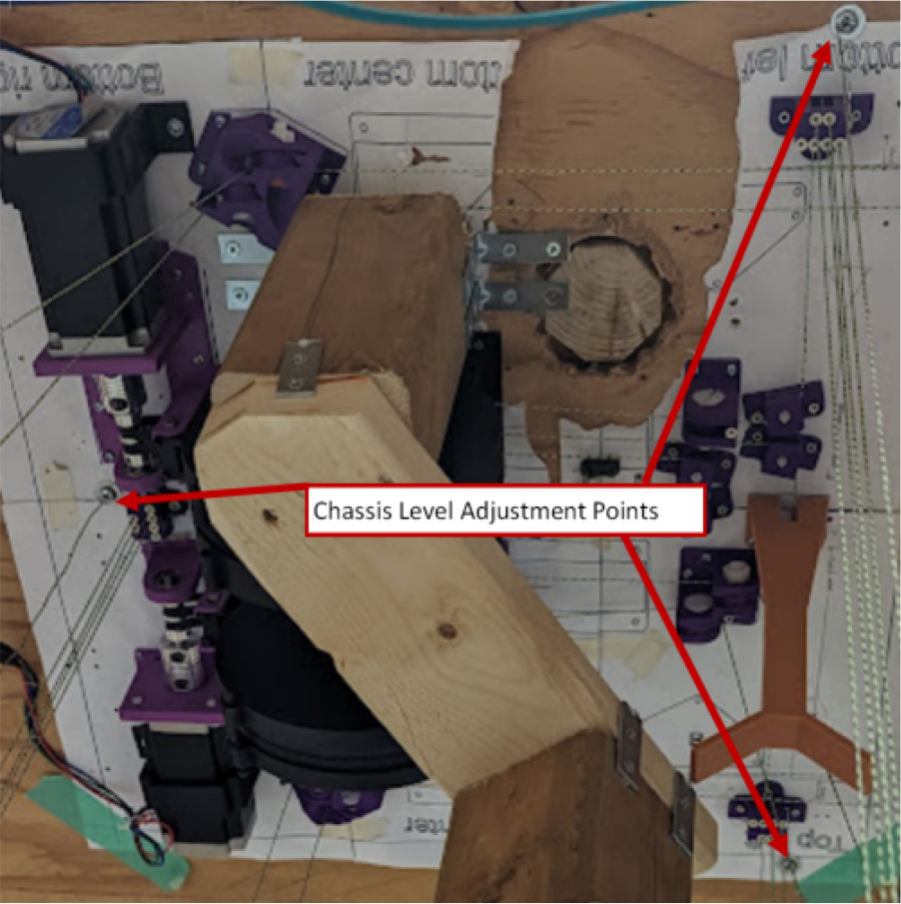
Chassis Level Adjustment Locations.

**Fig. 166:**
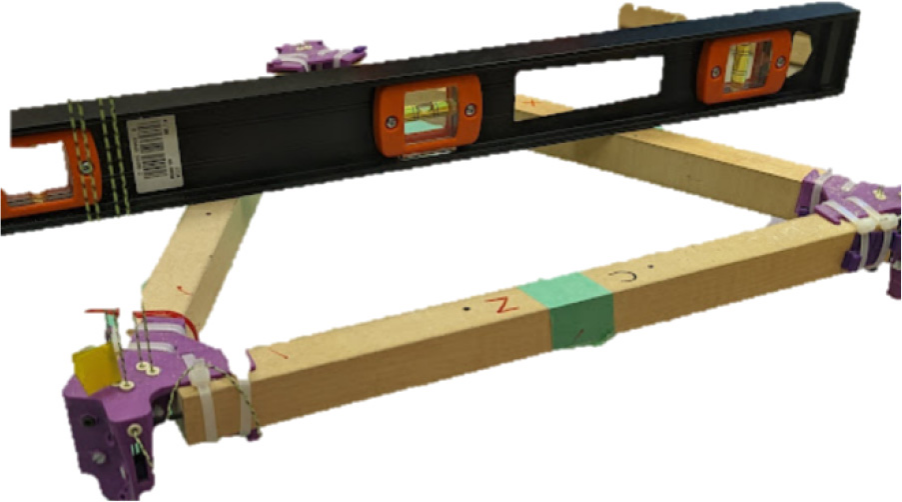
Adjusting Level of the Chassis.

Positioning print bed and grounding the anchors:

1. Remove the pellet extruder from the chassis if it is currently mounted.
2. Level the Hangprinter chassis by adjusting the length of line coming out from the “line verticalizer” by pulling or releasing the line terminated via bolt and washer on the ceiling mount. This is illustrated in Fig. 165 and Fig. 166. Once the proper level is reached, tighten the washer and screw with an impact driver to ensure the line will not move from position over time. Ensure that all three sides of the chassis are level using a spirit level across to edges of the triangle near their vertex as shown in Fig. 167. Do this for all 3 corners/pairs of edges of the chassis.

**Fig. 167:**
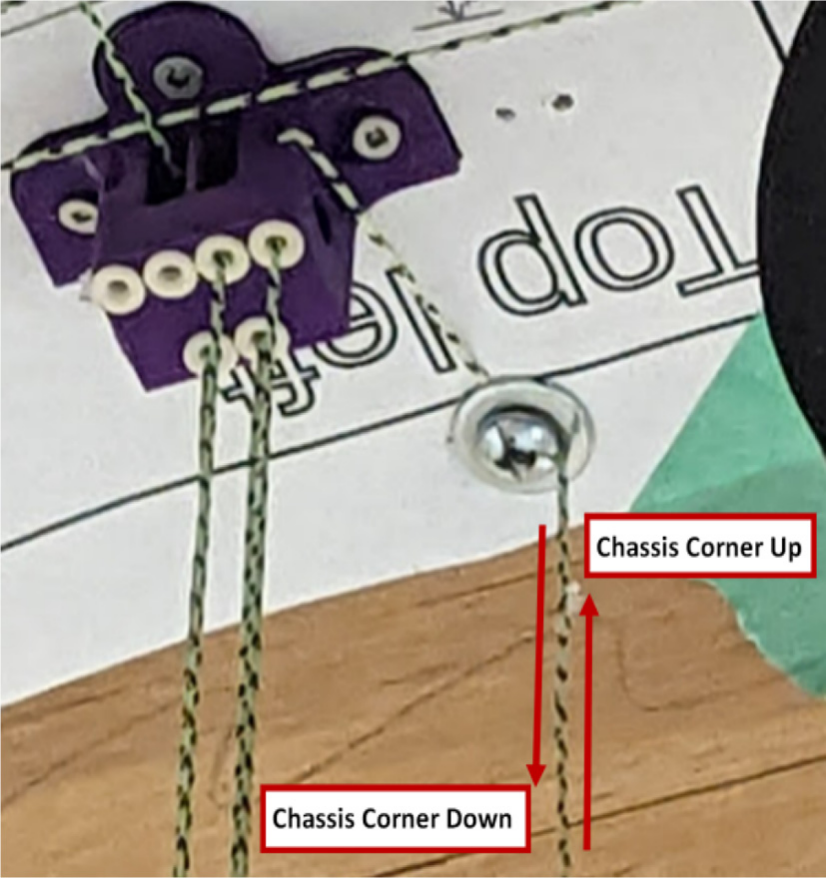
Checking the Level of the Chassis.
3. Ensure all A, B, C and D anchor lines are slack with the chassis approximately 1ft above the ground and in its natural resting position (Fig. 168).

**Fig. 168:**
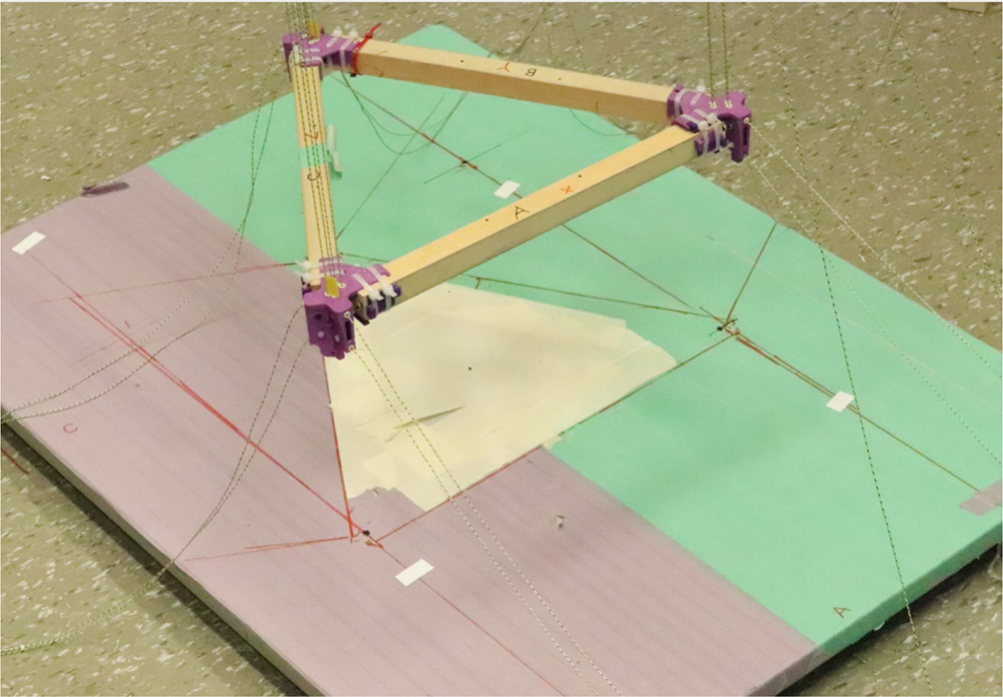
Chassis approximately 1ft above print bed and cables loose.
a) In relative mode (G91) slack lines using “G1 H2 Xx Yy Zz Uu Ff” where x,y,z,u is the amount of line to release for the A,B,C,D lines respectively (recommended to do increments of 100 mm per G1 command for ABC and 10 mm for D). Adjust feed rate, f, as needed (safe range between 1000 mm/min < f < 6000 mm/min).
b) Slack the lines enough so that anchor points can reach their defined position without causing tension on any of the Hangprinter components.
4. On the print bed, mark its center point and position the print bed under the chassis so it’s center point lines up roughly with the centroid formed by the triangular chassis as shown in Fig. 169. Ensure that one pair of edges of the rectangular print bed is parallel with the “A line” edge of the chassis. This is not necessary but makes measurements easier.

**Fig. 169:**
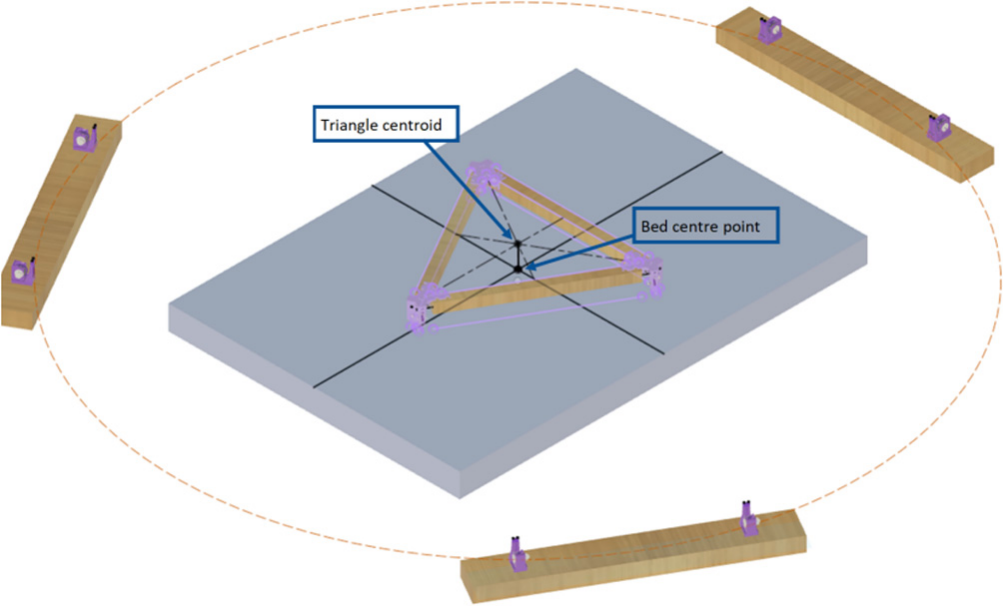
Centering the Chassis on the Print Bed.
5. With the chassis in its natural resting (no oscillations) position with ABC lines slack, slowly lower the chassis (with G91 G1 H2 Uu) so it touches the print bed. Put weights on the chassis so that it will not move from its position during the calibration process (Fig. 170). *Note: If at any time the chassis or print bed moves, the whole process must be started again from step 1.*

**Fig. 170:**
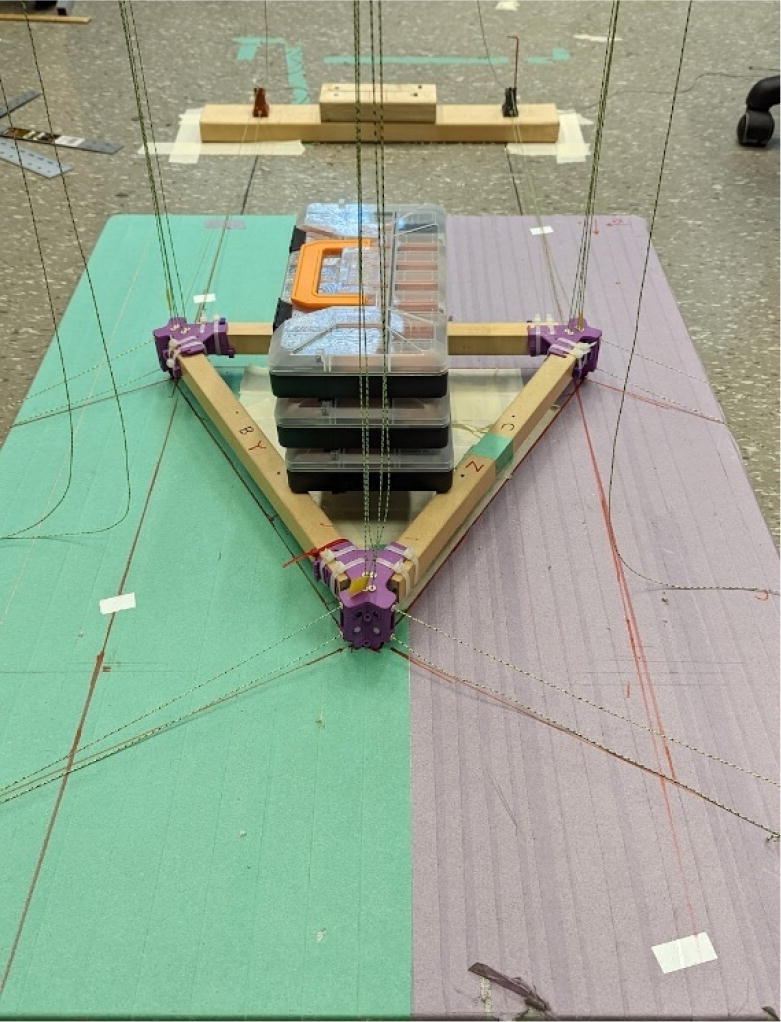
Chassis in resting position with weights and cables loosened.
6. With a marker, trace a perimeter around the triangle and mark the corners as shown in Fig. 171. Using a builder’s square, draw perpendicular lines on each corner of the drawn triangle (colinear with the lines coming out of each corner clamp) as shown in Fig. 172. Extend these perpendicular lines to the largest length possible with the square.
  a. If drawn correctly, these perpendicular lines will be used as visual indicators to ensure lines are perpendicular to both anchor beams and the chassis triangle.
  b. Extend these lines past the Hangprinter chassis as shown in Fig. 173. These perpendicular lines serve as the reference point against which the X and Y components of the B and C anchors are measured.

**Fig. 173:**
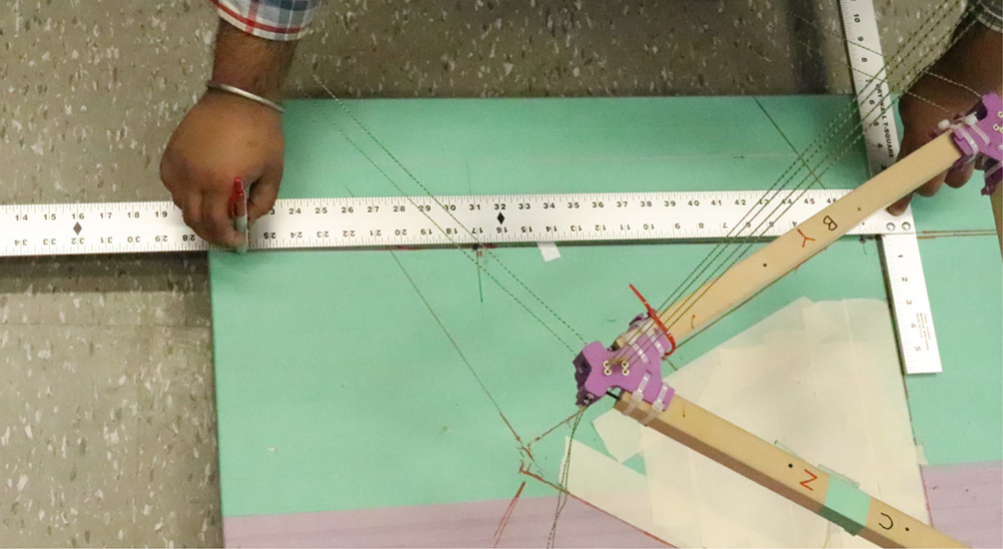
Extending the projected A lines past the Hangprinter chassis.

**Fig. 171:**
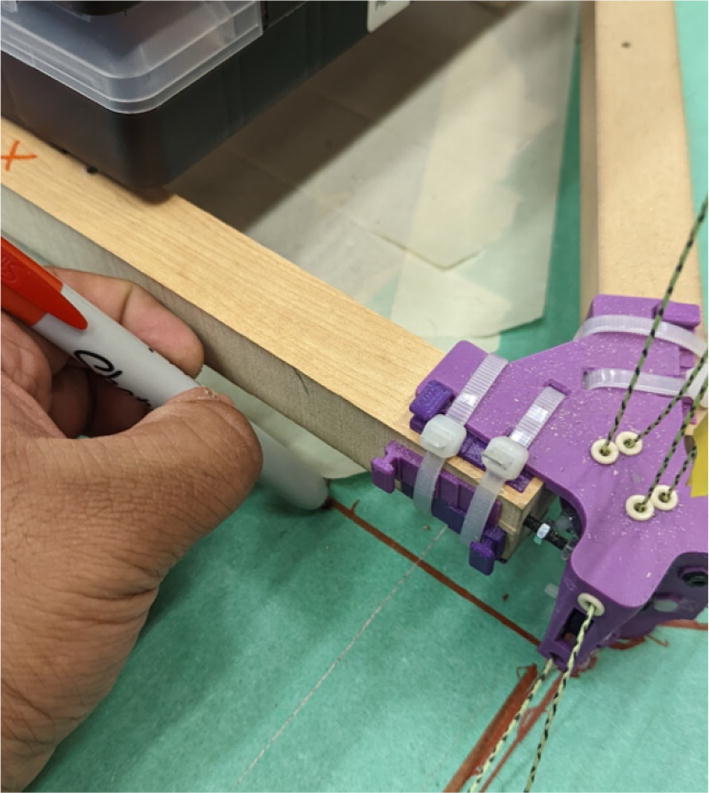
Drawing projection of chassis on print bed to use for calibration.

**Fig. 172:**
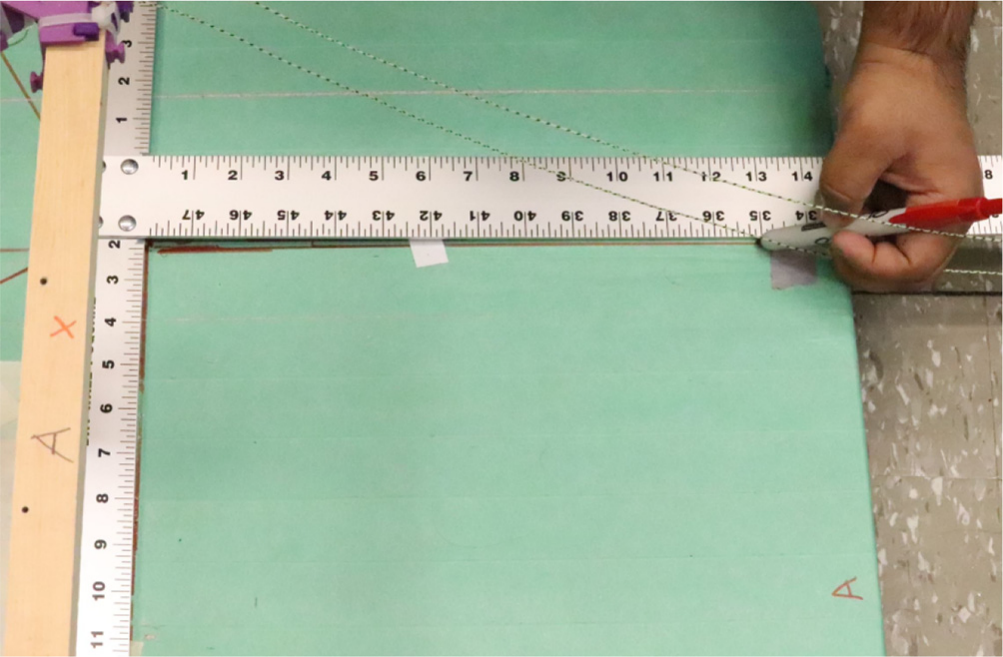
Perpendicular lines on print bed to use as reference for ensuring lines are perpendicular to chassis and anchor.
7. Mark the 3 inner and outer corners as shown in Fig. 174. There corners will be used as reference points against which the X and Y components of the lines will be measured.

**Fig. 174:**
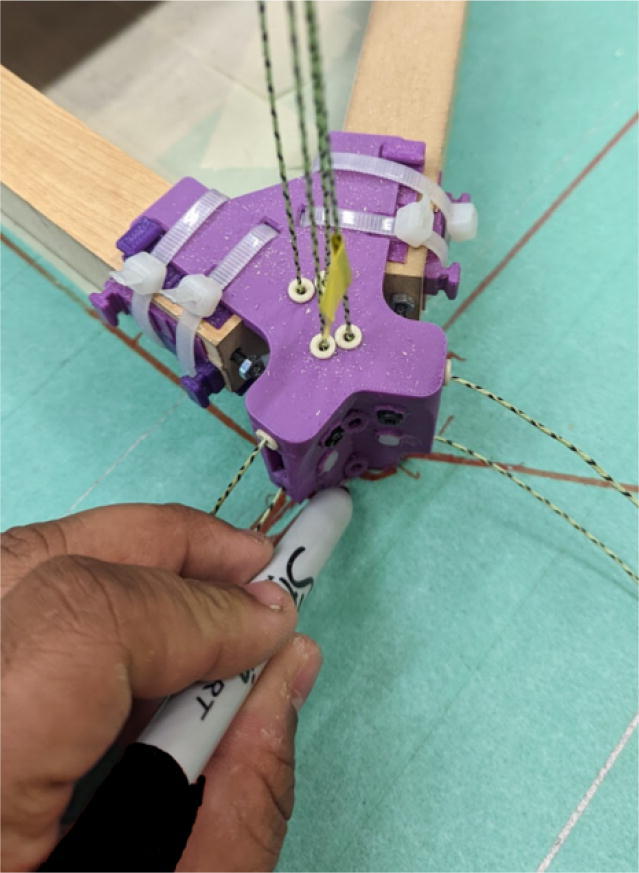
Marking chassis corners on print bed to use as reference points for calibration.
8. Starting with the A-anchor, position the ground anchor into place according to Fig. 175 and Fig. 176. Perform the following steps in order:
  a. Measure the distance from one chassis corner to the corresponding line roller anchor as shown in Fig. 177 using a meter stick or tape measure. Adjust this distance to the desired distance *D* specified in Table 7.

**Fig. 177:**
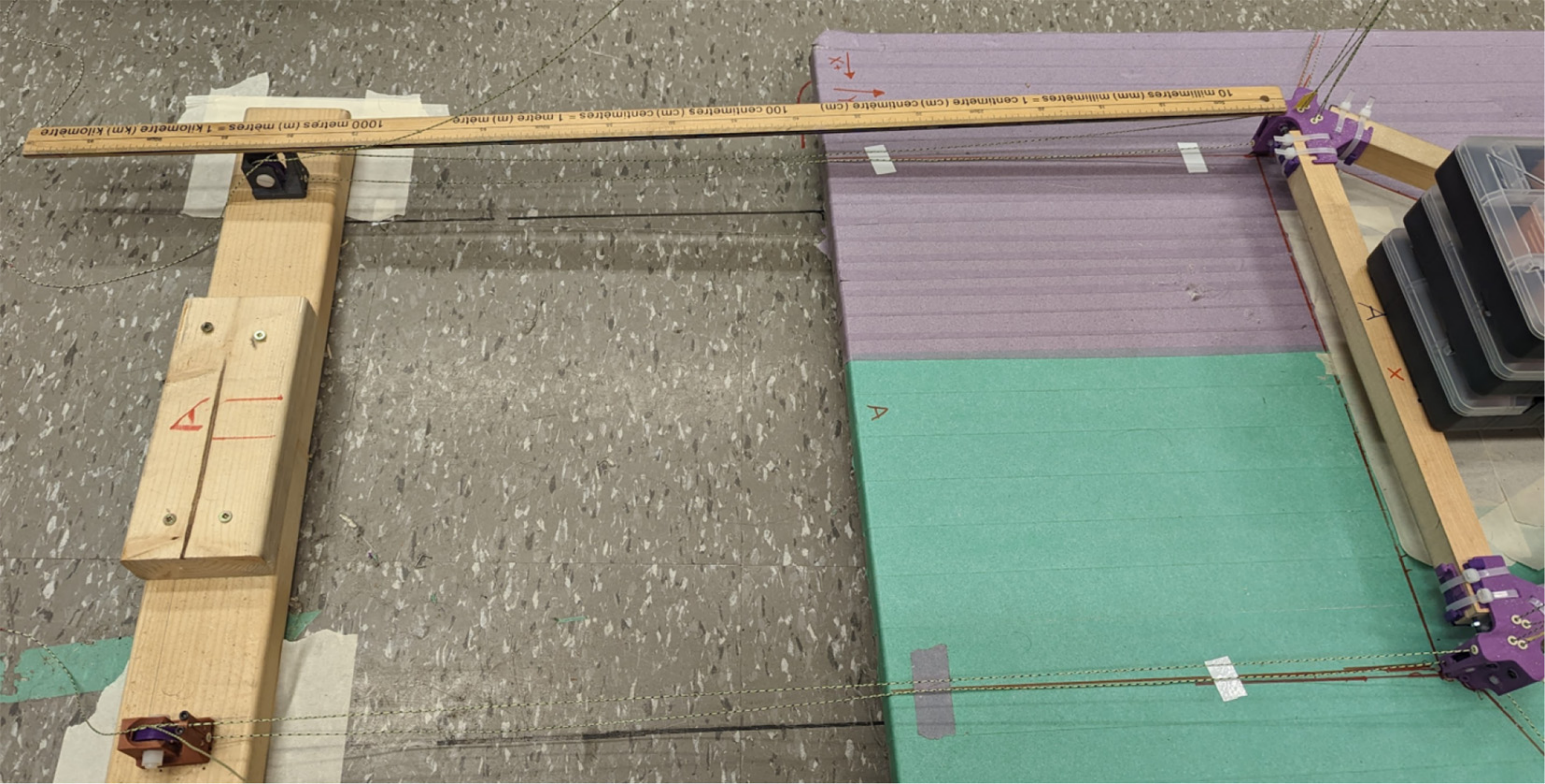
Position A anchor. Same procedure for B and C anchor.
  b. Without moving the position of that anchor, measure and adjust the distance between the other side of the anchor and chassis corner. This can be done by putting your foot or a weight on one end of the anchor and using it as a pivot to adjust the distance of the other side.
  c. Double check both line roller and chassis corner distances and adjust as needed. Ensure both distances are within +/-1mm. This ensures parallelism between the edge of the chassis triangle and anchor point beam on the ground.
  d. Using painters’ tape, lay out strips along the length of the anchor beam near the corners as shown in Fig. 178.

**Fig. 178:**
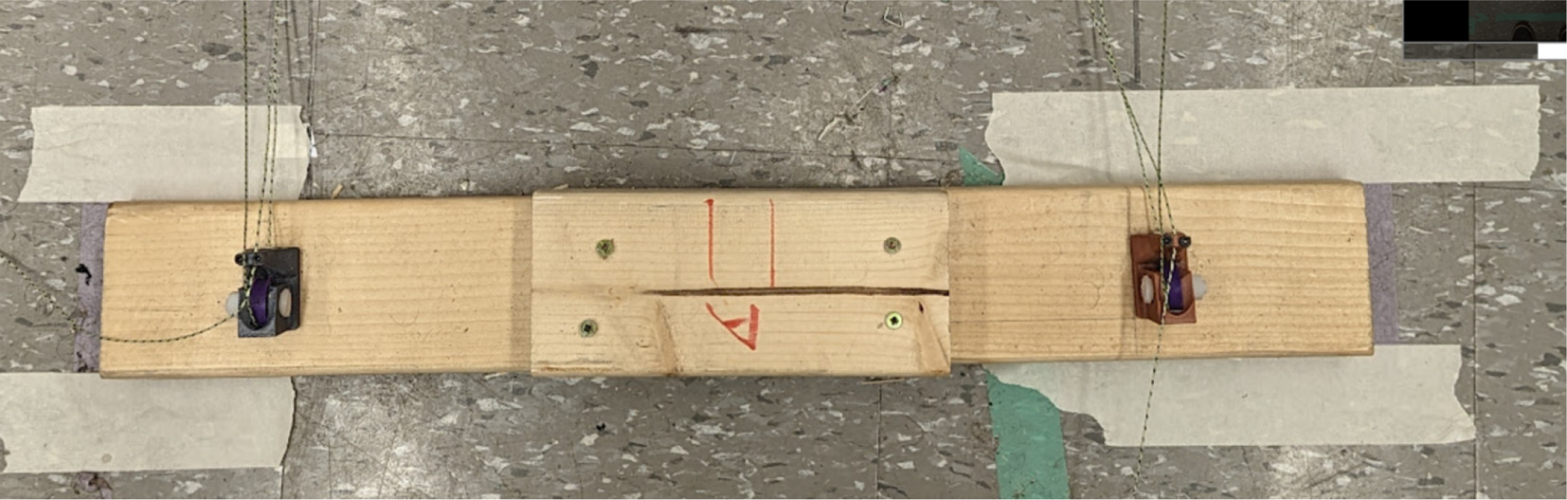
Marking down position of anchor.
  e. Next, align the A lines with the corresponding perpendicular lines drawn from step 6. This ensures perpendicularity of the lines and chassis edge as well as the lines and the anchor beam. Translate the anchor beam along the tape from step 8d and ensure the lines are coincident with the perpendicular lines drawn from step 5 (Fig. 172). Use a builder square as shown in Fig. 179.

**Fig. 179:**
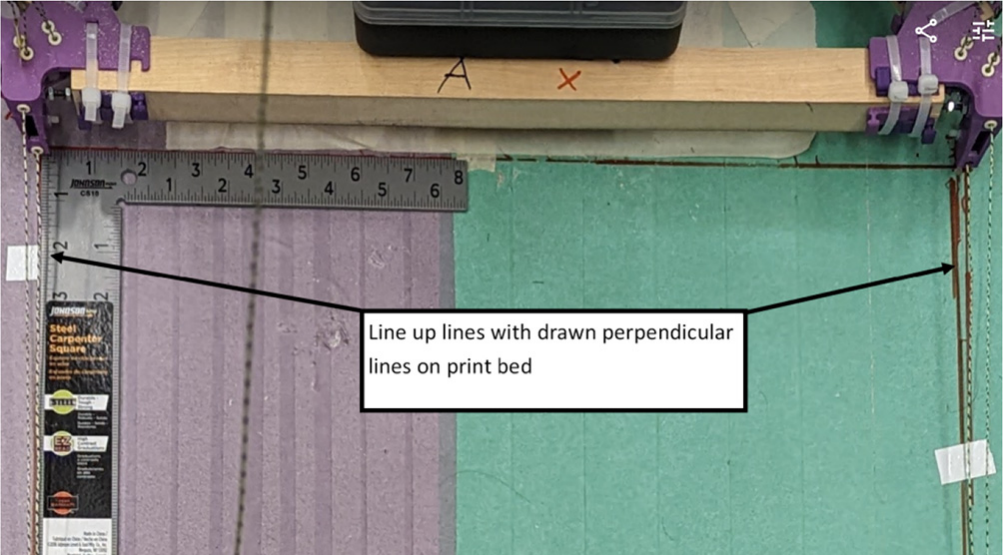
Checking perpendicularity of lines with respect to chassis and anchor.
  f. Once perpendicularity between the lines and anchor beams are achieved, lay out strips of painters tape along the width of the anchor beam to permanently mark the position of the anchor.
  g. Fix the anchor beam with strips of double sided tape spread across the bottom surface of the anchor beam.

**Fig. 175:**
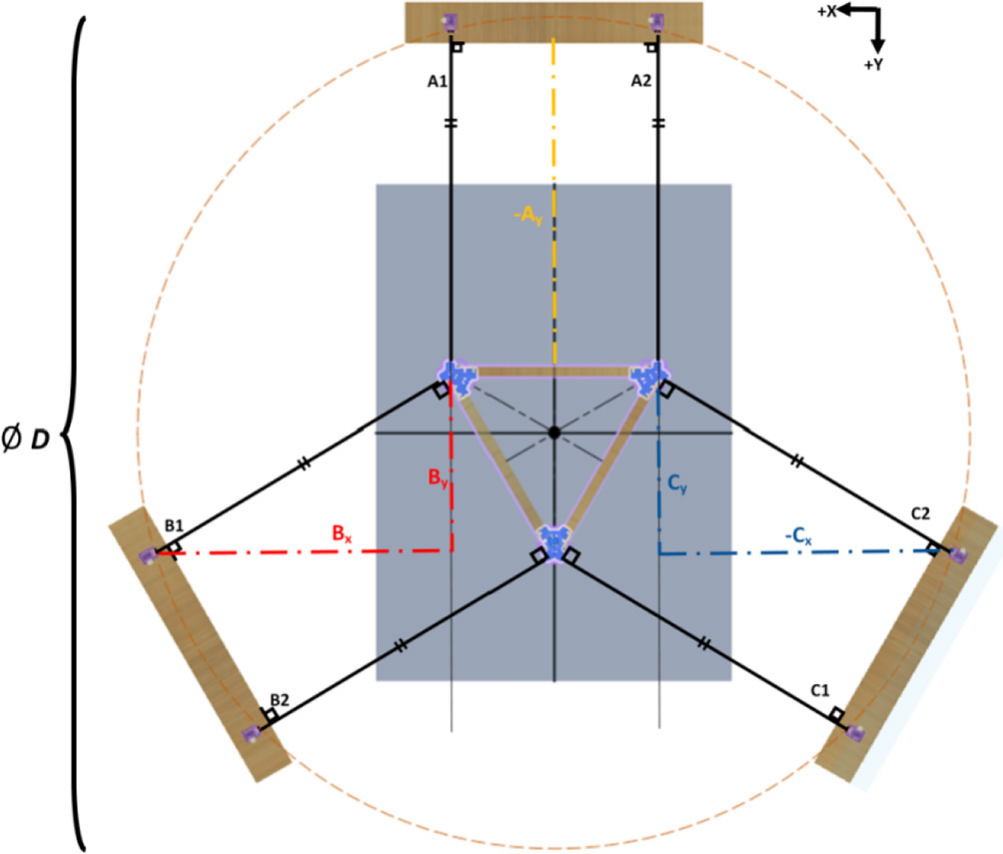
A,B,C lines' X and Y Components.

**Fig. 176:**
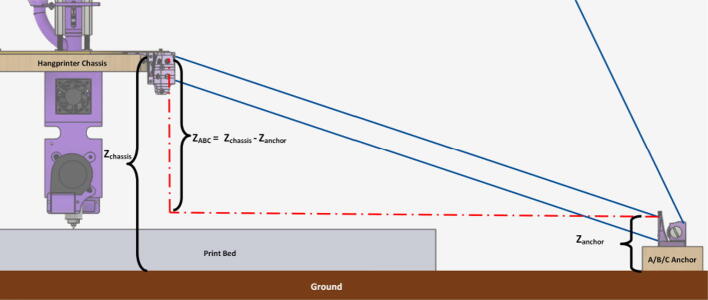
Z component of A,B,C and D anchors.
9. Perform the above steps for the B and C lines.
10. Once ground anchors are fixed, tighten the ABC lines so there is no slack using G1 H2 X-x Y-y Z-z while in relative mode (G91). Lines only need to be taught and take caution to prevent the chassis from moving from its initial position. A,B,C lines may need to be moved on at a time.
11. Verify that the anchor distances, perpendicularity, and parallelism from the previous steps are still maintained using a tape measure or meter stick and builders square (Fig. 180).

**Fig. 180:**
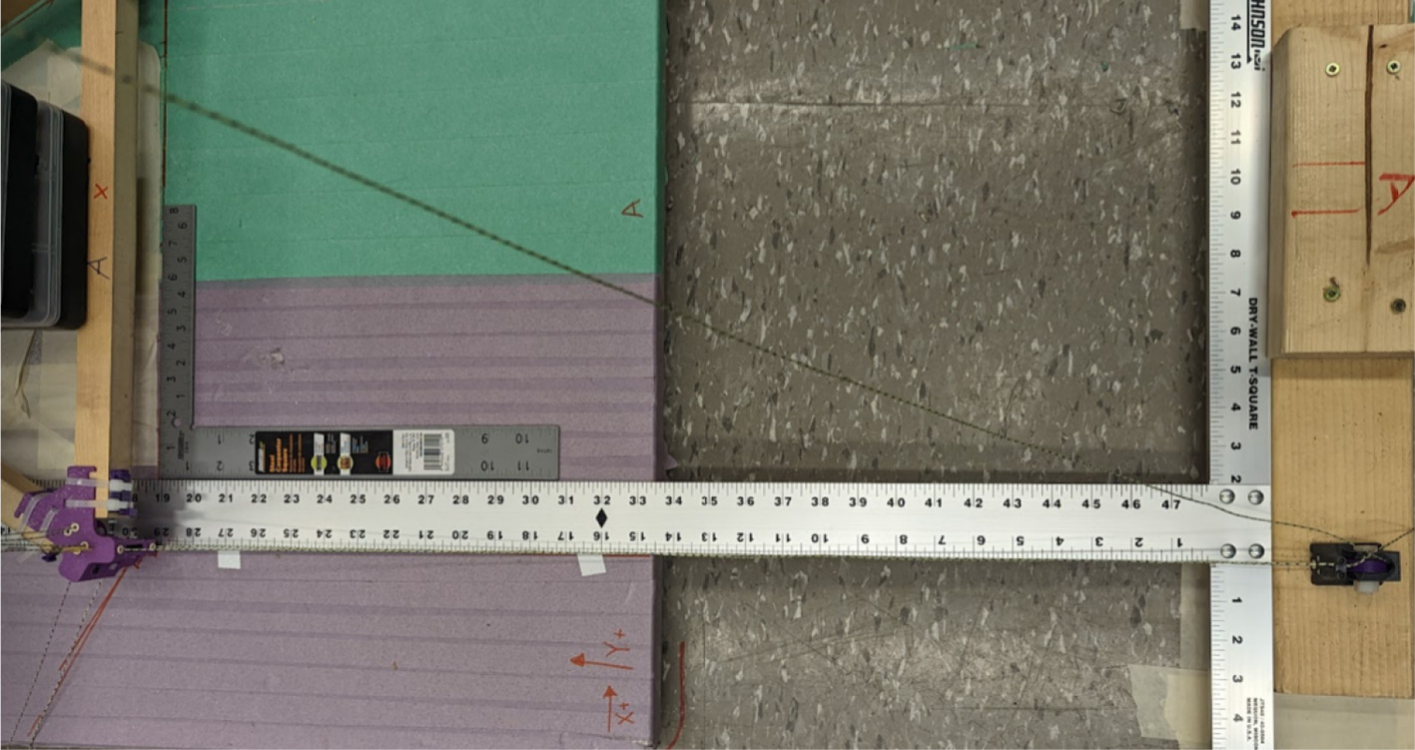
Verifying parallelism and perpendicularity.
12. If the chassis has rotated due to uneven tension between two lines on the same anchor, manually adjust the tension on the line roller anchors with a 3 mm Allen key as shown in Fig. 181.

**Fig. 181:**
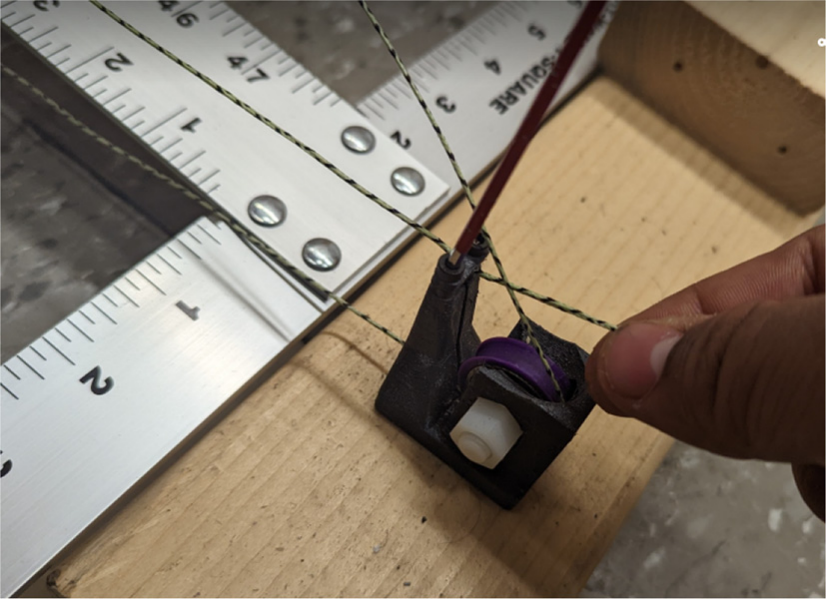
Adjusting tension in line roller anchors to prevent rotation of the chassis.
13. Lift the chassis up approx. 2ft above the print bed and mount the pellet extruder back on.
14. Lower the extruder using G1 H2 Uu in relative mode (G91).

This covers the preliminary setup required to measure line lengths and other parameters to input to the firmware of the DUET3 controller outlined below.

**Fig. 182:**
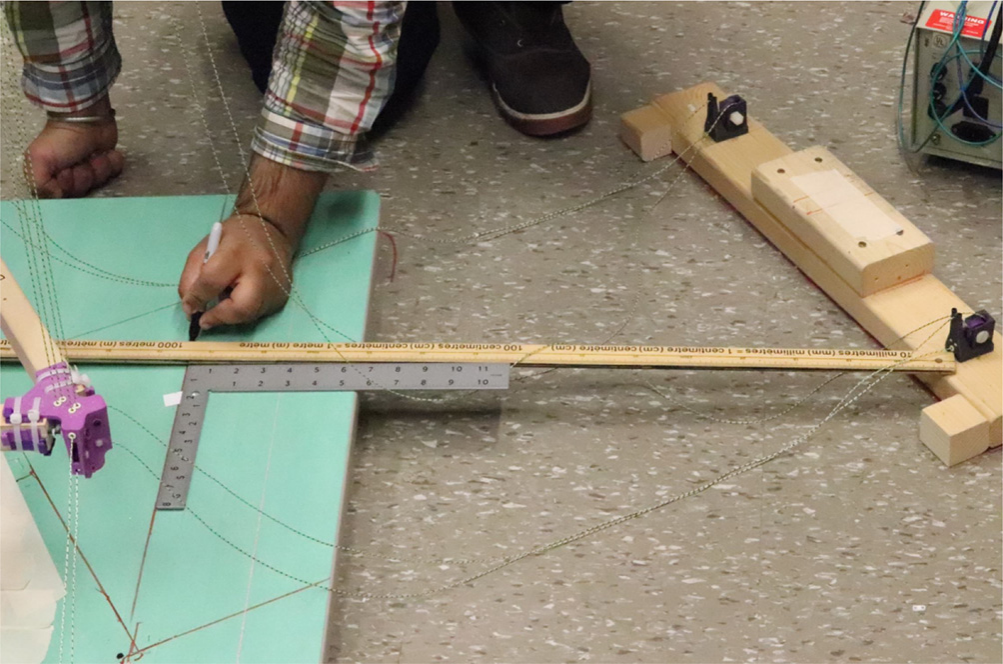
Measuring the X component of the B and C lines.
  b. Mark the point of intersection on the drawn A line with a marker.
  c. Measure the Y component by measuring the distance from this marked point down to the corner of the projected chassis according to Fig. 183.

Line Length Measurements and Other Physical/Firmware Parameters:

Perform the steps below to determine the line lengths between the Hangprinter chassis and each A.B,C,D anchor. To determine the A, B, C, D line lengths, refer to Fig. 175 and Fig. 176 and do the following:

1. With the extruder mounted, use individual motor move commands G1 H2 Xx Yy Zz Uu (while in relative mode G91) to center the chassis within the triangular perimeter drawn on the print bed.
2. Lower the chassis with a G1 H2 U-u in relative mode (G91). Stop until the printer nozzle is approximately 1–1.5 mm above the print bed. Mark a point under the nozzle on the print bed. This point represents the home position of the Hangprinter chassis. *Note: This marked point may not be coincident with the center mark of the print bed. Note the X and Y offset from the center point of the print bed as they will be used later.*
3. Measure the A_X_, AY and A_Z_ components of the A-line.
  a. The X component should be zero and the Y component should be the entire length of line from the chassis to the A anchor.
  b. The Z component is determined according Fig. 176 where it is the difference between the “chassis to groud” distance and “anchor to ground” distance. Measure these distances with a measuring tape or ruler.
4. Measure the B_X_, BY and B_Z_ components of the B-line.
  a. Measure the X component by finding the perpendicular distance from the extended A line drawn on the print bed and the inlet of the line roller anchor that is closer (inward) to the A anchor. Use a meterstick with a builder’s square to ensure that a perpendicular line is found. This is illustrated in Fig. 182.

**Fig. 183:**
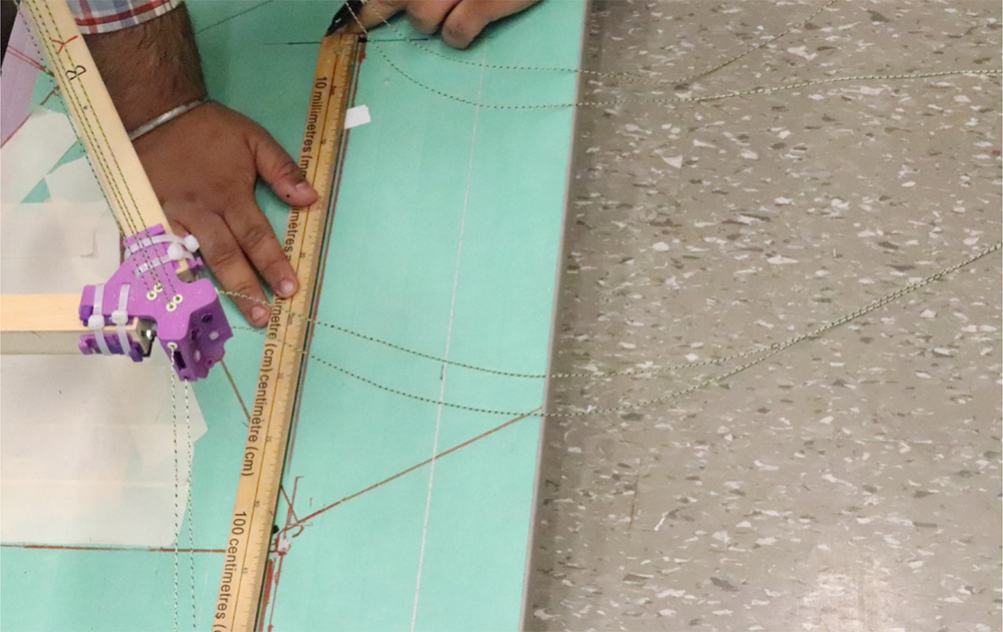
Measuring Y component of B and C line.
  d. The Z component is determined according Fig. 176 where it is the difference between the “chassis to ground” distance and “anchor to ground” distance. Measure these distances with a measuring tape or ruler.
5. Repeat step 4 for the C line.
6. Measure the D anchor line length with a measuring tape. It is the vertical distance from the top of the Hangprinter chassis to the surface of the “line verticalizer” component.
7. Enter these values in the corresponding M669 in the config.g file shown in Fig. 184.

**Fig. 184:**
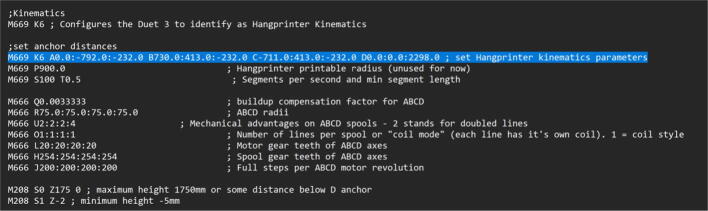
Updating the M669 anchor line lengths parameter in “config.g”.
8. Test movement of the Hangprinter by entering the DWC, tightening the lines and homing the printer using G92 X0 Y0 Z0. Then test movement in the X, Y and Z directions using G90 G1 Xx Yy Zz. If movement is off by a small amount, adjust the M666 and M669 parameters found in the configuration file. If movement is off by a lot or there is significant flex in the lines, re perform the calibration procedure with better measurements.

Troubleshooting steps and helpful resources:

• Hangprinter Discord Group
  o Very active Hangprinter community with many Hangprinter v3 and v4 builders [36].
  o This is the recommended route of action.
• “HangPrinter v3 Part 2 - The Calibration - Chris's Basement” [78].
• “HangPrinter v3 Part 3 - Corrections - Chris's Basement” [79].
• “Links Into Hangprinter V3 Build Video” [80].

### Extruder calibration and feeder system setup

*Notes/tips on extruder and feeder operation*

• *Always preheat extruder to at least 190 °C before running the extruder motor. The firmware will prevent you from extruding at 160 °C.*
• *Feeder motor synchronously release pellets relative to the extruder motor by way of setting E-steps and assigning feeder motor as a mixing extruder.*
• *Instructions below assumes a RepRap firmware (RRF) greater than version 3.2.*
• *Prusa slicer is used for this project.*

It is recommended to first use the parameters that are set in the config.g file provided in [47]. If those do not work or require tuning, some basic steps are provided below.

#### Hot-end PID autotuning

The DUET3 features a FOPDT control algorithm for extruder heaters. Controller parameters can be tuned manually or by using the auto-tuning functionality on the DUET3 board. To perform a PID autotuning routine:

1. Turn on system and enter DWC interface. Ensure that the primary hotend sensor is functional and is indicating room temperature as shown in.
2. In the command line, enter the command “M303 H1 P1 S240. H1 is heater 1, P1 is full pwm and S240 is the setpoint temperature for the tuning in ^o^C. This command will initiate the autotuning process.
3. A message in the console window will indicate that the autotune process has started. The autotune process consists of 4 phases and the completion and commencement of each phase will be indicated in the console window as shown in Fig. 185.

**Fig. 185:**
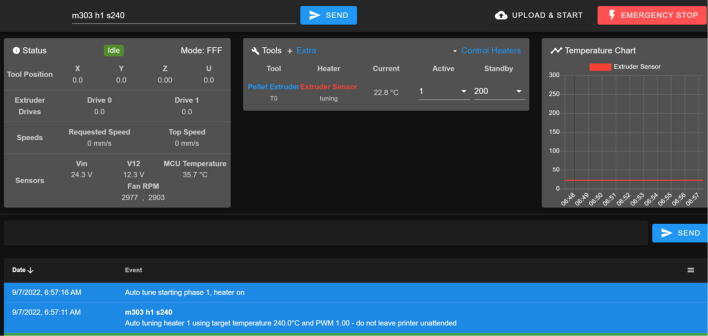
Performing PID autotuning in DWC interface.
4. After successful completion of the autotune process, the following will be displayed in the console window. An M307 command with its corresponding parameters are shown below in Fig. 186. The M307 command is used to set the extruder heater model parameters in the config.g file, thus replace the existing M307 line in config.g with the one shown in the console window and save the config.g file.

**Fig. 186:**
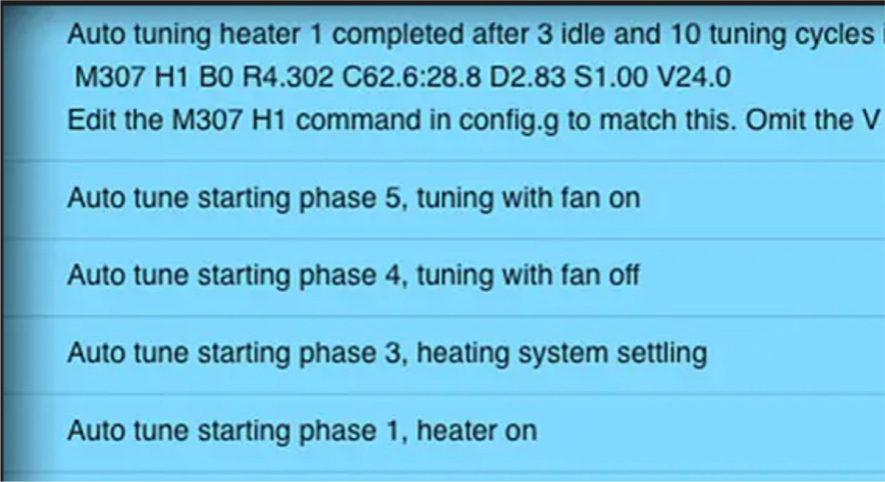
Auto tuning completion results.
5. Verify that the tuning has been successful by turning on the extruder via the DWC interface and changing the active temperature and assess its response on the temperature chart shown beside the temperature control.
6. Change any of the parameters in the M307 line manually if needed (Fig.187).

**Fig. 187:**
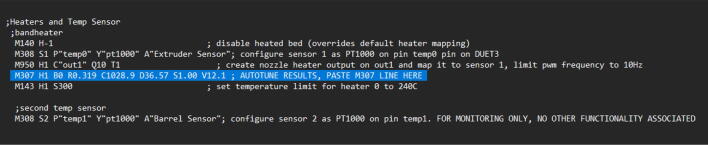
Updating the M307 heater model parameters in “config.g”.

For more information on the various parameters of the tuning process as well as troubleshooting steps in case of a failed autotune routine, visit [81].

Safety warning:

• *During the autotuning process, there is minimal protection against heater faults thus the printer must not be unattended at any time of the autotune process.*

#### Extruder E-step calibration

Since existing slicers do not yet support pellet extruders natively, settings for a pellet extruder does not officially exist in the RepRap community. Furthermore, there are limited methods established in the academic community regarding a calibration process.

A method like calibrating the M92 parameter (E-steps) for filament-based extruders can be adopted while considering the extruder geometry. The steps below assume a “pseudo” filament diameter of 2.85 mm to be entered into the slicer.

**Fig. 188:**
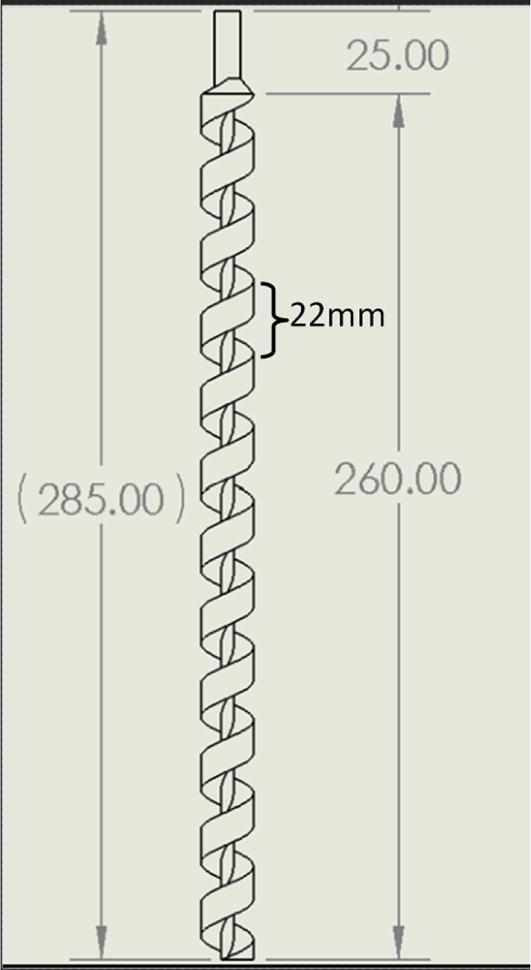
Auger screw parameters.

1. Use equation (10) to determine theoretical E-steps for the extruder motor. For this system, the gear ratio of the stepper motor is 50:1, no micro stepping and the extruder screw has a thread pitch of 22 mm/revolution (Fig. 188). This yields a base E step value of 510steps/mm. If using a different motor, micro stepping and screw adjust E steps appropriately.

(10) $$E\left(\frac{\mathrm{steps}}{\mathrm{mm}}\right)=\frac{S\left(\frac{\mathrm{steps}}{\mathrm{revolution}}\right)*gear\;ratio*driver\_microstep}{thread\_pitch(mm/revolution)}$$

**Fig. 189:**
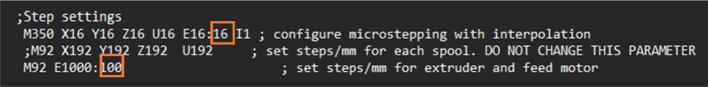
Modifying E steps of extruder.

**Fig. 190:**
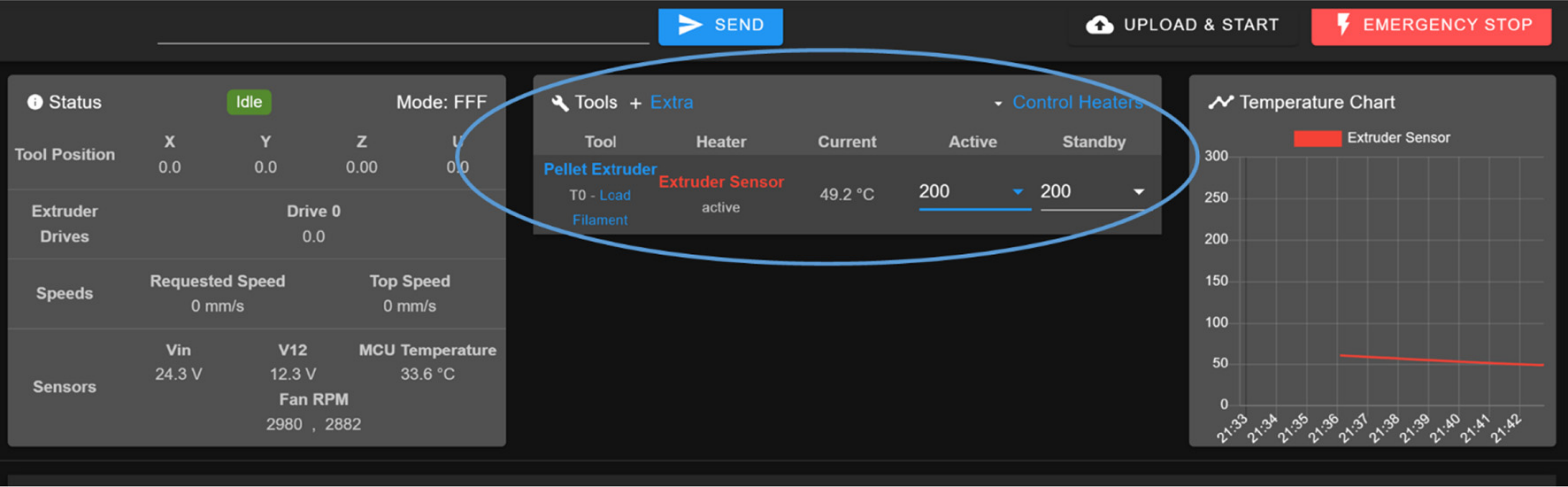
Setting temperature of nozzle via DWC.

**Fig. 191:**
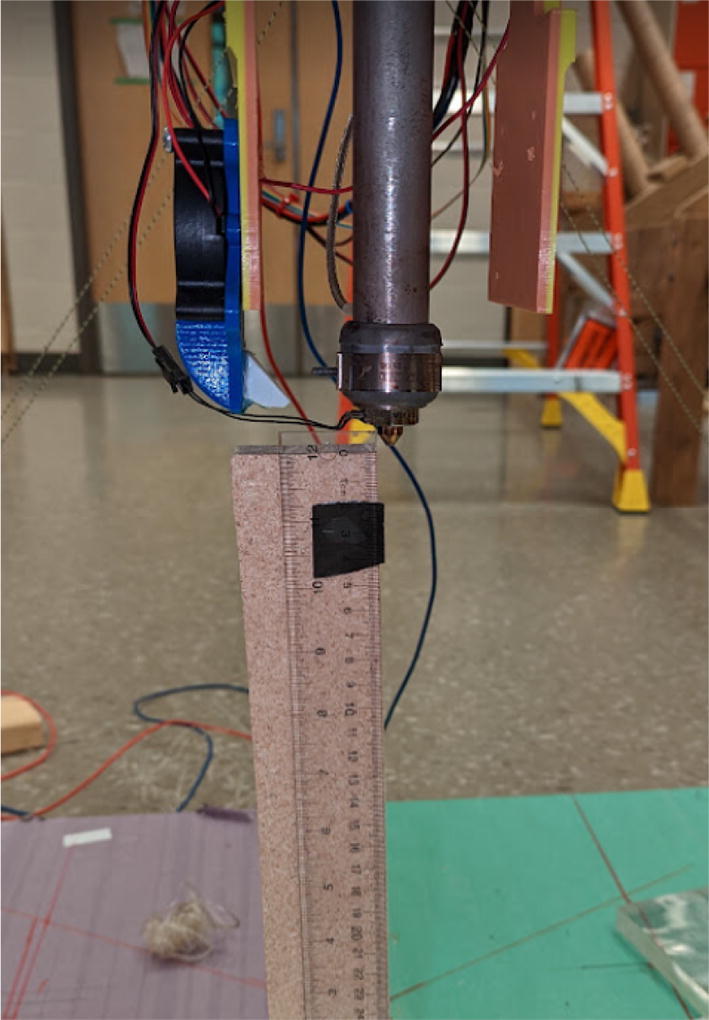
Measuring extrudate from nozzle.

2. Turn on the system and open the DWC. Open the config.g file and edit the M92 Ee parameter where “e” is the E-steps of the extruder in Fig. 189.

3. Set active temperature of the extruder to 200 °C (for PLA) on the dashboard as shown in Fig. 190.

4. Send a G1 E50 command to tell the firmware to extrude 50 mm of extrudate. Measure the length of extrudate coming from the nozzle with a ruler or caliper (Fig. 191) and note it down.

Use equation (11) to determine the new steps/mm setting

(11) NewEstep=(originalesteps∗50)/actuallengthextruded

6. Repeat step 4 and 5 until consistent extrusion at the right length is achieved. An extruded length with a variability of +/-2mm is acceptable.

7. In the config.g file, change the M92 Ee where “e” is steps/mm to the final calculated value.

*Note: The pellet feeding system is assigned as a secondary mixing extruder in the config.g file. During testing or manual extrusion, ensure that there is consistent feed to the extruder. Any signs of inconsistent extrusion may be a result of insufficient feed to the extruder from the feeding system. Based on M92 and mixing ratio settings, the feeder system will release pellets at a rate proportional to the extrusion rate of the pellet extruder via different E-steps set for the extruder.*

For more information on the calibration of various extruder parameters, see the “Characterization and Validation” section of this paper.

#### Feed system setup

**Fig. 192:**
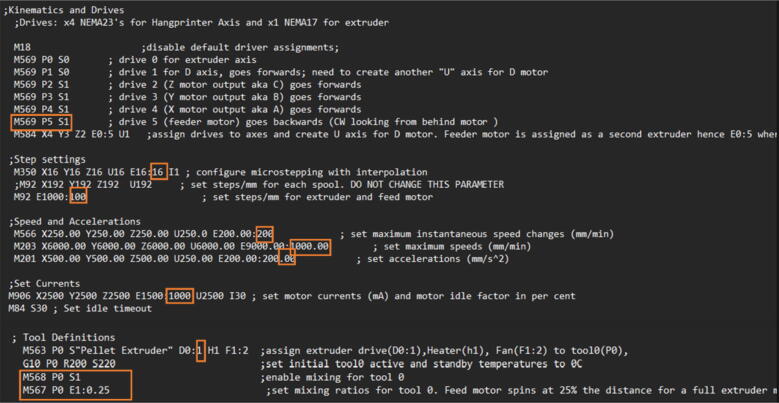
Feeding system parameters in Config.g file.

The feeder system for this project is set up as a second extruder in firmware. The feed motor is mapped to a second extruder drive. By setting up the feeder system as a simultaneous second extruder in firmware, E steps and mixing ratios can be manipulated to adjust the proportional of pellets fed to the pellet extruder relative to the extruder motor rotation. Shown in Fig. 192 are the relevant feed motor settings. All of these settings can also be found in the “config.g” file in [47]. For most of these settings the syntax is M/GXXX EX: Y where X is extruder motor and Y is the feeder motor.

The pellet extruder flow rate’s consistency depends on the feed rate of pellets incoming from the pellet feeding system. The E-steps and mixing ratio were experimentally determined for this project’s specific setup. Unless there are issues with extrusion, these values in the config.g file do not need to be changed. If inconsistent extrusion or under extrusion at a given RPM occurs, the e-steps (M92) or federate (F) of the feeding system motor may need to be changed by considering equation (12). Additionally, adjusting the mixing ratio specified by M568 and M567 shown the bottom of Fig. 192 is another way of controlling the number of pellets released. The syntax is as follows: M567 P0 EX:Y where X:Y is the ratio of the pellet extruder to the feeding system motor rotation.

(12) $$\mathrm{RPM}=\frac{F\left(\frac{\mathrm{mm}}{\min}\right)*E\left(\frac{\mathrm{steps}}{\mathrm{mm}}\right)}{S(\frac{\mathrm{steps}}{\mathrm{revolution}})}$$

• F is the feed rate and specified by the user in a G1 extruder command.
• S is the steps per revolution specification of the stepper motor. For this project, S = 200steps/revolution*5:1 gear ratio thus S = 1000 steps/revolution.
• E is the number of steps that produces 1 mm of linear movement from the stepper motor.

By changing the M92 E-step and M567 mixing ratio parameters, the number of pellets per rotation and the feed rate of pellets released from the hopper can be controlled. Experimental testing by trial and error will be required to properly determine parameters that result in consistent extrusion and no jamming in the extruder.

1. Feeder motor Esteps can be changed via the config.g file in the M92 command line. Change the Y(steps/mm) in M92 EX:Y.
2. Adjust mixing ratio in the M567 line of config.g file.
3. the extruder calibration steps outlined above and observe for any under extrusion or pellet jams.

### Slicing and using the machine for printing

The basic workflow for 3D printing with the Hangprinter is like that of a normal 3D printer. Some differences are:

• No end-stop based homing.
• No retraction.
• Volumetric based extrusion.
• Larger nozzle diameter and line widths.

Before starting a print, it is recommended to familiarize yourself with some basic Gcode commands that pertain to this system. These were outlined above in Table 24 and Table 25.

Tips:

• When homing Hangprinter on an initial power up, it is recommended to use individual motor moves (G1 H2 Xx Yy Zz Uu) and relatively slack lines when positioning nozzle to origin.

To start a print:

1. Load the feeding system hopper with pellets.
2. Using G1 H2 motor commands, position the print head approximately 2 mm above the center mark of the print bed. Tighten/loosen lines as needed.
3. Enter G92 X0 Y0 Z0 when nozzle is positioned directly over the center point marked on the print bed.
4. With the system plugged into an outlet, turn the system on and access the DWC web control UI by entering the IP address determined from section 6.1 above.
5. Upload the sliced.gcode file to the DUET3 by accessing the jobs menu and clicking upload.
6. Right click on the file and select Start.
7. The DWC will change to display print status and bring up print tuning controls.
8. Adjust and monitor printing as needed.

A base profile for slicer settings using the Prusa Slicer has been provided in [47]. It is recommended for the user to experiment with the slicer settings for themselves to get good quality prints. Furthermore, the slicer configuration includes custom startup G-code unique to the Hangprinter. If using another slicer, the user must ensure that they copy over this startup code to that slicer according to its G-code flavor.

### Maintenance and cleaning

• To clean the nozzle, use a bristle brush such as the one shown in Fig. 67 while the system is heated to a sufficient temperature.
• To clean the inside of the barrel assembly, heat the extruder to approximately 220 °C and carefully unscrew the nozzle adapter with a wrench shown in Fig. 67*.*
  o Use the bristle brush to clean the inside of the barrel.
• Use compressed air to clean the hopper and internal components of the extruder such as the intake tube and auger channels.

### Safety

• Ensure that the appropriate ladder is present for the height at which the ceiling mount will be suspended.
• The entire construction of the Hangprinter system can be a trip hazard if one is not cautious in minding the wires and lines.
• Do not stand within the Hangprinters print area while in operation.
• Do not touch any live AC wires, terminals, or the heater while it is on as it can cause electrocution and burns.
• It is recommended to plug the Hangprinter power supply into a power bar with a breaker and manual on/off switch.

## Validation and characterization

### Extruder

#### Methodology

**Fig. 193:**
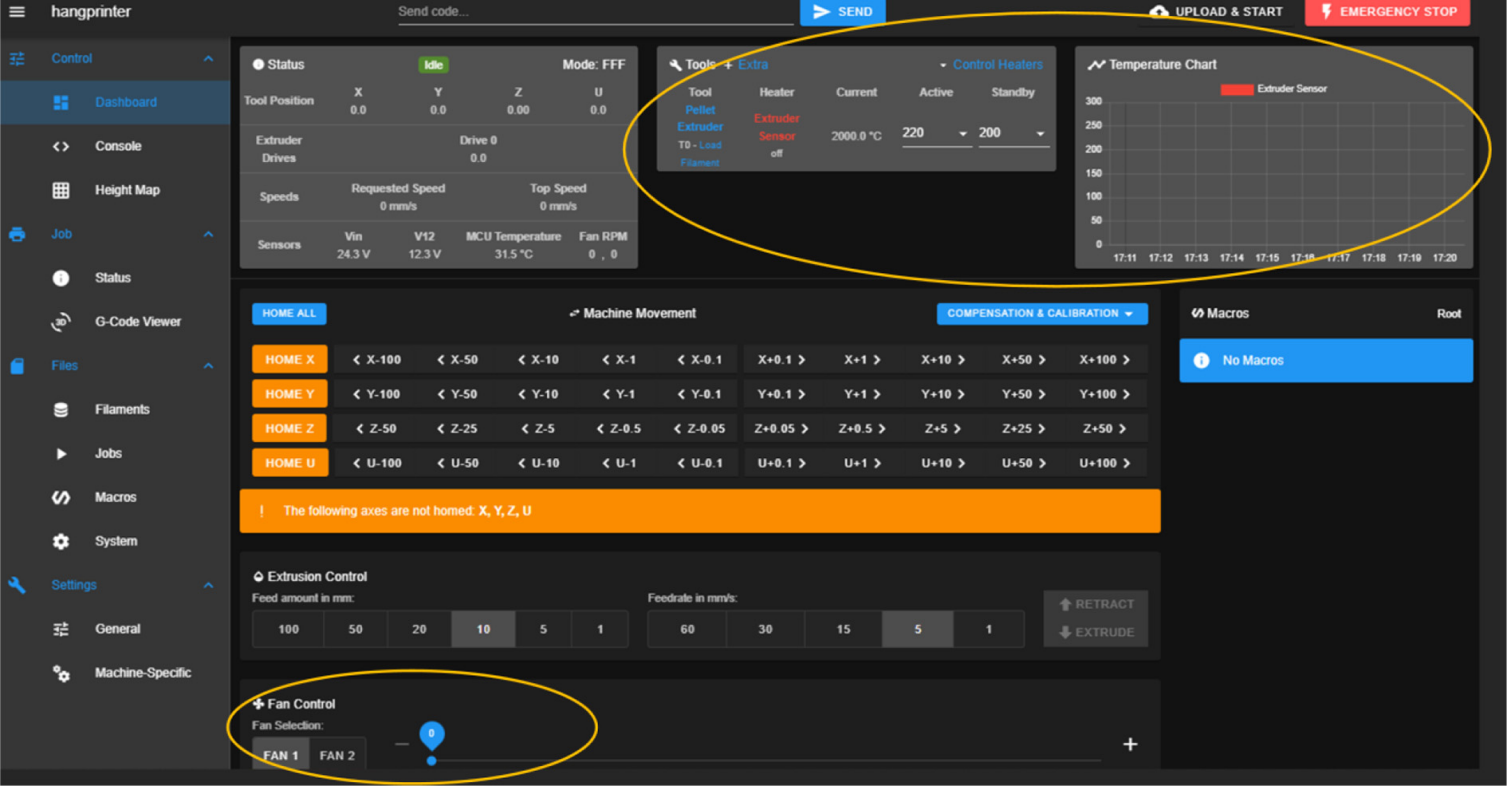
DWC Interface for controller fan speed and nozzle temperature.

Basic experimental testing was done to determine the important operating limitations of the extruder, perform E-step calibration, and assess the overall performance of the extruder at various temperatures and speeds. The various operating limits were determined according to methods outlined in Table 26. To further characterize the heating and cooling of the extruder, thermal images were captured to evaluate the heating and cooling of the nozzle and heatsink block respectively. Temperatures and fan speeds were controlled via the DWC GUI interface as shown in Fig. 193. Fan speeds of 0 % and 100 % were tested against nozzle temperatures of 180 °C-240 °C in 20 °C increments. Each temperature interval was held for 1 min before the measurement was taken to allow for the system to heat up evenly with the PLA pellets inside the extruder. Thermal images were taken with a FLIR c5 1.1 model camera [82], [83]. Next, the E-step calibration procedure outlined above in the operating instructions section was used to ensure that the correct amount of extrudate is extruded from the nozzle with respect to the commanded extrusion value. The above-mentioned tests were performed with the settings as found in the config.g file in [47].

**Table 26:** Operating limits of pellet extruder.

**Parameter**	**Test Criterion**	**Method**
Upper Temperature (°C)	Until system cannot raise temperature anymore or until it reaches firmware limit	Control temperature via DWC interface and monitor highest reachable temperature. Attempt to extrude 100 mm of extrudate using command G1 E100:0 F2000
Lower Temperature (°C)	Temperature at which extruder motor and auger stall consistently	Lower temperature in 20 deg intervals via DWC interface and monitor lowest reachable temperature. Attempt to extrude 100 mm of extrudate using command G1 E100:0 F2000 for each temperature.
Max Overshoot (°C)	Highest temperature reached minus stable temperature	Determine overshoot from temperature chart in DWC interface
Error (°C)	|Theoretical temperature - actual temperature|	Heat nozzle up to target temperature of 200 °C
Max Auger RPM	Speed at which extruder motor and auger consistently stall	In DWC, using command G1 Ee:0 Ff where *e* is some large extrusion value to provide at least 2 rotations and *f* is the max possible linear feed rate of extrudate before auger stalls Mark a point on shaft coupler of extruder and m ensure time for one rotation with stopwatch
Max Feedrate (mm/s)	Speed at which extruder motor and auger consistently stall	Extruder run at max auger RPM using the same G1 command above. Time of extrusion recorded on stop watch and extrudate measured

The extruder accuracy was tested to define the optimal extrusion process parameters. To this end, different cylindrical samples were extruded at 4, 8, and 16 mm/s by changing the extruder temperatures (165, 170, 175, 180, 185, 190, 195, and 200 °C), resulting in 24 3-D printed samples. Ingeo Biopolymer 4043D PLA (NatureWorks LCC, Plymouth, MN, US) in the form of pellets was used as testing material. The cylindrical shape has a nominal diameter of 70 mm, and a height of 15 mm. The sample was sliced with a layer height of 1 mm, one perimeter, no bottom/top layers, and 0 % infill. The samples were then visually inspected and measured to compare the actual weight, time, average line width, and overall dimensions with the nominal ones [84], [85], [28]. The expected weight was calculated by inserting the density value from the NatureWorks technical datasheet in PrusaSlicer (Prusa Research, Prague, Czech Republic).

#### Results

The primary operating limits and capabilities of the pellet extruder are outlined below. From a qualitative observation, the ideal extrusion temperature range for virgin PLA pellets is between 180 °C and 190 °C. Extrusion above this temperature resulted in significant oozing and degradation in quality of extrudate. The lowest temperature before extrusion does not occur due to excessive polymer viscosity and extruder clogging is 160 °C at a normal commanded extrusion speed of 22 mm/s (approximately 8RPM). One important observation from these experimental tests was that the Nema17 extruder stepper motor stalls very easily at higher RPM (>10RPM). This is due to the high gear ratio (51:1) and low rotor inertia. This in turn is the limiting factor in the print speed of the overall system. The limitation can be overcome in future work by using a stronger motor with a smaller gear ratio however, extra weight to the Hangprinter chassis only further limits acceleration and print speed. Furthermore, from the thermal image testing (Table 27, Table 28), it can be seen that the passive cooling system of two axial blower fans cooling the aluminum heatsink block does a satisfactory job of ensuring upper barrel temperatures do not exceed 60 °C, which is the glass transition temperature of PLA [86]. During extrusion tests with PLA pellet feedstock, no extruder clogs due to heat creep or cold starts up the barrel occurred even at 0 %, 50 % fan pwm. Furthermore, with fan pwm at 100 % and nozzle temperature of 220 °C-240 °C, ABS granules with glass transition of 97 °C [86] can also theoretically extruded without risk of heat creep up the barrel.

**Table 27:** Thermal captures of various nozzle temperatures.

**Nozzle Temperature (^o^C)**			
**180**	**200**	**220**	**240**

**Table 28:** Upper barrel temperatures and various fan speeds and nozzle temperatures.

**Fan PWM(%)**	**Nozzle Temperature (^o^C)**			
	**180**	**200**	**220**	**240**
**0**				
**100 %**				

Outlined below is a summary of the capabilities and specifications of the pellet extruder:

● Capable of printing PLA with a standard M6 1–3 mm orifice nozzle and off-the-shelf auger screw.
● Extrudes at a temperature from 165 °C to 210 °C for virgin PLA granules.
  ○ ±0.8 °C error @200 °C
  ○ <1°C overshoot on initial heat up
● Extrudes at feedrates up to 22 mm/s with auger RPM of approximately 8 RPM.
  ○ M92 (E-step setting) @1000 steps/mm

**Fig. 194:**
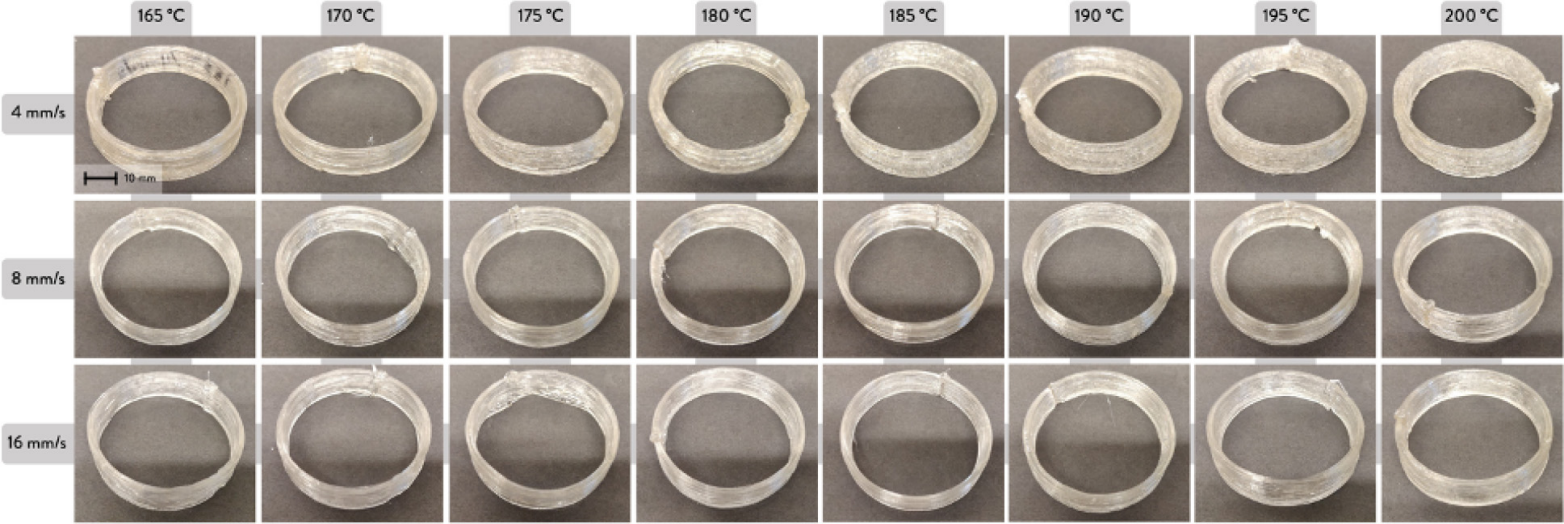
Cylindrical samples printed at different temperatures (165–200 °C) and speeds (4–16 mm/s).

Twenty-four cylindrical samples were 3-d-printed to assess extruder accuracy and select the optimal parameters using commercial PLA pellets. Fig. 194 shows the 24 samples obtained from the test. From a qualitative point of view, two main differences may be observed by comparing them. First, the samples show a change in their appearance. The more the temperature increased, the more the samples assumed a yellowish aspect, and the consistency of the extruded material decreased. Similarly, the same issue occurred by reducing the printing speed, resulting in a more visible effect in the sample printed at 4 mm/s, 200 °C. This issue is linked to the degradation of the PLA in the extruder chamber, which increases by extending the time the material remains in the extruder [87]. Despite the less color change, higher speeds increased the possible deformations of the samples, especially in the layer height consistency. The effect is noticeable by increasing the printing temperature and may affect the overall sample, i.e., slightly conical shape. This effect is common for large-scale 3-D printers and is usually linked to the longer cooling time influenced by the nozzle dimension, hence the thicker line width and layer height [88]. A difference in the wall thickness was also detected by changing the speed and temperature. In short, low printing speeds and higher temperatures resulted in thicker lines (over-extrusion), whereas higher printing speeds and lower temperatures led to thin walls (under-extrusion). In this case, the pseudoplastic behavior of PLA may affect this aspect by changing its viscosity during the extrusion according to the printing speed and temperature [89].

**Fig. 195:**
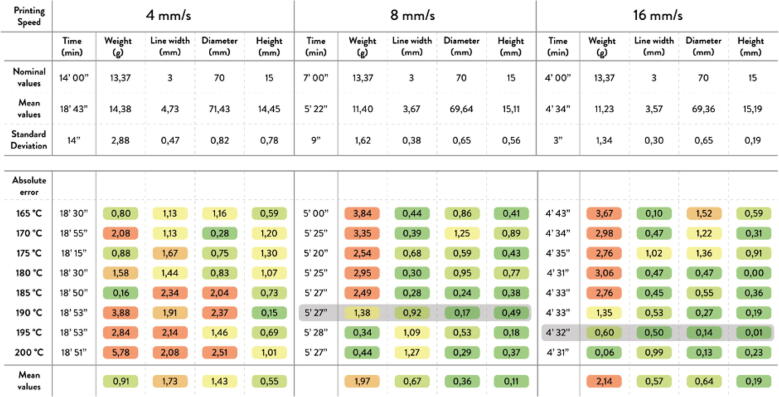
Time, weight, and dimensional accuracy of the 3-D printed circular samples at different temperatures (165–200 °C) and speeds (4–16 mm/s).

The weight and dimensions of the samples were then measured and compared to the nominal ones to assess the extrusion accuracy from a quantitative point of view. Fig. 195 summarizes the measurement results, together with the actual printing times. Less accurate values are visible for the samples printed at 4 mm/s, especially considering the weight. In particular, higher absolute errors are visible by increasing the printing temperature, causing over-extrusion, especially at 190–200 °C. On the contrary, under-extrusion occurred at 8 and 16 mm/s when using lower temperatures, i.e., 165–185 °C, with ∼2–4 g of absolute error. The average line width is higher for all speeds, although lower differences occur at 8 and 16 mm/s. Comparing the printing temperature, the line width is usually more accurate between 165 and 185 °C with a variation of ∼0.2–0.6 mm. According to the previous values, the samples printed at 4 mm/s have bigger diameters than the nominal values, whereas smaller diameters were measured from the samples printed at 8 and 16 mm/s. In this case, lower absolute errors occurred at 165–180 °C and 185–200 °C printing at 4 mm/s and 8–16 mm/s, respectively. Finally, the cylinders show the opposite variation of the mean height with smaller samples at 4 mm/s. Similarly, smaller cylinders were obtained at high temperatures for higher speeds, i.e., 185–200 °C, even if their absolute error is lower, ranging from ∼0.1 to ∼0.3 mm.

In general, the values are in line with the previous qualitative assessment, giving a preliminary confirmation of the optimal printing settings for the Hangprinter. Furthermore, these values empirically demonstrate the relationship between the printing temperature and speed settings on the weight and dimensional accuracy [87], [88], [89]. More accurate values can be reached at 8 and 16 mm/s, especially extruding at 190–200 °C. Additional tests are reserved for future work on different materials, i.e., recycled feedstock, and geometries, to enlarge the possible applications of the Hangprinter.

Recommended slicer settings pertinent to the extruder can be found in [47].

### Entire hangprinter system

#### Methodology

**Fig. 196:**
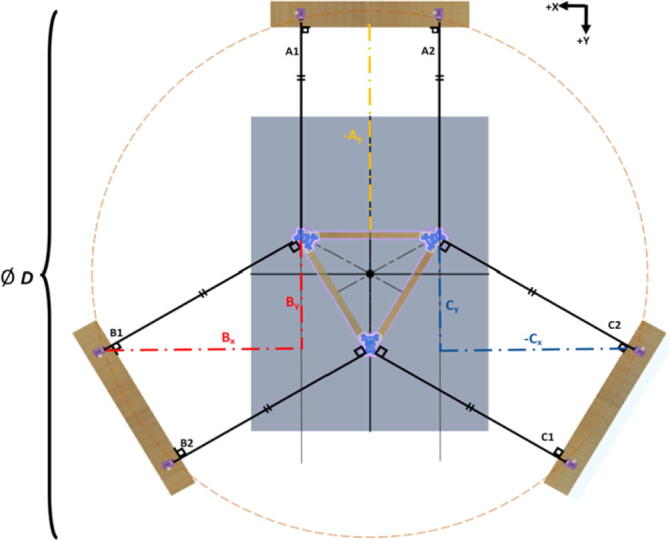
A,B,C anchors and their XYZ components.

**Fig. 197:**
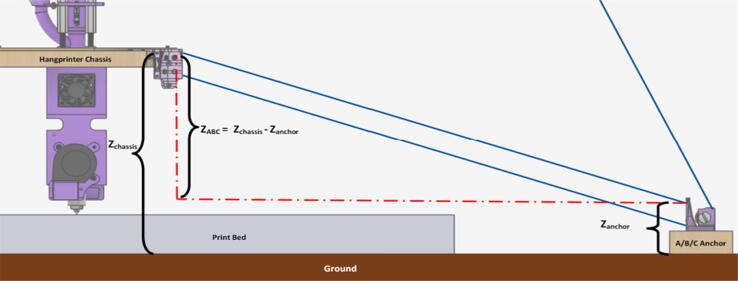
Z component of A,B,C anchors.

To assess the capabilities and performance of the Hangprinter, calibration of its anchor line distances is needed. This is important so that the firmware can correctly use equations (1), (2), (3) outlined in the beginning of this paper properly. Calibration of the Hangprinter system was carried out according to the procedure outlined in the “Hangprinter Parameters and Calibration” portion of the operating instrctions section. A manual calibration procedure similar to [78], [80] was adopted and a tape measure and builders square were used for measurements and ensuring parallelism and perpendicularity between the lines and anchors. Measurements for -Ay, Bx, By, -Cx,Cy, -Zabc in Fig. 196 and Fig. 197 were made. These measurements were then entered into the following line in the config.g file on the DWC interface:

M669 K6 Aax:-ay:-az Bbx:by:-bz C-cx:cy:-cz D0:0:dz

where ax,ay,az,bx,by,bz,cx,cy,cz, and dz are the x,y,z components of the A,B,C, and D anchors of the Hangprinter.

**Fig. 198:**
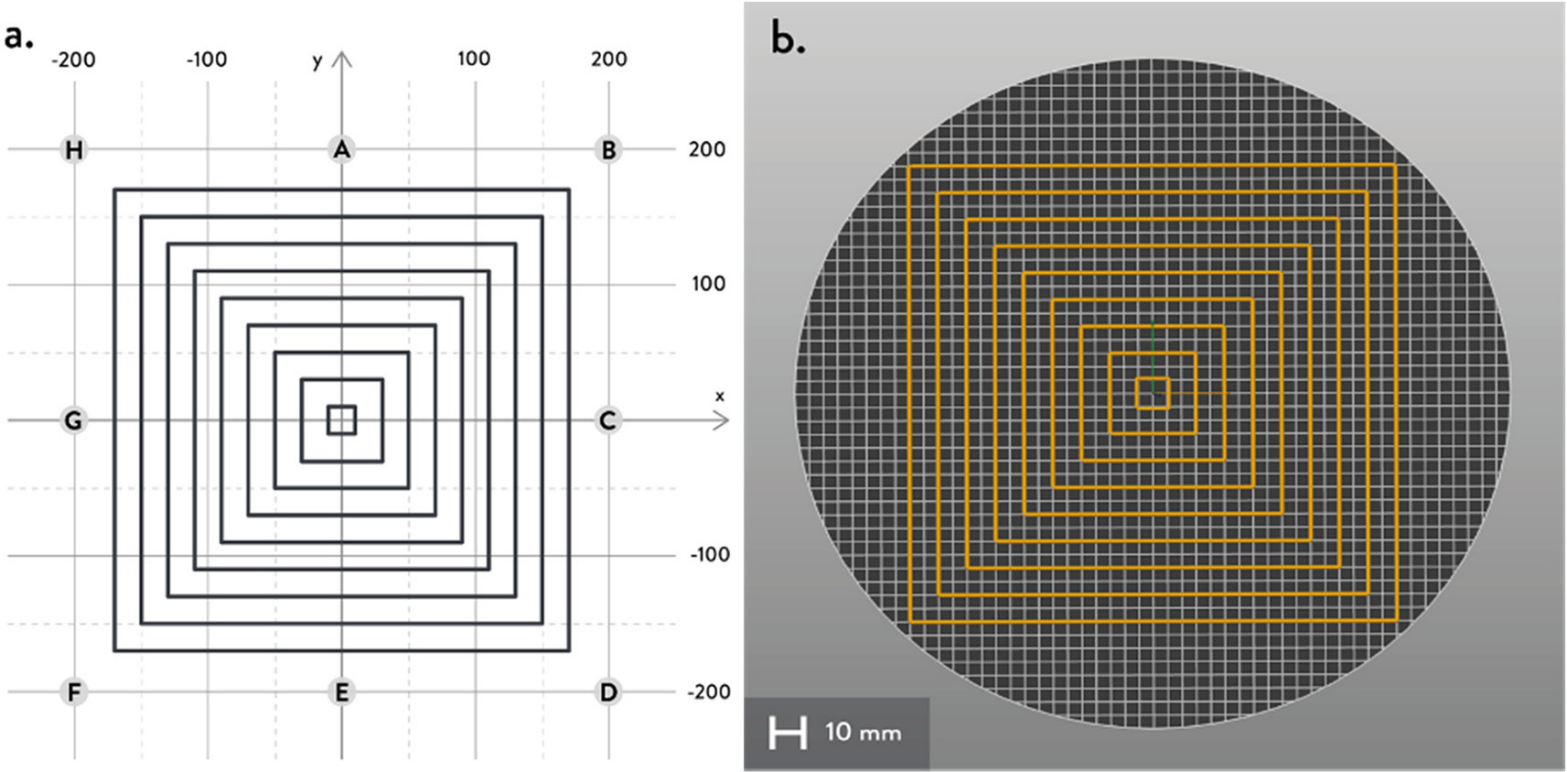
Positioning accuracy test. gcode files. (a) path of the test with its overall dimensions; (b) gcode preview of the square pattern.

The positioning and dimensional accuracy were tested after performing the Hangprinter system's manual calibration procedure as discussed in the Hangprinter operating instructions section and a specific test has been designed starting from the work of Petsiuk et al. [35]. This test consisted of a path with single-layer nine sharp concentric geometries with dimensions from 20 to 340 mm (Fig. 198a). A gcode file was then generated by using PrusaSlicer, disabling the extrusion (Fig. 198b). Some grid paper was then fixed to the printing bed, as well as a marker on the extruder head. The actual coordinates were tracked by starting the gcode and drawing the movements with the marker at a speed of 8 mm/s. The coordinates of the points intersecting with eight different lines were then measured for each square, resulting in eight sets of x-y coordinates (A-H). These values were compared with the nominal values to plot the absolute error in mm. The test was repeated at least three times to obtain the average absolute error values, and the ropes were tightened at the end of each test to reset the previous positioning errors.

#### Results

**Fig. 199:**
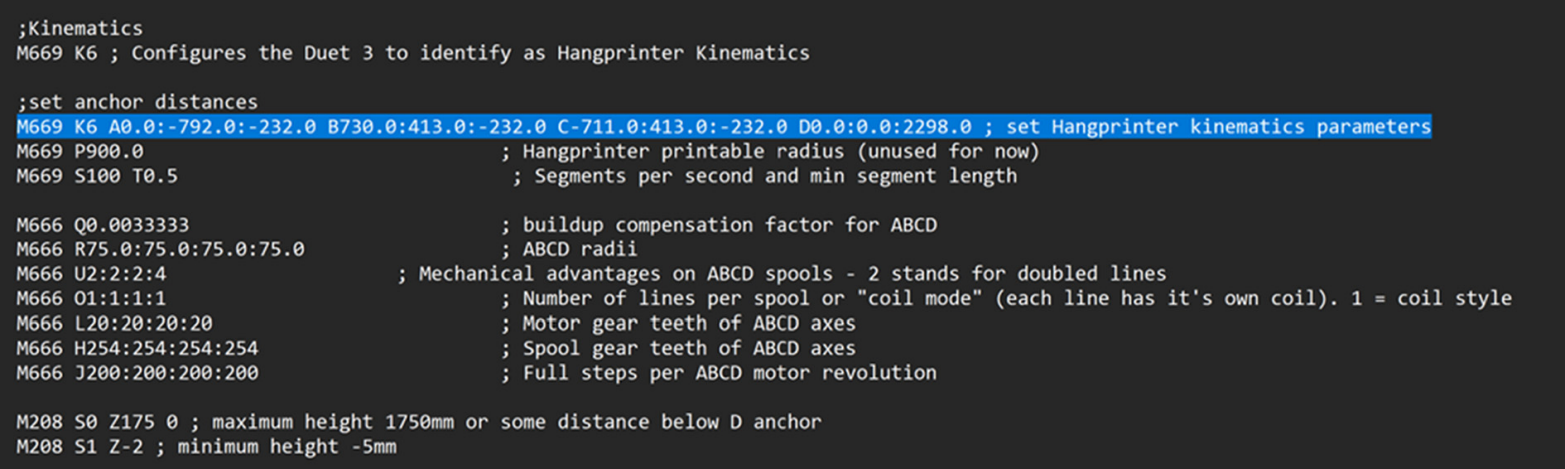
Line anchor distances in Config.g file.

The results of the Hangprinter calibration are listed below. The same values can also be found in the config.g file in [47]. Other “M669” and “M666” parameters pertaining to gear ratios, line build up compensation were also fine tuned by trial and error to ensure correct movement on all axes. The results are shown below in Fig. 199.

Anchor distances:

● Ax = 0 mm, Ay = -792 mm, Az = –232 mm
● Bx = 730 mm, By = 413 mm, Bz = –232 mm
● Cx = -711 mm, Cy = 413 mm, Cz = –232 mm
● Dx = 0 mm, Dy = 0 mm, Dz = 2298 mm

**Fig. 200:**
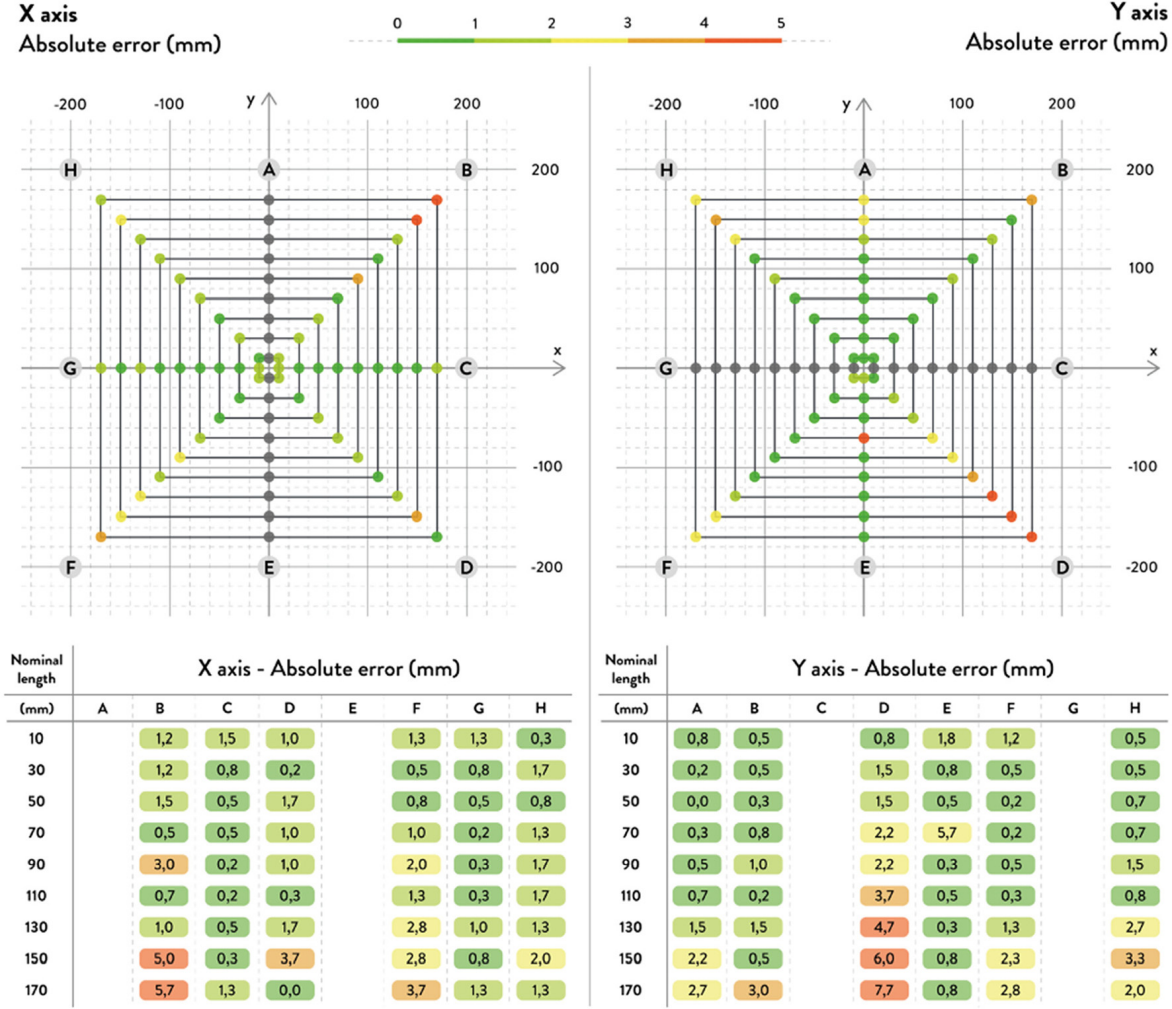
Positioning accuracy test of the Hangprinter. graphs and values of the average absolute error for the x-axis (left) and y-axis (right).

The positioning accuracy test was performed at the level of the printing bed to measure the average absolute error. As shown in Fig. 200, different absolute error values emerged in the x- and y-axis, as well as in correspondence with the same measuring points. For small and medium distances (10–110 mm), the absolute error was below 2 mm in most cases, whereas big differences occurred for longer paths (130–170 mm), reaching ∼6–8 mm. In particular, the paths of 30–50 mm show the lowest absolute error values, especially for the y-axis, ranging from 0.2 to 0.8 mm in most cases. Therefore, positioning accuracy decreases by moving away from the origin, which means the center of the printing bed. Furthermore, higher values of absolute error are visible in some areas of the printing bed, especially for the set of points B and D. In general, these results are in line with the previous work on similar systems [35] and confirm the variability of the positioning accuracy of this system for bigger areas. The general accuracy, however, is significantly lower, probably for the effects of the manual calibration and the tightening loss of the cables during the path generation.

### Tensile tests

#### Tensile specimens

**Fig. 201:**
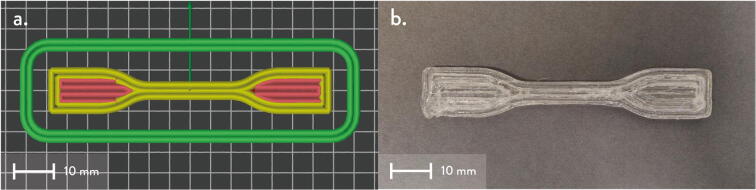
Tensile specimens. (a) gcode preview; (b) 3D printed bars.

Tensile tests were performed on a TPS-25KN universal testing machine (Adelaide Testing Machines Inc., Toronto, Ontario, Canada) machine equipped with a 25 KN cell load at a speed of 50 mm/min. Tests were carried out following the ASTM Standard Test Method D638-22, selecting the Type IV specimen with a gauge length of 33 mm, a thickness of 4 mm, and a width of 6 mm [90]. At least five specimens made of PLA were 3-D printed by using the printing speed and temperature selected in Section 7.1.3. The specimen gcode had a layer height of 0,8 mm, two perimeters, and a 100 % rectilinear infill with 50 % overlap to avoid intralayer voids (Fig. 201). The major asperities were manually sanded to have constant cross-sectional dimensions, which were then measured with a caliper. The mean and standard error values were evaluated by calculating the stress–strain curve from the values obtained in the tests.

#### Results

The samples showed a brittle behavior without yield points during the tensile tests. According to the first row of Table 29, the values of ultimate tensile strength and tensile strength at failure show no significant difference, confirming the brittle behavior of the specimens. The values show high variability, however, mainly linked to the presence of defects in the 3-D printed samples, i.e., some inner voids. These errors mainly derived from the large nozzle diameter, although some strategies were followed during the slicing to reduce its influence, i.e., increasing the infill overlap. Similarly, the thick layer height may affect these results since it represents a consequence of the nozzle dimension. Thick layers and large nozzle diameters mean a shorter extrusion path during the printing process, resulting in a bigger influence of each single extrusion inconsistency with respect to using small nozzle diameters [26]. Previous studies also confirm this issue. The experimental tensile results are comparable to the average values of elastic modulus and ultimate tensile strength (UTS) obtained by Woern et al., 2,1 GPa and 39 MPa, which used a bigger nozzle diameter compared to the other works, i.e., 1.75 mm [26].

**Table 29:** Results from the tensile tests (elastic modulus, ultimate tensile strength, and tensile strength at failure) and comparison with the main works from the literature.

**Nozzle diameter (mm)**	**Layer height (mm)**	**Elastic Modulus (GPa)**	**Ultimate Tensile Strength (MPa)**	**Tensile Strength at Failure (MPa)**	**References**
3	0.8	2.3 ± 1,1	37.2 ± 8.6	37.0 ± 8.7	Experimental tensile results this paper
1.75	0.4	2.1	39	//	[26]
0.5	0.2	3.5 ± 0.1	60.6 ± 0.4	57.7 ± 1.1	[98]
0.5	0.1	3.1 ± 0.2	//	46.4 ± 2.7	[92]
0.5	0.1	1.9 ± 1.1	44.1 ± 8.6	//	[91]
0.4	0.2	2.9 ± 0.1	38.7 ± 1.8	//	[93]
0.35	0.15	//	53.6 ± 0.8	//	[96]
//	0.1	2.5 ± 0.1	61.5 ± 1.7	//	[97]
//	//	3.1 ± 0.3	40.55 ± 1.1	//	[94]
//	0.3	1.7 ± 0.1	46.13 ± 1.7	//	[95]

Nevertheless, the results are in the range of most other works (Table 4). For example, Algarni obtained an average elastic modulus of 1.9 ± 1.1 GPa and an UTS of 44.1 ± 8.6 MPa by using a nozzle diameter of 0.5 mm and a layer height of 0.1 mm [91]. Focusing on the tensile strength, the values at failure from Cruz Sanchez et al. are comparable with the experimental tensile results obtained with the hangprinter [92]. Adrzejewska et al. and Raj et al. achieved similar ranges [93], [94], whereas slightly higher values were shown by Yao et al. and Lanzotti et al. [95], [96]. Considering the elastic modulus, Yao et al. achieved similar results with a layer height of 0.3 mm, a higher value compared to the other works [95]. Despite the higher UTS value, Brischetto and Torre obtained an elastic modulus of 2.5 ± 0.1 GPa [97], while Andrzejewska et al. of 2.9 ± 0.1 [92].

Therefore, the tensile tests on the Hangprinter demonstrated consistent results according to the nozzle diameter of its setup since higher and more accurate values were obtained with smaller nozzle diameters and layer heights, as in the work of Cruz Sanchez et al. [98]. Using the Hangprinter leads to lower values than conventional FFF 3D printing due to the different settings and setup [35], [26], especially layer height and nozzle diameter.

### Demonstration through a design case study

A further validation step is represented by using the Hangprinter for plausible applications. A case study can therefore validate the 3-D printing quality by fabricating objects and products with functional purposes. As previously seen, the Hangprinter leads to different properties and results than traditional FFF processes due to its setup and printing scale [35], [87], [88]. Nevertheless, this 3-D printing system may be used in different contexts considering its accessibility and adaptability, i.e., in building volumes and nozzle dimensions. Although some limitations currently exist in terms of dimensional accuracy for large parts, the Hangprinter may be used for the local production of small- and medium-scale products by taking advantage of the intrinsic flexibility of additive manufacturing, i.e., customization. Furthermore, this system appears as an appropriate way to implement some principles of circular economy within local communities through practical actions, i.e., repairing, reusing, refurbishing, and repurposing [99], [100]. At a later stage, recycled feedstock or waste-based materials can be selected as primary sources, encouraging recycling and upcycling paths through design products [101]. For these reasons, the demo product for this validation step belongs to the furniture sector, which previously demonstrated to be an appropriate application field for circular 3-D printed products [101], [102], [103].

**Fig. 202:**
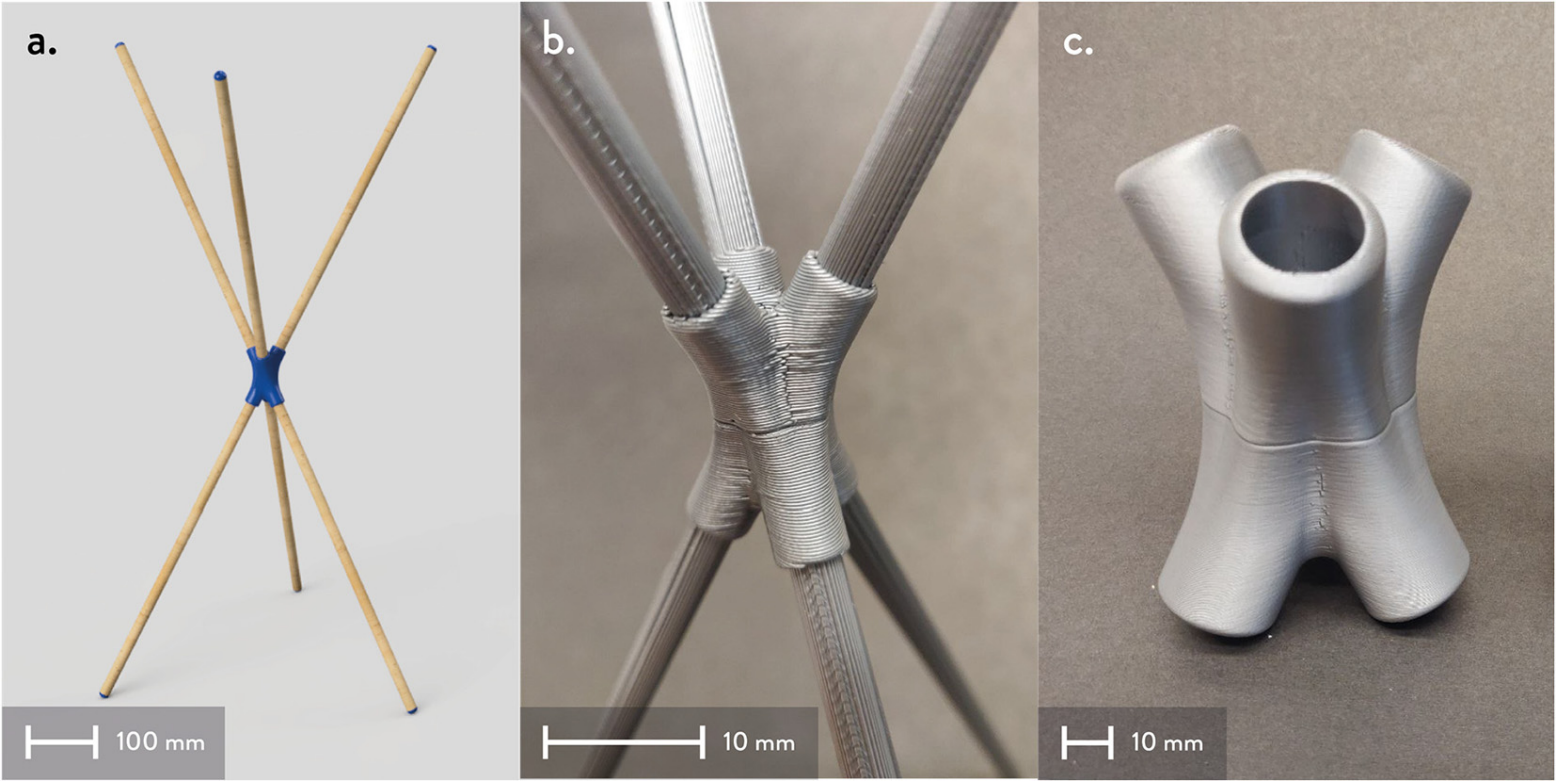
Demo product: (a) rendering of the product; (b) 1:4 prototype of the assembly, (c) 1:1 prototype of the demo part (overall dimensions: 89 × 82 × 124 mm).

The preview of the demo product is visible in Fig. 202a and Fig. 202b. This coat hanger is made of six wood dowels connected by a 3-D printed joint. This approach allows replacing some components to extend the product lifecycle, i.e., replacing some broken parts, modifying, customizing the coat hanger according to the users, or changing its function by modifying its current layout. For instance, this product may be used in learning contexts, where it could be repaired, customized, and modified to adapt to the students’ needs, i.e., during growth. In this case, the 3-D printed joint is the demo object to test the Hangprinter (Fig. 202c).

This joint was designed according to the Design for Additive Manufacturing (DfAM) principles [104]. It also aims to test the potential and limitations of the Hangprinter. In particular:

● Incremental overhangs were included to check the current limits, starting from extruded-like walls.
● Different cross-sectional shapes, dimensions, and times were designed to test the shape accuracy and the stringing effect, especially for small areas and short layer times.
● The part was designed to be part of a simple assembly to check the tolerances for its real use.
● Nozzle dimension influenced wall thicknesses and the number of perimeters used to define the length of printing paths for each layer, hence their cooling times.
● Support material was avoided to reduce material waste and for the higher difficulty in its removal due to the thick nozzle diameter and line width.
● Bridging features were limited to short paths to limit the deformations linked to longer cooling times of the extruded materials.
● High infill percentages were avoided to reduce extruder leakages during travels for the absence of retraction.

**Fig. 203:**
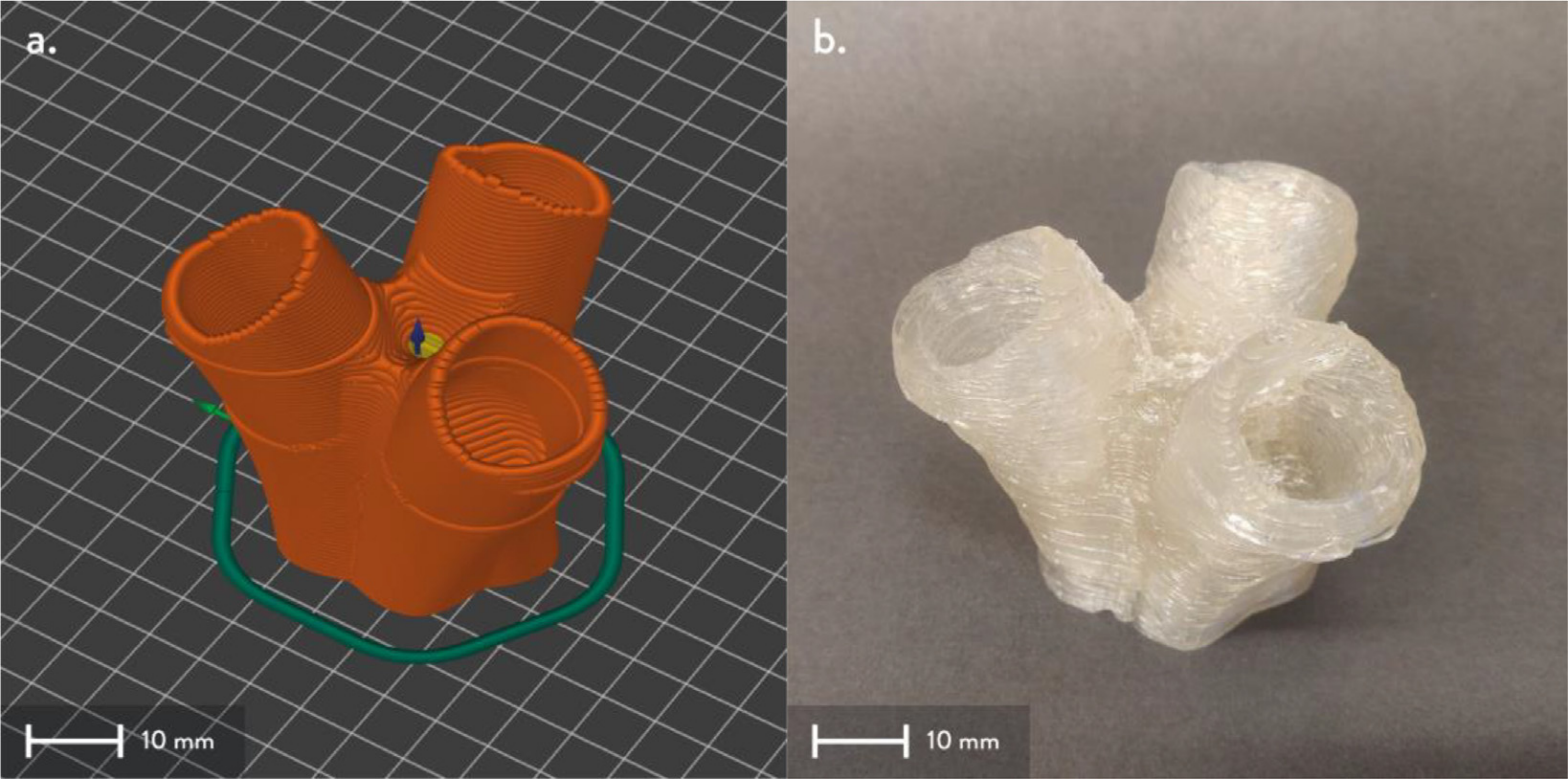
Demo product**:** (a) gcode preview, (b) 3D printed part of the joint.

Fig. 203a shows the preview of the gcode file. Slicing was made according to the optimal process parameters from the previous sections, setting a layer height of 0.8 mm, two perimeters, three bottom/top layers, and 0 % infill. In this way, the nominal printing time was less than two hours, close to the real one. A picture of the corresponding 3-D printed part is visible in Fig. 203b. Considering the previous aspects from the design phase, the incremental overhang was successfully obtained with little deformations in the upper part. The different cross-sectional areas were achieved, although some stringing occurred in the final part. Moreover, over-extrusion occurred in some areas, especially in the middle, probably due to the increasing number and length of travel paths. For these reasons, some post-processing is required to achieve the correct tolerances for assembling the wood dowels. The overall quality, however is functionally satisfactory, considering the current development of the Hangprinter, and validates its potential use for small-scale applications such as furniture elements. The following steps should pave the way to use different materials such as recycled feedstock and/or high-performing polymers, enlarging the range of possible applications, i.e., large-scale furniture and toys, as well as simple technical applications, i.e., 3-D printed molds.

### Limitations

There are several limitations of the current hybrid FGF Hangprinter design. The most notable issue from using the system for manufacturing is the need for manual calibration of anchor points and line lengths, which introduces errors in the print accuracy of Hangprinter effector due to errors in measurement. Improvements in the measurement process, such as using better instrumentation like a laser level/distance meter, can aid in reducing tolerances. In addition, there is the clear opportunity to use computer vision to create a self-calibrating Hangprinter. When the system is operating after calibration, the open loop stepper motors can skip steps under high loads and accelerations. The system also has no flex compensation in current firmware implementation so the end effector loses tension as it moves away from the print center, which reduces the accuracy at the edges of the print area as shown in the validation data. Perhaps the largest limitation of the current system is the print speed. The extruder motor stalls at > 15RPM, resulting in a reliable max flow rate of approximately 20 mm/s. To be used in high-speed production of larger objects a stronger extruder motor is needed, but this has design ramifications on the other components (e.g. cable strengths). The system does not use a compression screw that causes uneven mixing and a low-pressure buildup in the extruder resulting in inconsistent flow and air bubbles and due to the large pitch and channels depths of the auger, pellets free fall down the auger even without any rotation, causing inconsistent extrusion and possible clogs due to heat creep. The system can also jam in the hopper due to insufficient torque in extruder motor and vertical feeding of pellets. Finally, the flexible feeding tube can bend upwards, resulting in a blockage for pellets to flow.

### Future work

As can be seen from section 7.5 there is substantial future work needed to bring a FGF Hangprinter up to the same reliability as FFF 3-D printers. An advantage of this FGF Hybrid Hangprinter is that the mechanical assemblies and electronics are designed for expandability in mind. With that, one can implement solutions to the limitations discussed above. One area of improvement to explore is to make the stepper motors into closed loop servos just like the optional upgrade to the Hangprinter v3 which uses Mechaduinos on NEMA17 stepper motors [105]. One can design a controller PCB suited for the higher current NEMA23 stepper motors and integrate its existing code to make it work with the DUET3 controller. Another improvement is to implement a computer vision based autocalibration algorithm that detects the various line length components of the A, B, C and D axes and sends those values to the firmware in the DUET3. Such a process has been implemented in hp-mark [106], that works with the latest Hangprinter v4. That approach only works with its closed loop BLDC controllers, the O-drives, and cannot be used with open loop stepper motors. With a tighter calibration comes reduced print errors thus making better use of the limited 5 cycles of recycled plastic before 50 % degradation [92]. Another improvement that can speed up development time and testing is the implementation of G-code macros [107] enabled in RepRap firmware. Repetitive tasks such as homing, purging extruder, loading pellets, etc. can be performed by entering a singular command on the DWC interface. After initial testing of Hangprinter movement under load, it was determined that the Hangprinter can still handle a large weight. This opens the opportunity of using a stronger motor and overcome some of the issues listed in the limitations, most notably the printing speed.

For the pellet extruder, more work is needed to assess the capabilities of the extruder and determine optimal parameters for different types of waste feedstock (including material, shape, and distribution). A shared material library and set of parameters for different kinds of feedstocks will be beneficial for users so that they do not need to experiment from scratch, fostering the use of these layouts in real contexts. Proper calibration and consistent flowrates are dictated by the ability to address the limitations of the extruder discussed above. Improvements to the extruder to address these issues include 1) investigating alternate extrusion screws, 2) improvements to the feeder system and extruder inlet redesign and 3) adding closed loop control to extrusion. Further investigation of various off the shelf screws may lead to a more appropriate extrusion screw. A possible alternative can be using a masonry bit such as [108], which has shallower channel depth and smaller pitch. This more closely resembles an extrusion screw. Using a screw with shallower channel depths can aid in compression and pressure generation in the barrel and screw interface allowing for better mixing of the melt plastic before extruding out the die. A custom compression screw can also be used to improve extrusion characteristics and could be manufactured using an open source grinder [109]. Other pellet extruders in the academic and maker community such as the Universal Pellet Extruder [110], GigabotX Pellet Extruder [34] and even a metal filled polymer pellet extruder [111] have demonstrated the successful use of smaller scale extrusion screws without the use of complicated instrumentation involved in a traditional injection molding process. Jamming of pellets either in the hopper of a pellet extruder or barrel is a common as noted in [30], [51], [111]. This is due to the vertical feed of pellets into the hopper which causes pellets to free fall down the channel of the screw and clog. The pellet extruder implement here has a similar method of pellet feeding as [30] by treating the feeder as a 2nd extruder so pellets are released synchronously at a proportional rate. This is not the most ideal method of feeding as there is risk of pellet overflow which can adversely affect extruder performance by causing jams at the inlet. A potential fix to this may be introduced when the newest version of the RepRap Firmware releases (RRF 3.5) [112]. This update will bring asynchronous stepper motor movement which will allow the feeder systems motor to run in the background independent of the Hangprinter motors. By using an external level sensor and the firmware’s conditional G-code functionality, the feeding system will only release pellets when levels in the extruder are below a threshold. Lastly, work has also been done in adding closed loop pressure control to a pellet extruder for 3-D printing. By using sensors to measure parameters such as bead width, velocity to determine flowrate, an empirically determined extruder model can be derived from which an external PID controller can than dynamically adjust flow rates of the extruder [113]. A similar concept can be applied to this extruder and in conjunction the DUET3 controller’s nonlinear extrusion functionality.

As the FGF/FPF hangprinter has been demonstrated there are many applications in the industrial environment that may be built upon AM success in several domains including aerospace [114], robotics [115] biomedical applications [116]. At first the use of low-resolution plastic 3-D printing may not be obviously appropriate for such applications, however, there are several opportunities to make large bulk parts that do not need high-degrees of resolution. For example, an open source surgical fracture table, not only radically reduces costs from the equivalent medical technology, but also consists of many large 3-D printed structural parts that would be appropriate for such FGF/FPF hangprinting [117]. To truly meet a wider range of such high-performance applications the FGF/FPF hangprinter will need to be augmented to work with composites, fiber-reinforced thermoplastics [118] and engineering-grade and high-temperature polymers (e.g. PEEK [119], PEKK and PEI/ULTEM [120], and multi-materials [121], which can still be recycled as waste.

In summary, this machine presents an easy-to-use hardware and software foundation of a large area cable driven direct pellet of particle additive manufacturing. It provides the opportunity for large scale economic plastic recycling to reduce GHG emissions from plastic production. Along with constant support and updates from the Hangprinter and DUET3 community, the system described here provides open hardware developers a rich ecosystem to work on and modify to their needs for printing directly from waste plastic for DRAM.

## Declaration of Competing Interest

The authors declare that they have no known competing financial interests or personal relationships that could have appeared to influence the work reported in this paper.

## Appendix A

Design files: https://osf.io/wpcf7/
